# Meta-analyzing correlation matrices in the presence of hierarchical effect size multiplicity

**DOI:** 10.1017/rsm.2025.10027

**Published:** 2025-08-07

**Authors:** Ronny Scherer, Diego G. Campos

**Affiliations:** 1 Centre for Educational Measurement (CEMO), Faculty of Educational Sciences, University of Oslo, Oslo, Norway; 2 Centre for Research on Equality in Education (CREATE), Faculty of Educational Sciences, University of Oslo, Oslo, Norway

**Keywords:** correlation matrix, effect size multiplicity, meta-analytic structural equation modeling, multilevel meta-analysis, multivariate meta-analysis

## Abstract

To synthesize evidence on the relations among multiple constructs, measures, or concepts, meta-analyzing correlation matrices across primary studies has become a crucial analytic approach. Common meta-analytic approaches employ univariate or multivariate models to estimate a pooled correlation matrix, which is subjected to further analyses, such as structural equation modeling. In practice, meta-analysts often extract multiple correlation matrices per study from various samples, study sites, labs, or countries, thus introducing hierarchical effect size multiplicity into the meta-analytic data. However, this feature has largely been ignored when pooling correlation matrices for meta-analysis. To contribute to the methodological development in this area, we describe a multilevel, multivariate, and random-effects modeling approach, which pools correlation matrices meta-analytically and, at the same time, addresses hierarchical effect size multiplicity. Specifically, it allows meta-analysts to test various assumptions on the dependencies among random effects, aiding the selection of a meta-analytic baseline model. We describe this approach, present four working models within it, and illustrate them with an example and the corresponding R code.

## Highlights

### What is already known?

- Meta-analyzing correlation matrices allows meta-analysts to test theories and hypotheses on the relations between multiple variables.
- Pooling correlation matrices meta-analytically across studies often relies on univariate random-effects models or multivariate random-effects models (MV-REMs).
- Hierarchical effect size multiplicity can arise when meta-analysts extract multiple correlation matrices from a study, often due to the inclusion of multiple samples, study sites, or countries.
- Existing pooling approaches either largely ignore hierarchical effect size multiplicity or only partially account for it.

### What is new?

- Multilevel extensions of MV-REMs provide a framework for addressing hierarchical effect size multiplicity in the meta-analytic pooling of correlation matrices.
- Dependencies among correlations within correlation matrices can be specified in the asymptotic sampling covariance matrices.
- Multilevel, multivariate, and random-effects models (MLMV-REMs) enable meta-analysts to model the heterogeneity in correlation matrices within and between studies.
- These models can be estimated using the multilevel and multivariate features in the R package “metafor.”

### Potential impact for RSM readers

- Meta-analysts who wish to synthesize correlation matrices in the presence of hierarchical effect size multiplicity may take an MLMV-REM approach.
- We aim to encourage the development and evaluation of models addressing effect size multiplicity in the meta-analytic pooling of correlation matrices.
- We offer an illustrative example along with the corresponding analytical R code that meta-analysts can adapt to their research.

## 1 Introduction

Understanding how multiple constructs, concepts, or variables are related and testing theories that describe these relations requires the synthesis of *multiple* correlation coefficients.^1^ Multiple correlation coefficients are often stored in correlation matrices, and synthesizing them across primary studies means to pool the correlation coefficients via meta-analytic methods into an overall, weighted average correlation matrix.^2^ Notably, correlation coefficients within a correlation matrix are not independent, as they are usually derived from the same sample. Given this correlational dependence (“correlational effect size multiplicity”), the pooling of correlation matrices often involves multivariate models that provide more accurate meta-analytic estimates and standard errors than multiple, separate univariate models.^3^ ^,^ ^4^

With the growing availability of published studies and data, multiple independent samples, study sites, or countries may be included per primary study. This scenario is referred to as “hierarchical effect size multiplicity.”^5^ For instance, in their study of the relations among technology acceptance variables, Scherer et al.^6^ extracted multiple correlation matrices from primary studies, because some studies included more than one teacher sample (e.g., in-service and pre-service teachers). The resultant meta-analytic data comprised multiple correlation matrices that were hierarchically nested in studies.^7^

While dealing with hierarchical effect size multiplicity has been well described for *univariate* outcomes (e.g., via multilevel random-effects models^8^ or cluster-robust variance estimation^9^ of one correlation coefficient), it has received less attention for *multivariate* outcomes, especially when correlation matrices are pooled. Similar to a univariate scenario, ignoring hierarchical effect size multiplicity when meta-analyzing multivariate data may result in biased standard errors and heterogeneity estimates.^10^ ^,^ ^11^ In our view, existing approaches to pool correlation matrices meta-analytically largely ignored hierarchical effect size multiplicity or addressed it only to a limited extent.^10^ For instance, pooling correlation matrices across studies—as a key stage in meta-analytic structural equation modeling (SEM)—is often based on multivariate random-effects models (MV-REMs) assuming that correlation matrices are fully independent. Acknowledging this limitation, Wilson et al.^12^ developed a multilevel random-effects modeling approach—referred to as the “WPL approach”—that quantifies within- and between-study heterogeneity of the correlation coefficients within correlation matrices. However, this approach assumes that within- and between-heterogeneity are the same across correlations—an assumption that may or may not hold in practical applications.

To address these limitations, we present a multilevel, multivariate, and random-effects modeling (MLMV-REM) approach for pooling correlation matrices in the presence of hierarchical effect size multiplicity. Extending the existing approaches, the MLMV-REM approach allows meta-analysts to flexibly specify random-effects structures within and between studies, obtain information about correlation-specific heterogeneity, and examine moderator effects. In this tutorial paper, we describe the approach, highlight the key analytical decisions within, present four working models, and illustrate its application with an example, including the respective R code. Despite its flexibility, we hope to stimulate future evaluations of the MLMV-REM approach and to advance the development of approaches that deal with effect size multiplicity when pooling correlation matrices meta-analytically.

## 2 Existing approaches to pooling correlation matrices meta-analytically

In this section, we explain the different types of dependencies among correlation coefficients that may occur when meta-analyzing correlation matrices and review univariate and multivariate pooling approaches. While various methods have been developed to pool correlation matrices meta-analytically, each presents specific limitations. These shortcomings highlight the need for a more flexible and comprehensive method that can address both the correlational and hierarchical dependencies that may occur in meta-analytic data, ensuring more accurate and reliable meta-analytic conclusions.

### 2.1 Dependencies among effect sizes

While pooling correlation matrices across studies has gained popularity in meta-analysis, it still faces significant challenges. One of these challenges refers to effect size multiplicity that occurs due to the dependencies among effect sizes both within and across studies.^10^ ^,^ ^12^ ^,^ ^13^ Cheung^3^ summarized effect size dependencies as (a) dependencies among the sampling errors that occur due to the administration of multiple measures to the same sample in each study, (b) dependencies among true effect sizes at the population level across studies, and (c) dependencies due to hierarchies in the data, such as multiple correlation coefficients that are nested in studies.

In the context of pooling correlation matrices meta-analytically, such dependencies can occur not only because multiple correlation coefficients are stored in correlation matrices, but also because multiple correlation matrices are extracted from one study. For example, in a meta-analysis of psychological traits—such as anxiety, depression, and stress—studies might report multiple correlation matrices for samples defined by different clinical statuses (e.g., clinical vs. non-clinical samples). In another meta-analysis, authors may extract correlation matrices describing the relations among student achievement, motivation, and engagement in reading, mathematics, and science, using data from international large-scale assessments in education, such as the Programme for International Student Achievement or the Trends in Mathematics and Science Study.^7^ ^,^ ^14^ Given the inclusion of several countries in these assessments, multiple correlation matrices are available per assessment cycle.

These examples illustrate that dependencies among multiple correlation coefficients may not only exist within a correlation matrix, because multiple correlations were derived from the same sample, but also across multiple constructs or measures (i.e., correlational effect size multiplicity).^5^ The inclusion of multiple correlation matrices per study that are derived from independent samples also creates hierarchical effect size multiplicity.^15^

Failing to account for such dependencies can bias meta-analytic results, lead to inaccurate pooled estimates, and compromise statistical inferences.^5^ ^,^ ^15^ ^,^ ^16^ Several statistical approaches have been proposed to address effect size multiplicity when pooling correlation matrices meta-analytically, which we describe briefly in the next sections. Notably, we exclude approaches that ignore effect size multiplicity—be it the fact that multiple correlation coefficients are stored in correlation matrices or multiple correlation matrices are available per study. For an overview of these approaches, we kindly refer readers to Stolwijk et al.^10^

### 2.2 Univariate approaches

Univariate approaches pool each correlation coefficient within a correlation matrix separately across studies via a series of standard, univariate, fixed- or random-effects models.^1^ ^,^ ^3^ ^,^ ^17^ For instance, taking a univariate approach, the correlation between anxiety and depression would be meta-analyzed independently of the correlations between anxiety and stress and between stress and depression. In situations where multiple correlations between the same constructs or measures are available due to the inclusion of multiple samples, univariate approaches can account for this hierarchical effect size multiplicity via multilevel random-effects models. Such models estimate the variation of correlation coefficients within and between studies and allow meta-analysts to explore possible causes of this heterogeneity.^18^ ^,^ ^19^ However, this approach neglects the inherent multivariate structure of correlation matrices, where multiple correlations share common constructs and are derived from the same sample (i.e., correlational effect size multiplicity). Ignoring this dependence among correlations within matrices leads to an underestimation of standard errors and, consequently, inflated Type I error rates.^20^ ^,^ ^21^ Hence, univariate approaches are not recommended for synthesizing correlation matrices with or without hierarchical effect size multiplicity.^4^

### 2.3 Multivariate approaches

Multivariate approaches can account for dependencies among correlation coefficients within studies, which arise from measuring multiple outcomes on the same sample.^22^ ^,^ ^23^ These models incorporate not only the sampling variances but also some information about the covariances of correlation coefficients, allowing for the estimation of a pooled correlation matrix that reflects the interrelationships between multiple outcomes.^4^ By simultaneously leveraging all available correlations, multivariate approaches “borrow strength,” for instance, from the within-study correlations, leading to more precise estimates of mean effect sizes and more reliable assessments of between-study heterogeneity.^21^ ^,^ ^24^ ^,^ ^25^ Moreover, multivariate approaches often result in joint confidence intervals and distributions and allow meta-analysts to predict an effect controlling for another, correlated effect.^25^ However, the application of multivariate approaches in practice is often constrained by the limited availability of information about sampling covariances or correlations in primary studies, and estimation problems may occur due to the increased number of parameters and their joint distributions as compared to separate univariate meta-analyses.^25^ ^,^ ^26^ In the following section, we briefly describe some multivariate approaches to pooling correlation matrices meta-analytically.

#### 2.3.1 Generalized least squares approach

Becker^23^ ^,^ ^27^ developed a multi-step generalized least squares (GLS) approach, in which multiple correlation coefficients can be synthesized while accounting for their dependencies. Specifically, in this approach, the correlation coefficients and sampling variances are extracted from the primary studies, and sampling covariances are estimated for each pair of correlation coefficients. Using the sampling covariance matrix and between-study heterogeneity estimates for each correlation coefficient to obtain weights under a random-effects assumption, a weighted average, that is, a pooled correlation matrix, is then obtained.^27^ While the GLS approach can handle the dependencies among multiple correlations within correlation matrices, it does not account for hierarchical effect size multiplicity when multiple correlations are available for the same constructs across different samples within studies. It would likely need a multilevel extension of the step in which heterogeneity variances are estimated and incorporated in the weights.

#### 2.3.2 Multivariate, random-effects modeling approach

Given that correlation matrices within studies contain multiple correlation coefficients from the same sample, dependencies among these effect sizes occur (i.e., correlational effect size multiplicity). A multivariate, random-effects modeling (MV-REM) approach can handle these dependencies and overcome issues associated with estimating multiple, separate, univariate random-effects models, such as the risk of bias and reduced precision.^3^ ^,^ ^21^ Specifically, MV-REMs decompose multiple observed correlation coefficients into true effect sizes, random effects, and sampling error. Given the multivariate nature of the data, the sampling covariance matrix of a study does not only contain the sampling variances in the diagonal but also nonzero sampling covariances in the off-diagonal.^3^ These covariance matrices are study-specific, so that two primary studies may show different degrees of dependencies. At the population level, the dependencies among effect sizes are modeled by the structure of the random effects, that is, the structure of the heterogeneity covariance matrix. In this matrix, the between-study heterogeneity variances are stored in the diagonal, and the off-diagonal represents the assumptions on the dependencies at the population level. Several structures have been proposed (e.g., unstructured, diagonal, or compound symmetric [CS] matrices),^3^ ^,^ ^28^ and we will describe them in greater detail in Section 3. Overall, while the MV-REM approach handles correlational effect size multiplicity within correlation matrices, it does not consider hierarchical effect size multiplicity. Hence, taking this approach, meta-analysts are limited to meta-analytic data in which each study contributes one correlation matrix.

#### 2.3.3 Structural equation modeling approach

To synthesize correlation matrices across studies, Cheung^29^ translated multivariate meta-analysis into the SEM framework. In this framework, the vector of correlation coefficients and their variance components are modeled as mean and covariance structures in SEM. This approach follows two steps^3^ ^,^ ^30^: In the first step, researchers obtain the sampling variance–covariance matrix for each study via confirmatory factor analyses. In the second step, the vector of correlation coefficients is pooled via an MV-REM that also incorporates the sampling covariance matrices. In addition to a pooled correlation matrix, a between-study heterogeneity covariance matrix is estimated. The SEM approach offers several advantages, including the ability to handle missing correlation coefficients in some primary studies. Specifically, given that the meta-analytic models used to synthesize correlation matrices are translated into an SEM framework, the Full-Information Maximum-Likelihood procedure is available to handle missing correlations in the primary studies’ correlation matrices.^3^ This way, all available information from the correlation matrices is used to estimate a pooled correlation matrix and the respective sampling covariance matrix. In the pooling stage, the data are often vectorized, and missing entries in these vectors are kept. This procedure is more efficient than listwise deletion, in which correlation matrices with missing values are deleted.^4^ ^,^ ^31^

Despite its utility in accounting for the dependencies among correlation coefficients within a correlation matrix (correlational effect size multiplicity), the SEM approach is less applicable when multiple correlation matrices are reported for independent samples within a study (hierarchical effect size multiplicity), because it allows each study to contribute only one correlation to each cell of the synthesized correlation matrix.^12^ Hence, this approach is constrained by its assumption of independence between correlation matrices across studies.^10^

#### 2.3.4 Wilson–Polanin–Lipsey approach

The three-level random-effects modeling approach, proposed by Wilson et al.,^12^ addresses both correlational and hierarchical effect size multiplicity when pooling correlation matrices. This approach includes information from all available (dependent) effect sizes per study and explicitly models these dependencies in the pooled correlation matrix. Furthermore, it estimates both within- and between-study variances, allowing for the examination of heterogeneity at different levels (Cheung^16^). In essence, the WPL approach is based on a multivariate, multilevel, and random-effects model, which yields a pooled correlation matrix, a within-study variance estimate, and a between-study variance estimate.^10^ ^,^ ^12^ However, the WPL approach assumes that these heterogeneity variance estimates are the same for all correlation coefficients. This assumption may not hold in practice, especially when the correlation coefficients represent associations among diverse constructs that may vary substantially across studies. In such a situation, meta-analysts may expect different amounts of heterogeneity across correlation coefficients. Overall, the three-level random-effects approach by Wilson et al. addresses both correlational and hierarchical dependencies, but its simplifying assumption about within- and between-study heterogeneity limits its utility.

## 3 A multilevel, multivariate, and random-effects modeling approach

When multiple, independent samples provide correlation matrices, both correlational and hierarchical effect size multiplicity exist. One approach to account for these multiplicities is to model the dependencies among multiple correlations within a correlation matrix via a *multivariate* component and to model the hierarchical nesting of multiple, independent correlation matrices in primary studies via a *multilevel* component. This approach can be based on MLMV-REMs^32^ that systematically extend the existing multivariate approaches (e.g., the WPL and MV-REM approaches).

### 3.1 Specification of the MLMV-REM

Suppose we have a meta-analytic dataset that comprises correlation coefficients as effect sizes and their respective sampling variances.^i^ Let 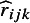 denote the estimate of the *i*th correlation coefficient in the *j*th correlation matrix (or, respectively, the *j*th sample) extracted from the *k*th primary study. Given this terminology, the *j*th correlation matrix within the *k*th primary study that contains *I* correlation coefficients can then be written as 
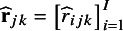
 .

A generic version of an MLMV-REM can be specified by distinguishing between three levels of analysis. At Level 1, the effect size estimates 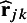 are decomposed into true effect size 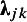 and sampling error^19^:

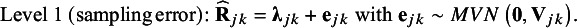



Given the multivariate nature of the data (i.e., multiple correlations within a correlation matrix due to multiple outcomes or constructs of interest), the sampling variances 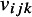 are stored in the diagonal of the sampling covariance matrices 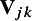 , and possible covariances among the sampling correlation coefficients are stored off the diagonal. 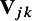 take the following symmetric form:

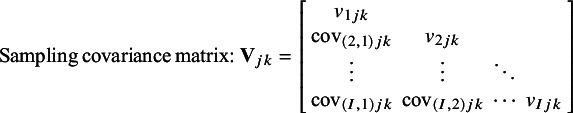



To access the sampling covariances 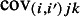 for the 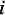 th and 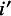 th variables, several solutions have been proposed.^ii^ Wei and Higgins^33^ described a strategy in which the available information about the correlations among effect sizes is meta-analyzed, and the resultant, pooled correlation coefficient is then used to impute the correlations for studies or matrices that do not provide this information. Another solution is to specify values of the correlations among effect sizes, either informed by existing research or estimated correlations among constructs from the primary study data and then derive the resultant sampling covariances. However, oftentimes, this approach requires restrictive assumptions on correlations between effect sizes, such as the assumption of a constant sampling correlation.^5^ Cheung^3^ suggested to translate the meta-analytic models that pool the correlation matrices into the framework of SEM, which makes available an asymptotic sampling covariance matrix. Olkin and Finn^34^ reviewed the explicit expressions for constructing the covariances between two correlation coefficients that were proposed by Olkin and Siotani^35^, who derived them based on large-sample theory. Specifically, skipping the abovementioned indices 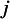 and 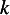 for simplicity, for variables 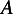 , 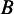 , 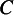 , 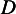 , and 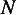 representing the sample size, the asymptotic covariance between the correlation coefficients 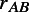 and 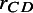 is^34^


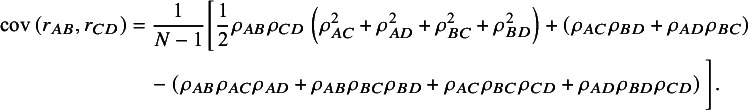



Conversely, the asymptotic covariance between 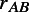 and 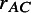 —two correlation coefficients sharing variable 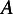 —is^34^


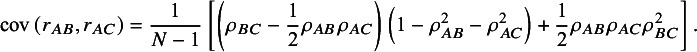



Finally, the asymptotic sampling variance of a correlation coefficient 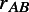 reduces to^17^


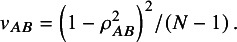



Similar equations have also been derived for Fisher- 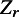 transformed correlations coefficients.^27^ ^,^ ^36^ Irrespective of the choice for an approach to approximate or construct the sampling covariance matrix, the Level 1 specification of the MLMV-REM is the first instance in which meta-analysts must decide on how to represent the effect size multiplicity within correlation matrices.

To further address the nesting of multiple correlation matrices (or samples) in primary studies, two additional levels of analysis are specified. Level 2 contains information about the within-study heterogeneity of correlation coefficients, and Level 3 contains information about the between-study heterogeneity of correlation coefficients.^8^ ^,^ ^18^ ^,^ ^19^ The decomposition of effect sizes and the respective assumptions can be specified as follows:

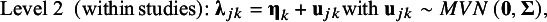




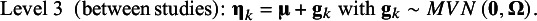



In this specification, the vector of correlation coefficients 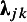 is decomposed into an average study-level vector of correlations 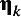 and its matrix- or sample-specific deviations from it, 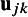 . At Level 2, these deviations may exhibit within-study heterogeneity, which is stored in the respective heterogeneity matrix 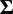 . Accordingly, 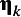 is then further decomposed into an overall effect size estimate 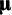 and the study-specific deviations from it, 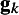 . At Level 3, these deviations may exhibit between-study heterogeneity, which is stored in the respective heterogeneity matrix 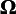 . Combining all levels of analysis results in the following overall model specification:

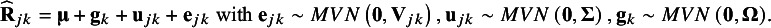



This MLMV-REM allows meta-analysts to quantify correlation-specific heterogeneity within and between primary studies (see Model 1 in Table 1). In this context, “correlation-specific” means that the parameters are estimated for each of the correlations within a correlation matrix. For instance, if meta-analysts wish to synthesize the correlations among four variables, then each correlation matrix contains six correlations off the diagonal, which represent the associations among the six pairs of variables. In our specification of the MLMV-REM, each of these six types of correlations has their own estimates of the within- and between-study heterogeneity variances. In the next section, we describe a range of structures that the heterogeneity matrices 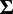 and 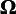 may have.

**Table 1:** Specification of meta-analytic working models with the MLMV-REM approach

Working	Effect	Within-study	Between-study	Random-effects	Example specification in metafor
model	sizes	heterogeneity	heterogeneity	correlations	
Model 1	Correlation-specific	Correlation-specific, HCS structure	Correlation-specific, HCS structure	Fixed 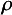 and 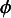	rma.mv(yi = Correlation, V = V, data = tas20, random = list(~ factor(Cell) | ESID, ~ factor(Cell) | StudyID), method = “REML,” struc = c(“HCS,” “HCS”), rho = 0, phi = 0, mods = ~ factor(Cell) − 1)
Model 2	Correlation-specific	Correlation-specific, HCS structure	Constrained, CS structure	Fixed 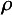 and 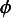	rma.mv(yi = Correlation, V = V, data = tas20, random = list(~ factor(Cell) | ESID, ~ factor(Cell) | StudyID), method = “REML,” struc = c(“HCS,” “CS”), rho = 0, phi = 0, mods = ~ factor(Cell) − 1)
Model 3	Correlation-specific	Constrained, CS structure	Correlation-specific, HCS structure	Fixed 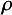 and 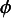	rma.mv(yi = Correlation, V = V, data = tas20, random = list(~ factor(Cell) | ESID, ~ factor(Cell) | StudyID), method = “REML,” struc = c(“CS,” “HCS”), rho = 0, phi = 0, mods = ~ factor(Cell) − 1)
Model 4	Correlation-specific	Constrained, CS structure	Constrained, CS structure	Fixed 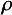 and 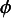	rma.mv(yi = Correlation, V = V, data = tas20, random = list(~ factor(Cell) | ESID, ~ factor(Cell) | StudyID), method = “REML,” struc = c(“CS,” “CS”), rho = 0, phi = 0, mods = ~ factor(Cell) − 1)

*Note.* The variable “Cell” is defined as a factor indicating the different types of correlations in the correlation matrix (e.g., “I2–I4” and “I11–I12”). “Correlation-specific” means that the parameters are estimated for each of the 10 correlations. “Constrained” means that the parameter is constrained to be equal across the types of correlations. “Correlation” contains the extracted Pearson correlation coefficients. “V” represents the sampling covariance matrix. “Cell” represents a nominal variable of the names of the different types of correlations. The random-effects correlations 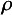 and 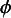 refer to the correlations among random effects within and, respectively, between studies. These correlations were fixed to zero here. “HCS” specifies a heteroscedastic compound symmetric (CS) structure of the random effects, while “CS” specifies a CS structure.^28^

The MLMV-REM approach can be further extended to a mixed-effects meta-regression model by adding explanatory variables, such as sample, study, publication, or measurement characteristics. In such an extension, 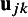 and 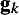 may become residuals in a regression sense, depending on the level at which the explanatory variables are defined.^37^ Moreover, heterogeneity could be explained specifically for each correlation coefficient at two levels of analysis (i.e., within and between studies). This entails that multiple measures of variance explanations are accessible for each correlation coefficient, once explanatory variables are added to the MLMV-REM (i.e., 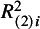 and 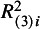 for each of the 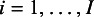 ) correlations).^3^ ^,^ ^38^

Finally, the MLMV-REM approach has the ability to pool correlation matrices with partially missing correlations using likelihood estimation. In this approach, missing correlation coefficients are typically omitted from the likelihood calculation, while all available correlations for each study are retained.^39^ ^,^ ^40^ Thus, rather than imputing unreported values or excluding entire studies, each unobserved correlation simply contributes no data to the joint likelihood, and only the observed correlations are modeled. This effectively results in pairwise deletion at the level of individual correlation coefficients. However, unlike naive pairwise methods that estimate each correlation separately, a joint multivariate model “borrows strength” across the various outcome measures and shared study-level effects, thereby leveraging the observed correlations from each study to improve the estimation of the weighted average correlations.^24^ ^,^ ^41^

### 3.2 Specification of the Level 2 and Level 3 random effects

As noted earlier, heterogeneity can be modeled with the MLMV-REM both within and between studies for each type of correlation. Specifically, the symmetric heterogeneity matrices 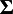 and 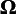 store the correlation-specific heterogeneity variances in the diagonal and the heterogeneity covariances in the off-diagonal. For *I* correlation coefficients, these matrices can take the following, often referred to as “unstructured” forms:

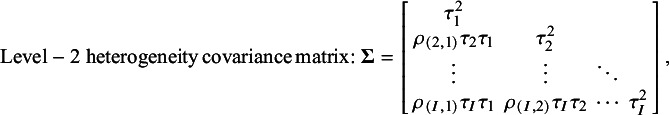




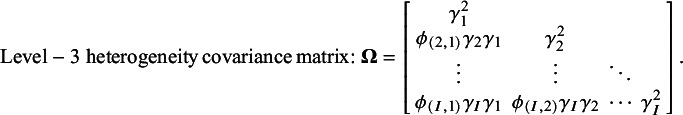



These random-effects covariance matrices contain the within-study variances 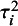 , the between-study variances 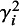 for each correlation, and the covariances that are composed of the within- or between-study standard deviations, 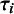 and 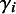 , and the correlations among the within- or between-level random effects of the correlation coefficients, 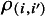 and 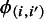 , for 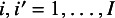 .

This form of 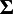 and 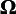 is “unstructured” and allows meta-analysts to include the covariances among random effects that are specific to each pair 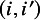 of correlation coefficients. However, although the specification of this form has a large degree of flexibility, meta-analysts may not be able to estimate all parameters within the heterogeneity covariance matrices. For instance, the likely small number of available correlations and primary studies could lead to unstable variances and small statistical power of the respective estimates. Moreover, information about the correlations among the random effects within and between studies may be lacking. Hence, meta-analysts often must simplify the structures of 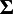 and 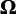 . We would like to highlight two approaches to simplifying the heterogeneity matrices. The first approach concerns the correlations among the random effects, 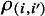 and 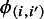 . Like the specification of the sampling covariance matrices 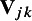 , meta-analysts could fix 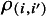 and 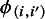 to some nonzero constant values, 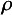 and 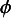 , that are the same for each random effect or approximate them in some way. The structure with constant random-effects correlations is referred to as the “heteroscedastic compound symmetry (HCS)” structure. If 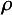 or 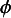 are fixed to zero, then the matrices 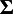 and 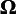 have a diagonal structure—a special case of the HCS structure in which meta-analysts can model only the within- and between-study heterogeneity variances and assume that random effects at Levels 2 and 3 are independent. This diagonal structure is often used in the first stage of MASEM, in which multiple correlation matrices are pooled via an MV-REM.^3^ The heterogeneity matrices with an HCS or diagonal structure are as follows:

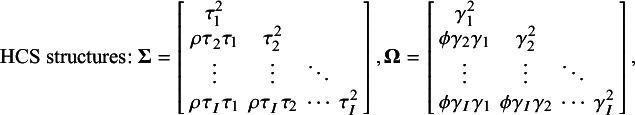




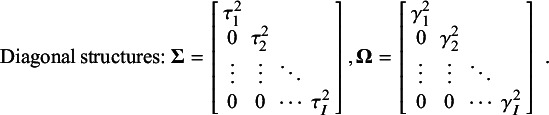



Notably, these structures contain heterogeneity variances that are specific to the random effects at Levels 2 and 3 and thus to the correlation coefficients. In other words, for each correlation coefficient (i.e., effect size), within- and between-study variance components are estimated.

A second approach to simplifying 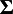 and 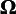 is to constrain these variance components to be equal across random effects. Imposing this constraint to an HCS structure results in a so-called “CS” structure. In the special case where 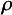 or 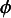 are fixed to zero, the structure is that of a “scaled identity” structure.^28^ Notably, the term “scaled identity structure” is based on the fact that the two heterogeneity matrices have the forms 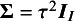 and 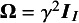 , where 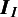 represents an 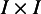 identity matrix:

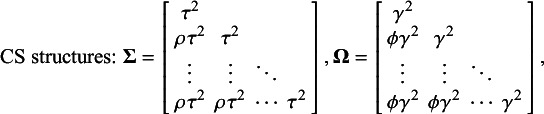




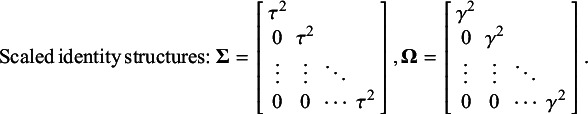



If meta-analysts choose one of the structures that assume fixed correlations among random effects, 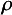 or 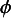 , in our view, they should examine the extent to which the meta-analytic findings and inferences are sensitive to the choice of these fixed values.^9^ ^,^ ^19^

### 3.3 Decision scheme and working models

#### 3.3.1 Analytical decisions

**Figure 1:**
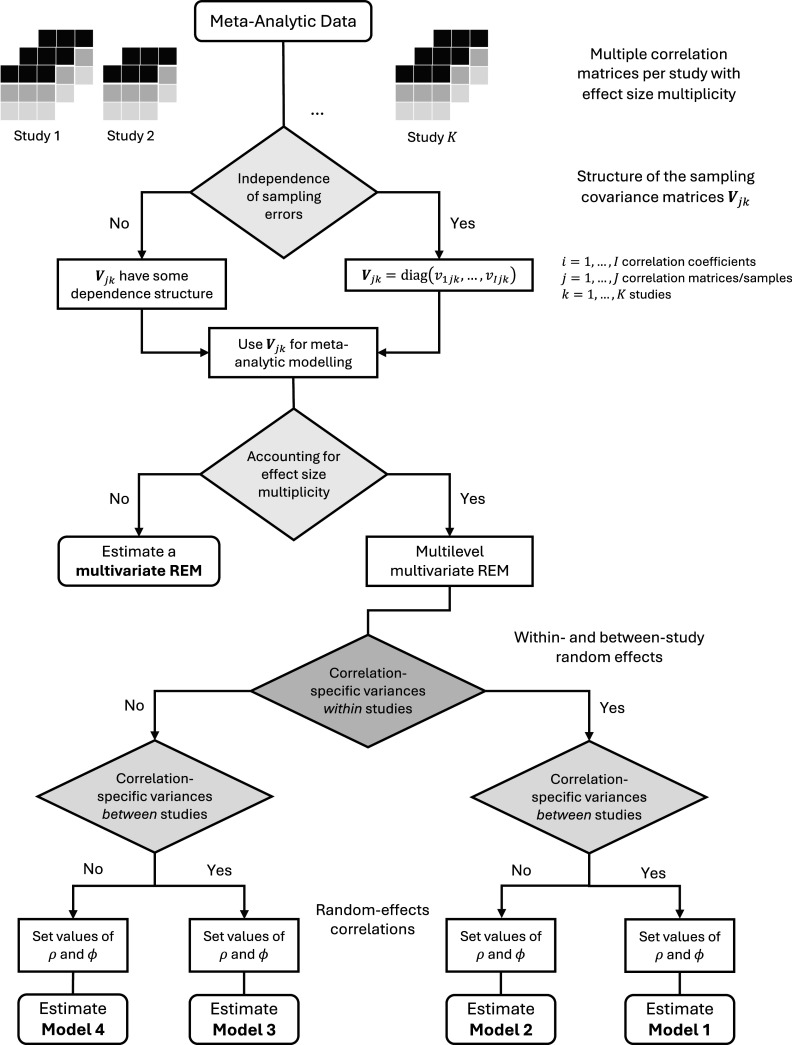
Decision scheme for selecting a working model.

To address hierarchical effect size multiplicity when pooling correlation matrices, meta-analysts must make several analytic decisions. Figure 1 provides an overview of these decisions and their possible outcomes. First, when meta-analysts have extracted the correlation matrices and the respective correlation coefficients from the primary study data, they must decide on the structure of the sampling covariance matrices 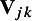 . Specifically, assuming that correlations within correlation matrices/samples are independent results in diagonal sampling covariance matrices that essentially only contain the sampling variances of the correlation coefficients. Alternatively, meta-analysts could assume some dependence among the sampling errors and then specify 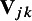 with sampling covariances off the diagonals. In scenarios where multiple measures of the same sample have been taken over time or multiple, correlated constructs were assessed for each sample, a dependency structure with some correlation among sampling errors is conceptually reasonable and preferable over an independence structure.^3^ ^,^ ^5^ ^,^ ^19^ The extracted correlation coefficients 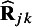 and sampling covariance matrices can then be submitted to meta-analytic modeling.

Second, if hierarchical effect size multiplicity is present in the meta-analytic data, meta-analysts must decide if they wish to account for it. In case they wish to ignore effect size multiplicity—for instance, due to close-to-zero within-study heterogeneity in the correlation coefficients—they may choose an MV-REM to pool the correlation matrices.^1^ ^,^ ^10^ ^,^ ^13^ To model hierarchical effect size multiplicity, meta-analysts may choose a working model within the MLMV-REM approach (see the following section).

Third, meta-analysts must decide on the structures of the within- and between-study heterogeneity matrices. Specifically, do they allow for correlation-specific within- and between-study variances in correlation coefficients, and which assumption do they make on the random-effects correlations within and between studies (i.e., independence assumption with 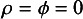 or dependence assumptions with some nonzero values of 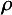 and 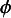 )? While the former decision determines which of the four working models is selected, the latter decision must be made for any model that has been selected.

#### 3.3.2 Selection of a working model

Table 1 provides an overview of four working models within the MLMV-REM approach, which estimate weighted average effect sizes and the within- and between-study variance components and assume constant correlations among the Level 2 and Level 3 random effects. Model 1 allows the heterogeneity estimates at Levels 2 and 3 to be specific to each correlation coefficient. Hence, 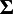 and 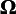 may simplify to an HCS or a diagonal structure. Model 2 estimates correlation-specific within-study heterogeneity variances but constrains the between-study heterogeneity variances to be equal across correlation coefficients. Hence, 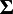 follows an HCS or a diagonal structure, while 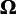 follows a CS or scaled identity structure. This model assumes that the correlation coefficients have the same amount of heterogeneity between (yet not within) studies. Conversely, Model 3 estimates correlation-specific between-study heterogeneity and constrains the within-study heterogeneity to be equal across correlation coefficients. Finally, Model 4 imposes the equality constraints on both the within- and between-study heterogeneity variances. In this model, 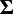 and 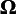 both have a CS or scaled identity structure. Notably, this model is similar to the meta-analytic model underlying the WPL approach.^12^

This selection of working models shows that meta-analysts have several options to specify and simplify similar or different Level 2 and Level 3 variance structures (see Table 1). We chose Models 1–4 as working models because, in our view, they are relevant for meta-analytic modeling practice for several reasons. First, these models allow meta-analysts to estimate correlation-specific heterogeneity variances. The information about the extent to which specific correlations within correlation matrices vary within and between studies identifies the levels at which heterogeneity is located and could be explained by potential moderators.

Second, as noted earlier, the oftentimes lacking or limited information about the correlations among the Level 2 and Level 3 random effects in a meta-analysis may not allow meta-analysts to estimate these correlations for each correlation coefficient separately. Hence, fixing these random-effects correlations to some constant value represents a pragmatic solution to still accounting for random-effects dependencies despite the lack of information about their extent.^5^

Third, the constant random-effects correlations 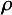 and 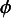 can also be fixed to zero, assuming the independence of random effects at Levels 2 and 3. This assumption is sometimes made when meta-analysts are only interested in estimating the heterogeneity variances.^3^ ^,^ ^4^ However, in this situation, the dependencies among the Level-2 and Level-3 random effects are not accounted for and may introduce some bias to the meta-analytic estimates and their standard errors.

Fourth, in Working Models 2–4, the within- and/or between-study heterogeneity variances are constrained to be equal across correlation coefficients. While these models make a strong and oftentimes unrealistic assumption about the heterogeneity in the meta-analytic data, they are still useful for meta-analytic practices. Specifically, comparing a model with such equality constraints to Working Model 1 allows meta-analysts to test hypotheses on the similarity or differences in heterogeneity variance estimates across the correlation coefficients.^3^ The constraints in Models 2–4 offer meta-analysts ways to simplify the meta-analytic models and thus strive for model parsimony.

Fifth, Models 1–4 represent several substantive assumptions that may represent meta-analysts’ theories and hypotheses on the heterogeneity in the meta-analytic data. Model 1 represents the most flexible model among the working models and is useful in situations where the correlation-specific effect sizes and heterogeneity variances are in meta-analysts’ key interest. Moreover, Model 4 may also be useful to model meta-analytic data that comprised highly heterogeneous samples within primary studies of different characteristics. Model 2 assumes that correlation coefficients can vary within studies to a different extent and between studies to the same extent. This model could be useful for meta-analytic data that comprised highly heterogeneous samples within primary studies that follow the same study protocol (e.g., multilab studies). Conversely, Model 3 might be useful for modeling meta-analytic data with highly homogeneous samples within studies but with substantial differences in study characteristics (e.g., different sampling protocols). Model 4 may be useful in situations where (a) meta-analysts wish to account for heterogeneity both within and between studies yet do not have a large-enough meta-analytic database to model correlation-specific heterogeneity variances; (b) within- and between-study heterogeneity variances are homogeneous (e.g., consistently close to zero across all correlation coefficients); or (c) meta-analysts wish to test hypotheses on the equality of the heterogeneity variances.

In addition to these substantive decisions, meta-analysts may also rely on fit information and the results of model comparisons. For instance, Working Models 2 and 3 are both nested in Model 1, so that, in addition to inspecting information criteria, likelihood-ratio testing could provide information about which model may be preferred. In this sense, the selection of a working model is both a substantive and statistical decision.

## 4 Illustrative example

To support meta-analysts with translating their decisions on the dependencies among sampling errors, the modeling of within- and between-study heterogeneity variances, and the choices for the Level 2 and Level 3 correlations among random effects, we illustrate the implementation of Models 1–4 in the R software with an example dataset. Specifically, we showcase the model estimation using the R package “metafor”^28^ and the generation of cluster-robust standard errors using the R package “clubSandwich.”^42^ Please find the full data in Supplementary Material S1 and the detailed R code with the respective output in Supplementary Materials S2 and S3.

In our illustrative example, we chose a subset of an openly available dataset that was published by Schroeders et al.^43^ This meta-analytic dataset contains the correlations among the items of the Toronto Alexithymia Scale (TAS-20) and/or the statistical parameters to derive them (e.g., standardized factor loadings and factor correlations). The goal of the original meta-analysis was to examine the factor structure underlying the scale, generalize reliability evidence, and ultimately craft a validity argument. While this goal may be specific to the study of assessments, our example can also be generalized to other situations and goals. For instance, the selected variables may not necessarily represent item responses, but other quantitative variables such as scores derived from other scales. Note that our illustrative example focuses on the meta-analytic pooling of the correlation matrices via the MLMV-REM approach, yet not the potential follow-up steps using the pooled correlation matrix. These steps may include conducting factor analyses, examining the psychometric network structure underlying the scale, or testing specific hypotheses on the relations among the variables.

### 4.1 Description of the meta-analytic data

For illustrative purposes, we chose the data focusing on participants’ self-reported difficulties with describing feelings. This dimension was measured by the following five items of the TAS-20 (see Schroeders et al.^43^):

- Item 2 (I2): *It is difficult for me to find the right words for my feelings.*
- Item 4 (I4): *I am able to describe my feelings easily. (reverse-coded)*
- Item 11 (I11): *I find it hard to describe how I feel about people.*
- Item 12 (I12): *People tell me to describe my feelings more.*
- Item 17 (I17): *It is difficult for me to reveal my innermost feeling, even to close friends.*

Given the focus on these five items, the correlation matrices we intended to pool meta-analytically were 5 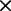 5 matrices with 10 correlation coefficients each. The data were accessed from a csv file, stored in an R object named tas20, and then supplemented by an effect size identifier ESID.

**Figure:**
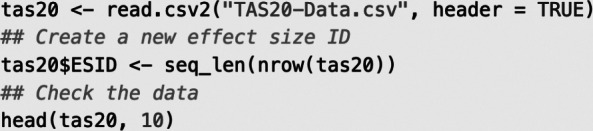

Output:

**Figure:**
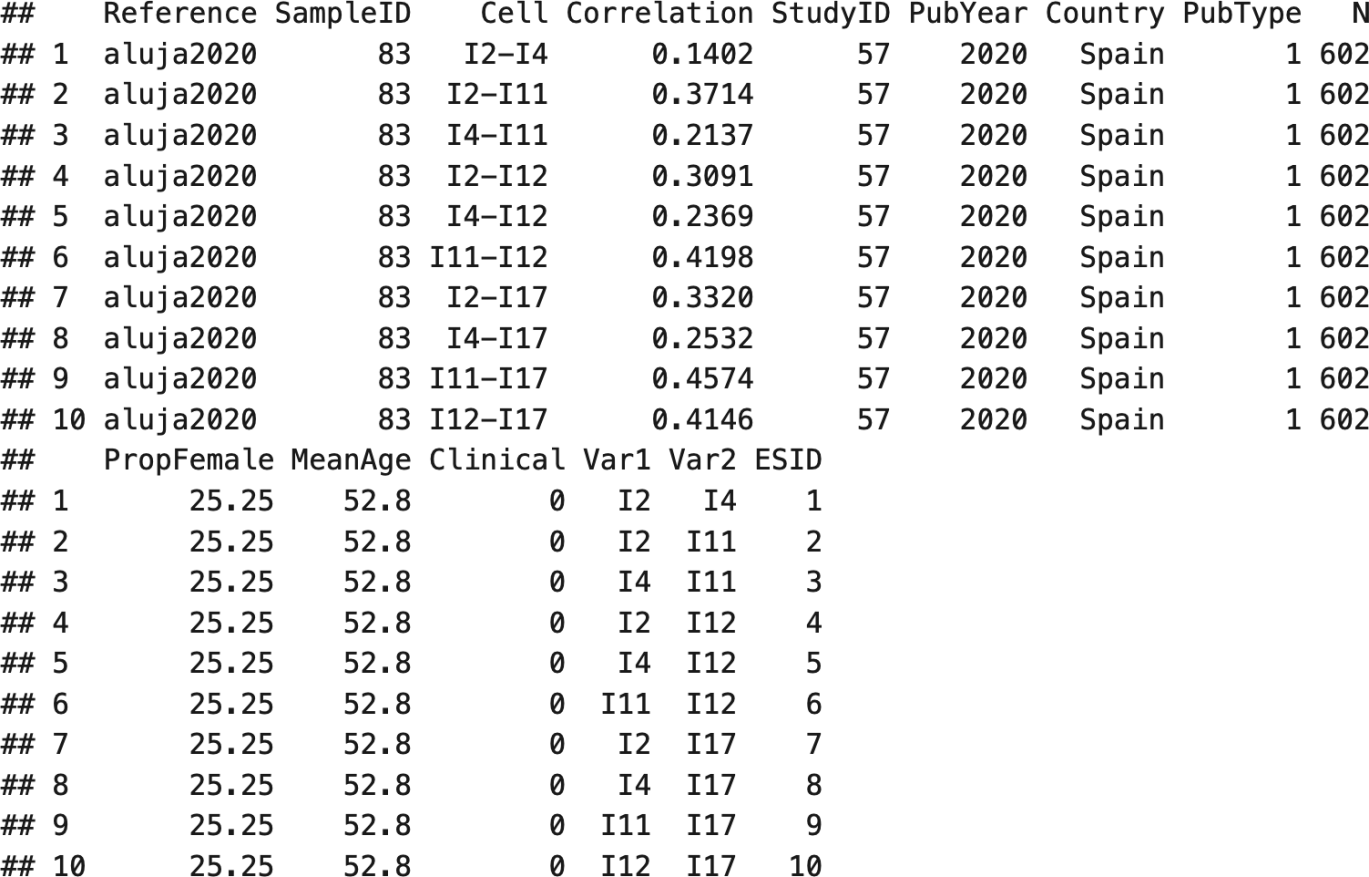

To facilitate the meta-analytic modeling and to indicate the different analytical levels, we created identifiers of the effect sizes (ESID), the samples (SampleID), the primary studies (StudyID), and the type of correlation (Cell; e.g., “I2–I4” indicates the correlation between Items 2 and 4). The correlation coefficients were stored in the variable Correlation, and the names of the corresponding variables are indicated by Var1 and Var2. The dataset contains 880 correlation coefficients that were extracted from 88 samples in 62 primary studies, totaling 69,722 participants. On average, the samples comprised 792 participants (*M* = 792.3, SD = 1,508.8, Mdn = 327, Min = 99, Max = 1,2706), and each study included 1.4 correlation matrices (SD = 0.6, Mdn = 1, Min = 1, Max = 4). Overall, 22 primary studies contained more than two correlation matrices (i.e., samples).

### 4.2 Constructing sampling covariance matrices

If meta-analysts assume independent sampling errors, then they may directly use the sampling variances of the correlation coefficients as input for the meta-analytic working model. If meta-analysts decide to account for the dependencies among sampling errors within correlation matrices for a specific sample, they may construct sampling covariance matrices using the explicit expressions proposed by Olkin and Siotani.^35^ There are several ways to generate the sampling matrices. For instance, the R package “metafor” contains the rcalc() function, which creates a list of sampling covariance matrices (V) for each sample-specific correlation matrix.

**Figure:**
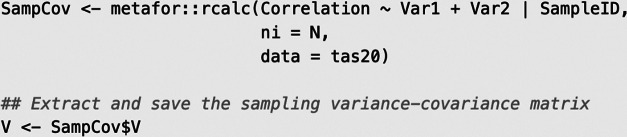

This function takes the variable containing the correlation coefficients (Correlation), the names of the respective variables (Var1, Var2), the clustering variable (SampleID), the sample sizes (ni = N), and the name of the dataset (data = tas20) as arguments. The first part specifies that multiple correlations are nested in sample-specific correlation matrices, because each independent sample provided one correlation matrix, and the last part specifies that sampling variances and covariances are generated using the raw correlation coefficients. The resultant object V contains the full sampling covariance matrix with a block design to indicate the correlation matrices.

## Sampling covariance matrix of the first sample

blsplit(V, tas20$SampleID)$`1`

Output:

**Figure:**
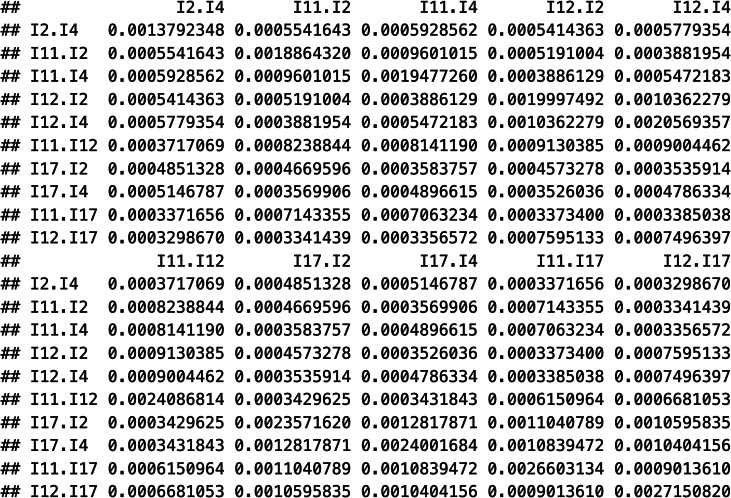

For the R code using Fisher’s- 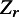 transformation (via the additional option rtoz = TRUE), please see Supplementary Material S3.

Another option to construct sampling covariance matrices is to use the asyCov() function in the R package “metaSEM.”^44^ This function has similar utilities to the above-described function. Nonetheless, it contains some additional features, such as the possibility to also construct sampling correlation matrices (via the cor.analysis option).

V <- asyCov(cmatnew, dat$n, cor.analysis = FALSE)

Several coding steps are leading up to using this function, such as generating a list of correlation matrices from the study samples (cmatnew), along with their sample sizes (n). Please find the details of these steps in Supplementary Material S2. Notice that, from version 1.2.6 of the “metaSEM” package, the asyCov() function no longer implements Cheung and Chan’s^45^ SEM approach that rests on a multivariate normality assumption but the equations by Olkin and Finn.^34^ In the following analyses, we used the V matrix generated from the rcalc() function.

### 4.3 Approaches to pooling correlation matrices

Once the correlation matrices and sampling (co-)variance matrices have been extracted or generated, meta-analysts can employ the working models we proposed to estimate a pooled correlation matrix and heterogeneity (co-)variances. In this section, we describe in detail the implementation of Working Model 1 and explain briefly the implementation of Models 2–4 in R.

#### 4.3.1 Working Model 1

**Figure 2:**
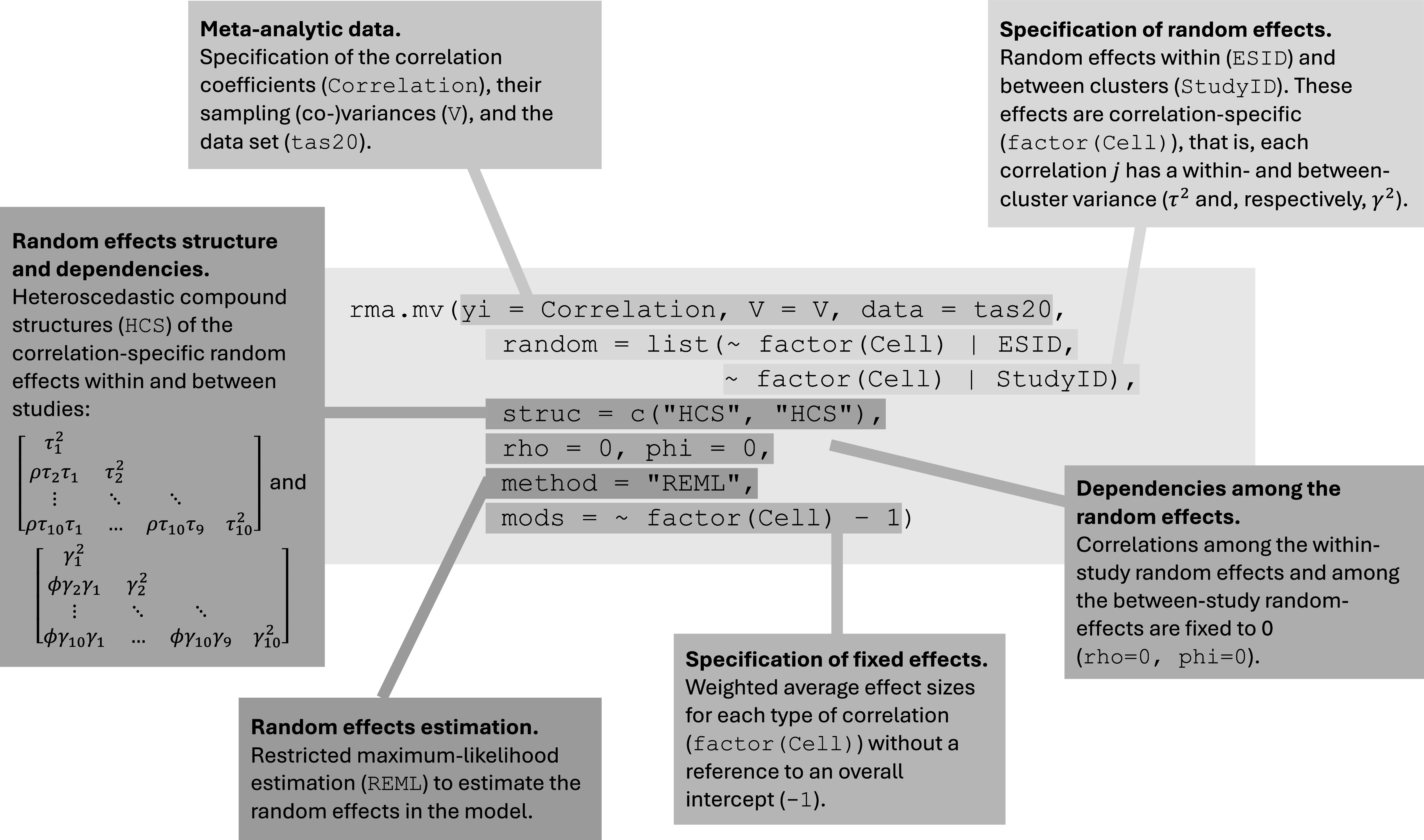
Elements of the analytic code to specify Working Model 1 in the R package “metafor”.

Given the multivariate nature of the data (i.e., multiple correlations per sample that are based on multiple, measured variables in the primary studies) and the hierarchical effect size multiplicity (i.e., multiple correlation matrices nested in primary studies), we used the rma.mv() function in “metafor” to estimate Model 1. Figure 2 shows an example code with explanations of its elements. Specifically, this function contains the specification of the correlation coefficients (yi), the sampling covariance matrices (V), and the dataset in which these elements are stored (data). Next, the random effects within (|ESID) and between studies (|StudyID) are specified as a list. Given that Model 1 assumes correlation-specific estimates of these random effects, the option ~factor(Cell) is added to this list. Note that the sampling covariances were generated in the previous step using SampleID as a clustering identifier to account for the nesting of multiple correlation coefficients in the samples’ correlation matrices. In the pooling step, the sample specificity of the correlation coefficients is represented by ESID (i.e., each sample contributes only one correlation coefficient within a correlation matrix), and the hierarchical nesting of multiple correlation matrices in primary studies is represented by StudyID.

Suppose that meta-analysts do have some evidence that the Level 2 and Level 3 random effects can be considered independent. Specifying an HCS structure within and between studies (struc = c(“HCS,”"HCS”)), they may assume a correlation of zero within and zero between studies (rho = 0 and, respectively, phi = 0). Next, the estimation procedure is set; in our example, we chose restricted maximum likelihood estimation (method = “REML”). Finally, we specified the variable indicating the type of correlation as a moderator and turned off the intercept of this meta-regression part (mods = ~factor(Cell)-1). This way, we get estimates of each pooled correlation coefficient directly. Labelling the rma.mv() object “tas20.mlmvrem1” and using the summary() function, we obtained the following output of this model estimation:

**Figure:**
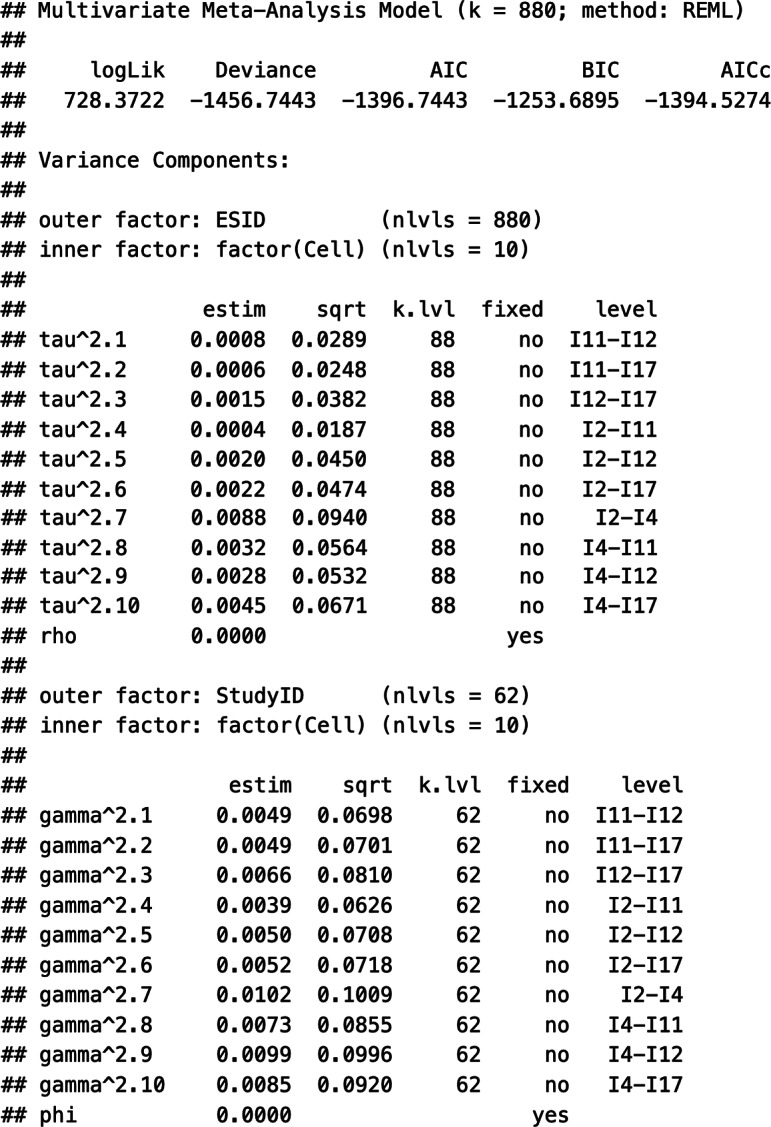

**Figure:**
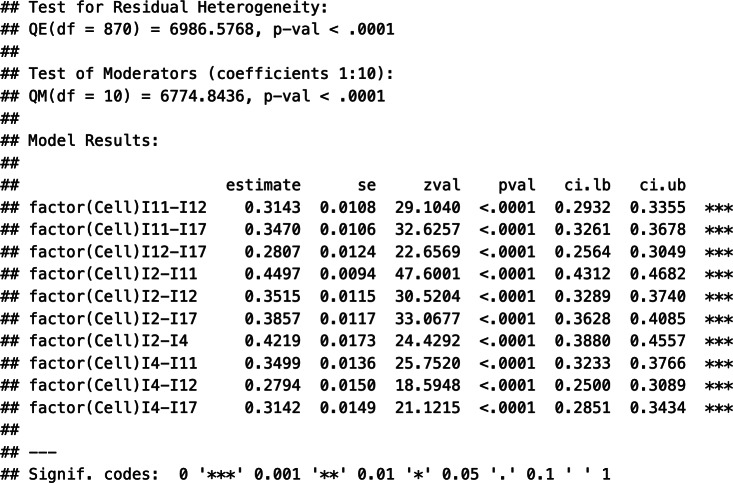

First, the output contains information about the log-likelihood, deviance, and information criteria, which can be used for further model comparisons. Second, the output displays the within-study variance estimates (tau^2.1-tau^2.10) and the between-study variance estimates (gamma^2.1-gamma^2.10) for each of the 10 correlation coefficients. These estimates exhibited a considerable range, with some variances being close to zero and others as high as 0.0088 (tau^2.7). Third, the *Q* statistics are shown, indicating, for instance, that statistically significant (residual) heterogeneity existed in the data ( 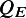 [870] = 6,986.58, *p* < .01). Fourth, the estimates of the weighted average correlation coefficients, their standard errors, *z*-statistics, *p*-values, and the respective confidence intervals are shown (section Model results). Notably, all pooled coefficients were positive and were statistically significantly different from zero.

**Table 2:** Weighted average effect sizes and variance components in Working Models 1–4 with independent random effects (with 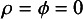 )

Item-item correlation	Weighted average effect sizes			Heterogeneity		Information criteria		
				Within-study	Between-study			
	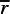	*SE*	95% CI	heterogeneity 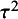	heterogeneity 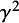	AIC	BIC	AICc
**Model 1** (Correlation-specific effect sizes, within- and between-study heterogeneity)	−1,396.7	−1,253.7	−1,394.5
I11–I12	0.314	0.013	[0.288, 0.341]	0.001	0.005			
I11–I17	0.347	0.013	[0.321, 0.373]	0.001	0.005			
I12–I17	0.281	0.015	[0.250, 0.311]	0.001	0.007			
I2–I11	0.450	0.011	[0.427, 0.473]	0.000	0.004			
I2–I12	0.351	0.014	[0.323, 0.380]	0.002	0.005			
I2–I17	0.386	0.014	[0.357, 0.414]	0.002	0.005			
I2–I4	0.422	0.019	[0.383, 0.461]	0.009	0.010			
I4–I11	0.350	0.016	[0.318, 0.382]	0.003	0.007			
I4–I12	0.279	0.018	[0.244, 0.315]	0.003	0.010			
I4–I17	0.314	0.017	[0.279, 0.349]	0.005	0.008			
**Model 2** (Correlation-specific effect sizes and within-study heterogeneity, constrained between-study heterogeneity)	−1,407.1	−1,307.0	−1,406.1
I11–I12	0.314	0.013	[0.287, 0.341]	0.001	0.005			
I11–I17	0.347	0.013	[0.321, 0.373]	0.001	0.005			
I12–I17	0.281	0.015	[0.250, 0.311]	0.002	0.005			
I2–I11	0.449	0.011	[0.426, 0.472]	0.000	0.005			
I2–I12	0.351	0.014	[0.323, 0.380]	0.002	0.005			
I2–I17	0.385	0.014	[0.357, 0.414]	0.002	0.005			
I2–I4	0.422	0.019	[0.385, 0.460]	0.012	0.005			
I4–I11	0.350	0.016	[0.319, 0.382]	0.004	0.005			
I4–I12	0.280	0.017	[0.246, 0.315]	0.005	0.005			
I4–I17	0.315	0.017	[0.280, 0.350]	0.006	0.005			
**Model** 3 (Correlation-specific effect sizes and between-study heterogeneity, constrained within-study heterogeneity)	−1,379.9	−1,279.7	−1,378.8
I11–I12	0.314	0.013	[0.287, 0.341]	0.002	0.004			
I11–I17	0.347	0.013	[0.321, 0.373]	0.002	0.003			
I12–I17	0.281	0.015	[0.251, 0.311]	0.002	0.006			
I2–I11	0.448	0.012	[0.425, 0.472]	0.002	0.003			
I2–I12	0.351	0.014	[0.323, 0.380]	0.002	0.005			
I2–I17	0.386	0.014	[0.357, 0.414]	0.002	0.005			
I2–I4	0.423	0.020	[0.383, 0.463]	0.002	0.016			
I4–I11	0.350	0.016	[0.318, 0.382]	0.002	0.008			
I4–I12	0.280	0.018	[0.244, 0.315]	0.002	0.010			
I4–I17	0.314	0.018	[0.279, 0.350]	0.002	0.010			
**Model 4** (Correlation-specific effect sizes, constrained within- and between-study heterogeneity)	−1,360.5	−1,303.2	−1,360.1
I11–I12	0.313	0.013	[0.287, 0.340]	0.003	0.007			
I11–I17	0.346	0.013	[0.320, 0.372]	0.003	0.007			
I12–I17	0.280	0.015	[0.250, 0.311]	0.003	0.007			
I2–I11	0.447	0.012	[0.424, 0.470]	0.003	0.007			
I2–I12	0.350	0.014	[0.322, 0.379]	0.003	0.007			
I2–I17	0.385	0.014	[0.356, 0.413]	0.003	0.007			
I2–I4	0.426	0.019	[0.387, 0.465]	0.003	0.007			
I4–I11	0.352	0.016	[0.320, 0.383]	0.003	0.007			
I4–I12	0.281	0.018	[0.246, 0.316]	0.003	0.007			
I4–I17	0.316	0.017	[0.281, 0.351]	0.003	0.007			

*Note*: 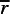 = Weighted average Pearson correlation coefficient with cluster-robust standard error and 95% confidence interval. “I” refers to the item in the TAS-20. Correlations are labeled using the item numbers (e.g., “I11–I12” refers to the correlation between Items 11 and 12). AIC = Akaike’s Information Criterion; BIC = Bayesian Information Criterion; AICc = Corrected AIC.^28^ All models assume independent random effects (i.e., 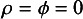 ) and block-diagonal sampling covariance matrices.

If meta-analysts wish to further obtain cluster-robust standard errors of the pooled correlation coefficient estimates (with primary studies as clusters, indicated by StudyID), they may use the convenience function robust() in the R package “metafor” that calls the “clubSandwich” package:

**Figure:**
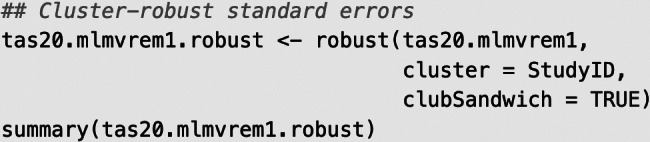

Selected output:

**Figure:**
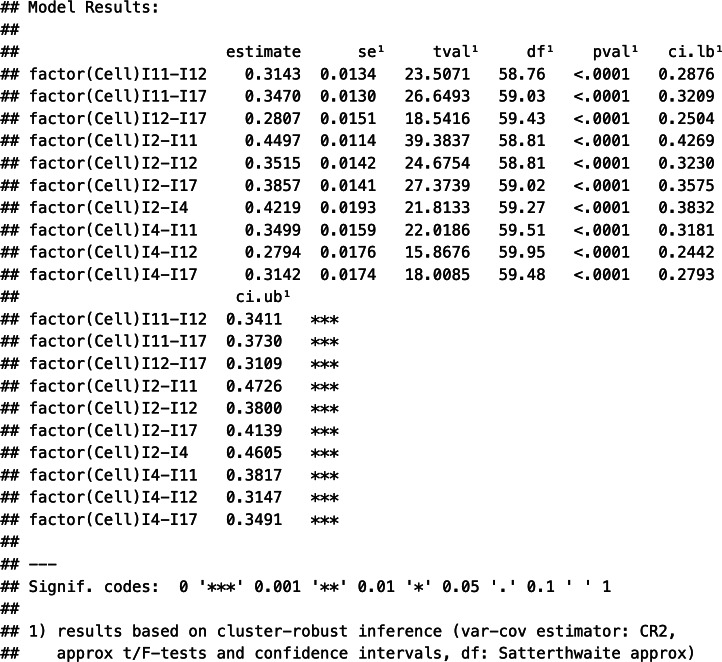

Table 2 summarizes the key estimates of Model 1. However, the previous output does not contain any information about the preference of Model 1 over alternative models. Hence, in the next section, we describe the estimation of Models 2–4 and perform model comparisons to select an appropriate working model for the meta-analytic pooling of the correlation matrices.

#### 4.3.2 Working Models 2–4

The specification of Working Models 2–4 is like that of Model 1. While Model 1 allowed for correlation-specific estimates of the heterogeneity variances, Models 2–4 restricted these estimates to be the same across correlations. This is reflected in the specification of the structures of Level 2 and Level 3 random effects. Table 1 shows sample codes to estimate Models 2–4 in “metafor.” For instance, in Model 2, the random-effects structures are specified as struc = c(“HCS,”"CS”), and the respective output contains the following variance estimates:

**Figure:**
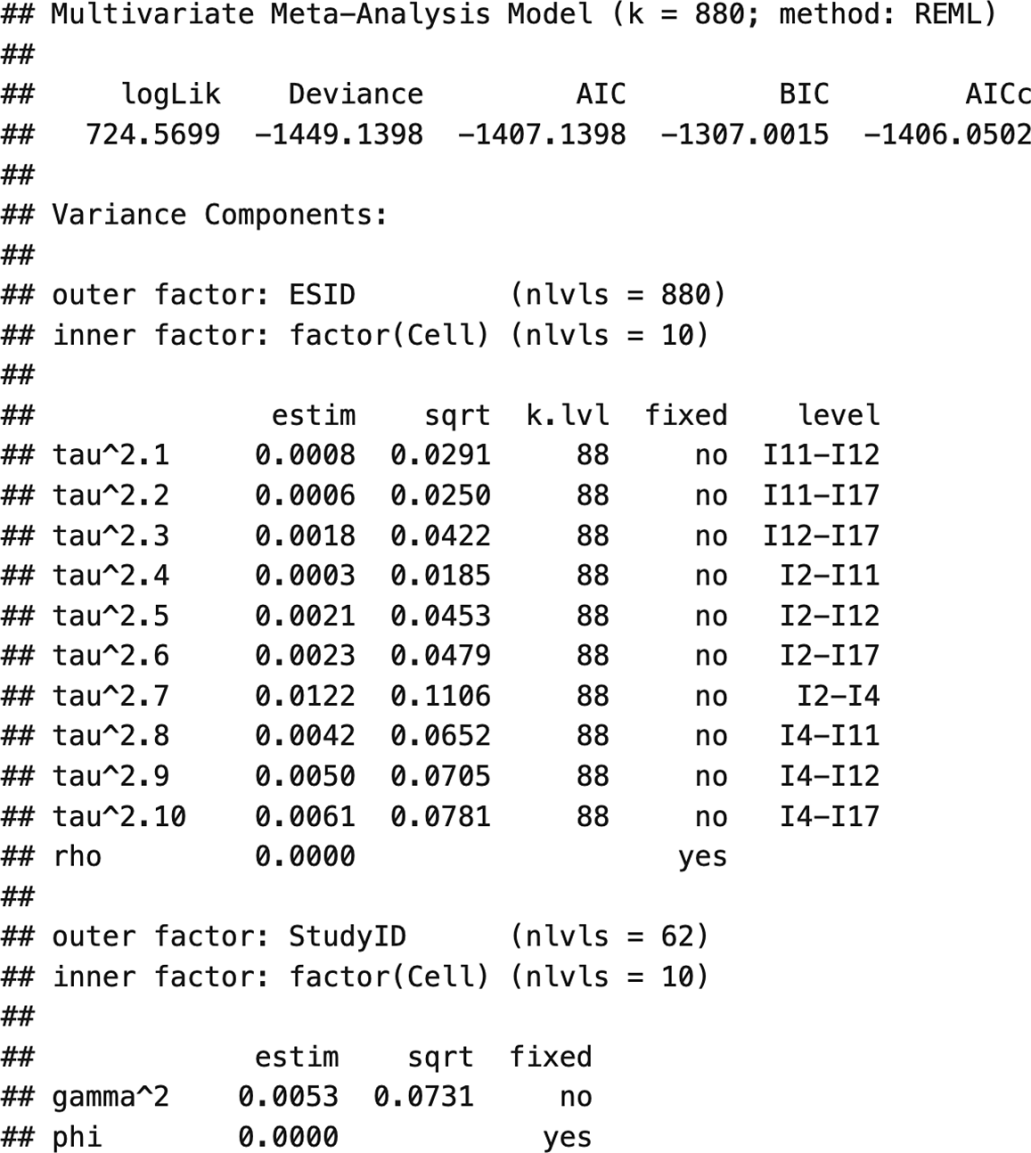

Notably, the within-study variance estimates are correlation-specific (tau^2.1-tau^2.10), and only one heterogeneity variance is estimated at the between level (gamma^2). Similarly, Model 3 can be specified using the option struc = c(“CS,”"HCS”) to implement the assumption of correlation-specific heterogeneity variances between studies but one uniform variance estimate within studies. Finally, the structure option in Model 4 specifies two compound structures struc = c(“CS,”"CS”). Please find the output of the estimation of Models 3 and 4 in Supplementary Material S2. Table 2 shows the parameters of Models 2–4.

#### 4.3.3 Model selection

To select a working model, we compared the information criteria and performed likelihood-ratio tests across Models 1–4. Table 2 contains the respective values of the AIC, BIC, and a corrected AIC. Consistently across these information criteria, Model 2 showed the lowest values and thus the best fit. Moreover, a likelihood-ratio test between Models 1 and 2 suggested that these models did not differ statistically significantly in their fit, 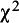 (9) = 7.60, *p* = .57. We performed this test using the anova() function:

**Figure:**
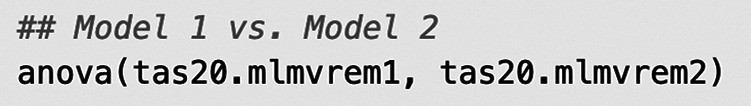

Output:

**Figure:**
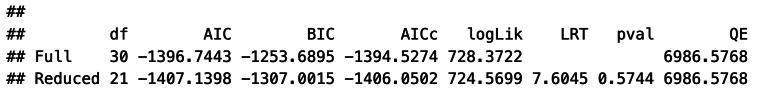

Further likelihood-ratio tests suggested the preference of Model 2 over Models 3 and 4. This preference of Model 2 indicated that the between-level variance estimates did not differ significantly and can thus be considered statistically equal across correlation coefficients. Hence, a meta-analyst may select Model 2 as her working model to pool the correlation matrices meta-analytically and conduct subsequent moderator analyses. Notice that our specifications of Models 1–4 assumed that 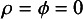 .

### 4.4 Moderator analyses

As mentioned earlier, Models 1–4 can be extended to mixed-effects meta-regression models to explore moderator effects and examine the extent to which heterogeneity can be explained by the characteristics of the primary studies, samples, or measures. In this section, we illustrate this possible extension by a categorical (i.e., type of the sample, coded as *0 = non-clinical* and *1 = clinical*; ClinicalBinary) and a continuous moderator (i.e., the proportion of female participants in the primary study; PropFemale).

#### 4.4.1 Categorical moderator

There are several ways of exploring the possible moderating effect of a categorical variable using the MLMVREM approach. One way is to extend the model specification of Model 2 in “metafor” by specifying a crossed effect between the moderator and the variable that indicates the type of correlation (i.e., factor(Cell)). This specification still contains the direct effect of factor(Cell), which serves as the intercepts, that is, the expected weighted average correlations for each type of correlation when the sample is nonclinical. Hence, the model specification no longer contains an overall intercept (−1).

**Figure:**
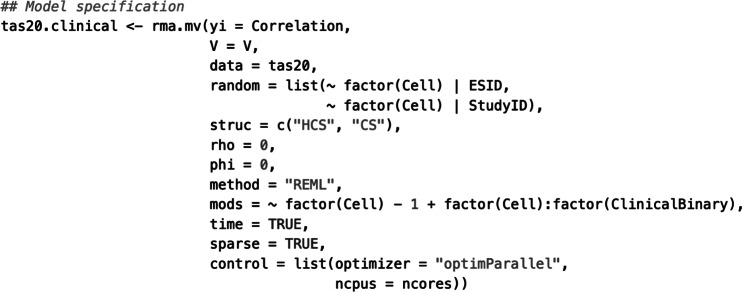

Selected output of the model summary:

**Figure:**
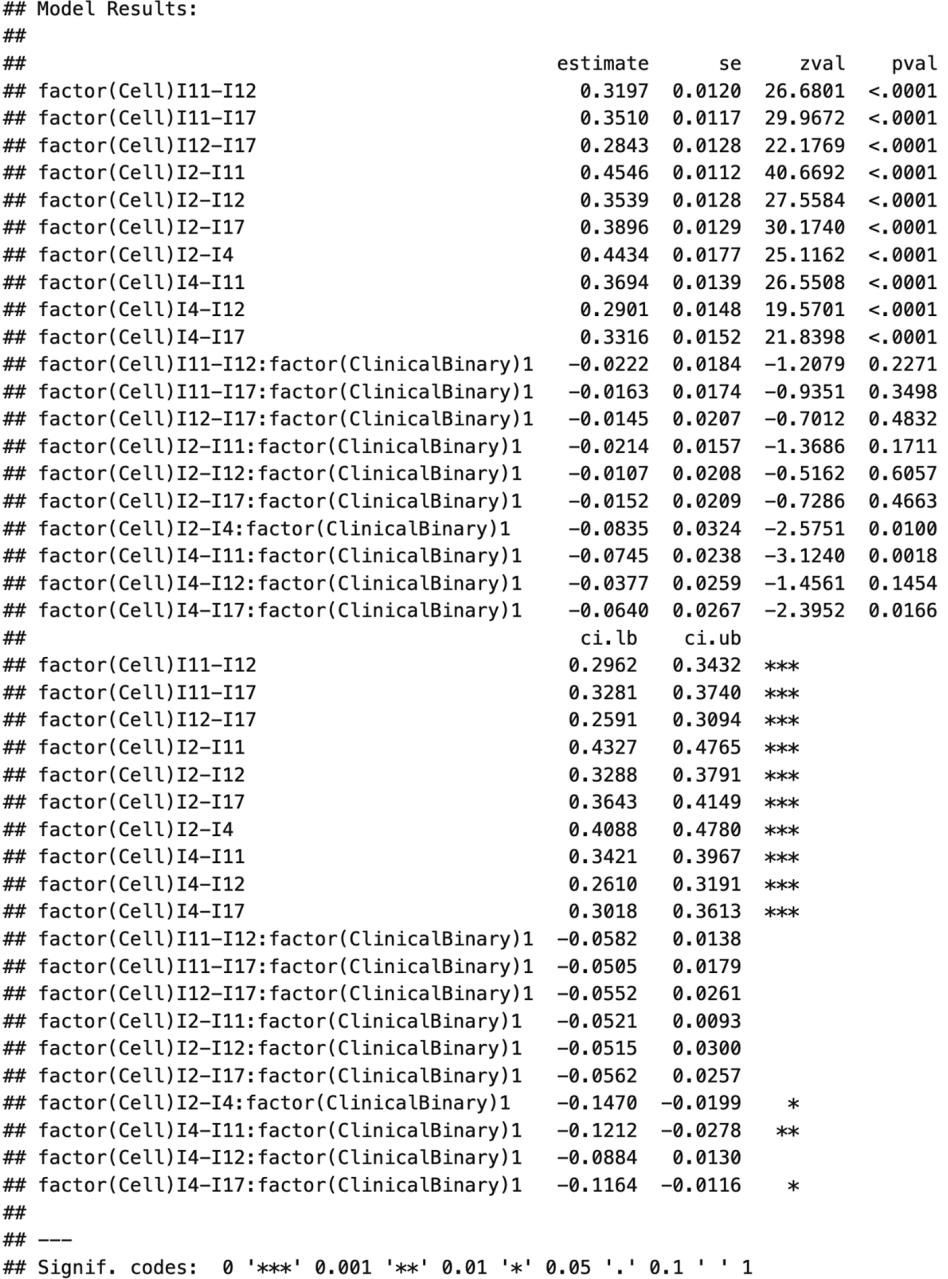

The estimates of the model parameters indicate statistically significant and negative moderator effects of the type of the sample on three correlation coefficients: I2–I4 (*B* = −0.084, SE = 0.032, *p* = .010), I4–I11 (*B* = −0.075, SE = 0.024, *p* = .002), and I4–I17 (*B* = −0.064, SE = 0.027, *p* = .017). Hence, these correlations tended to be smaller for clinical samples than for nonclinical samples. The cluster-robust standard errors supported this result. Next, we computed the variance explanations for each level of analysis and some selected correlations.

**Figure:**
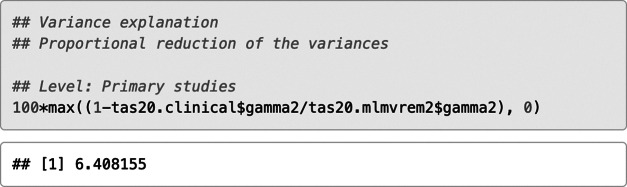

**Figure:**
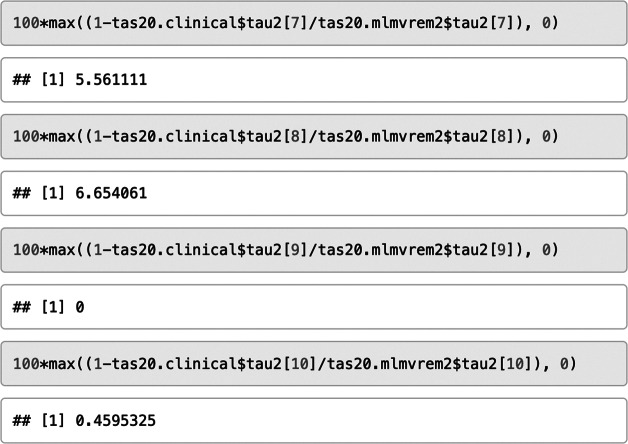

About 6.4% of the between-study variation in effect sizes was explained by the type of sample, and about 5.6% (6.7%, 0.5%) of the within-study variation in the correlations I2–I4 (I4–11, I4–I17) was explained.

Another way of exploring the effect sizes across the moderator’s categories is to estimate the subgroup-specific effect sizes directly. To achieve this, the moderator option in the above-described specification is changed into

mods =~ factor(Cell):factor(ClinicalBinary)-1.

Consequently, meta-analysts obtain the effect size estimates of each correlation for studies with nonclinical (code 0) and clinical samples (code 1). However, this way, the similarities and differences in effects are not tested statistically.

Selected output:

**Figure:**
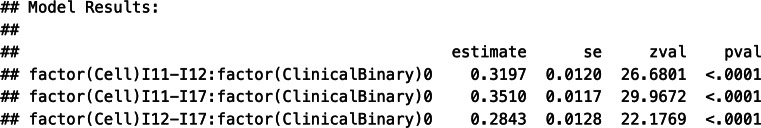

**Figure:**
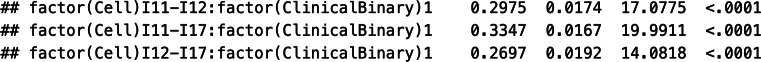

**Figure:**
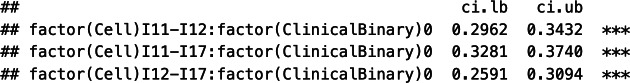

**Figure:**
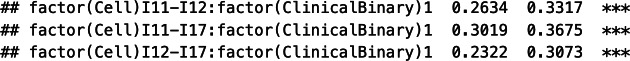

#### 4.4.2 Continuous moderator

Introducing continuous moderators works in the same way as adding categorical moderators. However, given that continuous moderators do not have discrete categories, subgroup-specific estimates of the effect sizes are not accessible. In this example, we chose the proportion of female study participants as a possible moderator without any variance-stabilizing transformation for better interpretability and to avoid issues related to back-transformations.^46^ ^,^ ^47^ The model specification code is similar to that of the categorical example and contains a modified mods option (model tas20.gender in Supplementary Material S2):

mods =~ factor(Cell) − 1 + factor(Cell):PropFemale.

The results indicated that the correlation I11–I17 was moderated by the proportion of female study participants, *B* = −0.001, SE = 0.001, *p* = .008. This effect was supported by the cluster-robust standard errors. Please find the detailed output in Supplementary Material S2.

### 4.5 Sensitivity analyses

As noted earlier, some decisions on model parameters in the MLMV-REM may be informed by prior research or meta-analysts’ substantive expertise. To examine the sensitivity of the meta-analytic findings, we first explored how different a-priori fixed values of 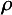 (0.0, 0.5, 1.0) and 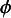 (0.0, 0.5, 1.0) affected (a) model selection and (b) key parameter estimates of Model 1, such as the weighted average correlation coefficients or heterogeneity variances. Second, we conducted the analyses using Fisher’s- 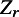 transformed scores.

For the raw correlations, Models 1 and 2 were suitable meta-analytic working models to pool the correlation matrices across the conditions. When 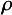 and 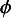 were small, Model 2 was likely to be preferred over Model 1. The relation between 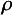 (or, respectively, 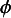 ) and the within-sample variance estimates was mostly V-shaped, and the largest values of the respective variance estimates occurred for 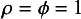 . The between-sample variance estimates showed the same pattern. Finally, pooled correlation coefficients increased with higher values of 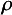 and 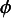 . For Fisher’s- 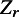 transformed scores and given values of 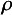 and 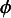 , pooled correlation coefficients, within- and between-sample variance estimates were slightly larger than those obtained from the raw correlations.

The detailed results of these sensitivity analyses are shown in Supplementary Materials S2 and S3. Overall, the pooled correlations and heterogeneity estimates were sensitive to the choices of 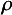 and 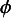 , and simulation studies are needed to examine this sensitivity systematically.

## 5 Discussion and recommendations

In this tutorial paper, we have described an MLMV-REM approach to addressing hierarchical effect size multiplicity when pooling correlation matrices across primary studies. This approach can be specified via several working models under various assumptions on the correlation-specific within- and between-study variances. We illustrated the implementation of four working models using the R package “metafor” and the respective cluster-robust standard errors using the R package “clubSandwich.” This illustration contained a description of the working models’ assumptions, the R code to specify and estimate them, and the resultant R output.

As noted earlier, the MLMV-REM approach allows meta-analysts to model within- and between-study heterogeneity, either specific for each correlation coefficient or under the assumption of equal heterogeneity across correlations at the respective levels of analysis. To the best of our knowledge, this approach is the first to make explicit these heterogeneity variances—existing approaches either provide one heterogeneity variance estimate for all correlation coefficients at the within and between level^12^ or correlation-specific heterogeneity variance estimates at a single level of analysis.^3^ We therefore consider the MLMV-REM approach an extension and integration of the existing approaches. This approach not only provides meta-analysts with information about associations among multiple constructs, concepts, or variables and their heterogeneity but also has the potential to advance meta-analytic SEM, in which meta-analysts can test their theories and hypotheses about these associations.^10^

The working models within the MLMV-REM approach represented several assumptions meta-analysts may have on the degree of sampling error dependence and the structure of the within- and between-level random effects. This not only allows meta-analysts to test hypotheses on these structures (e.g., equal vs. correlation-specific heterogeneity variances) but also enables them to select a model for the pooling of correlation matrices that represents the structure and nature of their meta-analytic data. We recommend selecting a meta-analytic working model by estimating and comparing models with several assumptions, evaluating model fit, and considering substantive aspects, such as theories or existing evidence from other studies. Moreover, we encourage meta-analysts to also consider the feasibility of working models with many correlation-specific parameter estimates. Sometimes, too few studies and correlation matrices are available to reliably estimate such models with sufficient statistical power.^3^ It remains for future research to examine systematically the sample size requirements of the working models and identify situations in which the respective fixed and random effects can be estimated reliably.

The MLMV-REM approach has several limitations and leaves some open questions that point to future research directions. First, while this approach offers a flexible specification of model assumptions on the structure of random effects, model selection becomes not only an opportunity but a necessity. Oftentimes, meta-analytic data contain too few studies to estimate all possible variance components of correlations within a correlation matrix.^3^ In this situation, meta-analysts would need to strive for model parsimony and select a model that may not capture all correlation-specific heterogeneity variances within and between studies, but some. Modern methods such as Bayesian meta-analysis may address the challenges of few available studies.

Nonetheless, we currently see the lack of guidance concerning the required number of studies and effect sizes when pooling correlation matrices meta-analytically. Some prior research has pointed to the need for sufficiently large numbers of studies when specifying complex random effects. For instance, Vembye et al.^48^ showcased the associations between the required number of studies and effect sizes, how many model parameters are estimated, how large their estimates are, the magnitude of the within- and between-study heterogeneity, and the strengths of the correlational dependencies. These authors have developed approximations and tools to estimate statistical power and explore sample size requirements for random-effects models with correlational and hierarchical effect size multiplicity.^49^ Cheung^3^ pointed out too few studies may not result in trustworthy, pooled correlation matrices, especially if many variables and thus correlations are involved. If, for instance, the number of estimated parameters in the pooling stage exceeds the number of studies, non-convergence or non-admissible solutions may occur. In our illustrative example, we focused on five variables and thus 10 correlation coefficients per correlation matrix. In Model 1, 10 pooled correlations are estimated, 10 within-study variances, and another 10 between-study variances, totaling 30 model parameters. Although the data obtained from the 88 samples may provide enough statistical power to estimate the pooled correlations, they may not provide enough statistical power to detect potentially small heterogeneity variances for all correlation coefficients. Despite these speculations, we call for simulation studies that shed more light on the meta-analytic sample size requirements and the factors associated with them.

Second, the current implementation of the MLMV-REM approach synthesizes correlation matrices meta-analytically and estimates the respective heterogeneity (co-)variances. Hence, meta-analysts who are interested in testing hypotheses and theories about the relations among variables would then submit these elements to SEM or psychometric network modeling.^30^ ^,^ ^50^ This two-stage approach addresses hierarchical effect size multiplicity at Stage 1. We encourage meta-analysts to develop and explore alternative approaches that may integrate these two analytic stages into one stage. Such an integration could improve estimation efficiency and reduce possible bias in the variance estimates.^51^

Third, the MLMV-REM approach requires substantive decisions on the model specification, such as the structure of random effects or the sampling covariance matrix 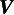 and the choices of the correlations between sampling errors in the matrix 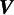 and the correlations between random effects in the matrices 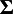 and 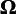 . These decisions also include the choices for an approach to construct sampling covariance matrices and the within- and between-level random effects. Oftentimes, researchers do not have specific hypotheses or prior evidence on these correlations, which leaves them to guess these values. In future model specifications, however, it may be possible to estimate such correlations.^5^ ^,^ ^28^ ^,^ ^32^ Overall, we recommend backing the analytical decisions meta-analysts have to make by conducting sensitivity analyses to examine the impact of these decisions on meta-analytic estimates.

Fourth, the MLMV-REM approach employs maximum-likelihood estimation to pool correlation matrices with partially missing data. However, it typically assumes that any unreported correlations are missing at random or missing completely at random.^52^ If, in reality, certain correlations are systematically omitted (i.e., missing not at random [MNAR]), the resulting pooled estimates can become biased. Moreover, when information on the within-sample correlation structure is limited or absent, the “borrowing strength” across studies is less effective, leading to larger standard errors for pooled estimates.^21^ Although alternative methods for handling missing correlations through multiple imputation have been proposed,^40^ ^,^ ^52^ further research is needed to assess their performance when estimating MLMV-REM models in the presence of the MNAR mechanism.

Fifth, effect size multiplicity may be hierarchical, correlational, or both.^5^ In our view, the MLMV-REM we have proposed is best suited for meta-analytic datasets with hierarchical multiplicity, in which multiple independent correlation matrices are nested in primary studies. In such a scenario, the variance components have a clear interpretation as within- and between-study heterogeneity variances of the correlation coefficients. In scenarios in which multiple correlation matrices are available for the same sample within a study, our proposed approach may not be best-suited and may only approximate the true weighted average correlation coefficients and their heterogeneity. Specifically, the MLMV-REM approach we proposed assumes zero correlation between correlation matrices within studies. Current methodological developments in the meta-analytic pooling of univariate effect sizes, such as the working models proposed by Pustejovsky and Tipton,^5^ and meta-analytic models describing longitudinal data^28^ could serve as starting points to develop an approach that handles meta-analytic data with correlational multiplicity.

In our view, the MLMV-REM approach represents *one* possible approach to address hierarchical effect size multiplicity when pooling correlation matrices meta-analytically, and alternative approaches may be developed in the future. This approach will have to stand trial against other approaches, and, in our view, investigating its accuracy, efficiency, and statistical power is a natural next step toward evaluating its performance.

## Supporting information

Scherer and Campos supplementary material Scherer and Campos supplementary material

## Data Availability

This study was pre-registered in the Open Science Framework (OSF) at https://doi.org/10.17605/OSF.IO/ZNWR6. The analytical R code, data, and supplementary materials are fully disclosed and contained in the respective OSF project at https://doi.org/10.17605/OSF.IO/XU8ES. The data underlying the illustrative example were extracted from Schroeders, Kubera, and Gnambs,^43^ who have made them openly available at https://osf.io/uxtks/.

## Notes

i.
Although we focus on synthesizing correlation coefficients in this study, the formulation of the MLMV-REM can be extended to other kinds of effect sizes.

ii.
In this list of approaches, we focus on those that approximate or construct sampling covariance matrices for correlation matrices. If the MLMV-REM is specified for other kinds of effect sizes, other approaches may be accessible, such as fixing a constant sampling correlation and then approximating the sampling covariance matrices.^5^

## Supplementary material

To view supplementary material for this article, please visit http://doi.org/10.1017/rsm.2025.10027.
