# Supplementary material for: Meta-analyzing correlation matrices in the presence of hierarchical effect size multiplicity
Source: Res Synth Methods. 2025 Aug 7;16(6):828–58. doi: 10.1017/rsm.2025.10027 (PMC12657669; doi:10.1017/rsm.2025.10027)
Supplement: Scherer and Campos supplementary material [file S1759287925100276sup001.zip › S1759287925100276sup001/S2-Supplementary-Material.html]

Supplementary Material


# Supplementary Material

#### Ilustrative Example: Factor Structure of the TAS-20 Subscale Measuring Difficulties Describing Feelings (DDF)

#### 27 March 2025

# Purpose, Content, and Context of this Illustrative Example

With this data example, we illustrate how the multilevel,
multivariate, and random-effects approach can be implemented in the R
package `metafor`. Specifically, we provide the analytic code
for specifying and estimating a series of models that accommodate both
the dependencies among multiple correlation coefficients and the
hierarchical structure of the data (e.g., with multiple correlation
coefficients nested in primary studies or study samples).

In this example, we specify block-diagonal sampling covariance
matrices of the correlation matrices of our interest. We essentially
assume that each correlation within a correlation matrix has its own
sampling variance, and effect sizes within a correlation matrix are
dependent. This is a **sampling covariance dependence
structure**.

In this structure, we construct the sampling variance-covariance
matrices, following the equations by Steiger (1980) and
Olkin and Finn
(1990), which have been implemented by Wolfgang Viechtbauer in the R
package `metafor`.

# Meta-Analytic Data

## Source and Acknowledgement

We chose a subset of the data published by Schroeders et
al. (2022), which contains item-item correlations and/or the
elements used to retrieve them from the Toronto Alexithymia Scale
(TAS-20). At this point, we wish to acknowledge the originality of
Schroeders et al.’s works and do not claim any credit for the work
behind generating this data set.

For illustrative purposes, we chose the data focusing on the
dimension DDF, that is, participants’ self-reported difficulties with
describing feelings. This dimension is measured by the following
indicators (see Schroeders et al. (2022)):

- Item 2 (I2): It is difficult for me to find the right words for my
  feelings.
- Item 4 (I4): I am able to describe my feelings easily.
  (reverse-coded)
- Item 11 (I11): I find it hard to describe how I feel about
  people.
- Item 12 (I12): People tell me to describe my feelings more.
- Item 17 (I17): It is difficult for me to reveal my innermost
  feeling, even to close friends.

## Install and load relevant `R` packages

```
# Install R packages (if needed)
# install.packages("pacman")
library(pacman)
pacman::p_load(psych,
               metaSEM,
               metafor,
               robumeta,
               clubSandwich,
               corrplot,
               dplyr,
               psych,
               optimParallel,
               semPlot,
               lattice)

## Number of available cores
ncores <- parallel::detectCores() - 1
```

## Data Input and Preparation

```
## Data set in wide format
tas20 <- read.csv2("TAS20-Data.csv", header = TRUE)
## Create a new effect size ID
tas20$ESID <- seq_len(nrow(tas20))
## Check the data
head(tas20, 10)
```

```
##    Reference SampleID    Cell Correlation StudyID PubYear Country PubType   N
## 1  aluja2020       83   I2-I4      0.1402      57    2020   Spain       1 602
## 2  aluja2020       83  I2-I11      0.3714      57    2020   Spain       1 602
## 3  aluja2020       83  I4-I11      0.2137      57    2020   Spain       1 602
## 4  aluja2020       83  I2-I12      0.3091      57    2020   Spain       1 602
## 5  aluja2020       83  I4-I12      0.2369      57    2020   Spain       1 602
## 6  aluja2020       83 I11-I12      0.4198      57    2020   Spain       1 602
## 7  aluja2020       83  I2-I17      0.3320      57    2020   Spain       1 602
## 8  aluja2020       83  I4-I17      0.2532      57    2020   Spain       1 602
## 9  aluja2020       83 I11-I17      0.4574      57    2020   Spain       1 602
## 10 aluja2020       83 I12-I17      0.4146      57    2020   Spain       1 602
##    PropFemale MeanAge Clinical Var1 Var2 ESID
## 1       25.25    52.8        0   I2   I4    1
## 2       25.25    52.8        0   I2  I11    2
## 3       25.25    52.8        0   I4  I11    3
## 4       25.25    52.8        0   I2  I12    4
## 5       25.25    52.8        0   I4  I12    5
## 6       25.25    52.8        0  I11  I12    6
## 7       25.25    52.8        0   I2  I17    7
## 8       25.25    52.8        0   I4  I17    8
## 9       25.25    52.8        0  I11  I17    9
## 10      25.25    52.8        0  I12  I17   10
```

# Description of the Meta-Analytic Dataset

In the following section, we describe the meta-analytic sample by
providing the number of effect sizes and articles, along with some
characteristics of the samples.

```
## Number of effect sizes
nrow(tas20)
```

```
## [1] 880
```

```
## Number of articles
length(table(tas20$StudyID))
```

```
## [1] 62
```

```
table(tas20$StudyID)
```

```
## 
##  1  2  3  4  5  6  7  8  9 10 11 12 13 14 15 16 17 18 19 20 21 22 23 24 25 26 
## 30 30 10 20 10 10 10 10 10 10 10 20 10 10 10 10 20 10 20 20 10 10 10 20 20 20 
## 27 28 29 30 31 32 33 34 35 36 37 38 39 40 41 42 43 44 45 46 47 48 49 50 51 52 
## 10 10 20 10 10 10 10 10 20 20 20 10 20 10 10 20 40 10 10 10 20 10 10 10 20 20 
## 53 54 55 56 57 58 59 60 61 62 
## 10 10 20 20 10 10 10 10 10 10
```

```
## Average number of correlation matrices per study
mean(table(tas20$StudyID)/10)
```

```
## [1] 1.419355
```

```
sd(table(tas20$StudyID)/10)
```

```
## [1] 0.6414223
```

```
median(table(tas20$StudyID)/10)
```

```
## [1] 1
```

```
min(table(tas20$StudyID)/10)
```

```
## [1] 1
```

```
max(table(tas20$StudyID)/10)
```

```
## [1] 4
```

```
## Number of samples
length(table(tas20$SampleID))
```

```
## [1] 88
```

```
table(tas20$SampleID)
```

```
## 
##  1  2  3  4  5  6  7  8  9 10 11 12 13 14 15 16 17 18 19 20 21 22 23 24 25 26 
## 10 10 10 10 10 10 10 10 10 10 10 10 10 10 10 10 10 10 10 10 10 10 10 10 10 10 
## 27 28 29 30 31 32 33 34 35 36 37 38 39 40 41 42 43 44 45 46 47 48 49 50 51 52 
## 10 10 10 10 10 10 10 10 10 10 10 10 10 10 10 10 10 10 10 10 10 10 10 10 10 10 
## 53 54 55 56 57 58 59 60 61 62 63 64 65 66 67 68 69 70 71 72 73 74 75 76 77 78 
## 10 10 10 10 10 10 10 10 10 10 10 10 10 10 10 10 10 10 10 10 10 10 10 10 10 10 
## 79 80 81 82 83 84 85 86 87 88 
## 10 10 10 10 10 10 10 10 10 10
```

```
## Overall sample size
OverallN <- round(round(sum(aggregate(tas20$N, 
                                      by = list(tas20$SampleID),
                                      FUN = mean)$x, 
                            na.rm = TRUE), 
                        0))
OverallN
```

```
## [1] 69722
```

```
## Distribution of sample sizes
psych::describe(tas20$N)
```

```
##    vars   n  mean      sd median trimmed    mad min   max range skew kurtosis
## X1    1 880 792.3 1508.81    327  493.85 247.59  99 12706 12607 5.94     42.1
##       se
## X1 50.86
```

```
hist(tas20$N,
     xlab = "Sample size",
     main = "Distribution of the study sample sizes")
```

```
## Number of types of correlations
length(table(tas20$Cell))
```

```
## [1] 10
```

```
## Correlation coefficients
psych::describe(tas20$Correlation)
```

```
##    vars   n mean   sd median trimmed  mad   min  max range  skew kurtosis se
## X1    1 880 0.35 0.14   0.36    0.36 0.13 -0.19 0.73  0.92 -0.33     0.45  0
```

```
hist(tas20$Correlation,
     xlab = "Pearson correlation coefficient",
     main = "Distribution of the correlations")
```

```
## Types of correlations
table(tas20$Cell)
```

```
## 
## I11-I12 I11-I17 I12-I17  I2-I11  I2-I12  I2-I17   I2-I4  I4-I11  I4-I12  I4-I17 
##      88      88      88      88      88      88      88      88      88      88
```

```
round(prop.table(table(tas20$Cell))*100,2)
```

```
## 
## I11-I12 I11-I17 I12-I17  I2-I11  I2-I12  I2-I17   I2-I4  I4-I11  I4-I12  I4-I17 
##      10      10      10      10      10      10      10      10      10      10
```

# Constructing Sampling Covariance Matrices

As a first step, we construct the sampling variance-covariance
matrices. These matrices contain the known sampling variances in the
diagonal and, if meta-analysts decide to account for the dependencies
among sampling errors, the sampling covariances in the off-diagonal
part.

```
## Generate the sampling covariance matrices
## Upper level: Correlation matrices (independent samples)
## Note: Each sample contributes only one correlation matrix.
## Hence, SampleID identifies the correlation matrix.

## Option 1: rcalc() in metafor
## Source: https://wviechtb.github.io/metafor/reference/rcalc.html
SampCov <- metafor::rcalc(Correlation ~ Var1 + Var2 | SampleID,
                          ni = N,
                          data = tas20)

## Extract and save the sampling variance-covariance matrix
V <- SampCov$V

## Illustrate some variances for some samples
blsplit(V, tas20$SampleID)$`1`
```

```
##                I2.I4       I11.I2       I11.I4       I12.I2       I12.I4
## I2.I4   0.0013792348 0.0005541643 0.0005928562 0.0005414363 0.0005779354
## I11.I2  0.0005541643 0.0018864320 0.0009601015 0.0005191004 0.0003881954
## I11.I4  0.0005928562 0.0009601015 0.0019477260 0.0003886129 0.0005472183
## I12.I2  0.0005414363 0.0005191004 0.0003886129 0.0019997492 0.0010362279
## I12.I4  0.0005779354 0.0003881954 0.0005472183 0.0010362279 0.0020569357
## I11.I12 0.0003717069 0.0008238844 0.0008141190 0.0009130385 0.0009004462
## I17.I2  0.0004851328 0.0004669596 0.0003583757 0.0004573278 0.0003535914
## I17.I4  0.0005146787 0.0003569906 0.0004896615 0.0003526036 0.0004786334
## I11.I17 0.0003371656 0.0007143355 0.0007063234 0.0003373400 0.0003385038
## I12.I17 0.0003298670 0.0003341439 0.0003356572 0.0007595133 0.0007496397
##              I11.I12       I17.I2       I17.I4      I11.I17      I12.I17
## I2.I4   0.0003717069 0.0004851328 0.0005146787 0.0003371656 0.0003298670
## I11.I2  0.0008238844 0.0004669596 0.0003569906 0.0007143355 0.0003341439
## I11.I4  0.0008141190 0.0003583757 0.0004896615 0.0007063234 0.0003356572
## I12.I2  0.0009130385 0.0004573278 0.0003526036 0.0003373400 0.0007595133
## I12.I4  0.0009004462 0.0003535914 0.0004786334 0.0003385038 0.0007496397
## I11.I12 0.0024086814 0.0003429625 0.0003431843 0.0006150964 0.0006681053
## I17.I2  0.0003429625 0.0023571620 0.0012817871 0.0011040789 0.0010595835
## I17.I4  0.0003431843 0.0012817871 0.0024001684 0.0010839472 0.0010404156
## I11.I17 0.0006150964 0.0011040789 0.0010839472 0.0026603134 0.0009013610
## I12.I17 0.0006681053 0.0010595835 0.0010404156 0.0009013610 0.0027150820
```

```
blsplit(V, tas20$SampleID)$`10`
```

```
##                I2.I4       I11.I2       I11.I4       I12.I2       I12.I4
## I2.I4   3.183939e-03 0.0006612807 0.0007307975 3.211570e-04 3.521811e-04
## I11.I2  6.612807e-04 0.0030724080 0.0005948673 3.656506e-04 1.284567e-04
## I11.I4  7.307975e-04 0.0005948673 0.0031309768 1.288042e-04 3.765045e-04
## I12.I2  3.211570e-04 0.0003656506 0.0001288042 3.422794e-03 7.383502e-04
## I12.I4  3.521811e-04 0.0001284567 0.0003765045 7.383502e-04 3.435937e-03
## I11.I12 1.290867e-04 0.0003310148 0.0003108167 8.522392e-04 7.941852e-04
## I17.I2  4.719360e-04 0.0005375727 0.0001841941 2.614661e-04 9.729918e-05
## I17.I4  5.188252e-04 0.0001838484 0.0005547881 9.737932e-05 2.678779e-04
## I11.I17 1.844074e-04 0.0004851596 0.0004554495 1.080332e-04 1.023967e-04
## I12.I17 9.684106e-05 0.0001071104 0.0001017967 5.926863e-04 5.523854e-04
##              I11.I12       I17.I2       I17.I4      I11.I17      I12.I17
## I2.I4   0.0001290867 4.719360e-04 5.188252e-04 0.0001844074 9.684106e-05
## I11.I2  0.0003310148 5.375727e-04 1.838484e-04 0.0004851596 1.071104e-04
## I11.I4  0.0003108167 1.841941e-04 5.547881e-04 0.0004554495 1.017967e-04
## I12.I2  0.0008522392 2.614661e-04 9.737932e-05 0.0001080332 5.926863e-04
## I12.I4  0.0007941852 9.729918e-05 2.678779e-04 0.0001023967 5.523854e-04
## I11.I12 0.0034070142 1.081449e-04 1.025870e-04 0.0002538329 6.374544e-04
## I17.I2  0.0001081449 3.303608e-03 6.885794e-04 0.0007909322 3.751519e-04
## I17.I4  0.0001025870 6.885794e-04 3.332459e-03 0.0007386503 3.504972e-04
## I11.I17 0.0002538329 7.909322e-04 7.386503e-04 0.0032690651 4.022974e-04
## I12.I17 0.0006374544 3.751519e-04 3.504972e-04 0.0004022974 3.466545e-03
```

```
## Option 2: asyCov() in metaSEM
## Load the original data
load("TAS20-Original.Rdata")

# Calculate reproduced correlation matrix
rmat <- function(L, R) {
  L[is.na(L)] <- 0       # impute missing factor loadings
  mm <- L %*% R %*% t(L) # reproduced correlations
  diag(mm) <- 1
  if (!metaSEM::is.pd(mm)) # try to derive positive definite matrices
    mm <- as.matrix(Matrix::nearPD(mm, corr = TRUE, posd.tol = 1e-05, maxit = 150)$mat)
  mm[lower.tri(mm)] <- t(mm)[lower.tri(mm)] # in case of rounding errors
  colnames(mm) <- rownames(mm) <- paste0("I", seq(1, ncol(mm)))
  mm
}

# Create a list of all (reproduced) correlation matrices
cmat <- lapply(mat, function(x) {
  if (!is.null(x$rm)) {
    x$rm             # item-level correlation matrix
  } else if (!is.null(x$fm)) {
    rmat(x$fm, x$fr) # calculate reproduced correlation matrix
  }
})
rm(rmat)

## Change some names with special characters
names(cmat)[35] <- "mueller2003a"
names(cmat)[36] <- "mueller2003b"
names(cmat)[58] <- "guelec2009"


## Select the relevant variables
selected.vars <- c("I2", "I4", "I11", "I12", "I17")

## Create a new but blank list
cmatnew <- vector("list", length(cmat))

## Create a subset of variables in the matrices
for (i in 1:length(cmat)){
  ## Access the correlation matrices
  ## Subset them to the list of selected variables
  ## Write the new matrices into the new list
  cmatnew[[i]] <- cmat[[i]][selected.vars, selected.vars]
  names(cmatnew) <- names(cmatnew)

}

## Inspect some example matrices
cmatnew[1]
```

```
## [[1]]
##         I2     I4    I11   I12   I17
## I2  1.0000 0.5928 0.4914 0.468 0.390
## I4  0.5928 1.0000 0.4788 0.456 0.380
## I11 0.4914 0.4788 1.0000 0.378 0.315
## I12 0.4680 0.4560 0.3780 1.000 0.300
## I17 0.3900 0.3800 0.3150 0.300 1.000
```

```
cmatnew[10]
```

```
## [[1]]
##           I2       I4      I11      I12      I17
## I2  1.000000 0.221552 0.256688 0.118584 0.177144
## I4  0.221552 1.000000 0.238804 0.110322 0.164802
## I11 0.256688 0.238804 1.000000 0.127818 0.190938
## I12 0.118584 0.110322 0.127818 1.000000 0.088209
## I17 0.177144 0.164802 0.190938 0.088209 1.000000
```

```
## Generate a list of asymptotic covariance matrices
V.asyCov <- metaSEM::asyCov(cmatnew, 
                            dat$n,
                            cor.analysis = FALSE,
                            as.matrix = FALSE)

## Check some example matrices
V.asyCov[1]
```

```
## [[1]]
##                I2_I2        I4_I2       I11_I2       I12_I2       I17_I2
## I2_I2   0.0065359477 0.0026294872 0.0030736265 0.0024852723 0.0026176483
## I4_I2   0.0026294872 0.0037969104 0.0017138653 0.0013936459 0.0015026343
## I11_I2  0.0030736265 0.0017138653 0.0039906831 0.0016977444 0.0017986911
## I12_I2  0.0024852723 0.0013936459 0.0016977444 0.0037404821 0.0014682715
## I17_I2  0.0026176483 0.0015026343 0.0017986911 0.0014682715 0.0037921582
## I4_I4   0.0010578731 0.0026294872 0.0008815351 0.0007191070 0.0007853759
## I11_I4  0.0012365554 0.0019775808 0.0018299599 0.0008682088 0.0009350296
## I12_I4  0.0009998537 0.0016021896 0.0008645177 0.0016545773 0.0007616326
## I17_I4  0.0010531101 0.0017015121 0.0009147969 0.0007484164 0.0017056635
## I11_I11 0.0014454185 0.0010304327 0.0030736265 0.0010471635 0.0011128320
## I12_I11 0.0011687362 0.0008368782 0.0017662179 0.0019601709 0.0009063437
## I17_I11 0.0012309880 0.0008977988 0.0018652402 0.0009023444 0.0020106839
## I12_I12 0.0009450165 0.0006796674 0.0008467153 0.0024852723 0.0007381314
## I17_I12 0.0009953520 0.0007290855 0.0008958142 0.0016778898 0.0016313598
## I17_I17 0.0010483687 0.0007818398 0.0009477413 0.0007774473 0.0026176483
##                I4_I4       I11_I4       I12_I4       I17_I4      I11_I11
## I2_I2   0.0010578731 0.0012365554 0.0009998537 0.0010531101 0.0014454185
## I4_I2   0.0026294872 0.0019775808 0.0016021896 0.0017015121 0.0010304327
## I11_I2  0.0008815351 0.0018299599 0.0008645177 0.0009147969 0.0030736265
## I12_I2  0.0007191070 0.0008682088 0.0016545773 0.0007484164 0.0010471635
## I17_I2  0.0007853759 0.0009350296 0.0007616326 0.0017056635 0.0011128320
## I4_I4   0.0065359477 0.0021911752 0.0017874381 0.0019521585 0.0007345911
## I11_I4  0.0021911752 0.0036352694 0.0014129955 0.0015104275 0.0021911752
## I12_I4  0.0017874381 0.0014129955 0.0035123864 0.0012375317 0.0007465184
## I17_I4  0.0019521585 0.0015104275 0.0012375317 0.0035595094 0.0007933332
## I11_I11 0.0007345911 0.0021911752 0.0007465184 0.0007933332 0.0065359477
## I12_I11 0.0005992383 0.0012669782 0.0014000716 0.0006489704 0.0022267527
## I17_I11 0.0006544608 0.0013727458 0.0006579355 0.0014489852 0.0023663942
## I12_I12 0.0004888250 0.0006089679 0.0017874381 0.0005308731 0.0007586394
## I17_I12 0.0005338724 0.0006561219 0.0012415158 0.0011836167 0.0008062143
## I17_I17 0.0005830712 0.0007067952 0.0005797954 0.0019521585 0.0008567727
##              I12_I11      I17_I11      I12_I12      I17_I12      I17_I17
## I2_I2   0.0011687362 0.0012309880 0.0009450165 0.0009953520 0.0010483687
## I4_I2   0.0008368782 0.0008977988 0.0006796674 0.0007290855 0.0007818398
## I11_I2  0.0017662179 0.0018652402 0.0008467153 0.0008958142 0.0009477413
## I12_I2  0.0019601709 0.0009023444 0.0024852723 0.0016778898 0.0007774473
## I17_I2  0.0009063437 0.0020106839 0.0007381314 0.0016313598 0.0026176483
## I4_I4   0.0005992383 0.0006544608 0.0004888250 0.0005338724 0.0005830712
## I11_I4  0.0012669782 0.0013727458 0.0006089679 0.0006561219 0.0007067952
## I12_I4  0.0014000716 0.0006579355 0.0017874381 0.0012415158 0.0005797954
## I17_I4  0.0006489704 0.0014489852 0.0005308731 0.0011836167 0.0019521585
## I11_I11 0.0022267527 0.0023663942 0.0007586394 0.0008062143 0.0008567727
## I12_I11 0.0036472935 0.0013737026 0.0022267527 0.0015138723 0.0007028243
## I17_I11 0.0013737026 0.0036963602 0.0006613505 0.0014647885 0.0023663942
## I12_I12 0.0022267527 0.0006613505 0.0065359477 0.0019411909 0.0005765380
## I17_I12 0.0015138723 0.0014647885 0.0019411909 0.0035562429 0.0019411909
## I17_I17 0.0007028243 0.0023663942 0.0005765380 0.0019411909 0.0065359477
```

```
V.asyCov[10]
```

```
## [[1]]
##               I2_I2        I4_I2       I11_I2       I12_I2       I17_I2
## I2_I2   0.007017544 0.0028232389 0.0033001042 0.0026683976 0.0028105277
## I4_I2   0.002823239 0.0040766827 0.0018401501 0.0014963356 0.0016133547
## I11_I2  0.003300104 0.0018401501 0.0042847334 0.0018228414 0.0019312262
## I12_I2  0.002668398 0.0014963356 0.0018228414 0.0040160966 0.0015764599
## I17_I2  0.002810528 0.0016133547 0.0019312262 0.0015764599 0.0040715804
## I4_I4   0.001135822 0.0028232389 0.0009464903 0.0007720938 0.0008432457
## I11_I4  0.001327670 0.0021232973 0.0019647991 0.0009321821 0.0010039265
## I12_I4  0.001073527 0.0017202457 0.0009282190 0.0017764935 0.0008177529
## I17_I4  0.001130708 0.0018268867 0.0009822030 0.0008035629 0.0018313440
## I11_I11 0.001551923 0.0011063593 0.0033001042 0.0011243229 0.0011948301
## I12_I11 0.001254854 0.0008985429 0.0018963603 0.0021046045 0.0009731269
## I17_I11 0.001321692 0.0009639524 0.0020026789 0.0009688329 0.0021588395
## I12_I12 0.001014649 0.0007297481 0.0009091048 0.0026683976 0.0007925200
## I17_I12 0.001068694 0.0007828076 0.0009618215 0.0018015238 0.0017515653
## I17_I17 0.001125617 0.0008394491 0.0010175749 0.0008347329 0.0028105277
##                I4_I4       I11_I4       I12_I4       I17_I4      I11_I11
## I2_I2   0.0011358216 0.0013276700 0.0010735272 0.0011307077 0.0015519230
## I4_I2   0.0028232389 0.0021232973 0.0017202457 0.0018268867 0.0011063593
## I11_I2  0.0009464903 0.0019647991 0.0009282190 0.0009822030 0.0033001042
## I12_I2  0.0007720938 0.0009321821 0.0017764935 0.0008035629 0.0011243229
## I17_I2  0.0008432457 0.0010039265 0.0008177529 0.0018313440 0.0011948301
## I4_I4   0.0070175439 0.0023526303 0.0019191440 0.0020960017 0.0007887188
## I11_I4  0.0023526303 0.0039031314 0.0015171109 0.0016217221 0.0023526303
## I12_I4  0.0019191440 0.0015171109 0.0037711938 0.0013287182 0.0008015250
## I17_I4  0.0020960017 0.0016217221 0.0013287182 0.0038217891 0.0008517893
## I11_I11 0.0007887188 0.0023526303 0.0008015250 0.0008517893 0.0070175439
## I12_I11 0.0006433927 0.0013603345 0.0015032347 0.0006967892 0.0023908292
## I17_I11 0.0007026842 0.0014738955 0.0007064149 0.0015557525 0.0025407601
## I12_I12 0.0005248437 0.0006538392 0.0019191440 0.0005699900 0.0008145391
## I17_I12 0.0005732104 0.0007044677 0.0013329959 0.0012708306 0.0008656196
## I17_I17 0.0006260343 0.0007588748 0.0006225172 0.0020960017 0.0009199033
##              I12_I11      I17_I11      I12_I12      I17_I12      I17_I17
## I2_I2   0.0012548536 0.0013216924 0.0010146493 0.0010686938 0.0011256169
## I4_I2   0.0008985429 0.0009639524 0.0007297481 0.0007828076 0.0008394491
## I11_I2  0.0018963603 0.0020026789 0.0009091048 0.0009618215 0.0010175749
## I12_I2  0.0021046045 0.0009688329 0.0026683976 0.0018015238 0.0008347329
## I17_I2  0.0009731269 0.0021588395 0.0007925200 0.0017515653 0.0028105277
## I4_I4   0.0006433927 0.0007026842 0.0005248437 0.0005732104 0.0006260343
## I11_I4  0.0013603345 0.0014738955 0.0006538392 0.0007044677 0.0007588748
## I12_I4  0.0015032347 0.0007064149 0.0019191440 0.0013329959 0.0006225172
## I17_I4  0.0006967892 0.0015557525 0.0005699900 0.0012708306 0.0020960017
## I11_I11 0.0023908292 0.0025407601 0.0008145391 0.0008656196 0.0009199033
## I12_I11 0.0039160415 0.0014749228 0.0023908292 0.0016254208 0.0007546114
## I17_I11 0.0014749228 0.0039687236 0.0007100815 0.0015727203 0.0025407601
## I12_I12 0.0023908292 0.0007100815 0.0070175439 0.0020842260 0.0006190197
## I17_I12 0.0016254208 0.0015727203 0.0020842260 0.0038182818 0.0020842260
## I17_I17 0.0007546114 0.0025407601 0.0006190197 0.0020842260 0.0070175439
```

# Pooling Correlation Matrices Meta-Analytically

As a next step, we pooled the correlation matrices via several
multilevel, multivariate, and random-effects models. These models
contain different assumptions on the within- and between-study random
effects and hence the variance estimates in these models. In other
words, these models quantify heterogeneity in the meta-analytic data
under different assumptions.

## Multilevel Multivariate Random-Effects Models Accounting for Hierarchical Effect Size Multiplicity

### Model with level-specific variance estimates (`Model 1`)

This model assumes that each type of pooled correlation has its own
set of estimates of the amounts of residual heterogeneity within and
between studies (`mlmvrem1`).

The structure of random effects follows a heteroscedastic compound
symmetry (`HCS`) structure. We vary the \(\rho\) and \(\phi\) parameters in this structure, using
values of 0, 0.5, and 1 to study the sensitivity of parameter choice. In
our view, selecting values of these correlations should be informed by
substantive reasons, theories, and evidence.

```
## Random effects: rho = 0, phi = 0
## Source: https://wviechtb.github.io/metafor/reference/rma.mv.html

## Model specification
tas20.mlmvrem1 <- rma.mv(yi = Correlation,
                         V = V, 
                         data = tas20,
                         random = list(~ factor(Cell) | ESID,
                                       ~ factor(Cell) | StudyID),
                         struc = c("HCS", "HCS"),
                         rho = 0,
                         phi = 0,
                         method = "REML",
                         mods = ~ factor(Cell) - 1,
                         time = TRUE,
                         sparse = TRUE,
                         control = list(optimizer = "optimParallel",
                                        ncpus = ncores))
```

```
## 
## Processing time: 0 hours, 0 minutes, 14.45 seconds
```

```
## Model summary
summary(tas20.mlmvrem1)
```

```
## 
## Multivariate Meta-Analysis Model (k = 880; method: REML)
## 
##     logLik    Deviance         AIC         BIC        AICc   
##   728.3722  -1456.7443  -1396.7443  -1253.6895  -1394.5274   
## 
## Variance Components:
## 
## outer factor: ESID         (nlvls = 880)
## inner factor: factor(Cell) (nlvls = 10)
## 
##              estim    sqrt  k.lvl  fixed    level 
## tau^2.1     0.0008  0.0289     88     no  I11-I12 
## tau^2.2     0.0006  0.0248     88     no  I11-I17 
## tau^2.3     0.0015  0.0382     88     no  I12-I17 
## tau^2.4     0.0004  0.0187     88     no   I2-I11 
## tau^2.5     0.0020  0.0450     88     no   I2-I12 
## tau^2.6     0.0022  0.0474     88     no   I2-I17 
## tau^2.7     0.0088  0.0940     88     no    I2-I4 
## tau^2.8     0.0032  0.0564     88     no   I4-I11 
## tau^2.9     0.0028  0.0532     88     no   I4-I12 
## tau^2.10    0.0045  0.0671     88     no   I4-I17 
## rho         0.0000                   yes          
## 
## outer factor: StudyID      (nlvls = 62)
## inner factor: factor(Cell) (nlvls = 10)
## 
##                estim    sqrt  k.lvl  fixed    level 
## gamma^2.1     0.0049  0.0698     62     no  I11-I12 
## gamma^2.2     0.0049  0.0701     62     no  I11-I17 
## gamma^2.3     0.0066  0.0810     62     no  I12-I17 
## gamma^2.4     0.0039  0.0626     62     no   I2-I11 
## gamma^2.5     0.0050  0.0708     62     no   I2-I12 
## gamma^2.6     0.0052  0.0718     62     no   I2-I17 
## gamma^2.7     0.0102  0.1009     62     no    I2-I4 
## gamma^2.8     0.0073  0.0855     62     no   I4-I11 
## gamma^2.9     0.0099  0.0996     62     no   I4-I12 
## gamma^2.10    0.0085  0.0920     62     no   I4-I17 
## phi           0.0000                   yes          
## 
## Test for Residual Heterogeneity:
## QE(df = 870) = 6986.5768, p-val < .0001
## 
## Test of Moderators (coefficients 1:10):
## QM(df = 10) = 6774.8436, p-val < .0001
## 
## Model Results:
## 
##                      estimate      se     zval    pval   ci.lb   ci.ub      
## factor(Cell)I11-I12    0.3143  0.0108  29.1040  <.0001  0.2932  0.3355  *** 
## factor(Cell)I11-I17    0.3470  0.0106  32.6257  <.0001  0.3261  0.3678  *** 
## factor(Cell)I12-I17    0.2807  0.0124  22.6569  <.0001  0.2564  0.3049  *** 
## factor(Cell)I2-I11     0.4497  0.0094  47.6001  <.0001  0.4312  0.4682  *** 
## factor(Cell)I2-I12     0.3515  0.0115  30.5204  <.0001  0.3289  0.3740  *** 
## factor(Cell)I2-I17     0.3857  0.0117  33.0677  <.0001  0.3628  0.4085  *** 
## factor(Cell)I2-I4      0.4219  0.0173  24.4292  <.0001  0.3880  0.4557  *** 
## factor(Cell)I4-I11     0.3499  0.0136  25.7520  <.0001  0.3233  0.3766  *** 
## factor(Cell)I4-I12     0.2794  0.0150  18.5948  <.0001  0.2500  0.3089  *** 
## factor(Cell)I4-I17     0.3142  0.0149  21.1215  <.0001  0.2851  0.3434  *** 
## 
## ---
## Signif. codes:  0 '***' 0.001 '**' 0.01 '*' 0.05 '.' 0.1 ' ' 1
```

```
## Cluster-robust standard errors
tas20.mlmvrem1.robust <- robust(tas20.mlmvrem1, 
                                cluster = StudyID, 
                                clubSandwich = TRUE)
summary(tas20.mlmvrem1.robust)
```

```
## 
## Multivariate Meta-Analysis Model (k = 880; method: REML)
## 
##     logLik    Deviance         AIC         BIC        AICc   
##   728.3722  -1456.7443  -1396.7443  -1253.6895  -1394.5274   
## 
## Variance Components:
## 
## outer factor: ESID         (nlvls = 880)
## inner factor: factor(Cell) (nlvls = 10)
## 
##              estim    sqrt  k.lvl  fixed    level 
## tau^2.1     0.0008  0.0289     88     no  I11-I12 
## tau^2.2     0.0006  0.0248     88     no  I11-I17 
## tau^2.3     0.0015  0.0382     88     no  I12-I17 
## tau^2.4     0.0004  0.0187     88     no   I2-I11 
## tau^2.5     0.0020  0.0450     88     no   I2-I12 
## tau^2.6     0.0022  0.0474     88     no   I2-I17 
## tau^2.7     0.0088  0.0940     88     no    I2-I4 
## tau^2.8     0.0032  0.0564     88     no   I4-I11 
## tau^2.9     0.0028  0.0532     88     no   I4-I12 
## tau^2.10    0.0045  0.0671     88     no   I4-I17 
## rho         0.0000                   yes          
## 
## outer factor: StudyID      (nlvls = 62)
## inner factor: factor(Cell) (nlvls = 10)
## 
##                estim    sqrt  k.lvl  fixed    level 
## gamma^2.1     0.0049  0.0698     62     no  I11-I12 
## gamma^2.2     0.0049  0.0701     62     no  I11-I17 
## gamma^2.3     0.0066  0.0810     62     no  I12-I17 
## gamma^2.4     0.0039  0.0626     62     no   I2-I11 
## gamma^2.5     0.0050  0.0708     62     no   I2-I12 
## gamma^2.6     0.0052  0.0718     62     no   I2-I17 
## gamma^2.7     0.0102  0.1009     62     no    I2-I4 
## gamma^2.8     0.0073  0.0855     62     no   I4-I11 
## gamma^2.9     0.0099  0.0996     62     no   I4-I12 
## gamma^2.10    0.0085  0.0920     62     no   I4-I17 
## phi           0.0000                   yes          
## 
## Test for Residual Heterogeneity:
## QE(df = 870) = 6986.5768, p-val < .0001
## 
## Number of estimates:   880
## Number of clusters:    62
## Estimates per cluster: 10-40 (mean: 14.19, median: 10)
## 
## Test of Moderators (coefficients 1:10):¹
## F(df1 = 10, df2 = 50.39) = 191.5031, p-val < .0001
## 
## Model Results:
## 
##                      estimate      se¹     tval¹     df¹    pval¹   ci.lb¹ 
## factor(Cell)I11-I12    0.3143  0.0134   23.5071   58.76   <.0001   0.2876  
## factor(Cell)I11-I17    0.3470  0.0130   26.6493   59.03   <.0001   0.3209  
## factor(Cell)I12-I17    0.2807  0.0151   18.5416   59.43   <.0001   0.2504  
## factor(Cell)I2-I11     0.4497  0.0114   39.3837   58.81   <.0001   0.4269  
## factor(Cell)I2-I12     0.3515  0.0142   24.6754   58.81   <.0001   0.3230  
## factor(Cell)I2-I17     0.3857  0.0141   27.3739   59.02   <.0001   0.3575  
## factor(Cell)I2-I4      0.4219  0.0193   21.8133   59.27   <.0001   0.3832  
## factor(Cell)I4-I11     0.3499  0.0159   22.0186   59.51   <.0001   0.3181  
## factor(Cell)I4-I12     0.2794  0.0176   15.8676   59.95   <.0001   0.2442  
## factor(Cell)I4-I17     0.3142  0.0174   18.0085   59.48   <.0001   0.2793  
##                       ci.ub¹      
## factor(Cell)I11-I12  0.3411   *** 
## factor(Cell)I11-I17  0.3730   *** 
## factor(Cell)I12-I17  0.3109   *** 
## factor(Cell)I2-I11   0.4726   *** 
## factor(Cell)I2-I12   0.3800   *** 
## factor(Cell)I2-I17   0.4139   *** 
## factor(Cell)I2-I4    0.4605   *** 
## factor(Cell)I4-I11   0.3817   *** 
## factor(Cell)I4-I12   0.3147   *** 
## factor(Cell)I4-I17   0.3491   *** 
## 
## ---
## Signif. codes:  0 '***' 0.001 '**' 0.01 '*' 0.05 '.' 0.1 ' ' 1
## 
## 1) results based on cluster-robust inference (var-cov estimator: CR2,
##    approx t/F-tests and confidence intervals, df: Satterthwaite approx)
```

```
## Processing time in seconds
tas20.mlmvrem1$time
```

```
## [1] 14.451
```

```
## Extract relevant elements
## Asymptotic covariance matrix
ACOV.mlmvrem1 <- tas20.mlmvrem1$vb

## Marginal differences between the asymptotic covariance matrices
## cluster-robust vs. not robust
max(tas20.mlmvrem1$vb-tas20.mlmvrem1.robust$vb)
```

```
## [1] -4.112846e-05
```

```
## Pooled correlation matrix
CORR.mlmvrem1 <- vec2symMat(x = c(1, tas20.mlmvrem1$b[7], tas20.mlmvrem1$b[4], tas20.mlmvrem1$b[5], tas20.mlmvrem1$b[6],
                                1, tas20.mlmvrem1$b[8], tas20.mlmvrem1$b[9], tas20.mlmvrem1$b[10],
                                1, tas20.mlmvrem1$b[1], tas20.mlmvrem1$b[2],
                                1, tas20.mlmvrem1$b[3],
                                1),
                          diag = TRUE)

colnames(CORR.mlmvrem1) <- c("Item2", "Item4", "Item11", "Item12", "Item17")
rownames(CORR.mlmvrem1) <- c("Item2", "Item4", "Item11", "Item12", "Item17")

## Inspect the pooled correlation matrix
CORR.mlmvrem1
```

```
##            Item2     Item4    Item11    Item12    Item17
## Item2  1.0000000 0.4218548 0.4497150 0.3514609 0.3856644
## Item4  0.4218548 1.0000000 0.3499278 0.2794383 0.3142220
## Item11 0.4497150 0.3499278 1.0000000 0.3143325 0.3469820
## Item12 0.3514609 0.2794383 0.3143325 1.0000000 0.2806570
## Item17 0.3856644 0.3142220 0.3469820 0.2806570 1.0000000
```

```
## Plot the pooled correlation matrix
corrplot(CORR.mlmvrem1, 
         type = "upper", 
         order = "original", 
         tl.col = "black", 
         tl.srt = 60,
         addCoef.col = "black",
         number.cex = 0.9,
         cl.cex = 1,
         tl.cex = 1)
```

```
## Extract the relevant information
mlmvrem1.results <- data.frame(round(tas20.mlmvrem1.robust$b[,1],3), 
                               round(tas20.mlmvrem1.robust$se,3),
                               round(tas20.mlmvrem1.robust$ci.lb,3),
                               round(tas20.mlmvrem1.robust$ci.ub,3),
                               round(tas20.mlmvrem1.robust$tau2,3),
                               round(tas20.mlmvrem1.robust$gamma2,3))

colnames(mlmvrem1.results) <- c("r",
                                "SE",
                                "CI95-low",
                                "CI95-high",
                                "tau2",
                                "gamma2")

## Data frame with results
mlmvrem1.results
```

```
##                         r    SE CI95-low CI95-high  tau2 gamma2
## factor(Cell)I11-I12 0.314 0.013    0.288     0.341 0.001  0.005
## factor(Cell)I11-I17 0.347 0.013    0.321     0.373 0.001  0.005
## factor(Cell)I12-I17 0.281 0.015    0.250     0.311 0.001  0.007
## factor(Cell)I2-I11  0.450 0.011    0.427     0.473 0.000  0.004
## factor(Cell)I2-I12  0.351 0.014    0.323     0.380 0.002  0.005
## factor(Cell)I2-I17  0.386 0.014    0.357     0.414 0.002  0.005
## factor(Cell)I2-I4   0.422 0.019    0.383     0.461 0.009  0.010
## factor(Cell)I4-I11  0.350 0.016    0.318     0.382 0.003  0.007
## factor(Cell)I4-I12  0.279 0.018    0.244     0.315 0.003  0.010
## factor(Cell)I4-I17  0.314 0.017    0.279     0.349 0.005  0.008
```

```
##write.csv2(mlmvrem1.results, file = "MLMVREM-Table2-mlmvrem1.csv")


## Model fit
metafor::fitstats(tas20.mlmvrem1)
```

```
##                 REML
## logLik:     728.3722
## deviance: -1456.7443
## AIC:      -1396.7443
## BIC:      -1253.6895
## AICc:     -1394.5274
```

```
logLik.rma(tas20.mlmvrem1)
```

```
## 'log Lik.' 728.3722 (df=30)
```

```
### Create a new model fit data frame
newfit.mlmvrem1 <- data.frame(
  c("logLik", "parms", "k",
    "Deviance", 
    "AIC", "BIC", "AICc"),
  c(## logLik
    tas20.mlmvrem1$fit.stats$REML[1],
    ## Number of parameters (parms)
    tas20.mlmvrem1$parms,
    ## Number of effect sizes (k)
    length(tas20$Correlation),
    ## Deviance
    tas20.mlmvrem1$fit.stats$REML[2],
    ## AIC
    tas20.mlmvrem1$fit.stats$REML[3],
    ## BIC
    tas20.mlmvrem1$fit.stats$REML[4],
    ## AICc
    tas20.mlmvrem1$fit.stats$REML[5]))

colnames(newfit.mlmvrem1) <- c("Fit criterion", "mlmvrem1")
newfit.mlmvrem1
```

```
##   Fit criterion   mlmvrem1
## 1        logLik   728.3722
## 2         parms    30.0000
## 3             k   880.0000
## 4      Deviance -1456.7443
## 5           AIC -1396.7443
## 6           BIC -1253.6895
## 7          AICc -1394.5274
```

```
## Sensitivity analyses
## Rho = Phi = 0.5
## Model specification
tas20.mlmvrem1.sens <- rma.mv(yi = Correlation,
                              V = V, 
                              data = tas20,
                              random = list(~ factor(Cell) | ESID,
                                            ~ factor(Cell) | StudyID),
                              struc = c("HCS", "HCS"),
                              rho = 0.5,
                              phi = 0.5,
                              method = "REML",
                              mods = ~ factor(Cell) - 1,
                              time = TRUE,
                              sparse = TRUE,
                              control = list(optimizer = "optimParallel",
                                             ncpus = ncores))
```

```
## 
## Processing time: 0 hours, 1 minute, 4 seconds
```

```
## Model summary
summary(tas20.mlmvrem1.sens)
```

```
## 
## Multivariate Meta-Analysis Model (k = 880; method: REML)
## 
##     logLik    Deviance         AIC         BIC        AICc   
##   914.5862  -1829.1723  -1769.1723  -1626.1175  -1766.9554   
## 
## Variance Components:
## 
## outer factor: ESID         (nlvls = 880)
## inner factor: factor(Cell) (nlvls = 10)
## 
##              estim    sqrt  k.lvl  fixed    level 
## tau^2.1     0.0004  0.0206     88     no  I11-I12 
## tau^2.2     0.0003  0.0187     88     no  I11-I17 
## tau^2.3     0.0007  0.0265     88     no  I12-I17 
## tau^2.4     0.0002  0.0140     88     no   I2-I11 
## tau^2.5     0.0011  0.0332     88     no   I2-I12 
## tau^2.6     0.0013  0.0366     88     no   I2-I17 
## tau^2.7     0.0056  0.0748     88     no    I2-I4 
## tau^2.8     0.0017  0.0415     88     no   I4-I11 
## tau^2.9     0.0019  0.0438     88     no   I4-I12 
## tau^2.10    0.0024  0.0490     88     no   I4-I17 
## rho         0.5000                   yes          
## 
## outer factor: StudyID      (nlvls = 62)
## inner factor: factor(Cell) (nlvls = 10)
## 
##                estim    sqrt  k.lvl  fixed    level 
## gamma^2.1     0.0047  0.0685     62     no  I11-I12 
## gamma^2.2     0.0045  0.0673     62     no  I11-I17 
## gamma^2.3     0.0062  0.0784     62     no  I12-I17 
## gamma^2.4     0.0036  0.0597     62     no   I2-I11 
## gamma^2.5     0.0050  0.0709     62     no   I2-I12 
## gamma^2.6     0.0049  0.0699     62     no   I2-I17 
## gamma^2.7     0.0098  0.0991     62     no    I2-I4 
## gamma^2.8     0.0068  0.0825     62     no   I4-I11 
## gamma^2.9     0.0082  0.0907     62     no   I4-I12 
## gamma^2.10    0.0081  0.0901     62     no   I4-I17 
## phi           0.5000                   yes          
## 
## Test for Residual Heterogeneity:
## QE(df = 870) = 6986.5768, p-val < .0001
## 
## Test of Moderators (coefficients 1:10):
## QM(df = 10) = 3053.3055, p-val < .0001
## 
## Model Results:
## 
##                      estimate      se     zval    pval   ci.lb   ci.ub      
## factor(Cell)I11-I12    0.3195  0.0104  30.7312  <.0001  0.2991  0.3399  *** 
## factor(Cell)I11-I17    0.3510  0.0102  34.5846  <.0001  0.3311  0.3709  *** 
## factor(Cell)I12-I17    0.2848  0.0117  24.3465  <.0001  0.2619  0.3078  *** 
## factor(Cell)I2-I11     0.4536  0.0090  50.4116  <.0001  0.4359  0.4712  *** 
## factor(Cell)I2-I12     0.3566  0.0110  32.3956  <.0001  0.3350  0.3782  *** 
## factor(Cell)I2-I17     0.3897  0.0110  35.5823  <.0001  0.3683  0.4112  *** 
## factor(Cell)I2-I4      0.4264  0.0159  26.8703  <.0001  0.3953  0.4575  *** 
## factor(Cell)I4-I11     0.3569  0.0126  28.3880  <.0001  0.3322  0.3815  *** 
## factor(Cell)I4-I12     0.2873  0.0137  21.0309  <.0001  0.2605  0.3140  *** 
## factor(Cell)I4-I17     0.3214  0.0138  23.3426  <.0001  0.2944  0.3484  *** 
## 
## ---
## Signif. codes:  0 '***' 0.001 '**' 0.01 '*' 0.05 '.' 0.1 ' ' 1
```

```
## Cluster-robust standard errors
summary(
  robust(tas20.mlmvrem1.sens,
         cluster = StudyID,
         clubSandwich = TRUE)
)
```

```
## 
## Multivariate Meta-Analysis Model (k = 880; method: REML)
## 
##     logLik    Deviance         AIC         BIC        AICc   
##   914.5862  -1829.1723  -1769.1723  -1626.1175  -1766.9554   
## 
## Variance Components:
## 
## outer factor: ESID         (nlvls = 880)
## inner factor: factor(Cell) (nlvls = 10)
## 
##              estim    sqrt  k.lvl  fixed    level 
## tau^2.1     0.0004  0.0206     88     no  I11-I12 
## tau^2.2     0.0003  0.0187     88     no  I11-I17 
## tau^2.3     0.0007  0.0265     88     no  I12-I17 
## tau^2.4     0.0002  0.0140     88     no   I2-I11 
## tau^2.5     0.0011  0.0332     88     no   I2-I12 
## tau^2.6     0.0013  0.0366     88     no   I2-I17 
## tau^2.7     0.0056  0.0748     88     no    I2-I4 
## tau^2.8     0.0017  0.0415     88     no   I4-I11 
## tau^2.9     0.0019  0.0438     88     no   I4-I12 
## tau^2.10    0.0024  0.0490     88     no   I4-I17 
## rho         0.5000                   yes          
## 
## outer factor: StudyID      (nlvls = 62)
## inner factor: factor(Cell) (nlvls = 10)
## 
##                estim    sqrt  k.lvl  fixed    level 
## gamma^2.1     0.0047  0.0685     62     no  I11-I12 
## gamma^2.2     0.0045  0.0673     62     no  I11-I17 
## gamma^2.3     0.0062  0.0784     62     no  I12-I17 
## gamma^2.4     0.0036  0.0597     62     no   I2-I11 
## gamma^2.5     0.0050  0.0709     62     no   I2-I12 
## gamma^2.6     0.0049  0.0699     62     no   I2-I17 
## gamma^2.7     0.0098  0.0991     62     no    I2-I4 
## gamma^2.8     0.0068  0.0825     62     no   I4-I11 
## gamma^2.9     0.0082  0.0907     62     no   I4-I12 
## gamma^2.10    0.0081  0.0901     62     no   I4-I17 
## phi           0.5000                   yes          
## 
## Test for Residual Heterogeneity:
## QE(df = 870) = 6986.5768, p-val < .0001
## 
## Number of estimates:   880
## Number of clusters:    62
## Estimates per cluster: 10-40 (mean: 14.19, median: 10)
## 
## Test of Moderators (coefficients 1:10):¹
## F(df1 = 10, df2 = 49.37) = 189.2668, p-val < .0001
## 
## Model Results:
## 
##                      estimate      se¹     tval¹     df¹    pval¹   ci.lb¹ 
## factor(Cell)I11-I12    0.3195  0.0137   23.2513   58.62   <.0001   0.2920  
## factor(Cell)I11-I17    0.3510  0.0133   26.3171    58.8   <.0001   0.3243  
## factor(Cell)I12-I17    0.2848  0.0155   18.4358   59.34   <.0001   0.2539  
## factor(Cell)I2-I11     0.4536  0.0117   38.8677   58.53   <.0001   0.4302  
## factor(Cell)I2-I12     0.3566  0.0143   24.8618    58.9   <.0001   0.3279  
## factor(Cell)I2-I17     0.3897  0.0141   27.6743   59.01   <.0001   0.3616  
## factor(Cell)I2-I4      0.4264  0.0196   21.7658   59.69   <.0001   0.3872  
## factor(Cell)I4-I11     0.3569  0.0159   22.4075   59.59   <.0001   0.3250  
## factor(Cell)I4-I12     0.2873  0.0176   16.3634   59.72   <.0001   0.2522  
## factor(Cell)I4-I17     0.3214  0.0176   18.2465    59.7   <.0001   0.2861  
##                       ci.ub¹      
## factor(Cell)I11-I12  0.3470   *** 
## factor(Cell)I11-I17  0.3777   *** 
## factor(Cell)I12-I17  0.3157   *** 
## factor(Cell)I2-I11   0.4769   *** 
## factor(Cell)I2-I12   0.3853   *** 
## factor(Cell)I2-I17   0.4179   *** 
## factor(Cell)I2-I4    0.4656   *** 
## factor(Cell)I4-I11   0.3887   *** 
## factor(Cell)I4-I12   0.3224   *** 
## factor(Cell)I4-I17   0.3566   *** 
## 
## ---
## Signif. codes:  0 '***' 0.001 '**' 0.01 '*' 0.05 '.' 0.1 ' ' 1
## 
## 1) results based on cluster-robust inference (var-cov estimator: CR2,
##    approx t/F-tests and confidence intervals, df: Satterthwaite approx)
```

```
## Sensitivity analyses
## Rho = 0, Phi is estimated
## Model specification
tas20.mlmvrem1.sense <- rma.mv(yi = Correlation,
                              V = V, 
                              data = tas20,
                              random = list(~ factor(Cell) | ESID,
                                            ~ factor(Cell) | StudyID),
                              struc = c("HCS", "HCS"),
                              rho = 0,
                              method = "REML",
                              mods = ~ factor(Cell) - 1,
                              time = TRUE,
                              sparse = TRUE,
                              control = list(optimizer = "optimParallel",
                                             ncpus = ncores))
```

```
## 
## Processing time: 0 hours, 1 minute, 6 seconds
```

```
## Model summary
summary(tas20.mlmvrem1.sense)
```

```
## 
## Multivariate Meta-Analysis Model (k = 880; method: REML)
## 
##     logLik    Deviance         AIC         BIC        AICc   
##   953.4645  -1906.9291  -1844.9291  -1697.1058  -1842.5615   
## 
## Variance Components:
## 
## outer factor: ESID         (nlvls = 880)
## inner factor: factor(Cell) (nlvls = 10)
## 
##              estim    sqrt  k.lvl  fixed    level 
## tau^2.1     0.0011  0.0330     88     no  I11-I12 
## tau^2.2     0.0011  0.0338     88     no  I11-I17 
## tau^2.3     0.0010  0.0320     88     no  I12-I17 
## tau^2.4     0.0011  0.0326     88     no   I2-I11 
## tau^2.5     0.0014  0.0368     88     no   I2-I12 
## tau^2.6     0.0019  0.0441     88     no   I2-I17 
## tau^2.7     0.0077  0.0876     88     no    I2-I4 
## tau^2.8     0.0028  0.0525     88     no   I4-I11 
## tau^2.9     0.0033  0.0578     88     no   I4-I12 
## tau^2.10    0.0032  0.0570     88     no   I4-I17 
## rho         0.0000                   yes          
## 
## outer factor: StudyID      (nlvls = 62)
## inner factor: factor(Cell) (nlvls = 10)
## 
##                estim    sqrt  k.lvl  fixed    level 
## gamma^2.1     0.0088  0.0940     62     no  I11-I12 
## gamma^2.2     0.0079  0.0891     62     no  I11-I17 
## gamma^2.3     0.0122  0.1105     62     no  I12-I17 
## gamma^2.4     0.0060  0.0774     62     no   I2-I11 
## gamma^2.5     0.0101  0.1007     62     no   I2-I12 
## gamma^2.6     0.0090  0.0947     62     no   I2-I17 
## gamma^2.7     0.0149  0.1221     62     no    I2-I4 
## gamma^2.8     0.0116  0.1078     62     no   I4-I11 
## gamma^2.9     0.0137  0.1171     62     no   I4-I12 
## gamma^2.10    0.0149  0.1223     62     no   I4-I17 
## phi           0.9252                    no          
## 
## Test for Residual Heterogeneity:
## QE(df = 870) = 6986.5768, p-val < .0001
## 
## Test of Moderators (coefficients 1:10):
## QM(df = 10) = 2260.3056, p-val < .0001
## 
## Model Results:
## 
##                      estimate      se     zval    pval   ci.lb   ci.ub      
## factor(Cell)I11-I12    0.3220  0.0135  23.9160  <.0001  0.2956  0.3484  *** 
## factor(Cell)I11-I17    0.3525  0.0129  27.3645  <.0001  0.3273  0.3778  *** 
## factor(Cell)I12-I17    0.2868  0.0154  18.6393  <.0001  0.2566  0.3170  *** 
## factor(Cell)I2-I11     0.4544  0.0114  40.0026  <.0001  0.4321  0.4767  *** 
## factor(Cell)I2-I12     0.3600  0.0143  25.1522  <.0001  0.3320  0.3881  *** 
## factor(Cell)I2-I17     0.3926  0.0138  28.3864  <.0001  0.3655  0.4197  *** 
## factor(Cell)I2-I4      0.4288  0.0188  22.7805  <.0001  0.3919  0.4657  *** 
## factor(Cell)I4-I11     0.3607  0.0157  23.0131  <.0001  0.3300  0.3914  *** 
## factor(Cell)I4-I12     0.2926  0.0170  17.1826  <.0001  0.2592  0.3260  *** 
## factor(Cell)I4-I17     0.3258  0.0175  18.5785  <.0001  0.2915  0.3602  *** 
## 
## ---
## Signif. codes:  0 '***' 0.001 '**' 0.01 '*' 0.05 '.' 0.1 ' ' 1
```

```
## Cluster-robust standard errors
summary(
  robust(tas20.mlmvrem1.sense,
         cluster = StudyID,
         clubSandwich = TRUE)
)
```

```
## 
## Multivariate Meta-Analysis Model (k = 880; method: REML)
## 
##     logLik    Deviance         AIC         BIC        AICc   
##   953.4645  -1906.9291  -1844.9291  -1697.1058  -1842.5615   
## 
## Variance Components:
## 
## outer factor: ESID         (nlvls = 880)
## inner factor: factor(Cell) (nlvls = 10)
## 
##              estim    sqrt  k.lvl  fixed    level 
## tau^2.1     0.0011  0.0330     88     no  I11-I12 
## tau^2.2     0.0011  0.0338     88     no  I11-I17 
## tau^2.3     0.0010  0.0320     88     no  I12-I17 
## tau^2.4     0.0011  0.0326     88     no   I2-I11 
## tau^2.5     0.0014  0.0368     88     no   I2-I12 
## tau^2.6     0.0019  0.0441     88     no   I2-I17 
## tau^2.7     0.0077  0.0876     88     no    I2-I4 
## tau^2.8     0.0028  0.0525     88     no   I4-I11 
## tau^2.9     0.0033  0.0578     88     no   I4-I12 
## tau^2.10    0.0032  0.0570     88     no   I4-I17 
## rho         0.0000                   yes          
## 
## outer factor: StudyID      (nlvls = 62)
## inner factor: factor(Cell) (nlvls = 10)
## 
##                estim    sqrt  k.lvl  fixed    level 
## gamma^2.1     0.0088  0.0940     62     no  I11-I12 
## gamma^2.2     0.0079  0.0891     62     no  I11-I17 
## gamma^2.3     0.0122  0.1105     62     no  I12-I17 
## gamma^2.4     0.0060  0.0774     62     no   I2-I11 
## gamma^2.5     0.0101  0.1007     62     no   I2-I12 
## gamma^2.6     0.0090  0.0947     62     no   I2-I17 
## gamma^2.7     0.0149  0.1221     62     no    I2-I4 
## gamma^2.8     0.0116  0.1078     62     no   I4-I11 
## gamma^2.9     0.0137  0.1171     62     no   I4-I12 
## gamma^2.10    0.0149  0.1223     62     no   I4-I17 
## phi           0.9252                    no          
## 
## Test for Residual Heterogeneity:
## QE(df = 870) = 6986.5768, p-val < .0001
## 
## Number of estimates:   880
## Number of clusters:    62
## Estimates per cluster: 10-40 (mean: 14.19, median: 10)
## 
## Test of Moderators (coefficients 1:10):¹
## F(df1 = 10, df2 = 45.12) = 179.7401, p-val < .0001
## 
## Model Results:
## 
##                      estimate      se¹     tval¹     df¹    pval¹   ci.lb¹ 
## factor(Cell)I11-I12    0.3220  0.0142   22.6635   60.01   <.0001   0.2936  
## factor(Cell)I11-I17    0.3525  0.0134   26.3397   59.95   <.0001   0.3258  
## factor(Cell)I12-I17    0.2868  0.0155   18.5165   60.41   <.0001   0.2558  
## factor(Cell)I2-I11     0.4544  0.0121   37.6789   59.74   <.0001   0.4303  
## factor(Cell)I2-I12     0.3600  0.0145   24.7623   60.21   <.0001   0.3310  
## factor(Cell)I2-I17     0.3926  0.0142   27.7129   60.04   <.0001   0.3643  
## factor(Cell)I2-I4      0.4288  0.0191   22.4370   59.88   <.0001   0.3906  
## factor(Cell)I4-I11     0.3607  0.0156   23.1334    60.2   <.0001   0.3295  
## factor(Cell)I4-I12     0.2926  0.0173   16.9605   60.21   <.0001   0.2581  
## factor(Cell)I4-I17     0.3258  0.0179   18.2306   60.36   <.0001   0.2901  
##                       ci.ub¹      
## factor(Cell)I11-I12  0.3504   *** 
## factor(Cell)I11-I17  0.3793   *** 
## factor(Cell)I12-I17  0.3178   *** 
## factor(Cell)I2-I11   0.4785   *** 
## factor(Cell)I2-I12   0.3891   *** 
## factor(Cell)I2-I17   0.4210   *** 
## factor(Cell)I2-I4    0.4670   *** 
## factor(Cell)I4-I11   0.3919   *** 
## factor(Cell)I4-I12   0.3271   *** 
## factor(Cell)I4-I17   0.3616   *** 
## 
## ---
## Signif. codes:  0 '***' 0.001 '**' 0.01 '*' 0.05 '.' 0.1 ' ' 1
## 
## 1) results based on cluster-robust inference (var-cov estimator: CR2,
##    approx t/F-tests and confidence intervals, df: Satterthwaite approx)
```

### Models with some level-specific and some constrained variance estimates (`Model 2` and `Model 3`)

Model `mlmvrem2` assumes that each type of pooled
correlation has its own set of estimates of the amounts of residual
heterogeneity between effect sizes within studies (aka
`Model 2`).

#### Correlation-specific effect sizes and within-study heterogeneity, overall between-study heterogeneity (`Model 2`)

```
## Model specification
tas20.mlmvrem2 <- rma.mv(yi = Correlation,
                         V = V, 
                         data = tas20,
                         random = list(~ factor(Cell) | ESID,
                                       ~ factor(Cell) | StudyID),
                         struc = c("HCS", "CS"),
                         rho = 0,
                         phi = 0,
                         method = "REML",
                         mods = ~ factor(Cell) - 1,
                         time = TRUE,
                         sparse = TRUE,
                         control = list(optimizer = "optimParallel",
                                        ncpus = ncores))
```

```
## 
## Processing time: 0 hours, 0 minutes, 10.9 seconds
```

```
## Model summary
summary(tas20.mlmvrem2)
```

```
## 
## Multivariate Meta-Analysis Model (k = 880; method: REML)
## 
##     logLik    Deviance         AIC         BIC        AICc   
##   724.5699  -1449.1398  -1407.1398  -1307.0015  -1406.0502   
## 
## Variance Components:
## 
## outer factor: ESID         (nlvls = 880)
## inner factor: factor(Cell) (nlvls = 10)
## 
##              estim    sqrt  k.lvl  fixed    level 
## tau^2.1     0.0008  0.0291     88     no  I11-I12 
## tau^2.2     0.0006  0.0250     88     no  I11-I17 
## tau^2.3     0.0018  0.0422     88     no  I12-I17 
## tau^2.4     0.0003  0.0185     88     no   I2-I11 
## tau^2.5     0.0021  0.0453     88     no   I2-I12 
## tau^2.6     0.0023  0.0479     88     no   I2-I17 
## tau^2.7     0.0122  0.1106     88     no    I2-I4 
## tau^2.8     0.0042  0.0652     88     no   I4-I11 
## tau^2.9     0.0050  0.0705     88     no   I4-I12 
## tau^2.10    0.0061  0.0781     88     no   I4-I17 
## rho         0.0000                   yes          
## 
## outer factor: StudyID      (nlvls = 62)
## inner factor: factor(Cell) (nlvls = 10)
## 
##             estim    sqrt  fixed 
## gamma^2    0.0053  0.0731     no 
## phi        0.0000            yes 
## 
## Test for Residual Heterogeneity:
## QE(df = 870) = 6986.5768, p-val < .0001
## 
## Test of Moderators (coefficients 1:10):
## QM(df = 10) = 6560.1643, p-val < .0001
## 
## Model Results:
## 
##                      estimate      se     zval    pval   ci.lb   ci.ub      
## factor(Cell)I11-I12    0.3140  0.0112  28.1221  <.0001  0.2921  0.3359  *** 
## factor(Cell)I11-I17    0.3468  0.0110  31.6096  <.0001  0.3253  0.3683  *** 
## factor(Cell)I12-I17    0.2805  0.0117  23.9381  <.0001  0.2576  0.3035  *** 
## factor(Cell)I2-I11     0.4490  0.0106  42.2816  <.0001  0.4282  0.4698  *** 
## factor(Cell)I2-I12     0.3511  0.0118  29.8313  <.0001  0.3281  0.3742  *** 
## factor(Cell)I2-I17     0.3855  0.0118  32.5951  <.0001  0.3623  0.4086  *** 
## factor(Cell)I2-I4      0.4223  0.0160  26.3417  <.0001  0.3909  0.4537  *** 
## factor(Cell)I4-I11     0.3503  0.0129  27.2549  <.0001  0.3251  0.3755  *** 
## factor(Cell)I4-I12     0.2804  0.0133  21.1107  <.0001  0.2544  0.3064  *** 
## factor(Cell)I4-I17     0.3151  0.0137  22.9329  <.0001  0.2882  0.3420  *** 
## 
## ---
## Signif. codes:  0 '***' 0.001 '**' 0.01 '*' 0.05 '.' 0.1 ' ' 1
```

```
## Cluster-robust standard errors
tas20.mlmvrem2.robust <- robust(tas20.mlmvrem2, 
                                cluster = StudyID, 
                                clubSandwich = TRUE)

summary(tas20.mlmvrem2.robust)
```

```
## 
## Multivariate Meta-Analysis Model (k = 880; method: REML)
## 
##     logLik    Deviance         AIC         BIC        AICc   
##   724.5699  -1449.1398  -1407.1398  -1307.0015  -1406.0502   
## 
## Variance Components:
## 
## outer factor: ESID         (nlvls = 880)
## inner factor: factor(Cell) (nlvls = 10)
## 
##              estim    sqrt  k.lvl  fixed    level 
## tau^2.1     0.0008  0.0291     88     no  I11-I12 
## tau^2.2     0.0006  0.0250     88     no  I11-I17 
## tau^2.3     0.0018  0.0422     88     no  I12-I17 
## tau^2.4     0.0003  0.0185     88     no   I2-I11 
## tau^2.5     0.0021  0.0453     88     no   I2-I12 
## tau^2.6     0.0023  0.0479     88     no   I2-I17 
## tau^2.7     0.0122  0.1106     88     no    I2-I4 
## tau^2.8     0.0042  0.0652     88     no   I4-I11 
## tau^2.9     0.0050  0.0705     88     no   I4-I12 
## tau^2.10    0.0061  0.0781     88     no   I4-I17 
## rho         0.0000                   yes          
## 
## outer factor: StudyID      (nlvls = 62)
## inner factor: factor(Cell) (nlvls = 10)
## 
##             estim    sqrt  fixed 
## gamma^2    0.0053  0.0731     no 
## phi        0.0000            yes 
## 
## Test for Residual Heterogeneity:
## QE(df = 870) = 6986.5768, p-val < .0001
## 
## Number of estimates:   880
## Number of clusters:    62
## Estimates per cluster: 10-40 (mean: 14.19, median: 10)
## 
## Test of Moderators (coefficients 1:10):¹
## F(df1 = 10, df2 = 49.94) = 189.9699, p-val < .0001
## 
## Model Results:
## 
##                      estimate      se¹     tval¹     df¹    pval¹   ci.lb¹ 
## factor(Cell)I11-I12    0.3140  0.0133   23.5539   59.01   <.0001   0.2874  
## factor(Cell)I11-I17    0.3468  0.0130   26.6516   59.23   <.0001   0.3207  
## factor(Cell)I12-I17    0.2805  0.0151   18.6216   58.91   <.0001   0.2504  
## factor(Cell)I2-I11     0.4490  0.0115   39.1235   59.56   <.0001   0.4260  
## factor(Cell)I2-I12     0.3511  0.0142   24.6562   58.97   <.0001   0.3226  
## factor(Cell)I2-I17     0.3855  0.0141   27.3582   59.09   <.0001   0.3573  
## factor(Cell)I2-I4      0.4223  0.0188   22.5082   56.97   <.0001   0.3847  
## factor(Cell)I4-I11     0.3503  0.0156   22.4180   58.58   <.0001   0.3190  
## factor(Cell)I4-I12     0.2804  0.0173   16.2109   58.23   <.0001   0.2458  
## factor(Cell)I4-I17     0.3151  0.0173   18.1747   58.05   <.0001   0.2804  
##                       ci.ub¹      
## factor(Cell)I11-I12  0.3407   *** 
## factor(Cell)I11-I17  0.3728   *** 
## factor(Cell)I12-I17  0.3107   *** 
## factor(Cell)I2-I11   0.4720   *** 
## factor(Cell)I2-I12   0.3796   *** 
## factor(Cell)I2-I17   0.4137   *** 
## factor(Cell)I2-I4    0.4599   *** 
## factor(Cell)I4-I11   0.3816   *** 
## factor(Cell)I4-I12   0.3150   *** 
## factor(Cell)I4-I17   0.3498   *** 
## 
## ---
## Signif. codes:  0 '***' 0.001 '**' 0.01 '*' 0.05 '.' 0.1 ' ' 1
## 
## 1) results based on cluster-robust inference (var-cov estimator: CR2,
##    approx t/F-tests and confidence intervals, df: Satterthwaite approx)
```

```
## Processing time in seconds
tas20.mlmvrem2$time
```

```
## [1] 10.897
```

```
## Extract relevant elements
## Asymptotic covariance matrix
ACOV.mlmvrem2 <- tas20.mlmvrem2$vb

## Marginal differences between the asymptotic covariance matrices
## cluster-robust vs. not robust
max(tas20.mlmvrem2$vb-tas20.mlmvrem2.robust$vb)
```

```
## [1] -1.894066e-05
```

```
## Pooled correlation matrix
CORR.mlmvrem2 <- vec2symMat(x = c(1, tas20.mlmvrem2$b[7], tas20.mlmvrem2$b[4], tas20.mlmvrem2$b[5], tas20.mlmvrem2$b[6],
                                1, tas20.mlmvrem2$b[8], tas20.mlmvrem2$b[9], tas20.mlmvrem2$b[10],
                                1, tas20.mlmvrem2$b[1], tas20.mlmvrem2$b[2],
                                1, tas20.mlmvrem2$b[3],
                                1),
                          diag = TRUE)

colnames(CORR.mlmvrem2) <- c("Item2", "Item4", "Item11", "Item12", "Item17")
rownames(CORR.mlmvrem2) <- c("Item2", "Item4", "Item11", "Item12", "Item17")

## Inspect the pooled correlation matrix
CORR.mlmvrem2
```

```
##            Item2     Item4    Item11    Item12    Item17
## Item2  1.0000000 0.4222957 0.4490002 0.3511203 0.3854616
## Item4  0.4222957 1.0000000 0.3503105 0.2804074 0.3150984
## Item11 0.4490002 0.3503105 1.0000000 0.3140357 0.3467795
## Item12 0.3511203 0.2804074 0.3140357 1.0000000 0.2805494
## Item17 0.3854616 0.3150984 0.3467795 0.2805494 1.0000000
```

```
## Plot the pooled correlation matrix
corrplot(CORR.mlmvrem2, 
         type = "upper", 
         order = "original", 
         tl.col = "black", 
         tl.srt = 60,
         addCoef.col = "black",
         number.cex = 0.9,
         cl.cex = 1,
         tl.cex = 1)
```

```
## Extract the relevant information
mlmvrem2.results <- data.frame(round(tas20.mlmvrem2.robust$b[,1],3), 
                               round(tas20.mlmvrem2.robust$se,3),
                               round(tas20.mlmvrem2.robust$ci.lb,3),
                               round(tas20.mlmvrem2.robust$ci.ub,3),
                               round(tas20.mlmvrem2.robust$tau2,3),
                               round(tas20.mlmvrem2.robust$gamma2,3))

colnames(mlmvrem2.results) <- c("r",
                                "SE",
                                "CI95-low",
                                "CI95-high",
                                "tau2",
                                "gamma2")

## Data frame with results
mlmvrem2.results
```

```
##                         r    SE CI95-low CI95-high  tau2 gamma2
## factor(Cell)I11-I12 0.314 0.013    0.287     0.341 0.001  0.005
## factor(Cell)I11-I17 0.347 0.013    0.321     0.373 0.001  0.005
## factor(Cell)I12-I17 0.281 0.015    0.250     0.311 0.002  0.005
## factor(Cell)I2-I11  0.449 0.011    0.426     0.472 0.000  0.005
## factor(Cell)I2-I12  0.351 0.014    0.323     0.380 0.002  0.005
## factor(Cell)I2-I17  0.385 0.014    0.357     0.414 0.002  0.005
## factor(Cell)I2-I4   0.422 0.019    0.385     0.460 0.012  0.005
## factor(Cell)I4-I11  0.350 0.016    0.319     0.382 0.004  0.005
## factor(Cell)I4-I12  0.280 0.017    0.246     0.315 0.005  0.005
## factor(Cell)I4-I17  0.315 0.017    0.280     0.350 0.006  0.005
```

```
##write.csv2(mlmvrem2.results, file = "MLMVREM-Table2-mlmvrem2.csv")


### Create a new model fit data frame
newfit.mlmvrem2 <- data.frame(
  c("logLik", "parms", "k",
    "Deviance", 
    "AIC", "BIC", "AICc"),
  c(## logLik
    tas20.mlmvrem2$fit.stats$REML[1],
    ## Number of parameters (parms)
    tas20.mlmvrem2$parms,
    ## Number of effect sizes (k)
    length(tas20$Correlation),
    ## Deviance
    tas20.mlmvrem2$fit.stats$REML[2],
    ## AIC
    tas20.mlmvrem2$fit.stats$REML[3],
    ## BIC
    tas20.mlmvrem2$fit.stats$REML[4],
    ## AICc
    tas20.mlmvrem2$fit.stats$REML[5]))

colnames(newfit.mlmvrem2) <- c("Fit criterion", "mlmvrem2")
newfit.mlmvrem2
```

```
##   Fit criterion   mlmvrem2
## 1        logLik   724.5699
## 2         parms    21.0000
## 3             k   880.0000
## 4      Deviance -1449.1398
## 5           AIC -1407.1398
## 6           BIC -1307.0015
## 7          AICc -1406.0502
```

```
## Sensitivity analysis
## Random effects: rho = 0.5, phi = 0.5
## Model specification
tas20.mlmvrem2.sens <- rma.mv(yi = Correlation,
                              V = V, 
                              data = tas20,
                              random = list(~ factor(Cell) | ESID,
                                            ~ factor(Cell) | StudyID),
                              struc = c("HCS", "CS"),
                              rho = 0.5,
                              phi = 0.5,
                              method = "REML",
                              mods = ~ factor(Cell) - 1,
                              time = TRUE,
                              sparse = TRUE,
                              control = list(optimizer = "optimParallel",
                                             ncpus = ncores))
```

```
## 
## Processing time: 0 hours, 0 minutes, 44.56 seconds
```

```
## Model summary
summary(tas20.mlmvrem2.sens)
```

```
## 
## Multivariate Meta-Analysis Model (k = 880; method: REML)
## 
##     logLik    Deviance         AIC         BIC        AICc   
##   904.2915  -1808.5830  -1766.5830  -1666.4447  -1765.4934   
## 
## Variance Components:
## 
## outer factor: ESID         (nlvls = 880)
## inner factor: factor(Cell) (nlvls = 10)
## 
##              estim    sqrt  k.lvl  fixed    level 
## tau^2.1     0.0004  0.0203     88     no  I11-I12 
## tau^2.2     0.0003  0.0176     88     no  I11-I17 
## tau^2.3     0.0008  0.0276     88     no  I12-I17 
## tau^2.4     0.0002  0.0135     88     no   I2-I11 
## tau^2.5     0.0011  0.0335     88     no   I2-I12 
## tau^2.6     0.0013  0.0364     88     no   I2-I17 
## tau^2.7     0.0075  0.0865     88     no    I2-I4 
## tau^2.8     0.0020  0.0451     88     no   I4-I11 
## tau^2.9     0.0027  0.0520     88     no   I4-I12 
## tau^2.10    0.0030  0.0544     88     no   I4-I17 
## rho         0.5000                   yes          
## 
## outer factor: StudyID      (nlvls = 62)
## inner factor: factor(Cell) (nlvls = 10)
## 
##             estim    sqrt  fixed 
## gamma^2    0.0056  0.0748     no 
## phi        0.5000            yes 
## 
## Test for Residual Heterogeneity:
## QE(df = 870) = 6986.5768, p-val < .0001
## 
## Test of Moderators (coefficients 1:10):
## QM(df = 10) = 2430.0650, p-val < .0001
## 
## Model Results:
## 
##                      estimate      se     zval    pval   ci.lb   ci.ub      
## factor(Cell)I11-I12    0.3189  0.0111  28.7337  <.0001  0.2972  0.3407  *** 
## factor(Cell)I11-I17    0.3505  0.0110  31.9461  <.0001  0.3290  0.3720  *** 
## factor(Cell)I12-I17    0.2845  0.0113  25.1053  <.0001  0.2623  0.3067  *** 
## factor(Cell)I2-I11     0.4523  0.0107  42.2162  <.0001  0.4313  0.4733  *** 
## factor(Cell)I2-I12     0.3558  0.0114  31.1125  <.0001  0.3334  0.3782  *** 
## factor(Cell)I2-I17     0.3890  0.0115  33.8922  <.0001  0.3665  0.4115  *** 
## factor(Cell)I2-I4      0.4270  0.0143  29.9338  <.0001  0.3991  0.4550  *** 
## factor(Cell)I4-I11     0.3573  0.0119  30.0127  <.0001  0.3339  0.3806  *** 
## factor(Cell)I4-I12     0.2877  0.0123  23.3061  <.0001  0.2635  0.3119  *** 
## factor(Cell)I4-I17     0.3220  0.0124  25.9243  <.0001  0.2976  0.3463  *** 
## 
## ---
## Signif. codes:  0 '***' 0.001 '**' 0.01 '*' 0.05 '.' 0.1 ' ' 1
```

```
## Cluster-robust standard errors
summary(
  robust(tas20.mlmvrem2.sens,
         cluster = StudyID,
         clubSandwich = TRUE)
)
```

```
## 
## Multivariate Meta-Analysis Model (k = 880; method: REML)
## 
##     logLik    Deviance         AIC         BIC        AICc   
##   904.2915  -1808.5830  -1766.5830  -1666.4447  -1765.4934   
## 
## Variance Components:
## 
## outer factor: ESID         (nlvls = 880)
## inner factor: factor(Cell) (nlvls = 10)
## 
##              estim    sqrt  k.lvl  fixed    level 
## tau^2.1     0.0004  0.0203     88     no  I11-I12 
## tau^2.2     0.0003  0.0176     88     no  I11-I17 
## tau^2.3     0.0008  0.0276     88     no  I12-I17 
## tau^2.4     0.0002  0.0135     88     no   I2-I11 
## tau^2.5     0.0011  0.0335     88     no   I2-I12 
## tau^2.6     0.0013  0.0364     88     no   I2-I17 
## tau^2.7     0.0075  0.0865     88     no    I2-I4 
## tau^2.8     0.0020  0.0451     88     no   I4-I11 
## tau^2.9     0.0027  0.0520     88     no   I4-I12 
## tau^2.10    0.0030  0.0544     88     no   I4-I17 
## rho         0.5000                   yes          
## 
## outer factor: StudyID      (nlvls = 62)
## inner factor: factor(Cell) (nlvls = 10)
## 
##             estim    sqrt  fixed 
## gamma^2    0.0056  0.0748     no 
## phi        0.5000            yes 
## 
## Test for Residual Heterogeneity:
## QE(df = 870) = 6986.5768, p-val < .0001
## 
## Number of estimates:   880
## Number of clusters:    62
## Estimates per cluster: 10-40 (mean: 14.19, median: 10)
## 
## Test of Moderators (coefficients 1:10):¹
## F(df1 = 10, df2 = 49.24) = 185.0655, p-val < .0001
## 
## Model Results:
## 
##                      estimate      se¹     tval¹     df¹    pval¹   ci.lb¹ 
## factor(Cell)I11-I12    0.3189  0.0137   23.3390    59.1   <.0001   0.2916  
## factor(Cell)I11-I17    0.3505  0.0134   26.2036   59.33   <.0001   0.3238  
## factor(Cell)I12-I17    0.2845  0.0154   18.4321   59.11   <.0001   0.2536  
## factor(Cell)I2-I11     0.4523  0.0118   38.3051   59.64   <.0001   0.4287  
## factor(Cell)I2-I12     0.3558  0.0144   24.7759   59.16   <.0001   0.3271  
## factor(Cell)I2-I17     0.3890  0.0142   27.4857   59.32   <.0001   0.3607  
## factor(Cell)I2-I4      0.4270  0.0191   22.4048   57.95   <.0001   0.3889  
## factor(Cell)I4-I11     0.3573  0.0157   22.6949    59.1   <.0001   0.3258  
## factor(Cell)I4-I12     0.2877  0.0174   16.5230   58.72   <.0001   0.2529  
## factor(Cell)I4-I17     0.3220  0.0175   18.3495   58.77   <.0001   0.2869  
##                       ci.ub¹      
## factor(Cell)I11-I12  0.3463   *** 
## factor(Cell)I11-I17  0.3773   *** 
## factor(Cell)I12-I17  0.3154   *** 
## factor(Cell)I2-I11   0.4759   *** 
## factor(Cell)I2-I12   0.3846   *** 
## factor(Cell)I2-I17   0.4173   *** 
## factor(Cell)I2-I4    0.4652   *** 
## factor(Cell)I4-I11   0.3888   *** 
## factor(Cell)I4-I12   0.3226   *** 
## factor(Cell)I4-I17   0.3571   *** 
## 
## ---
## Signif. codes:  0 '***' 0.001 '**' 0.01 '*' 0.05 '.' 0.1 ' ' 1
## 
## 1) results based on cluster-robust inference (var-cov estimator: CR2,
##    approx t/F-tests and confidence intervals, df: Satterthwaite approx)
```

```
## Sensitivity analysis
## Random effects: rho = 1, phi = 1
## Model specification
tas20.mlmvrem2.sent <- rma.mv(yi = Correlation,
                              V = V, 
                              data = tas20,
                              random = list(~ factor(Cell) | ESID,
                                            ~ factor(Cell) | StudyID),
                              struc = c("HCS", "CS"),
                              rho = 1,
                              phi = 1,
                              method = "REML",
                              mods = ~ factor(Cell) - 1,
                              time = TRUE,
                              sparse = TRUE,
                              control = list(optimizer = "optimParallel",
                                             ncpus = ncores))
```

```
## 
## Processing time: 0 hours, 0 minutes, 19.93 seconds
```

```
## Model summary
summary(tas20.mlmvrem2.sent)
```

```
## 
## Multivariate Meta-Analysis Model (k = 880; method: REML)
## 
##     logLik    Deviance         AIC         BIC        AICc   
##   938.0735  -1876.1470  -1834.1470  -1734.0087  -1833.0574   
## 
## Variance Components:
## 
## outer factor: ESID         (nlvls = 880)
## inner factor: factor(Cell) (nlvls = 10)
## 
##              estim    sqrt  k.lvl  fixed    level 
## tau^2.1     0.0017  0.0406     88     no  I11-I12 
## tau^2.2     0.0015  0.0392     88     no  I11-I17 
## tau^2.3     0.0017  0.0411     88     no  I12-I17 
## tau^2.4     0.0018  0.0429     88     no   I2-I11 
## tau^2.5     0.0018  0.0419     88     no   I2-I12 
## tau^2.6     0.0026  0.0509     88     no   I2-I17 
## tau^2.7     0.0101  0.1006     88     no    I2-I4 
## tau^2.8     0.0042  0.0646     88     no   I4-I11 
## tau^2.9     0.0052  0.0721     88     no   I4-I12 
## tau^2.10    0.0055  0.0741     88     no   I4-I17 
## rho         1.0000                   yes          
## 
## outer factor: StudyID      (nlvls = 62)
## inner factor: factor(Cell) (nlvls = 10)
## 
##             estim    sqrt  fixed 
## gamma^2    0.0091  0.0954     no 
## phi        1.0000            yes 
## 
## Test for Residual Heterogeneity:
## QE(df = 870) = 6986.5768, p-val < .0001
## 
## Test of Moderators (coefficients 1:10):
## QM(df = 10) = 1332.5104, p-val < .0001
## 
## Model Results:
## 
##                      estimate      se     zval    pval   ci.lb   ci.ub      
## factor(Cell)I11-I12    0.3215  0.0138  23.2608  <.0001  0.2944  0.3486  *** 
## factor(Cell)I11-I17    0.3514  0.0137  25.6098  <.0001  0.3246  0.3783  *** 
## factor(Cell)I12-I17    0.2868  0.0139  20.6969  <.0001  0.2597  0.3140  *** 
## factor(Cell)I2-I11     0.4517  0.0137  32.8772  <.0001  0.4248  0.4787  *** 
## factor(Cell)I2-I12     0.3603  0.0138  26.0706  <.0001  0.3332  0.3874  *** 
## factor(Cell)I2-I17     0.3926  0.0141  27.7808  <.0001  0.3649  0.4203  *** 
## factor(Cell)I2-I4      0.4295  0.0169  25.4387  <.0001  0.3964  0.4626  *** 
## factor(Cell)I4-I11     0.3618  0.0148  24.4236  <.0001  0.3327  0.3908  *** 
## factor(Cell)I4-I12     0.2939  0.0153  19.2147  <.0001  0.2639  0.3238  *** 
## factor(Cell)I4-I17     0.3277  0.0154  21.3296  <.0001  0.2976  0.3578  *** 
## 
## ---
## Signif. codes:  0 '***' 0.001 '**' 0.01 '*' 0.05 '.' 0.1 ' ' 1
```

```
## Cluster-robust standard errors
summary(
  robust(tas20.mlmvrem2.sent,
         cluster = StudyID,
         clubSandwich = TRUE)
)
```

```
## 
## Multivariate Meta-Analysis Model (k = 880; method: REML)
## 
##     logLik    Deviance         AIC         BIC        AICc   
##   938.0735  -1876.1470  -1834.1470  -1734.0087  -1833.0574   
## 
## Variance Components:
## 
## outer factor: ESID         (nlvls = 880)
## inner factor: factor(Cell) (nlvls = 10)
## 
##              estim    sqrt  k.lvl  fixed    level 
## tau^2.1     0.0017  0.0406     88     no  I11-I12 
## tau^2.2     0.0015  0.0392     88     no  I11-I17 
## tau^2.3     0.0017  0.0411     88     no  I12-I17 
## tau^2.4     0.0018  0.0429     88     no   I2-I11 
## tau^2.5     0.0018  0.0419     88     no   I2-I12 
## tau^2.6     0.0026  0.0509     88     no   I2-I17 
## tau^2.7     0.0101  0.1006     88     no    I2-I4 
## tau^2.8     0.0042  0.0646     88     no   I4-I11 
## tau^2.9     0.0052  0.0721     88     no   I4-I12 
## tau^2.10    0.0055  0.0741     88     no   I4-I17 
## rho         1.0000                   yes          
## 
## outer factor: StudyID      (nlvls = 62)
## inner factor: factor(Cell) (nlvls = 10)
## 
##             estim    sqrt  fixed 
## gamma^2    0.0091  0.0954     no 
## phi        1.0000            yes 
## 
## Test for Residual Heterogeneity:
## QE(df = 870) = 6986.5768, p-val < .0001
## 
## Number of estimates:   880
## Number of clusters:    62
## Estimates per cluster: 10-40 (mean: 14.19, median: 10)
## 
## Test of Moderators (coefficients 1:10):¹
## F(df1 = 10, df2 = 40.96) = 162.6361, p-val < .0001
## 
## Model Results:
## 
##                      estimate      se¹     tval¹     df¹    pval¹   ci.lb¹ 
## factor(Cell)I11-I12    0.3215  0.0143   22.5505   59.94   <.0001   0.2930  
## factor(Cell)I11-I17    0.3514  0.0133   26.4391   60.06   <.0001   0.3249  
## factor(Cell)I12-I17    0.2868  0.0150   19.0651   59.92   <.0001   0.2567  
## factor(Cell)I2-I11     0.4517  0.0124   36.3883   60.19   <.0001   0.4269  
## factor(Cell)I2-I12     0.3603  0.0145   24.8778   59.99   <.0001   0.3313  
## factor(Cell)I2-I17     0.3926  0.0143   27.5257    59.9   <.0001   0.3641  
## factor(Cell)I2-I4      0.4295  0.0186   23.0502   58.22   <.0001   0.3922  
## factor(Cell)I4-I11     0.3618  0.0153   23.5757   59.46   <.0001   0.3311  
## factor(Cell)I4-I12     0.2939  0.0170   17.2845   59.06   <.0001   0.2598  
## factor(Cell)I4-I17     0.3277  0.0183   17.9250   59.04   <.0001   0.2911  
##                       ci.ub¹      
## factor(Cell)I11-I12  0.3500   *** 
## factor(Cell)I11-I17  0.3780   *** 
## factor(Cell)I12-I17  0.3169   *** 
## factor(Cell)I2-I11   0.4766   *** 
## factor(Cell)I2-I12   0.3893   *** 
## factor(Cell)I2-I17   0.4212   *** 
## factor(Cell)I2-I4    0.4668   *** 
## factor(Cell)I4-I11   0.3925   *** 
## factor(Cell)I4-I12   0.3279   *** 
## factor(Cell)I4-I17   0.3643   *** 
## 
## ---
## Signif. codes:  0 '***' 0.001 '**' 0.01 '*' 0.05 '.' 0.1 ' ' 1
## 
## 1) results based on cluster-robust inference (var-cov estimator: CR2,
##    approx t/F-tests and confidence intervals, df: Satterthwaite approx)
```

```
## Sensitivity analysis
## Random effects: rho = 0, phi estimated
## Model specification
tas20.mlmvrem2.senu <- rma.mv(yi = Correlation,
                              V = V, 
                              data = tas20,
                              random = list(~ factor(Cell) | ESID,
                                            ~ factor(Cell) | StudyID),
                              struc = c("HCS", "CS"),
                              rho = 0,
                              phi = NA,
                              method = "REML",
                              mods = ~ factor(Cell) - 1,
                              time = TRUE,
                              sparse = TRUE,
                              control = list(optimizer = "optimParallel",
                                             ncpus = ncores))
```

```
## 
## Processing time: 0 hours, 0 minutes, 29.75 seconds
```

```
## Model summary
summary(tas20.mlmvrem2.senu)
```

```
## 
## Multivariate Meta-Analysis Model (k = 880; method: REML)
## 
##     logLik    Deviance         AIC         BIC        AICc   
##   942.3666  -1884.7332  -1840.7332  -1735.8263  -1839.5384   
## 
## Variance Components:
## 
## outer factor: ESID         (nlvls = 880)
## inner factor: factor(Cell) (nlvls = 10)
## 
##              estim    sqrt  k.lvl  fixed    level 
## tau^2.1     0.0008  0.0284     88     no  I11-I12 
## tau^2.2     0.0008  0.0281     88     no  I11-I17 
## tau^2.3     0.0012  0.0342     88     no  I12-I17 
## tau^2.4     0.0007  0.0273     88     no   I2-I11 
## tau^2.5     0.0013  0.0366     88     no   I2-I12 
## tau^2.6     0.0018  0.0422     88     no   I2-I17 
## tau^2.7     0.0089  0.0945     88     no    I2-I4 
## tau^2.8     0.0031  0.0557     88     no   I4-I11 
## tau^2.9     0.0042  0.0648     88     no   I4-I12 
## tau^2.10    0.0041  0.0641     88     no   I4-I17 
## rho         0.0000                   yes          
## 
## outer factor: StudyID      (nlvls = 62)
## inner factor: factor(Cell) (nlvls = 10)
## 
##             estim    sqrt  fixed 
## gamma^2    0.0099  0.0995     no 
## phi        0.9144             no 
## 
## Test for Residual Heterogeneity:
## QE(df = 870) = 6986.5768, p-val < .0001
## 
## Test of Moderators (coefficients 1:10):
## QM(df = 10) = 1298.6732, p-val < .0001
## 
## Model Results:
## 
##                      estimate      se     zval    pval   ci.lb   ci.ub      
## factor(Cell)I11-I12    0.3219  0.0140  23.0006  <.0001  0.2945  0.3493  *** 
## factor(Cell)I11-I17    0.3522  0.0139  25.2657  <.0001  0.3249  0.3795  *** 
## factor(Cell)I12-I17    0.2867  0.0142  20.2202  <.0001  0.2589  0.3145  *** 
## factor(Cell)I2-I11     0.4538  0.0138  32.9060  <.0001  0.4268  0.4809  *** 
## factor(Cell)I2-I12     0.3597  0.0142  25.3566  <.0001  0.3319  0.3875  *** 
## factor(Cell)I2-I17     0.3920  0.0143  27.3667  <.0001  0.3639  0.4200  *** 
## factor(Cell)I2-I4      0.4293  0.0169  25.3444  <.0001  0.3961  0.4625  *** 
## factor(Cell)I4-I11     0.3610  0.0149  24.2273  <.0001  0.3318  0.3902  *** 
## factor(Cell)I4-I12     0.2925  0.0154  18.9793  <.0001  0.2623  0.3227  *** 
## factor(Cell)I4-I17     0.3263  0.0153  21.2838  <.0001  0.2963  0.3564  *** 
## 
## ---
## Signif. codes:  0 '***' 0.001 '**' 0.01 '*' 0.05 '.' 0.1 ' ' 1
```

```
## Cluster-robust standard errors
summary(
  robust(tas20.mlmvrem2.senu,
         cluster = StudyID,
         clubSandwich = TRUE)
)
```

```
## 
## Multivariate Meta-Analysis Model (k = 880; method: REML)
## 
##     logLik    Deviance         AIC         BIC        AICc   
##   942.3666  -1884.7332  -1840.7332  -1735.8263  -1839.5384   
## 
## Variance Components:
## 
## outer factor: ESID         (nlvls = 880)
## inner factor: factor(Cell) (nlvls = 10)
## 
##              estim    sqrt  k.lvl  fixed    level 
## tau^2.1     0.0008  0.0284     88     no  I11-I12 
## tau^2.2     0.0008  0.0281     88     no  I11-I17 
## tau^2.3     0.0012  0.0342     88     no  I12-I17 
## tau^2.4     0.0007  0.0273     88     no   I2-I11 
## tau^2.5     0.0013  0.0366     88     no   I2-I12 
## tau^2.6     0.0018  0.0422     88     no   I2-I17 
## tau^2.7     0.0089  0.0945     88     no    I2-I4 
## tau^2.8     0.0031  0.0557     88     no   I4-I11 
## tau^2.9     0.0042  0.0648     88     no   I4-I12 
## tau^2.10    0.0041  0.0641     88     no   I4-I17 
## rho         0.0000                   yes          
## 
## outer factor: StudyID      (nlvls = 62)
## inner factor: factor(Cell) (nlvls = 10)
## 
##             estim    sqrt  fixed 
## gamma^2    0.0099  0.0995     no 
## phi        0.9144             no 
## 
## Test for Residual Heterogeneity:
## QE(df = 870) = 6986.5768, p-val < .0001
## 
## Number of estimates:   880
## Number of clusters:    62
## Estimates per cluster: 10-40 (mean: 14.19, median: 10)
## 
## Test of Moderators (coefficients 1:10):¹
## F(df1 = 10, df2 = 45.49) = 175.7490, p-val < .0001
## 
## Model Results:
## 
##                      estimate      se¹     tval¹     df¹    pval¹   ci.lb¹ 
## factor(Cell)I11-I12    0.3219  0.0140   22.9563    60.2   <.0001   0.2939  
## factor(Cell)I11-I17    0.3522  0.0134   26.2714   60.29   <.0001   0.3254  
## factor(Cell)I12-I17    0.2867  0.0153   18.7011   60.15   <.0001   0.2560  
## factor(Cell)I2-I11     0.4538  0.0121   37.4958   60.45   <.0001   0.4296  
## factor(Cell)I2-I12     0.3597  0.0145   24.8760   60.18   <.0001   0.3308  
## factor(Cell)I2-I17     0.3920  0.0142   27.6479    60.2   <.0001   0.3636  
## factor(Cell)I2-I4      0.4293  0.0188   22.8538   58.82   <.0001   0.3917  
## factor(Cell)I4-I11     0.3610  0.0155   23.3415   59.91   <.0001   0.3301  
## factor(Cell)I4-I12     0.2925  0.0171   17.0853   59.56   <.0001   0.2582  
## factor(Cell)I4-I17     0.3263  0.0178   18.3042   59.63   <.0001   0.2906  
##                       ci.ub¹      
## factor(Cell)I11-I12  0.3499   *** 
## factor(Cell)I11-I17  0.3790   *** 
## factor(Cell)I12-I17  0.3173   *** 
## factor(Cell)I2-I11   0.4780   *** 
## factor(Cell)I2-I12   0.3886   *** 
## factor(Cell)I2-I17   0.4203   *** 
## factor(Cell)I2-I4    0.4669   *** 
## factor(Cell)I4-I11   0.3919   *** 
## factor(Cell)I4-I12   0.3267   *** 
## factor(Cell)I4-I17   0.3620   *** 
## 
## ---
## Signif. codes:  0 '***' 0.001 '**' 0.01 '*' 0.05 '.' 0.1 ' ' 1
## 
## 1) results based on cluster-robust inference (var-cov estimator: CR2,
##    approx t/F-tests and confidence intervals, df: Satterthwaite approx)
```

#### Correlation-specific effect sizes and between-study heterogeneity, overall within-study heterogeneity (`Model 3`)

Model `mlmvrem3` specifies study-specific random-effects
per correlation coefficient and assumes only one variance estimate at
the level of effect sizes (aka `Model 3`).

```
## Random effects: rho = 0, phi = 0
## Model specification
tas20.mlmvrem3 <- rma.mv(yi = Correlation,
                         V = V, 
                         data = tas20,
                         random = list(~ factor(Cell) | ESID,
                                       ~ factor(Cell) | StudyID),
                         struc = c("CS", "HCS"),
                         rho = 0,
                         phi = 0,
                         method = "REML",
                         mods = ~ factor(Cell) - 1,
                         time = TRUE,
                         sparse = TRUE)
```

```
## 
## Processing time: 0 hours, 0 minutes, 13.14 seconds
```

```
## Model summary
summary(tas20.mlmvrem3)
```

```
## 
## Multivariate Meta-Analysis Model (k = 880; method: REML)
## 
##     logLik    Deviance         AIC         BIC        AICc   
##   710.9364  -1421.8728  -1379.8728  -1279.7344  -1378.7831   
## 
## Variance Components:
## 
## outer factor: ESID         (nlvls = 880)
## inner factor: factor(Cell) (nlvls = 10)
## 
##             estim    sqrt  fixed 
## tau^2      0.0025  0.0500     no 
## rho        0.0000            yes 
## 
## outer factor: StudyID      (nlvls = 62)
## inner factor: factor(Cell) (nlvls = 10)
## 
##                estim    sqrt  k.lvl  fixed    level 
## gamma^2.1     0.0037  0.0612     62     no  I11-I12 
## gamma^2.2     0.0035  0.0590     62     no  I11-I17 
## gamma^2.3     0.0056  0.0751     62     no  I12-I17 
## gamma^2.4     0.0026  0.0514     62     no   I2-I11 
## gamma^2.5     0.0047  0.0683     62     no   I2-I12 
## gamma^2.6     0.0050  0.0707     62     no   I2-I17 
## gamma^2.7     0.0164  0.1280     62     no    I2-I4 
## gamma^2.8     0.0080  0.0895     62     no   I4-I11 
## gamma^2.9     0.0103  0.1013     62     no   I4-I12 
## gamma^2.10    0.0102  0.1009     62     no   I4-I17 
## phi           0.0000                   yes          
## 
## Test for Residual Heterogeneity:
## QE(df = 870) = 6986.5768, p-val < .0001
## 
## Test of Moderators (coefficients 1:10):
## QM(df = 10) = 6639.0568, p-val < .0001
## 
## Model Results:
## 
##                      estimate      se     zval    pval   ci.lb   ci.ub      
## factor(Cell)I11-I12    0.3139  0.0109  28.8270  <.0001  0.2926  0.3353  *** 
## factor(Cell)I11-I17    0.3467  0.0106  32.6641  <.0001  0.3259  0.3675  *** 
## factor(Cell)I12-I17    0.2806  0.0123  22.8208  <.0001  0.2565  0.3047  *** 
## factor(Cell)I2-I11     0.4483  0.0097  46.0978  <.0001  0.4292  0.4673  *** 
## factor(Cell)I2-I12     0.3514  0.0115  30.5012  <.0001  0.3288  0.3740  *** 
## factor(Cell)I2-I17     0.3855  0.0117  32.9958  <.0001  0.3626  0.4084  *** 
## factor(Cell)I2-I4      0.4228  0.0179  23.6183  <.0001  0.3878  0.4579  *** 
## factor(Cell)I4-I11     0.3503  0.0137  25.5766  <.0001  0.3235  0.3772  *** 
## factor(Cell)I4-I12     0.2798  0.0151  18.5589  <.0001  0.2502  0.3093  *** 
## factor(Cell)I4-I17     0.3144  0.0150  20.9744  <.0001  0.2850  0.3438  *** 
## 
## ---
## Signif. codes:  0 '***' 0.001 '**' 0.01 '*' 0.05 '.' 0.1 ' ' 1
```

```
## Cluster-robust standard errors
tas20.mlmvrem3.robust <- robust(tas20.mlmvrem3, 
                                cluster = StudyID, 
                                clubSandwich = TRUE)

summary(tas20.mlmvrem3.robust)
```

```
## 
## Multivariate Meta-Analysis Model (k = 880; method: REML)
## 
##     logLik    Deviance         AIC         BIC        AICc   
##   710.9364  -1421.8728  -1379.8728  -1279.7344  -1378.7831   
## 
## Variance Components:
## 
## outer factor: ESID         (nlvls = 880)
## inner factor: factor(Cell) (nlvls = 10)
## 
##             estim    sqrt  fixed 
## tau^2      0.0025  0.0500     no 
## rho        0.0000            yes 
## 
## outer factor: StudyID      (nlvls = 62)
## inner factor: factor(Cell) (nlvls = 10)
## 
##                estim    sqrt  k.lvl  fixed    level 
## gamma^2.1     0.0037  0.0612     62     no  I11-I12 
## gamma^2.2     0.0035  0.0590     62     no  I11-I17 
## gamma^2.3     0.0056  0.0751     62     no  I12-I17 
## gamma^2.4     0.0026  0.0514     62     no   I2-I11 
## gamma^2.5     0.0047  0.0683     62     no   I2-I12 
## gamma^2.6     0.0050  0.0707     62     no   I2-I17 
## gamma^2.7     0.0164  0.1280     62     no    I2-I4 
## gamma^2.8     0.0080  0.0895     62     no   I4-I11 
## gamma^2.9     0.0103  0.1013     62     no   I4-I12 
## gamma^2.10    0.0102  0.1009     62     no   I4-I17 
## phi           0.0000                   yes          
## 
## Test for Residual Heterogeneity:
## QE(df = 870) = 6986.5768, p-val < .0001
## 
## Number of estimates:   880
## Number of clusters:    62
## Estimates per cluster: 10-40 (mean: 14.19, median: 10)
## 
## Test of Moderators (coefficients 1:10):¹
## F(df1 = 10, df2 = 50.21) = 171.8088, p-val < .0001
## 
## Model Results:
## 
##                      estimate      se¹     tval¹     df¹    pval¹   ci.lb¹ 
## factor(Cell)I11-I12    0.3139  0.0134   23.3905   57.76   <.0001   0.2871  
## factor(Cell)I11-I17    0.3467  0.0130   26.7337   57.76   <.0001   0.3207  
## factor(Cell)I12-I17    0.2806  0.0150   18.6861   58.93   <.0001   0.2506  
## factor(Cell)I2-I11     0.4483  0.0117   38.4236   57.28   <.0001   0.4249  
## factor(Cell)I2-I12     0.3514  0.0142   24.7087   58.53   <.0001   0.3229  
## factor(Cell)I2-I17     0.3855  0.0141   27.3651   58.89   <.0001   0.3573  
## factor(Cell)I2-I4      0.4228  0.0200   21.1592   60.64   <.0001   0.3829  
## factor(Cell)I4-I11     0.3503  0.0160   21.9376   59.79   <.0001   0.3184  
## factor(Cell)I4-I12     0.2798  0.0176   15.8649   60.03   <.0001   0.2445  
## factor(Cell)I4-I17     0.3144  0.0176   17.9060   60.08   <.0001   0.2793  
##                       ci.ub¹      
## factor(Cell)I11-I12  0.3408   *** 
## factor(Cell)I11-I17  0.3726   *** 
## factor(Cell)I12-I17  0.3107   *** 
## factor(Cell)I2-I11   0.4716   *** 
## factor(Cell)I2-I12   0.3798   *** 
## factor(Cell)I2-I17   0.4137   *** 
## factor(Cell)I2-I4    0.4628   *** 
## factor(Cell)I4-I11   0.3823   *** 
## factor(Cell)I4-I12   0.3150   *** 
## factor(Cell)I4-I17   0.3495   *** 
## 
## ---
## Signif. codes:  0 '***' 0.001 '**' 0.01 '*' 0.05 '.' 0.1 ' ' 1
## 
## 1) results based on cluster-robust inference (var-cov estimator: CR2,
##    approx t/F-tests and confidence intervals, df: Satterthwaite approx)
```

```
## Processing time in seconds
tas20.mlmvrem3$time
```

```
## [1] 13.137
```

```
## Extract relevant elements
## Asymptotic covariance matrix
ACOV.mlmvrem3 <- tas20.mlmvrem3$vb

## Marginal differences between the asymptotic covariance matrices
## cluster-robust vs. not robust
max(tas20.mlmvrem3$vb-tas20.mlmvrem3.robust$vb)
```

```
## [1] -4.154529e-05
```

```
## Pooled correlation matrix
CORR.mlmvrem3 <- vec2symMat(x = c(1, tas20.mlmvrem3$b[7], tas20.mlmvrem3$b[4], tas20.mlmvrem3$b[5], tas20.mlmvrem3$b[6],
                                1, tas20.mlmvrem3$b[8], tas20.mlmvrem3$b[9], tas20.mlmvrem3$b[10],
                                1, tas20.mlmvrem3$b[1], tas20.mlmvrem3$b[2],
                                1, tas20.mlmvrem3$b[3],
                                1),
                          diag = TRUE)

colnames(CORR.mlmvrem3) <- c("Item2", "Item4", "Item11", "Item12", "Item17")
rownames(CORR.mlmvrem3) <- c("Item2", "Item4", "Item11", "Item12", "Item17")

## Inspect the pooled correlation matrix
CORR.mlmvrem3
```

```
##            Item2     Item4    Item11    Item12    Item17
## Item2  1.0000000 0.4228450 0.4482698 0.3513735 0.3855322
## Item4  0.4228450 1.0000000 0.3503236 0.2797589 0.3144012
## Item11 0.4482698 0.3503236 1.0000000 0.3139224 0.3466569
## Item12 0.3513735 0.2797589 0.3139224 1.0000000 0.2806056
## Item17 0.3855322 0.3144012 0.3466569 0.2806056 1.0000000
```

```
## Plot the pooled correlation matrix
corrplot(CORR.mlmvrem3, 
         type = "upper", 
         order = "original", 
         tl.col = "black", 
         tl.srt = 60,
         addCoef.col = "black",
         number.cex = 0.9,
         cl.cex = 1,
         tl.cex = 1)
```

```
## Extract the relevant information
mlmvrem3.results <- data.frame(round(tas20.mlmvrem3.robust$b[,1],3), 
                               round(tas20.mlmvrem3.robust$se,3),
                               round(tas20.mlmvrem3.robust$ci.lb,3),
                               round(tas20.mlmvrem3.robust$ci.ub,3),
                               round(tas20.mlmvrem3.robust$tau2,3),
                               round(tas20.mlmvrem3.robust$gamma2,3))

colnames(mlmvrem3.results) <- c("r",
                                "SE",
                                "CI95-low",
                                "CI95-high",
                                "tau2",
                                "gamma2")

## Data frame with results
mlmvrem3.results
```

```
##                         r    SE CI95-low CI95-high  tau2 gamma2
## factor(Cell)I11-I12 0.314 0.013    0.287     0.341 0.002  0.004
## factor(Cell)I11-I17 0.347 0.013    0.321     0.373 0.002  0.003
## factor(Cell)I12-I17 0.281 0.015    0.251     0.311 0.002  0.006
## factor(Cell)I2-I11  0.448 0.012    0.425     0.472 0.002  0.003
## factor(Cell)I2-I12  0.351 0.014    0.323     0.380 0.002  0.005
## factor(Cell)I2-I17  0.386 0.014    0.357     0.414 0.002  0.005
## factor(Cell)I2-I4   0.423 0.020    0.383     0.463 0.002  0.016
## factor(Cell)I4-I11  0.350 0.016    0.318     0.382 0.002  0.008
## factor(Cell)I4-I12  0.280 0.018    0.244     0.315 0.002  0.010
## factor(Cell)I4-I17  0.314 0.018    0.279     0.350 0.002  0.010
```

```
##write.csv2(mlmvrem3.results, file = "MLMVREM-Table2-mlmvrem3.csv")


### Create a new model fit data frame
newfit.mlmvrem3 <- data.frame(
  c("logLik", "parms", "k",
    "Deviance", 
    "AIC", "BIC", "AICc"),
  c(## logLik
    tas20.mlmvrem3$fit.stats$REML[1],
    ## Number of parameters (parms)
    tas20.mlmvrem3$parms,
    ## Number of effect sizes (k)
    length(tas20$Correlation),
    ## Deviance
    tas20.mlmvrem3$fit.stats$REML[2],
    ## AIC
    tas20.mlmvrem3$fit.stats$REML[3],
    ## BIC
    tas20.mlmvrem3$fit.stats$REML[4],
    ## AICc
    tas20.mlmvrem3$fit.stats$REML[5]))

colnames(newfit.mlmvrem3) <- c("Fit criterion", "mlmvrem3")
newfit.mlmvrem3
```

```
##   Fit criterion   mlmvrem3
## 1        logLik   710.9364
## 2         parms    21.0000
## 3             k   880.0000
## 4      Deviance -1421.8728
## 5           AIC -1379.8728
## 6           BIC -1279.7344
## 7          AICc -1378.7831
```

```
## Sensitivity analysis
## Random effects: rho = 0.5, phi = 0.5
## Model specification
tas20.mlmvrem3.sens <- rma.mv(yi = Correlation,
                              V = V, 
                              data = tas20,
                              random = list(~ factor(Cell) | ESID,
                                            ~ factor(Cell) | StudyID),
                              struc = c("CS", "HCS"),
                              rho = 0.5,
                              phi = 0.5,
                              method = "REML",
                              mods = ~ factor(Cell) - 1,
                              time = TRUE,
                              sparse = TRUE)
```

```
## 
## Processing time: 0 hours, 0 minutes, 50.81 seconds
```

```
## Model summary
summary(tas20.mlmvrem3.sens)
```

```
## 
## Multivariate Meta-Analysis Model (k = 880; method: REML)
## 
##     logLik    Deviance         AIC         BIC        AICc   
##   894.8170  -1789.6341  -1747.6341  -1647.4957  -1746.5445   
## 
## Variance Components:
## 
## outer factor: ESID         (nlvls = 880)
## inner factor: factor(Cell) (nlvls = 10)
## 
##             estim    sqrt  fixed 
## tau^2      0.0015  0.0390     no 
## rho        0.5000            yes 
## 
## outer factor: StudyID      (nlvls = 62)
## inner factor: factor(Cell) (nlvls = 10)
## 
##                estim    sqrt  k.lvl  fixed    level 
## gamma^2.1     0.0042  0.0647     62     no  I11-I12 
## gamma^2.2     0.0039  0.0625     62     no  I11-I17 
## gamma^2.3     0.0057  0.0757     62     no  I12-I17 
## gamma^2.4     0.0031  0.0554     62     no   I2-I11 
## gamma^2.5     0.0048  0.0696     62     no   I2-I12 
## gamma^2.6     0.0048  0.0690     62     no   I2-I17 
## gamma^2.7     0.0120  0.1097     62     no    I2-I4 
## gamma^2.8     0.0068  0.0824     62     no   I4-I11 
## gamma^2.9     0.0083  0.0914     62     no   I4-I12 
## gamma^2.10    0.0083  0.0914     62     no   I4-I17 
## phi           0.5000                   yes          
## 
## Test for Residual Heterogeneity:
## QE(df = 870) = 6986.5768, p-val < .0001
## 
## Test of Moderators (coefficients 1:10):
## QM(df = 10) = 3000.8733, p-val < .0001
## 
## Model Results:
## 
##                      estimate      se     zval    pval   ci.lb   ci.ub      
## factor(Cell)I11-I12    0.3185  0.0107  29.9037  <.0001  0.2977  0.3394  *** 
## factor(Cell)I11-I17    0.3504  0.0104  33.8010  <.0001  0.3301  0.3707  *** 
## factor(Cell)I12-I17    0.2845  0.0118  24.0199  <.0001  0.2612  0.3077  *** 
## factor(Cell)I2-I11     0.4515  0.0095  47.7536  <.0001  0.4330  0.4701  *** 
## factor(Cell)I2-I12     0.3560  0.0111  32.0610  <.0001  0.3343  0.3778  *** 
## factor(Cell)I2-I17     0.3894  0.0110  35.5215  <.0001  0.3679  0.4109  *** 
## factor(Cell)I2-I4      0.4277  0.0154  27.7415  <.0001  0.3975  0.4579  *** 
## factor(Cell)I4-I11     0.3569  0.0125  28.6477  <.0001  0.3324  0.3813  *** 
## factor(Cell)I4-I12     0.2874  0.0136  21.2011  <.0001  0.2608  0.3139  *** 
## factor(Cell)I4-I17     0.3216  0.0135  23.8248  <.0001  0.2952  0.3481  *** 
## 
## ---
## Signif. codes:  0 '***' 0.001 '**' 0.01 '*' 0.05 '.' 0.1 ' ' 1
```

```
## Cluster-robust standard errors
summary(
  robust(tas20.mlmvrem3.sens,
         cluster = StudyID,
         clubSandwich = TRUE)
)
```

```
## 
## Multivariate Meta-Analysis Model (k = 880; method: REML)
## 
##     logLik    Deviance         AIC         BIC        AICc   
##   894.8170  -1789.6341  -1747.6341  -1647.4957  -1746.5445   
## 
## Variance Components:
## 
## outer factor: ESID         (nlvls = 880)
## inner factor: factor(Cell) (nlvls = 10)
## 
##             estim    sqrt  fixed 
## tau^2      0.0015  0.0390     no 
## rho        0.5000            yes 
## 
## outer factor: StudyID      (nlvls = 62)
## inner factor: factor(Cell) (nlvls = 10)
## 
##                estim    sqrt  k.lvl  fixed    level 
## gamma^2.1     0.0042  0.0647     62     no  I11-I12 
## gamma^2.2     0.0039  0.0625     62     no  I11-I17 
## gamma^2.3     0.0057  0.0757     62     no  I12-I17 
## gamma^2.4     0.0031  0.0554     62     no   I2-I11 
## gamma^2.5     0.0048  0.0696     62     no   I2-I12 
## gamma^2.6     0.0048  0.0690     62     no   I2-I17 
## gamma^2.7     0.0120  0.1097     62     no    I2-I4 
## gamma^2.8     0.0068  0.0824     62     no   I4-I11 
## gamma^2.9     0.0083  0.0914     62     no   I4-I12 
## gamma^2.10    0.0083  0.0914     62     no   I4-I17 
## phi           0.5000                   yes          
## 
## Test for Residual Heterogeneity:
## QE(df = 870) = 6986.5768, p-val < .0001
## 
## Number of estimates:   880
## Number of clusters:    62
## Estimates per cluster: 10-40 (mean: 14.19, median: 10)
## 
## Test of Moderators (coefficients 1:10):¹
## F(df1 = 10, df2 = 49.3) = 173.7281, p-val < .0001
## 
## Model Results:
## 
##                      estimate      se¹     tval¹     df¹    pval¹   ci.lb¹ 
## factor(Cell)I11-I12    0.3185  0.0138   23.1167   58.19   <.0001   0.2910  
## factor(Cell)I11-I17    0.3504  0.0133   26.3643   58.24   <.0001   0.3238  
## factor(Cell)I12-I17    0.2845  0.0154   18.5302   59.08   <.0001   0.2537  
## factor(Cell)I2-I11     0.4515  0.0119   38.0874   57.91   <.0001   0.4278  
## factor(Cell)I2-I12     0.3560  0.0144   24.8111   58.74   <.0001   0.3273  
## factor(Cell)I2-I17     0.3894  0.0141   27.5956   58.91   <.0001   0.3612  
## factor(Cell)I2-I4      0.4277  0.0200   21.4230   60.49   <.0001   0.3878  
## factor(Cell)I4-I11     0.3569  0.0159   22.3734   59.62   <.0001   0.3249  
## factor(Cell)I4-I12     0.2874  0.0176   16.3364   59.79   <.0001   0.2522  
## factor(Cell)I4-I17     0.3216  0.0176   18.2319   59.88   <.0001   0.2863  
##                       ci.ub¹      
## factor(Cell)I11-I12  0.3461   *** 
## factor(Cell)I11-I17  0.3770   *** 
## factor(Cell)I12-I17  0.3152   *** 
## factor(Cell)I2-I11   0.4753   *** 
## factor(Cell)I2-I12   0.3848   *** 
## factor(Cell)I2-I17   0.4176   *** 
## factor(Cell)I2-I4    0.4676   *** 
## factor(Cell)I4-I11   0.3888   *** 
## factor(Cell)I4-I12   0.3225   *** 
## factor(Cell)I4-I17   0.3569   *** 
## 
## ---
## Signif. codes:  0 '***' 0.001 '**' 0.01 '*' 0.05 '.' 0.1 ' ' 1
## 
## 1) results based on cluster-robust inference (var-cov estimator: CR2,
##    approx t/F-tests and confidence intervals, df: Satterthwaite approx)
```

```
## Sensitivity analysis
## Random effects: rho = 1, phi = 1
## Model specification
tas20.mlmvrem3.sent <- rma.mv(yi = Correlation,
                              V = V, 
                              data = tas20,
                              random = list(~ factor(Cell) | ESID,
                                            ~ factor(Cell) | StudyID),
                              struc = c("CS", "HCS"),
                              rho = 1,
                              phi = 1,
                              method = "REML",
                              mods = ~ factor(Cell) - 1,
                              time = TRUE,
                              sparse = TRUE)
```

```
## 
## Processing time: 0 hours, 1 minute, 20 seconds
```

```
## Model summary
summary(tas20.mlmvrem3.sent)
```

```
## 
## Multivariate Meta-Analysis Model (k = 880; method: REML)
## 
##     logLik    Deviance         AIC         BIC        AICc   
##   929.9608  -1859.9217  -1817.9217  -1717.7833  -1816.8320   
## 
## Variance Components:
## 
## outer factor: ESID         (nlvls = 880)
## inner factor: factor(Cell) (nlvls = 10)
## 
##             estim    sqrt  fixed 
## tau^2      0.0031  0.0559     no 
## rho        1.0000            yes 
## 
## outer factor: StudyID      (nlvls = 62)
## inner factor: factor(Cell) (nlvls = 10)
## 
##                estim    sqrt  k.lvl  fixed    level 
## gamma^2.1     0.0064  0.0800     62     no  I11-I12 
## gamma^2.2     0.0061  0.0781     62     no  I11-I17 
## gamma^2.3     0.0107  0.1033     62     no  I12-I17 
## gamma^2.4     0.0044  0.0663     62     no   I2-I11 
## gamma^2.5     0.0088  0.0940     62     no   I2-I12 
## gamma^2.6     0.0079  0.0888     62     no   I2-I17 
## gamma^2.7     0.0166  0.1290     62     no    I2-I4 
## gamma^2.8     0.0122  0.1107     62     no   I4-I11 
## gamma^2.9     0.0145  0.1204     62     no   I4-I12 
## gamma^2.10    0.0158  0.1257     62     no   I4-I17 
## phi           1.0000                   yes          
## 
## Test for Residual Heterogeneity:
## QE(df = 870) = 6986.5768, p-val < .0001
## 
## Test of Moderators (coefficients 1:10):
## QM(df = 10) = 2386.8092, p-val < .0001
## 
## Model Results:
## 
##                      estimate      se     zval    pval   ci.lb   ci.ub      
## factor(Cell)I11-I12    0.3202  0.0128  24.9886  <.0001  0.2951  0.3453  *** 
## factor(Cell)I11-I17    0.3514  0.0126  27.9566  <.0001  0.3268  0.3760  *** 
## factor(Cell)I12-I17    0.2859  0.0153  18.6585  <.0001  0.2558  0.3159  *** 
## factor(Cell)I2-I11     0.4513  0.0113  40.0934  <.0001  0.4292  0.4733  *** 
## factor(Cell)I2-I12     0.3583  0.0142  25.1680  <.0001  0.3304  0.3862  *** 
## factor(Cell)I2-I17     0.3924  0.0136  28.7893  <.0001  0.3657  0.4192  *** 
## factor(Cell)I2-I4      0.4299  0.0181  23.7668  <.0001  0.3944  0.4653  *** 
## factor(Cell)I4-I11     0.3603  0.0161  22.4166  <.0001  0.3288  0.3918  *** 
## factor(Cell)I4-I12     0.2931  0.0172  16.9926  <.0001  0.2593  0.3269  *** 
## factor(Cell)I4-I17     0.3268  0.0178  18.3358  <.0001  0.2919  0.3617  *** 
## 
## ---
## Signif. codes:  0 '***' 0.001 '**' 0.01 '*' 0.05 '.' 0.1 ' ' 1
```

```
## Cluster-robust standard errors
summary(
  robust(tas20.mlmvrem3.sent,
         cluster = StudyID,
         clubSandwich = TRUE)
)
```

```
## 
## Multivariate Meta-Analysis Model (k = 880; method: REML)
## 
##     logLik    Deviance         AIC         BIC        AICc   
##   929.9608  -1859.9217  -1817.9217  -1717.7833  -1816.8320   
## 
## Variance Components:
## 
## outer factor: ESID         (nlvls = 880)
## inner factor: factor(Cell) (nlvls = 10)
## 
##             estim    sqrt  fixed 
## tau^2      0.0031  0.0559     no 
## rho        1.0000            yes 
## 
## outer factor: StudyID      (nlvls = 62)
## inner factor: factor(Cell) (nlvls = 10)
## 
##                estim    sqrt  k.lvl  fixed    level 
## gamma^2.1     0.0064  0.0800     62     no  I11-I12 
## gamma^2.2     0.0061  0.0781     62     no  I11-I17 
## gamma^2.3     0.0107  0.1033     62     no  I12-I17 
## gamma^2.4     0.0044  0.0663     62     no   I2-I11 
## gamma^2.5     0.0088  0.0940     62     no   I2-I12 
## gamma^2.6     0.0079  0.0888     62     no   I2-I17 
## gamma^2.7     0.0166  0.1290     62     no    I2-I4 
## gamma^2.8     0.0122  0.1107     62     no   I4-I11 
## gamma^2.9     0.0145  0.1204     62     no   I4-I12 
## gamma^2.10    0.0158  0.1257     62     no   I4-I17 
## phi           1.0000                   yes          
## 
## Test for Residual Heterogeneity:
## QE(df = 870) = 6986.5768, p-val < .0001
## 
## Number of estimates:   880
## Number of clusters:    62
## Estimates per cluster: 10-40 (mean: 14.19, median: 10)
## 
## Test of Moderators (coefficients 1:10):¹
## F(df1 = 10, df2 = 41.32) = 173.3837, p-val < .0001
## 
## Model Results:
## 
##                      estimate      se¹     tval¹     df¹    pval¹   ci.lb¹ 
## factor(Cell)I11-I12    0.3202  0.0146   22.0029   58.83   <.0001   0.2911  
## factor(Cell)I11-I17    0.3514  0.0134   26.1450   58.84   <.0001   0.3245  
## factor(Cell)I12-I17    0.2859  0.0154   18.5988    59.9   <.0001   0.2551  
## factor(Cell)I2-I11     0.4513  0.0126   35.9539   58.27   <.0001   0.4262  
## factor(Cell)I2-I12     0.3583  0.0148   24.1512   59.64   <.0001   0.3287  
## factor(Cell)I2-I17     0.3924  0.0143   27.3697   59.51   <.0001   0.3638  
## factor(Cell)I2-I4      0.4299  0.0192   22.4315   60.56   <.0001   0.3915  
## factor(Cell)I4-I11     0.3603  0.0156   23.0478   60.18   <.0001   0.3290  
## factor(Cell)I4-I12     0.2931  0.0173   16.9034   60.28   <.0001   0.2584  
## factor(Cell)I4-I17     0.3268  0.0183   17.8351   60.41   <.0001   0.2902  
##                       ci.ub¹      
## factor(Cell)I11-I12  0.3493   *** 
## factor(Cell)I11-I17  0.3783   *** 
## factor(Cell)I12-I17  0.3166   *** 
## factor(Cell)I2-I11   0.4764   *** 
## factor(Cell)I2-I12   0.3880   *** 
## factor(Cell)I2-I17   0.4211   *** 
## factor(Cell)I2-I4    0.4682   *** 
## factor(Cell)I4-I11   0.3915   *** 
## factor(Cell)I4-I12   0.3278   *** 
## factor(Cell)I4-I17   0.3634   *** 
## 
## ---
## Signif. codes:  0 '***' 0.001 '**' 0.01 '*' 0.05 '.' 0.1 ' ' 1
## 
## 1) results based on cluster-robust inference (var-cov estimator: CR2,
##    approx t/F-tests and confidence intervals, df: Satterthwaite approx)
```

```
## Sensitivity analysis
## Random effects: rho = 0, phi estimated
## Model specification
tas20.mlmvrem3.senu <- rma.mv(yi = Correlation,
                              V = V, 
                              data = tas20,
                              random = list(~ factor(Cell) | ESID,
                                            ~ factor(Cell) | StudyID),
                              struc = c("CS", "HCS"),
                              rho = 0,
                              phi = NA,
                              method = "REML",
                              mods = ~ factor(Cell) - 1,
                              time = TRUE,
                              sparse = TRUE)
```

```
## 
## Processing time: 0 hours, 0 minutes, 53.18 seconds
```

```
## Model summary
summary(tas20.mlmvrem3.senu)
```

```
## 
## Multivariate Meta-Analysis Model (k = 880; method: REML)
## 
##     logLik    Deviance         AIC         BIC        AICc   
##   935.2501  -1870.5003  -1826.5003  -1721.5934  -1825.3055   
## 
## Variance Components:
## 
## outer factor: ESID         (nlvls = 880)
## inner factor: factor(Cell) (nlvls = 10)
## 
##             estim    sqrt  fixed 
## tau^2      0.0022  0.0467     no 
## rho        0.0000            yes 
## 
## outer factor: StudyID      (nlvls = 62)
## inner factor: factor(Cell) (nlvls = 10)
## 
##                estim    sqrt  k.lvl  fixed    level 
## gamma^2.1     0.0075  0.0867     62     no  I11-I12 
## gamma^2.2     0.0068  0.0827     62     no  I11-I17 
## gamma^2.3     0.0111  0.1053     62     no  I12-I17 
## gamma^2.4     0.0053  0.0727     62     no   I2-I11 
## gamma^2.5     0.0094  0.0972     62     no   I2-I12 
## gamma^2.6     0.0086  0.0927     62     no   I2-I17 
## gamma^2.7     0.0200  0.1415     62     no    I2-I4 
## gamma^2.8     0.0126  0.1121     62     no   I4-I11 
## gamma^2.9     0.0152  0.1235     62     no   I4-I12 
## gamma^2.10    0.0161  0.1270     62     no   I4-I17 
## phi           0.9177                    no          
## 
## Test for Residual Heterogeneity:
## QE(df = 870) = 6986.5768, p-val < .0001
## 
## Test of Moderators (coefficients 1:10):
## QM(df = 10) = 2197.2800, p-val < .0001
## 
## Model Results:
## 
##                      estimate      se     zval    pval   ci.lb   ci.ub      
## factor(Cell)I11-I12    0.3206  0.0131  24.3858  <.0001  0.2949  0.3464  *** 
## factor(Cell)I11-I17    0.3519  0.0127  27.7827  <.0001  0.3271  0.3767  *** 
## factor(Cell)I12-I17    0.2862  0.0152  18.7741  <.0001  0.2564  0.3161  *** 
## factor(Cell)I2-I11     0.4524  0.0115  39.4828  <.0001  0.4299  0.4748  *** 
## factor(Cell)I2-I12     0.3587  0.0143  25.1414  <.0001  0.3307  0.3867  *** 
## factor(Cell)I2-I17     0.3922  0.0137  28.6043  <.0001  0.3653  0.4190  *** 
## factor(Cell)I2-I4      0.4302  0.0193  22.2618  <.0001  0.3923  0.4680  *** 
## factor(Cell)I4-I11     0.3601  0.0160  22.5734  <.0001  0.3289  0.3914  *** 
## factor(Cell)I4-I12     0.2919  0.0173  16.8243  <.0001  0.2579  0.3259  *** 
## factor(Cell)I4-I17     0.3257  0.0177  18.3733  <.0001  0.2909  0.3604  *** 
## 
## ---
## Signif. codes:  0 '***' 0.001 '**' 0.01 '*' 0.05 '.' 0.1 ' ' 1
```

```
## Cluster-robust standard errors
summary(
  robust(tas20.mlmvrem3.senu,
         cluster = StudyID,
         clubSandwich = TRUE)
)
```

```
## 
## Multivariate Meta-Analysis Model (k = 880; method: REML)
## 
##     logLik    Deviance         AIC         BIC        AICc   
##   935.2501  -1870.5003  -1826.5003  -1721.5934  -1825.3055   
## 
## Variance Components:
## 
## outer factor: ESID         (nlvls = 880)
## inner factor: factor(Cell) (nlvls = 10)
## 
##             estim    sqrt  fixed 
## tau^2      0.0022  0.0467     no 
## rho        0.0000            yes 
## 
## outer factor: StudyID      (nlvls = 62)
## inner factor: factor(Cell) (nlvls = 10)
## 
##                estim    sqrt  k.lvl  fixed    level 
## gamma^2.1     0.0075  0.0867     62     no  I11-I12 
## gamma^2.2     0.0068  0.0827     62     no  I11-I17 
## gamma^2.3     0.0111  0.1053     62     no  I12-I17 
## gamma^2.4     0.0053  0.0727     62     no   I2-I11 
## gamma^2.5     0.0094  0.0972     62     no   I2-I12 
## gamma^2.6     0.0086  0.0927     62     no   I2-I17 
## gamma^2.7     0.0200  0.1415     62     no    I2-I4 
## gamma^2.8     0.0126  0.1121     62     no   I4-I11 
## gamma^2.9     0.0152  0.1235     62     no   I4-I12 
## gamma^2.10    0.0161  0.1270     62     no   I4-I17 
## phi           0.9177                    no          
## 
## Test for Residual Heterogeneity:
## QE(df = 870) = 6986.5768, p-val < .0001
## 
## Number of estimates:   880
## Number of clusters:    62
## Estimates per cluster: 10-40 (mean: 14.19, median: 10)
## 
## Test of Moderators (coefficients 1:10):¹
## F(df1 = 10, df2 = 45.62) = 165.6487, p-val < .0001
## 
## Model Results:
## 
##                      estimate      se¹     tval¹     df¹    pval¹   ci.lb¹ 
## factor(Cell)I11-I12    0.3206  0.0143   22.4288   59.51   <.0001   0.2920  
## factor(Cell)I11-I17    0.3519  0.0134   26.1958   59.45   <.0001   0.3250  
## factor(Cell)I12-I17    0.2862  0.0154   18.5992   60.15   <.0001   0.2555  
## factor(Cell)I2-I11     0.4524  0.0123   36.8343   59.15   <.0001   0.4278  
## factor(Cell)I2-I12     0.3587  0.0146   24.5603   59.98   <.0001   0.3295  
## factor(Cell)I2-I17     0.3922  0.0142   27.5624   59.93   <.0001   0.3637  
## factor(Cell)I2-I4      0.4302  0.0196   21.9079   60.75   <.0001   0.3909  
## factor(Cell)I4-I11     0.3601  0.0157   22.8857   60.37   <.0001   0.3287  
## factor(Cell)I4-I12     0.2919  0.0175   16.7182   60.47   <.0001   0.2570  
## factor(Cell)I4-I17     0.3257  0.0178   18.2787   60.54   <.0001   0.2900  
##                       ci.ub¹      
## factor(Cell)I11-I12  0.3492   *** 
## factor(Cell)I11-I17  0.3788   *** 
## factor(Cell)I12-I17  0.3170   *** 
## factor(Cell)I2-I11   0.4770   *** 
## factor(Cell)I2-I12   0.3879   *** 
## factor(Cell)I2-I17   0.4206   *** 
## factor(Cell)I2-I4    0.4694   *** 
## factor(Cell)I4-I11   0.3916   *** 
## factor(Cell)I4-I12   0.3268   *** 
## factor(Cell)I4-I17   0.3613   *** 
## 
## ---
## Signif. codes:  0 '***' 0.001 '**' 0.01 '*' 0.05 '.' 0.1 ' ' 1
## 
## 1) results based on cluster-robust inference (var-cov estimator: CR2,
##    approx t/F-tests and confidence intervals, df: Satterthwaite approx)
```

#### Correlation-specific effect sizes, overall within- and between-study heterogeneity (`Model 4`)

This approach assumes that there are two estimates of the amounts of
residual heterogeneity which are the same across the 21 correlations.
Moreover, we assume a zero correlation between the levels. The structure
of the random effects corresponds to a compound symmetric structure (CS)
with \(\rho = \phi = 0\).

This approach differs from the so-called WPL approach in the
meta-analytic structural equation modeling literature (see Wilson et al., 2016 and Stolwijk et al.,
2022). In the WPL approach, \(\rho = \phi
= 1\), and the within-study heterogeneity contains variation
among all correlations within a study, irrespective of their type.

```
## Multilevel no-intercept random-effects model
## Effect sizes nested in articles
## Note: This model assumes a compound symmetric (CS) structure.
## Source: https://wviechtb.github.io/metafor/reference/rma.mv.html

## Model specification
tas20.mlmvrem4 <- rma.mv(yi = Correlation,
                         V = V,
                         data = tas20,
                         random = list(~ factor(Cell) | ESID,
                                       ~ factor(Cell) | StudyID),
                         method = "REML",
                         mods = ~ factor(Cell) - 1,
                         struc = c("CS", "CS"),
                         phi = 0,
                         rho = 0,
                         time = TRUE,
                         sparse = TRUE,
                         control = list(optimizer = "optimParallel", 
                                        ncpus = ncores))
```

```
## 
## Processing time: 0 hours, 0 minutes, 5.15 seconds
```

```
## Model summary
summary(tas20.mlmvrem4)
```

```
## 
## Multivariate Meta-Analysis Model (k = 880; method: REML)
## 
##     logLik    Deviance         AIC         BIC        AICc   
##   692.2354  -1384.4708  -1360.4708  -1303.2489  -1360.1067   
## 
## Variance Components:
## 
## outer factor: ESID         (nlvls = 880)
## inner factor: factor(Cell) (nlvls = 10)
## 
##             estim    sqrt  fixed 
## tau^2      0.0026  0.0515     no 
## rho        0.0000            yes 
## 
## outer factor: StudyID      (nlvls = 62)
## inner factor: factor(Cell) (nlvls = 10)
## 
##             estim    sqrt  fixed 
## gamma^2    0.0068  0.0824     no 
## phi        0.0000            yes 
## 
## Test for Residual Heterogeneity:
## QE(df = 870) = 6986.5768, p-val < .0001
## 
## Test of Moderators (coefficients 1:10):
## QM(df = 10) = 5840.4700, p-val < .0001
## 
## Model Results:
## 
##                      estimate      se     zval    pval   ci.lb   ci.ub      
## factor(Cell)I11-I12    0.3133  0.0131  23.9189  <.0001  0.2876  0.3389  *** 
## factor(Cell)I11-I17    0.3462  0.0130  26.5518  <.0001  0.3206  0.3717  *** 
## factor(Cell)I12-I17    0.2804  0.0131  21.3577  <.0001  0.2546  0.3061  *** 
## factor(Cell)I2-I11     0.4470  0.0129  34.7088  <.0001  0.4218  0.4723  *** 
## factor(Cell)I2-I12     0.3503  0.0130  26.8645  <.0001  0.3247  0.3759  *** 
## factor(Cell)I2-I17     0.3847  0.0130  29.6595  <.0001  0.3593  0.4101  *** 
## factor(Cell)I2-I4      0.4256  0.0129  33.0133  <.0001  0.4004  0.4509  *** 
## factor(Cell)I4-I11     0.3516  0.0130  27.0009  <.0001  0.3261  0.3772  *** 
## factor(Cell)I4-I12     0.2811  0.0131  21.4216  <.0001  0.2554  0.3069  *** 
## factor(Cell)I4-I17     0.3160  0.0131  24.1753  <.0001  0.2904  0.3416  *** 
## 
## ---
## Signif. codes:  0 '***' 0.001 '**' 0.01 '*' 0.05 '.' 0.1 ' ' 1
```

```
## Cluster-robust standard errors
tas20.mlmvrem4.robust <- robust(tas20.mlmvrem4, 
                           cluster = StudyID, 
                           clubSandwich = TRUE)
summary(tas20.mlmvrem4.robust)
```

```
## 
## Multivariate Meta-Analysis Model (k = 880; method: REML)
## 
##     logLik    Deviance         AIC         BIC        AICc   
##   692.2354  -1384.4708  -1360.4708  -1303.2489  -1360.1067   
## 
## Variance Components:
## 
## outer factor: ESID         (nlvls = 880)
## inner factor: factor(Cell) (nlvls = 10)
## 
##             estim    sqrt  fixed 
## tau^2      0.0026  0.0515     no 
## rho        0.0000            yes 
## 
## outer factor: StudyID      (nlvls = 62)
## inner factor: factor(Cell) (nlvls = 10)
## 
##             estim    sqrt  fixed 
## gamma^2    0.0068  0.0824     no 
## phi        0.0000            yes 
## 
## Test for Residual Heterogeneity:
## QE(df = 870) = 6986.5768, p-val < .0001
## 
## Number of estimates:   880
## Number of clusters:    62
## Estimates per cluster: 10-40 (mean: 14.19, median: 10)
## 
## Test of Moderators (coefficients 1:10):¹
## F(df1 = 10, df2 = 50.53) = 179.7611, p-val < .0001
## 
## Model Results:
## 
##                      estimate      se¹     tval¹     df¹    pval¹   ci.lb¹ 
## factor(Cell)I11-I12    0.3133  0.0133   23.5376   59.33   <.0001   0.2866  
## factor(Cell)I11-I17    0.3462  0.0131   26.4020   59.46   <.0001   0.3199  
## factor(Cell)I12-I17    0.2804  0.0151   18.5677   59.32   <.0001   0.2501  
## factor(Cell)I2-I11     0.4470  0.0117   38.2241   59.67   <.0001   0.4237  
## factor(Cell)I2-I12     0.3503  0.0142   24.6161    59.4   <.0001   0.3218  
## factor(Cell)I2-I17     0.3847  0.0142   27.0329   59.52   <.0001   0.3562  
## factor(Cell)I2-I4      0.4256  0.0194   21.8882   59.63   <.0001   0.3867  
## factor(Cell)I4-I11     0.3516  0.0158   22.2590   59.46   <.0001   0.3200  
## factor(Cell)I4-I12     0.2811  0.0176   15.9944   59.28   <.0001   0.2460  
## factor(Cell)I4-I17     0.3160  0.0175   18.0636   59.36   <.0001   0.2810  
##                       ci.ub¹      
## factor(Cell)I11-I12  0.3399   *** 
## factor(Cell)I11-I17  0.3724   *** 
## factor(Cell)I12-I17  0.3106   *** 
## factor(Cell)I2-I11   0.4704   *** 
## factor(Cell)I2-I12   0.3788   *** 
## factor(Cell)I2-I17   0.4131   *** 
## factor(Cell)I2-I4    0.4645   *** 
## factor(Cell)I4-I11   0.3833   *** 
## factor(Cell)I4-I12   0.3163   *** 
## factor(Cell)I4-I17   0.3510   *** 
## 
## ---
## Signif. codes:  0 '***' 0.001 '**' 0.01 '*' 0.05 '.' 0.1 ' ' 1
## 
## 1) results based on cluster-robust inference (var-cov estimator: CR2,
##    approx t/F-tests and confidence intervals, df: Satterthwaite approx)
```

```
## Processing time in seconds
tas20.mlmvrem4$time
```

```
## [1] 5.146
```

```
## Extract relevant elements
## Asymptotic covariance matrix
ACOV.mlmvrem4 <- tas20.mlmvrem4$vb

## Marginal differences between the asymptotic covariance matrices
## cluster-robust vs. not robust
max(tas20.mlmvrem4$vb-tas20.mlmvrem4.robust$vb)
```

```
## [1] 2.911016e-05
```

```
## Pooled correlation matrix
CORR.mlmvrem4 <- vec2symMat(x = c(1, tas20.mlmvrem4$b[7], tas20.mlmvrem4$b[4], tas20.mlmvrem4$b[5], tas20.mlmvrem4$b[6],
                                1, tas20.mlmvrem4$b[8], tas20.mlmvrem4$b[9], tas20.mlmvrem4$b[10],
                                1, tas20.mlmvrem4$b[1], tas20.mlmvrem4$b[2],
                                1, tas20.mlmvrem4$b[3],
                                1),
                          diag = TRUE)

colnames(CORR.mlmvrem4) <- c("Item2", "Item4", "Item11", "Item12", "Item17")
rownames(CORR.mlmvrem4) <- c("Item2", "Item4", "Item11", "Item12", "Item17")

## Inspect the pooled correlation matrix
CORR.mlmvrem4
```

```
##            Item2     Item4    Item11    Item12    Item17
## Item2  1.0000000 0.4256196 0.4470500 0.3503000 0.3846699
## Item4  0.4256196 1.0000000 0.3516450 0.2811302 0.3159751
## Item11 0.4470500 0.3516450 1.0000000 0.3132782 0.3461513
## Item12 0.3503000 0.2811302 0.3132782 1.0000000 0.2803560
## Item17 0.3846699 0.3159751 0.3461513 0.2803560 1.0000000
```

```
## Plot the pooled correlation matrix
corrplot(CORR.mlmvrem4, 
         type = "upper", 
         order = "original", 
         tl.col = "black", 
         tl.srt = 60,
         addCoef.col = "black",
         number.cex = 0.9,
         cl.cex = 1,
         tl.cex = 1)
```

```
## Extract the relevant information
tas20.mlmvrem4.results <- data.frame(round(tas20.mlmvrem4.robust$b[,1],3), 
                               round(tas20.mlmvrem4.robust$se,3),
                               round(tas20.mlmvrem4.robust$ci.lb,3),
                               round(tas20.mlmvrem4.robust$ci.ub,3),
                               round(tas20.mlmvrem4.robust$tau2,3),
                               round(tas20.mlmvrem4.robust$gamma2,3))

colnames(tas20.mlmvrem4.results) <- c("r",
                                "SE",
                                "CI95-low",
                                "CI95-high",
                                "tau2",
                                "gamma2")

## Data frame with results
tas20.mlmvrem4.results
```

```
##                         r    SE CI95-low CI95-high  tau2 gamma2
## factor(Cell)I11-I12 0.313 0.013    0.287     0.340 0.003  0.007
## factor(Cell)I11-I17 0.346 0.013    0.320     0.372 0.003  0.007
## factor(Cell)I12-I17 0.280 0.015    0.250     0.311 0.003  0.007
## factor(Cell)I2-I11  0.447 0.012    0.424     0.470 0.003  0.007
## factor(Cell)I2-I12  0.350 0.014    0.322     0.379 0.003  0.007
## factor(Cell)I2-I17  0.385 0.014    0.356     0.413 0.003  0.007
## factor(Cell)I2-I4   0.426 0.019    0.387     0.465 0.003  0.007
## factor(Cell)I4-I11  0.352 0.016    0.320     0.383 0.003  0.007
## factor(Cell)I4-I12  0.281 0.018    0.246     0.316 0.003  0.007
## factor(Cell)I4-I17  0.316 0.017    0.281     0.351 0.003  0.007
```

```
##write.csv2(tas20.mlmvrem4.results, file = "MLMVREM-Table2-tas20mlmvrem4.csv")


## Create a new model fit data frame
## Source for the computation of the AIC, BIC, and AICc:
## https://github.com/cran/metafor/blob/master/R/rma.mv.r#L1245

newfit.mlmvrem4 <- data.frame(
  c("logLik", "parms", "k",
    "Deviance", 
    "AIC", "BIC", "AICc"),
  c(## logLik
    tas20.mlmvrem4$fit.stats$REML[1],
    ## Number of parameters (parms)
    tas20.mlmvrem4$parms,
    ## Number of effect sizes (k)
    length(tas20$Correlation),
    ## Deviance
    tas20.mlmvrem4$fit.stats$REML[2],
    ## AIC
    tas20.mlmvrem4$fit.stats$REML[3],
    ## BIC
    tas20.mlmvrem4$fit.stats$REML[4],
    ## AICc
    tas20.mlmvrem4$fit.stats$REML[5]))

colnames(newfit.mlmvrem4) <- c("Fit criterion", "mlmvrem4")
newfit.mlmvrem4
```

```
##   Fit criterion   mlmvrem4
## 1        logLik   692.2354
## 2         parms    12.0000
## 3             k   880.0000
## 4      Deviance -1384.4708
## 5           AIC -1360.4708
## 6           BIC -1303.2489
## 7          AICc -1360.1067
```

```
## Alternative model specification
summary( rma.mv(yi = Correlation,
                     V = V,
                     data = tas20,
                     random = list(~ factor(Cell) | ESID,
                                   ~ factor(Cell) | StudyID),
                     method = "REML",
                     mods = ~ factor(Cell) - 1,
                     struc = "ID",
                     time = TRUE,
                     sparse = TRUE,
                     control = list(optimizer = "optimParallel", 
                                    ncpus = ncores)) 
)
```

```
## 
## Processing time: 0 hours, 0 minutes, 4.56 seconds
```

```
## 
## Multivariate Meta-Analysis Model (k = 880; method: REML)
## 
##     logLik    Deviance         AIC         BIC        AICc   
##   692.2354  -1384.4708  -1360.4708  -1303.2489  -1360.1067   
## 
## Variance Components:
## 
## outer factor: ESID         (nlvls = 880)
## inner factor: factor(Cell) (nlvls = 10)
## 
##             estim    sqrt  fixed 
## tau^2      0.0026  0.0515     no 
## 
## outer factor: StudyID      (nlvls = 62)
## inner factor: factor(Cell) (nlvls = 10)
## 
##             estim    sqrt  fixed 
## gamma^2    0.0068  0.0824     no 
## 
## Test for Residual Heterogeneity:
## QE(df = 870) = 6986.5768, p-val < .0001
## 
## Test of Moderators (coefficients 1:10):
## QM(df = 10) = 5840.4700, p-val < .0001
## 
## Model Results:
## 
##                      estimate      se     zval    pval   ci.lb   ci.ub      
## factor(Cell)I11-I12    0.3133  0.0131  23.9189  <.0001  0.2876  0.3389  *** 
## factor(Cell)I11-I17    0.3462  0.0130  26.5518  <.0001  0.3206  0.3717  *** 
## factor(Cell)I12-I17    0.2804  0.0131  21.3577  <.0001  0.2546  0.3061  *** 
## factor(Cell)I2-I11     0.4470  0.0129  34.7088  <.0001  0.4218  0.4723  *** 
## factor(Cell)I2-I12     0.3503  0.0130  26.8645  <.0001  0.3247  0.3759  *** 
## factor(Cell)I2-I17     0.3847  0.0130  29.6595  <.0001  0.3593  0.4101  *** 
## factor(Cell)I2-I4      0.4256  0.0129  33.0133  <.0001  0.4004  0.4509  *** 
## factor(Cell)I4-I11     0.3516  0.0130  27.0009  <.0001  0.3261  0.3772  *** 
## factor(Cell)I4-I12     0.2811  0.0131  21.4216  <.0001  0.2554  0.3069  *** 
## factor(Cell)I4-I17     0.3160  0.0131  24.1753  <.0001  0.2904  0.3416  *** 
## 
## ---
## Signif. codes:  0 '***' 0.001 '**' 0.01 '*' 0.05 '.' 0.1 ' ' 1
```

```
## Sensitivity analysis
## Rho = 1, phi = 1
## Model specification
tas20.mlmvrem4.sent <- rma.mv(yi = Correlation,
                              V = V,
                              data = tas20,
                              random = list(~ factor(Cell) | ESID,
                                            ~ factor(Cell) | StudyID),
                              struc = c("CS", "CS"),
                              rho = 1,
                              phi = 1,
                              method = "REML",
                              mods = ~ factor(Cell) - 1,
                              time = TRUE,
                              sparse = TRUE,
                              control = list(optimizer = "optimParallel", 
                                             ncpus = ncores))
```

```
## 
## Processing time: 0 hours, 0 minutes, 9.44 seconds
```

```
## Model summary
summary(tas20.mlmvrem4.sent)
```

```
## 
## Multivariate Meta-Analysis Model (k = 880; method: REML)
## 
##     logLik    Deviance         AIC         BIC        AICc   
##   906.6167  -1813.2334  -1789.2334  -1732.0114  -1788.8693   
## 
## Variance Components:
## 
## outer factor: ESID         (nlvls = 880)
## inner factor: factor(Cell) (nlvls = 10)
## 
##             estim    sqrt  fixed 
## tau^2      0.0034  0.0584     no 
## rho        1.0000            yes 
## 
## outer factor: StudyID      (nlvls = 62)
## inner factor: factor(Cell) (nlvls = 10)
## 
##             estim    sqrt  fixed 
## gamma^2    0.0100  0.0999     no 
## phi        1.0000            yes 
## 
## Test for Residual Heterogeneity:
## QE(df = 870) = 6986.5768, p-val < .0001
## 
## Test of Moderators (coefficients 1:10):
## QM(df = 10) = 1199.0580, p-val < .0001
## 
## Model Results:
## 
##                      estimate      se     zval    pval   ci.lb   ci.ub      
## factor(Cell)I11-I12    0.3197  0.0151  21.2325  <.0001  0.2902  0.3492  *** 
## factor(Cell)I11-I17    0.3508  0.0150  23.3693  <.0001  0.3214  0.3803  *** 
## factor(Cell)I12-I17    0.2861  0.0151  18.9767  <.0001  0.2565  0.3156  *** 
## factor(Cell)I2-I11     0.4498  0.0149  30.1952  <.0001  0.4206  0.4790  *** 
## factor(Cell)I2-I12     0.3577  0.0150  23.8288  <.0001  0.3283  0.3871  *** 
## factor(Cell)I2-I17     0.3916  0.0150  26.1822  <.0001  0.3623  0.4210  *** 
## factor(Cell)I2-I4      0.4331  0.0149  29.0840  <.0001  0.4039  0.4623  *** 
## factor(Cell)I4-I11     0.3627  0.0150  24.1865  <.0001  0.3333  0.3921  *** 
## factor(Cell)I4-I12     0.2947  0.0151  19.5544  <.0001  0.2651  0.3242  *** 
## factor(Cell)I4-I17     0.3291  0.0150  21.9003  <.0001  0.2996  0.3586  *** 
## 
## ---
## Signif. codes:  0 '***' 0.001 '**' 0.01 '*' 0.05 '.' 0.1 ' ' 1
```

```
## Cluster-robust standard errors
summary(
  robust(tas20.mlmvrem4.sent,
         cluster = StudyID,
         clubSandwich = TRUE)
)
```

```
## 
## Multivariate Meta-Analysis Model (k = 880; method: REML)
## 
##     logLik    Deviance         AIC         BIC        AICc   
##   906.6167  -1813.2334  -1789.2334  -1732.0114  -1788.8693   
## 
## Variance Components:
## 
## outer factor: ESID         (nlvls = 880)
## inner factor: factor(Cell) (nlvls = 10)
## 
##             estim    sqrt  fixed 
## tau^2      0.0034  0.0584     no 
## rho        1.0000            yes 
## 
## outer factor: StudyID      (nlvls = 62)
## inner factor: factor(Cell) (nlvls = 10)
## 
##             estim    sqrt  fixed 
## gamma^2    0.0100  0.0999     no 
## phi        1.0000            yes 
## 
## Test for Residual Heterogeneity:
## QE(df = 870) = 6986.5768, p-val < .0001
## 
## Number of estimates:   880
## Number of clusters:    62
## Estimates per cluster: 10-40 (mean: 14.19, median: 10)
## 
## Test of Moderators (coefficients 1:10):¹
## F(df1 = 10, df2 = 41.52) = 159.1645, p-val < .0001
## 
## Model Results:
## 
##                      estimate      se¹     tval¹     df¹    pval¹   ci.lb¹ 
## factor(Cell)I11-I12    0.3197  0.0144   22.1796   59.74   <.0001   0.2909  
## factor(Cell)I11-I17    0.3508  0.0135   25.9552   59.82   <.0001   0.3238  
## factor(Cell)I12-I17    0.2861  0.0152   18.8135   59.72   <.0001   0.2557  
## factor(Cell)I2-I11     0.4498  0.0128   35.0176   59.99   <.0001   0.4241  
## factor(Cell)I2-I12     0.3577  0.0147   24.3716   59.79   <.0001   0.3283  
## factor(Cell)I2-I17     0.3916  0.0144   27.1290   59.87   <.0001   0.3628  
## factor(Cell)I2-I4      0.4331  0.0186   23.2669   59.95   <.0001   0.3959  
## factor(Cell)I4-I11     0.3627  0.0154   23.5225   59.81   <.0001   0.3318  
## factor(Cell)I4-I12     0.2947  0.0172   17.1711   59.67   <.0001   0.2604  
## factor(Cell)I4-I17     0.3291  0.0183   17.9771   59.72   <.0001   0.2925  
##                       ci.ub¹      
## factor(Cell)I11-I12  0.3486   *** 
## factor(Cell)I11-I17  0.3779   *** 
## factor(Cell)I12-I17  0.3165   *** 
## factor(Cell)I2-I11   0.4754   *** 
## factor(Cell)I2-I12   0.3871   *** 
## factor(Cell)I2-I17   0.4205   *** 
## factor(Cell)I2-I4    0.4704   *** 
## factor(Cell)I4-I11   0.3935   *** 
## factor(Cell)I4-I12   0.3290   *** 
## factor(Cell)I4-I17   0.3657   *** 
## 
## ---
## Signif. codes:  0 '***' 0.001 '**' 0.01 '*' 0.05 '.' 0.1 ' ' 1
## 
## 1) results based on cluster-robust inference (var-cov estimator: CR2,
##    approx t/F-tests and confidence intervals, df: Satterthwaite approx)
```

```
## Sensitivity analysis
## Rho = 0.5, phi = 0.5
## Model specification
tas20.mlmvrem4.sens <- rma.mv(yi = Correlation,
                              V = V,
                              data = tas20,
                              random = list(~ factor(Cell) | ESID,
                                            ~ factor(Cell) | StudyID),
                              struc = c("CS", "CS"),
                              rho = 0.5,
                              phi = 0.5,
                              method = "REML",
                              mods = ~ factor(Cell) - 1,
                              time = TRUE,
                              sparse = TRUE,
                              control = list(optimizer = "optimParallel", 
                                             ncpus = ncores))
```

```
## 
## Processing time: 0 hours, 0 minutes, 8.67 seconds
```

```
## Model summary
summary(tas20.mlmvrem4.sens)
```

```
## 
## Multivariate Meta-Analysis Model (k = 880; method: REML)
## 
##     logLik    Deviance         AIC         BIC        AICc   
##   874.0814  -1748.1629  -1724.1629  -1666.9410  -1723.7988   
## 
## Variance Components:
## 
## outer factor: ESID         (nlvls = 880)
## inner factor: factor(Cell) (nlvls = 10)
## 
##             estim    sqrt  fixed 
## tau^2      0.0017  0.0408     no 
## rho        0.5000            yes 
## 
## outer factor: StudyID      (nlvls = 62)
## inner factor: factor(Cell) (nlvls = 10)
## 
##             estim    sqrt  fixed 
## gamma^2    0.0063  0.0792     no 
## phi        0.5000            yes 
## 
## Test for Residual Heterogeneity:
## QE(df = 870) = 6986.5768, p-val < .0001
## 
## Test of Moderators (coefficients 1:10):
## QM(df = 10) = 2213.5614, p-val < .0001
## 
## Model Results:
## 
##                      estimate      se     zval    pval   ci.lb   ci.ub      
## factor(Cell)I11-I12    0.3182  0.0123  25.9348  <.0001  0.2942  0.3423  *** 
## factor(Cell)I11-I17    0.3502  0.0122  28.6904  <.0001  0.3263  0.3741  *** 
## factor(Cell)I12-I17    0.2844  0.0123  23.1294  <.0001  0.2603  0.3085  *** 
## factor(Cell)I2-I11     0.4506  0.0120  37.4170  <.0001  0.4270  0.4742  *** 
## factor(Cell)I2-I12     0.3552  0.0122  29.0928  <.0001  0.3313  0.3791  *** 
## factor(Cell)I2-I17     0.3887  0.0121  32.0245  <.0001  0.3649  0.4125  *** 
## factor(Cell)I2-I4      0.4307  0.0120  35.7470  <.0001  0.4071  0.4543  *** 
## factor(Cell)I4-I11     0.3583  0.0122  29.3948  <.0001  0.3344  0.3822  *** 
## factor(Cell)I4-I12     0.2886  0.0123  23.4762  <.0001  0.2645  0.3127  *** 
## factor(Cell)I4-I17     0.3231  0.0122  26.4014  <.0001  0.2991  0.3471  *** 
## 
## ---
## Signif. codes:  0 '***' 0.001 '**' 0.01 '*' 0.05 '.' 0.1 ' ' 1
```

```
## Cluster-robust standard errors
summary(
  robust(tas20.mlmvrem4.sens,
         cluster = StudyID,
         clubSandwich = TRUE)
)
```

```
## 
## Multivariate Meta-Analysis Model (k = 880; method: REML)
## 
##     logLik    Deviance         AIC         BIC        AICc   
##   874.0814  -1748.1629  -1724.1629  -1666.9410  -1723.7988   
## 
## Variance Components:
## 
## outer factor: ESID         (nlvls = 880)
## inner factor: factor(Cell) (nlvls = 10)
## 
##             estim    sqrt  fixed 
## tau^2      0.0017  0.0408     no 
## rho        0.5000            yes 
## 
## outer factor: StudyID      (nlvls = 62)
## inner factor: factor(Cell) (nlvls = 10)
## 
##             estim    sqrt  fixed 
## gamma^2    0.0063  0.0792     no 
## phi        0.5000            yes 
## 
## Test for Residual Heterogeneity:
## QE(df = 870) = 6986.5768, p-val < .0001
## 
## Number of estimates:   880
## Number of clusters:    62
## Estimates per cluster: 10-40 (mean: 14.19, median: 10)
## 
## Test of Moderators (coefficients 1:10):¹
## F(df1 = 10, df2 = 49.59) = 178.9525, p-val < .0001
## 
## Model Results:
## 
##                      estimate      se¹     tval¹     df¹    pval¹   ci.lb¹ 
## factor(Cell)I11-I12    0.3182  0.0137   23.2036   59.25   <.0001   0.2908  
## factor(Cell)I11-I17    0.3502  0.0135   26.0328   59.43   <.0001   0.3233  
## factor(Cell)I12-I17    0.2844  0.0154   18.4488   59.27   <.0001   0.2536  
## factor(Cell)I2-I11     0.4506  0.0120   37.5628   59.67   <.0001   0.4266  
## factor(Cell)I2-I12     0.3552  0.0144   24.7170   59.34   <.0001   0.3265  
## factor(Cell)I2-I17     0.3887  0.0143   27.2493    59.5   <.0001   0.3602  
## factor(Cell)I2-I4      0.4307  0.0195   22.1335   59.64   <.0001   0.3917  
## factor(Cell)I4-I11     0.3583  0.0158   22.6553   59.43   <.0001   0.3266  
## factor(Cell)I4-I12     0.2886  0.0176   16.4249    59.2   <.0001   0.2535  
## factor(Cell)I4-I17     0.3231  0.0176   18.3590   59.31   <.0001   0.2879  
##                       ci.ub¹      
## factor(Cell)I11-I12  0.3457   *** 
## factor(Cell)I11-I17  0.3771   *** 
## factor(Cell)I12-I17  0.3153   *** 
## factor(Cell)I2-I11   0.4746   *** 
## factor(Cell)I2-I12   0.3840   *** 
## factor(Cell)I2-I17   0.4173   *** 
## factor(Cell)I2-I4    0.4696   *** 
## factor(Cell)I4-I11   0.3899   *** 
## factor(Cell)I4-I12   0.3238   *** 
## factor(Cell)I4-I17   0.3583   *** 
## 
## ---
## Signif. codes:  0 '***' 0.001 '**' 0.01 '*' 0.05 '.' 0.1 ' ' 1
## 
## 1) results based on cluster-robust inference (var-cov estimator: CR2,
##    approx t/F-tests and confidence intervals, df: Satterthwaite approx)
```

```
## Sensitivity analysis
## Rho = 0, phi estimated
## Model specification
tas20.mlmvrem4.senu <- rma.mv(yi = Correlation,
                              V = V,
                              data = tas20,
                              random = list(~ factor(Cell) | ESID,
                                            ~ factor(Cell) | StudyID),
                              struc = c("CS", "CS"),
                              rho = 0,
                              phi = NA,
                              method = "REML",
                              mods = ~ factor(Cell) - 1,
                              time = TRUE,
                              sparse = TRUE,
                              control = list(optimizer = "optimParallel", 
                                             ncpus = ncores))
```

```
## 
## Processing time: 0 hours, 0 minutes, 7.3 seconds
```

```
## Model summary
summary(tas20.mlmvrem4.senu)
```

```
## 
## Multivariate Meta-Analysis Model (k = 880; method: REML)
## 
##     logLik    Deviance         AIC         BIC        AICc   
##   910.8310  -1821.6619  -1795.6619  -1733.6715  -1795.2367   
## 
## Variance Components:
## 
## outer factor: ESID         (nlvls = 880)
## inner factor: factor(Cell) (nlvls = 10)
## 
##             estim    sqrt  fixed 
## tau^2      0.0025  0.0497     no 
## rho        0.0000            yes 
## 
## outer factor: StudyID      (nlvls = 62)
## inner factor: factor(Cell) (nlvls = 10)
## 
##             estim    sqrt  fixed 
## gamma^2    0.0109  0.1045     no 
## phi        0.9161             no 
## 
## Test for Residual Heterogeneity:
## QE(df = 870) = 6986.5768, p-val < .0001
## 
## Test of Moderators (coefficients 1:10):
## QM(df = 10) = 1160.4139, p-val < .0001
## 
## Model Results:
## 
##                      estimate      se     zval    pval   ci.lb   ci.ub      
## factor(Cell)I11-I12    0.3204  0.0153  20.9878  <.0001  0.2904  0.3503  *** 
## factor(Cell)I11-I17    0.3516  0.0152  23.1073  <.0001  0.3218  0.3814  *** 
## factor(Cell)I12-I17    0.2863  0.0153  18.7335  <.0001  0.2563  0.3162  *** 
## factor(Cell)I2-I11     0.4514  0.0151  29.9056  <.0001  0.4218  0.4810  *** 
## factor(Cell)I2-I12     0.3580  0.0152  23.5235  <.0001  0.3281  0.3878  *** 
## factor(Cell)I2-I17     0.3914  0.0152  25.8105  <.0001  0.3617  0.4211  *** 
## factor(Cell)I2-I4      0.4333  0.0151  28.7034  <.0001  0.4037  0.4629  *** 
## factor(Cell)I4-I11     0.3620  0.0152  23.8147  <.0001  0.3322  0.3918  *** 
## factor(Cell)I4-I12     0.2934  0.0153  19.2000  <.0001  0.2634  0.3233  *** 
## factor(Cell)I4-I17     0.3276  0.0152  21.5007  <.0001  0.2977  0.3575  *** 
## 
## ---
## Signif. codes:  0 '***' 0.001 '**' 0.01 '*' 0.05 '.' 0.1 ' ' 1
```

```
## Cluster-robust standard errors
summary(
  robust(tas20.mlmvrem4.senu,
         cluster = StudyID,
         clubSandwich = TRUE)
)
```

```
## 
## Multivariate Meta-Analysis Model (k = 880; method: REML)
## 
##     logLik    Deviance         AIC         BIC        AICc   
##   910.8310  -1821.6619  -1795.6619  -1733.6715  -1795.2367   
## 
## Variance Components:
## 
## outer factor: ESID         (nlvls = 880)
## inner factor: factor(Cell) (nlvls = 10)
## 
##             estim    sqrt  fixed 
## tau^2      0.0025  0.0497     no 
## rho        0.0000            yes 
## 
## outer factor: StudyID      (nlvls = 62)
## inner factor: factor(Cell) (nlvls = 10)
## 
##             estim    sqrt  fixed 
## gamma^2    0.0109  0.1045     no 
## phi        0.9161             no 
## 
## Test for Residual Heterogeneity:
## QE(df = 870) = 6986.5768, p-val < .0001
## 
## Number of estimates:   880
## Number of clusters:    62
## Estimates per cluster: 10-40 (mean: 14.19, median: 10)
## 
## Test of Moderators (coefficients 1:10):¹
## F(df1 = 10, df2 = 45.79) = 169.5300, p-val < .0001
## 
## Model Results:
## 
##                      estimate      se¹     tval¹     df¹    pval¹   ci.lb¹ 
## factor(Cell)I11-I12    0.3204  0.0142   22.6112   60.08   <.0001   0.2920  
## factor(Cell)I11-I17    0.3516  0.0136   25.9334   60.16   <.0001   0.3245  
## factor(Cell)I12-I17    0.2863  0.0154   18.6407   60.08   <.0001   0.2556  
## factor(Cell)I2-I11     0.4514  0.0124   36.3311   60.29   <.0001   0.4266  
## factor(Cell)I2-I12     0.3580  0.0146   24.5926   60.13   <.0001   0.3288  
## factor(Cell)I2-I17     0.3914  0.0144   27.2624    60.2   <.0001   0.3627  
## factor(Cell)I2-I4      0.4333  0.0190   22.8262   60.28   <.0001   0.3953  
## factor(Cell)I4-I11     0.3620  0.0156   23.2649   60.16   <.0001   0.3309  
## factor(Cell)I4-I12     0.2934  0.0173   16.9105   60.04   <.0001   0.2587  
## factor(Cell)I4-I17     0.3276  0.0178   18.3946    60.1   <.0001   0.2920  
##                       ci.ub¹      
## factor(Cell)I11-I12  0.3487   *** 
## factor(Cell)I11-I17  0.3787   *** 
## factor(Cell)I12-I17  0.3170   *** 
## factor(Cell)I2-I11   0.4763   *** 
## factor(Cell)I2-I12   0.3871   *** 
## factor(Cell)I2-I17   0.4201   *** 
## factor(Cell)I2-I4    0.4712   *** 
## factor(Cell)I4-I11   0.3931   *** 
## factor(Cell)I4-I12   0.3281   *** 
## factor(Cell)I4-I17   0.3632   *** 
## 
## ---
## Signif. codes:  0 '***' 0.001 '**' 0.01 '*' 0.05 '.' 0.1 ' ' 1
## 
## 1) results based on cluster-robust inference (var-cov estimator: CR2,
##    approx t/F-tests and confidence intervals, df: Satterthwaite approx)
```

### Model comparisons and selection

#### Rho and phi fixed to zero

```
## Overview of the model fit
newfit.overview <- cbind.data.frame(newfit.mlmvrem1,
                                    newfit.mlmvrem2$mlmvrem2,
                                    newfit.mlmvrem3$mlmvrem3,
                                    newfit.mlmvrem4$mlmvrem4)

colnames(newfit.overview) <- c("Fit criterion",
                               "Model 1",
                               "Model 2",
                               "Model 3",
                               "Model 4")

newfit.overview
```

```
##   Fit criterion    Model 1    Model 2    Model 3    Model 4
## 1        logLik   728.3722   724.5699   710.9364   692.2354
## 2         parms    30.0000    21.0000    21.0000    12.0000
## 3             k   880.0000   880.0000   880.0000   880.0000
## 4      Deviance -1456.7443 -1449.1398 -1421.8728 -1384.4708
## 5           AIC -1396.7443 -1407.1398 -1379.8728 -1360.4708
## 6           BIC -1253.6895 -1307.0015 -1279.7344 -1303.2489
## 7          AICc -1394.5274 -1406.0502 -1378.7831 -1360.1067
```

```
## Minimum AIC
min(newfit.overview[5,2:5])
```

```
## [1] -1407.14
```

```
## Minimum BIC
min(newfit.overview[6,2:5])
```

```
## [1] -1307.001
```

```
## Minimum AICc
min(newfit.overview[7,2:5])
```

```
## [1] -1406.05
```

```
## Result: Model 2 shows the lowest information criteria.


## Likelihood-ratio tests
## Model 1 vs. Model 2
anova(tas20.mlmvrem1, tas20.mlmvrem2)
```

```
## 
##         df        AIC        BIC       AICc   logLik    LRT   pval        QE 
## Full    30 -1396.7443 -1253.6895 -1394.5274 728.3722               6986.5768 
## Reduced 21 -1407.1398 -1307.0015 -1406.0502 724.5699 7.6045 0.5744 6986.5768
```

```
## Result: Models 1 and 2 do not differ significantly.

## Model 1 vs. Model 3
anova(tas20.mlmvrem1, tas20.mlmvrem3)
```

```
## 
##         df        AIC        BIC       AICc   logLik     LRT   pval        QE 
## Full    30 -1396.7443 -1253.6895 -1394.5274 728.3722                6986.5768 
## Reduced 21 -1379.8728 -1279.7344 -1378.7831 710.9364 34.8716 <.0001 6986.5768
```

```
## Result: Model 1 is preferred over Model 3.

## Model 1 vs. Model 4
anova(tas20.mlmvrem1, tas20.mlmvrem4)
```

```
## 
##         df        AIC        BIC       AICc   logLik     LRT   pval        QE 
## Full    30 -1396.7443 -1253.6895 -1394.5274 728.3722                6986.5768 
## Reduced 12 -1360.4708 -1303.2489 -1360.1067 692.2354 72.2736 <.0001 6986.5768
```

```
## Result: Model 1 is preferred over Model 4.

## Model 2 vs. Model 4
anova(tas20.mlmvrem2, tas20.mlmvrem4)
```

```
## 
##         df        AIC        BIC       AICc   logLik     LRT   pval        QE 
## Full    21 -1407.1398 -1307.0015 -1406.0502 724.5699                6986.5768 
## Reduced 12 -1360.4708 -1303.2489 -1360.1067 692.2354 64.6691 <.0001 6986.5768
```

```
## Result: Model 2 is preferred over Model 4.

## Model 3 vs. Model 4
anova(tas20.mlmvrem3, tas20.mlmvrem4)
```

```
## 
##         df        AIC        BIC       AICc   logLik     LRT   pval        QE 
## Full    21 -1379.8728 -1279.7344 -1378.7831 710.9364                6986.5768 
## Reduced 12 -1360.4708 -1303.2489 -1360.1067 692.2354 37.4020 <.0001 6986.5768
```

```
## Result: Model 3 is preferred over Model 4.

## Result: Preference of Model 2
```

Overall, Model 2 (i.e., `mlmvrem2`) seems a reasonable
model choice. In the subsequent moderator analyses, we illustrate the
model specification and estimation using the parsimonious Model 2.

#### Rho and phi fixed to 0.5

```
## Extract the fit indices
### Create a new model fit data frame
newfit.mlmvrem1.sens <- data.frame(
  c("logLik", "parms", "k",
    "Deviance", 
    "AIC", "BIC", "AICc"),
  c(## logLik
    tas20.mlmvrem1.sens$fit.stats$REML[1],
    ## Number of parameters (parms)
    tas20.mlmvrem1.sens$parms,
    ## Number of effect sizes (k)
    length(tas20$Correlation),
    ## Deviance
    tas20.mlmvrem1.sens$fit.stats$REML[2],
    ## AIC
    tas20.mlmvrem1.sens$fit.stats$REML[3],
    ## BIC
    tas20.mlmvrem1.sens$fit.stats$REML[4],
    ## AICc
    tas20.mlmvrem1.sens$fit.stats$REML[5]))

colnames(newfit.mlmvrem1.sens) <- c("Fit criterion", "mlmvrem1.sens")
newfit.mlmvrem1.sens
```

```
##   Fit criterion mlmvrem1.sens
## 1        logLik      914.5862
## 2         parms       30.0000
## 3             k      880.0000
## 4      Deviance    -1829.1723
## 5           AIC    -1769.1723
## 6           BIC    -1626.1175
## 7          AICc    -1766.9554
```

```
### Create a new model fit data frame
newfit.mlmvrem2.sens <- data.frame(
  c("logLik", "parms", "k",
    "Deviance", 
    "AIC", "BIC", "AICc"),
  c(## logLik
    tas20.mlmvrem2.sens$fit.stats$REML[1],
    ## Number of parameters (parms)
    tas20.mlmvrem2.sens$parms,
    ## Number of effect sizes (k)
    length(tas20$Correlation),
    ## Deviance
    tas20.mlmvrem2.sens$fit.stats$REML[2],
    ## AIC
    tas20.mlmvrem2.sens$fit.stats$REML[3],
    ## BIC
    tas20.mlmvrem2.sens$fit.stats$REML[4],
    ## AICc
    tas20.mlmvrem2.sens$fit.stats$REML[5]))

colnames(newfit.mlmvrem2.sens) <- c("Fit criterion", "mlmvrem2.sens")
newfit.mlmvrem2.sens
```

```
##   Fit criterion mlmvrem2.sens
## 1        logLik      904.2915
## 2         parms       21.0000
## 3             k      880.0000
## 4      Deviance    -1808.5830
## 5           AIC    -1766.5830
## 6           BIC    -1666.4447
## 7          AICc    -1765.4934
```

```
### Create a new model fit data frame
newfit.mlmvrem3.sens <- data.frame(
  c("logLik", "parms", "k",
    "Deviance", 
    "AIC", "BIC", "AICc"),
  c(## logLik
    tas20.mlmvrem3.sens$fit.stats$REML[1],
    ## Number of parameters (parms)
    tas20.mlmvrem3.sens$parms,
    ## Number of effect sizes (k)
    length(tas20$Correlation),
    ## Deviance
    tas20.mlmvrem3.sens$fit.stats$REML[2],
    ## AIC
    tas20.mlmvrem3.sens$fit.stats$REML[3],
    ## BIC
    tas20.mlmvrem3.sens$fit.stats$REML[4],
    ## AICc
    tas20.mlmvrem3.sens$fit.stats$REML[5]))

colnames(newfit.mlmvrem3.sens) <- c("Fit criterion", "mlmvrem3.sens")
newfit.mlmvrem3.sens
```

```
##   Fit criterion mlmvrem3.sens
## 1        logLik       894.817
## 2         parms        21.000
## 3             k       880.000
## 4      Deviance     -1789.634
## 5           AIC     -1747.634
## 6           BIC     -1647.496
## 7          AICc     -1746.544
```

```
### Create a new model fit data frame
newfit.mlmvrem4.sens <- data.frame(
  c("logLik", "parms", "k",
    "Deviance", 
    "AIC", "BIC", "AICc"),
  c(## logLik
    tas20.mlmvrem4.sens$fit.stats$REML[1],
    ## Number of parameters (parms)
    tas20.mlmvrem4.sens$parms,
    ## Number of effect sizes (k)
    length(tas20$Correlation),
    ## Deviance
    tas20.mlmvrem4.sens$fit.stats$REML[2],
    ## AIC
    tas20.mlmvrem4.sens$fit.stats$REML[3],
    ## BIC
    tas20.mlmvrem4.sens$fit.stats$REML[4],
    ## AICc
    tas20.mlmvrem4.sens$fit.stats$REML[5]))

colnames(newfit.mlmvrem4.sens) <- c("Fit criterion", "mlmvrem4.sens")
newfit.mlmvrem4.sens
```

```
##   Fit criterion mlmvrem4.sens
## 1        logLik      874.0814
## 2         parms       12.0000
## 3             k      880.0000
## 4      Deviance    -1748.1629
## 5           AIC    -1724.1629
## 6           BIC    -1666.9410
## 7          AICc    -1723.7988
```

```
## Overview of the model fit
newfit.overview.sens <- cbind.data.frame(newfit.mlmvrem1.sens,
                                    newfit.mlmvrem2.sens$mlmvrem2.sens,
                                    newfit.mlmvrem3.sens$mlmvrem3.sens,
                                    newfit.mlmvrem4.sens$mlmvrem4.sens)

colnames(newfit.overview.sens) <- c("Fit criterion",
                               "Model 1",
                               "Model 2",
                               "Model 3",
                               "Model 4")

newfit.overview.sens
```

```
##   Fit criterion    Model 1    Model 2   Model 3    Model 4
## 1        logLik   914.5862   904.2915   894.817   874.0814
## 2         parms    30.0000    21.0000    21.000    12.0000
## 3             k   880.0000   880.0000   880.000   880.0000
## 4      Deviance -1829.1723 -1808.5830 -1789.634 -1748.1629
## 5           AIC -1769.1723 -1766.5830 -1747.634 -1724.1629
## 6           BIC -1626.1175 -1666.4447 -1647.496 -1666.9410
## 7          AICc -1766.9554 -1765.4934 -1746.544 -1723.7988
```

```
## Additional comparisons
anova(tas20.mlmvrem1.sens, tas20.mlmvrem2.sens)
```

```
## 
##         df        AIC        BIC       AICc   logLik     LRT   pval        QE 
## Full    30 -1769.1723 -1626.1175 -1766.9554 914.5862                6986.5768 
## Reduced 21 -1766.5830 -1666.4447 -1765.4934 904.2915 20.5893 0.0146 6986.5768
```

```
## Result: Model 1 is preferred over Model 2.

anova(tas20.mlmvrem1.sens, tas20.mlmvrem3.sens)
```

```
## 
##         df        AIC        BIC       AICc   logLik     LRT   pval        QE 
## Full    30 -1769.1723 -1626.1175 -1766.9554 914.5862                6986.5768 
## Reduced 21 -1747.6341 -1647.4957 -1746.5445 894.8170 39.5383 <.0001 6986.5768
```

```
## Result: Model 1 is preferred over Model 3.

anova(tas20.mlmvrem1.sens, tas20.mlmvrem4.sens)
```

```
## 
##         df        AIC        BIC       AICc   logLik     LRT   pval        QE 
## Full    30 -1769.1723 -1626.1175 -1766.9554 914.5862                6986.5768 
## Reduced 12 -1724.1629 -1666.9410 -1723.7988 874.0814 81.0094 <.0001 6986.5768
```

```
## Result: Model 1 is preferred over Model 4.
```

`Model 1` is favored over the other models (given the AIC,
AICs, and outcomes of LRTs).

#### Rho and phi fixed to 1.0

```
## Sensitivity analyses
## Model 1


## Rho = Phi = 1
## Random effects are considered dependent.
## Source: https://wviechtb.github.io/metafor/reference/rma.mv.html

## Model specification
tas20.mlmvrem1.sent <- rma.mv(yi = Correlation,
                              V = V, 
                              data = tas20,
                              random = list(~ factor(Cell) | ESID,
                                            ~ factor(Cell) | StudyID),
                              struc = c("HCS", "HCS"),
                              rho = 1,
                              phi = 1,
                              method = "REML",
                              mods = ~ factor(Cell) - 1,
                              time = TRUE,
                              sparse = TRUE,
                              control = list(optimizer = "optimParallel",
                                             ncpus = ncores))
```

```
## 
## Processing time: 0 hours, 0 minutes, 57.77 seconds
```

```
## Model summary
summary(tas20.mlmvrem1.sent)
```

```
## 
## Multivariate Meta-Analysis Model (k = 880; method: REML)
## 
##     logLik    Deviance         AIC         BIC        AICc   
##   950.2943  -1900.5887  -1840.5887  -1697.5339  -1838.3718   
## 
## Variance Components:
## 
## outer factor: ESID         (nlvls = 880)
## inner factor: factor(Cell) (nlvls = 10)
## 
##              estim    sqrt  k.lvl  fixed    level 
## tau^2.1     0.0019  0.0436     88     no  I11-I12 
## tau^2.2     0.0018  0.0424     88     no  I11-I17 
## tau^2.3     0.0015  0.0384     88     no  I12-I17 
## tau^2.4     0.0018  0.0426     88     no   I2-I11 
## tau^2.5     0.0018  0.0421     88     no   I2-I12 
## tau^2.6     0.0027  0.0520     88     no   I2-I17 
## tau^2.7     0.0090  0.0946     88     no    I2-I4 
## tau^2.8     0.0037  0.0607     88     no   I4-I11 
## tau^2.9     0.0043  0.0658     88     no   I4-I12 
## tau^2.10    0.0047  0.0684     88     no   I4-I17 
## rho         1.0000                   yes          
## 
## outer factor: StudyID      (nlvls = 62)
## inner factor: factor(Cell) (nlvls = 10)
## 
##                estim    sqrt  k.lvl  fixed    level 
## gamma^2.1     0.0081  0.0900     62     no  I11-I12 
## gamma^2.2     0.0073  0.0855     62     no  I11-I17 
## gamma^2.3     0.0118  0.1088     62     no  I12-I17 
## gamma^2.4     0.0052  0.0718     62     no   I2-I11 
## gamma^2.5     0.0099  0.0995     62     no   I2-I12 
## gamma^2.6     0.0083  0.0912     62     no   I2-I17 
## gamma^2.7     0.0134  0.1156     62     no    I2-I4 
## gamma^2.8     0.0105  0.1025     62     no   I4-I11 
## gamma^2.9     0.0127  0.1128     62     no   I4-I12 
## gamma^2.10    0.0136  0.1166     62     no   I4-I17 
## phi           1.0000                   yes          
## 
## Test for Residual Heterogeneity:
## QE(df = 870) = 6986.5768, p-val < .0001
## 
## Test of Moderators (coefficients 1:10):
## QM(df = 10) = 2425.0050, p-val < .0001
## 
## Model Results:
## 
##                      estimate      se     zval    pval   ci.lb   ci.ub      
## factor(Cell)I11-I12    0.3213  0.0133  24.1251  <.0001  0.2952  0.3474  *** 
## factor(Cell)I11-I17    0.3518  0.0127  27.6381  <.0001  0.3269  0.3768  *** 
## factor(Cell)I12-I17    0.2864  0.0153  18.7097  <.0001  0.2564  0.3164  *** 
## factor(Cell)I2-I11     0.4530  0.0111  40.7704  <.0001  0.4312  0.4747  *** 
## factor(Cell)I2-I12     0.3602  0.0143  25.1940  <.0001  0.3322  0.3882  *** 
## factor(Cell)I2-I17     0.3930  0.0137  28.6438  <.0001  0.3661  0.4199  *** 
## factor(Cell)I2-I4      0.4287  0.0185  23.2025  <.0001  0.3925  0.4649  *** 
## factor(Cell)I4-I11     0.3611  0.0154  23.4739  <.0001  0.3310  0.3913  *** 
## factor(Cell)I4-I12     0.2939  0.0168  17.4696  <.0001  0.2609  0.3269  *** 
## factor(Cell)I4-I17     0.3271  0.0173  18.8916  <.0001  0.2932  0.3611  *** 
## 
## ---
## Signif. codes:  0 '***' 0.001 '**' 0.01 '*' 0.05 '.' 0.1 ' ' 1
```

```
## Cluster-robust standard errors
summary(
  robust(tas20.mlmvrem1.sent,
         cluster = StudyID,
         clubSandwich = TRUE)
)
```

```
## 
## Multivariate Meta-Analysis Model (k = 880; method: REML)
## 
##     logLik    Deviance         AIC         BIC        AICc   
##   950.2943  -1900.5887  -1840.5887  -1697.5339  -1838.3718   
## 
## Variance Components:
## 
## outer factor: ESID         (nlvls = 880)
## inner factor: factor(Cell) (nlvls = 10)
## 
##              estim    sqrt  k.lvl  fixed    level 
## tau^2.1     0.0019  0.0436     88     no  I11-I12 
## tau^2.2     0.0018  0.0424     88     no  I11-I17 
## tau^2.3     0.0015  0.0384     88     no  I12-I17 
## tau^2.4     0.0018  0.0426     88     no   I2-I11 
## tau^2.5     0.0018  0.0421     88     no   I2-I12 
## tau^2.6     0.0027  0.0520     88     no   I2-I17 
## tau^2.7     0.0090  0.0946     88     no    I2-I4 
## tau^2.8     0.0037  0.0607     88     no   I4-I11 
## tau^2.9     0.0043  0.0658     88     no   I4-I12 
## tau^2.10    0.0047  0.0684     88     no   I4-I17 
## rho         1.0000                   yes          
## 
## outer factor: StudyID      (nlvls = 62)
## inner factor: factor(Cell) (nlvls = 10)
## 
##                estim    sqrt  k.lvl  fixed    level 
## gamma^2.1     0.0081  0.0900     62     no  I11-I12 
## gamma^2.2     0.0073  0.0855     62     no  I11-I17 
## gamma^2.3     0.0118  0.1088     62     no  I12-I17 
## gamma^2.4     0.0052  0.0718     62     no   I2-I11 
## gamma^2.5     0.0099  0.0995     62     no   I2-I12 
## gamma^2.6     0.0083  0.0912     62     no   I2-I17 
## gamma^2.7     0.0134  0.1156     62     no    I2-I4 
## gamma^2.8     0.0105  0.1025     62     no   I4-I11 
## gamma^2.9     0.0127  0.1128     62     no   I4-I12 
## gamma^2.10    0.0136  0.1166     62     no   I4-I17 
## phi           1.0000                   yes          
## 
## Test for Residual Heterogeneity:
## QE(df = 870) = 6986.5768, p-val < .0001
## 
## Number of estimates:   880
## Number of clusters:    62
## Estimates per cluster: 10-40 (mean: 14.19, median: 10)
## 
## Test of Moderators (coefficients 1:10):¹
## F(df1 = 10, df2 = 40.88) = 178.7110, p-val < .0001
## 
## Model Results:
## 
##                      estimate      se¹     tval¹     df¹    pval¹   ci.lb¹ 
## factor(Cell)I11-I12    0.3213  0.0144   22.2927   59.69   <.0001   0.2925  
## factor(Cell)I11-I17    0.3518  0.0133   26.4425   59.64   <.0001   0.3252  
## factor(Cell)I12-I17    0.2864  0.0153   18.6707   60.31   <.0001   0.2557  
## factor(Cell)I2-I11     0.4530  0.0123   36.8934   59.18   <.0001   0.4284  
## factor(Cell)I2-I12     0.3602  0.0147   24.5274   60.11   <.0001   0.3308  
## factor(Cell)I2-I17     0.3930  0.0142   27.5889   59.72   <.0001   0.3645  
## factor(Cell)I2-I4      0.4287  0.0189   22.6866    59.4   <.0001   0.3909  
## factor(Cell)I4-I11     0.3611  0.0154   23.4197   59.84   <.0001   0.3303  
## factor(Cell)I4-I12     0.2939  0.0171   17.1719   59.91   <.0001   0.2596  
## factor(Cell)I4-I17     0.3271  0.0183   17.8641   59.99   <.0001   0.2905  
##                       ci.ub¹      
## factor(Cell)I11-I12  0.3502   *** 
## factor(Cell)I11-I17  0.3784   *** 
## factor(Cell)I12-I17  0.3171   *** 
## factor(Cell)I2-I11   0.4775   *** 
## factor(Cell)I2-I12   0.3896   *** 
## factor(Cell)I2-I17   0.4215   *** 
## factor(Cell)I2-I4    0.4665   *** 
## factor(Cell)I4-I11   0.3920   *** 
## factor(Cell)I4-I12   0.3281   *** 
## factor(Cell)I4-I17   0.3638   *** 
## 
## ---
## Signif. codes:  0 '***' 0.001 '**' 0.01 '*' 0.05 '.' 0.1 ' ' 1
## 
## 1) results based on cluster-robust inference (var-cov estimator: CR2,
##    approx t/F-tests and confidence intervals, df: Satterthwaite approx)
```

```
## Sensitivity analyses
## Rho = 0, phi estimated
## Model specification
tas20.mlmvrem1.senu <- rma.mv(yi = Correlation,
                              V = V, 
                              data = tas20,
                              random = list(~ factor(Cell) | ESID,
                                            ~ factor(Cell) | StudyID),
                              struc = c("HCS", "HCS"),
                              rho = 0,
                              phi = NA,
                              method = "REML",
                              mods = ~ factor(Cell) - 1,
                              time = TRUE,
                              sparse = TRUE,
                              control = list(optimizer = "optimParallel",
                                             ncpus = ncores))
```

```
## 
## Processing time: 0 hours, 1 minute, 25 seconds
```

```
## Model summary
summary(tas20.mlmvrem1.senu)
```

```
## 
## Multivariate Meta-Analysis Model (k = 880; method: REML)
## 
##     logLik    Deviance         AIC         BIC        AICc   
##   953.4645  -1906.9291  -1844.9291  -1697.1058  -1842.5615   
## 
## Variance Components:
## 
## outer factor: ESID         (nlvls = 880)
## inner factor: factor(Cell) (nlvls = 10)
## 
##              estim    sqrt  k.lvl  fixed    level 
## tau^2.1     0.0011  0.0330     88     no  I11-I12 
## tau^2.2     0.0011  0.0338     88     no  I11-I17 
## tau^2.3     0.0010  0.0320     88     no  I12-I17 
## tau^2.4     0.0011  0.0326     88     no   I2-I11 
## tau^2.5     0.0014  0.0368     88     no   I2-I12 
## tau^2.6     0.0019  0.0441     88     no   I2-I17 
## tau^2.7     0.0077  0.0876     88     no    I2-I4 
## tau^2.8     0.0028  0.0525     88     no   I4-I11 
## tau^2.9     0.0033  0.0578     88     no   I4-I12 
## tau^2.10    0.0032  0.0570     88     no   I4-I17 
## rho         0.0000                   yes          
## 
## outer factor: StudyID      (nlvls = 62)
## inner factor: factor(Cell) (nlvls = 10)
## 
##                estim    sqrt  k.lvl  fixed    level 
## gamma^2.1     0.0088  0.0940     62     no  I11-I12 
## gamma^2.2     0.0079  0.0891     62     no  I11-I17 
## gamma^2.3     0.0122  0.1105     62     no  I12-I17 
## gamma^2.4     0.0060  0.0774     62     no   I2-I11 
## gamma^2.5     0.0101  0.1007     62     no   I2-I12 
## gamma^2.6     0.0090  0.0947     62     no   I2-I17 
## gamma^2.7     0.0149  0.1221     62     no    I2-I4 
## gamma^2.8     0.0116  0.1078     62     no   I4-I11 
## gamma^2.9     0.0137  0.1171     62     no   I4-I12 
## gamma^2.10    0.0149  0.1223     62     no   I4-I17 
## phi           0.9252                    no          
## 
## Test for Residual Heterogeneity:
## QE(df = 870) = 6986.5768, p-val < .0001
## 
## Test of Moderators (coefficients 1:10):
## QM(df = 10) = 2260.3056, p-val < .0001
## 
## Model Results:
## 
##                      estimate      se     zval    pval   ci.lb   ci.ub      
## factor(Cell)I11-I12    0.3220  0.0135  23.9160  <.0001  0.2956  0.3484  *** 
## factor(Cell)I11-I17    0.3525  0.0129  27.3645  <.0001  0.3273  0.3778  *** 
## factor(Cell)I12-I17    0.2868  0.0154  18.6393  <.0001  0.2566  0.3170  *** 
## factor(Cell)I2-I11     0.4544  0.0114  40.0026  <.0001  0.4321  0.4767  *** 
## factor(Cell)I2-I12     0.3600  0.0143  25.1522  <.0001  0.3320  0.3881  *** 
## factor(Cell)I2-I17     0.3926  0.0138  28.3864  <.0001  0.3655  0.4197  *** 
## factor(Cell)I2-I4      0.4288  0.0188  22.7805  <.0001  0.3919  0.4657  *** 
## factor(Cell)I4-I11     0.3607  0.0157  23.0131  <.0001  0.3300  0.3914  *** 
## factor(Cell)I4-I12     0.2926  0.0170  17.1826  <.0001  0.2592  0.3260  *** 
## factor(Cell)I4-I17     0.3258  0.0175  18.5785  <.0001  0.2915  0.3602  *** 
## 
## ---
## Signif. codes:  0 '***' 0.001 '**' 0.01 '*' 0.05 '.' 0.1 ' ' 1
```

```
## Cluster-robust standard errors
summary(
  robust(tas20.mlmvrem1.senu,
         cluster = StudyID,
         clubSandwich = TRUE)
)
```

```
## 
## Multivariate Meta-Analysis Model (k = 880; method: REML)
## 
##     logLik    Deviance         AIC         BIC        AICc   
##   953.4645  -1906.9291  -1844.9291  -1697.1058  -1842.5615   
## 
## Variance Components:
## 
## outer factor: ESID         (nlvls = 880)
## inner factor: factor(Cell) (nlvls = 10)
## 
##              estim    sqrt  k.lvl  fixed    level 
## tau^2.1     0.0011  0.0330     88     no  I11-I12 
## tau^2.2     0.0011  0.0338     88     no  I11-I17 
## tau^2.3     0.0010  0.0320     88     no  I12-I17 
## tau^2.4     0.0011  0.0326     88     no   I2-I11 
## tau^2.5     0.0014  0.0368     88     no   I2-I12 
## tau^2.6     0.0019  0.0441     88     no   I2-I17 
## tau^2.7     0.0077  0.0876     88     no    I2-I4 
## tau^2.8     0.0028  0.0525     88     no   I4-I11 
## tau^2.9     0.0033  0.0578     88     no   I4-I12 
## tau^2.10    0.0032  0.0570     88     no   I4-I17 
## rho         0.0000                   yes          
## 
## outer factor: StudyID      (nlvls = 62)
## inner factor: factor(Cell) (nlvls = 10)
## 
##                estim    sqrt  k.lvl  fixed    level 
## gamma^2.1     0.0088  0.0940     62     no  I11-I12 
## gamma^2.2     0.0079  0.0891     62     no  I11-I17 
## gamma^2.3     0.0122  0.1105     62     no  I12-I17 
## gamma^2.4     0.0060  0.0774     62     no   I2-I11 
## gamma^2.5     0.0101  0.1007     62     no   I2-I12 
## gamma^2.6     0.0090  0.0947     62     no   I2-I17 
## gamma^2.7     0.0149  0.1221     62     no    I2-I4 
## gamma^2.8     0.0116  0.1078     62     no   I4-I11 
## gamma^2.9     0.0137  0.1171     62     no   I4-I12 
## gamma^2.10    0.0149  0.1223     62     no   I4-I17 
## phi           0.9252                    no          
## 
## Test for Residual Heterogeneity:
## QE(df = 870) = 6986.5768, p-val < .0001
## 
## Number of estimates:   880
## Number of clusters:    62
## Estimates per cluster: 10-40 (mean: 14.19, median: 10)
## 
## Test of Moderators (coefficients 1:10):¹
## F(df1 = 10, df2 = 45.12) = 179.7401, p-val < .0001
## 
## Model Results:
## 
##                      estimate      se¹     tval¹     df¹    pval¹   ci.lb¹ 
## factor(Cell)I11-I12    0.3220  0.0142   22.6635   60.01   <.0001   0.2936  
## factor(Cell)I11-I17    0.3525  0.0134   26.3397   59.95   <.0001   0.3258  
## factor(Cell)I12-I17    0.2868  0.0155   18.5165   60.41   <.0001   0.2558  
## factor(Cell)I2-I11     0.4544  0.0121   37.6789   59.74   <.0001   0.4303  
## factor(Cell)I2-I12     0.3600  0.0145   24.7623   60.21   <.0001   0.3310  
## factor(Cell)I2-I17     0.3926  0.0142   27.7129   60.04   <.0001   0.3643  
## factor(Cell)I2-I4      0.4288  0.0191   22.4370   59.88   <.0001   0.3906  
## factor(Cell)I4-I11     0.3607  0.0156   23.1334    60.2   <.0001   0.3295  
## factor(Cell)I4-I12     0.2926  0.0173   16.9605   60.21   <.0001   0.2581  
## factor(Cell)I4-I17     0.3258  0.0179   18.2306   60.36   <.0001   0.2901  
##                       ci.ub¹      
## factor(Cell)I11-I12  0.3504   *** 
## factor(Cell)I11-I17  0.3793   *** 
## factor(Cell)I12-I17  0.3178   *** 
## factor(Cell)I2-I11   0.4785   *** 
## factor(Cell)I2-I12   0.3891   *** 
## factor(Cell)I2-I17   0.4210   *** 
## factor(Cell)I2-I4    0.4670   *** 
## factor(Cell)I4-I11   0.3919   *** 
## factor(Cell)I4-I12   0.3271   *** 
## factor(Cell)I4-I17   0.3616   *** 
## 
## ---
## Signif. codes:  0 '***' 0.001 '**' 0.01 '*' 0.05 '.' 0.1 ' ' 1
## 
## 1) results based on cluster-robust inference (var-cov estimator: CR2,
##    approx t/F-tests and confidence intervals, df: Satterthwaite approx)
```

```
## Extract the fit indices
### Create a new model fit data frame
newfit.mlmvrem1.sent <- data.frame(
  c("logLik", "parms", "k",
    "Deviance", 
    "AIC", "BIC", "AICc"),
  c(## logLik
    tas20.mlmvrem1.sent$fit.stats$REML[1],
    ## Number of parameters (parms)
    tas20.mlmvrem1.sent$parms,
    ## Number of effect sizes (k)
    length(tas20$Correlation),
    ## Deviance
    tas20.mlmvrem1.sent$fit.stats$REML[2],
    ## AIC
    tas20.mlmvrem1.sent$fit.stats$REML[3],
    ## BIC
    tas20.mlmvrem1.sent$fit.stats$REML[4],
    ## AICc
    tas20.mlmvrem1.sent$fit.stats$REML[5]))

colnames(newfit.mlmvrem1.sent) <- c("Fit criterion", "mlmvrem1.sent")
newfit.mlmvrem1.sent
```

```
##   Fit criterion mlmvrem1.sent
## 1        logLik      950.2943
## 2         parms       30.0000
## 3             k      880.0000
## 4      Deviance    -1900.5887
## 5           AIC    -1840.5887
## 6           BIC    -1697.5339
## 7          AICc    -1838.3718
```

```
### Create a new model fit data frame
newfit.mlmvrem2.sent <- data.frame(
  c("logLik", "parms", "k",
    "Deviance", 
    "AIC", "BIC", "AICc"),
  c(## logLik
    tas20.mlmvrem2.sent$fit.stats$REML[1],
    ## Number of parameters (parms)
    tas20.mlmvrem2.sent$parms,
    ## Number of effect sizes (k)
    length(tas20$Correlation),
    ## Deviance
    tas20.mlmvrem2.sent$fit.stats$REML[2],
    ## AIC
    tas20.mlmvrem2.sent$fit.stats$REML[3],
    ## BIC
    tas20.mlmvrem2.sent$fit.stats$REML[4],
    ## AICc
    tas20.mlmvrem2.sent$fit.stats$REML[5]))

colnames(newfit.mlmvrem2.sent) <- c("Fit criterion", "mlmvrem2.sent")
newfit.mlmvrem2.sent
```

```
##   Fit criterion mlmvrem2.sent
## 1        logLik      938.0735
## 2         parms       21.0000
## 3             k      880.0000
## 4      Deviance    -1876.1470
## 5           AIC    -1834.1470
## 6           BIC    -1734.0087
## 7          AICc    -1833.0574
```

```
### Create a new model fit data frame
newfit.mlmvrem3.sent <- data.frame(
  c("logLik", "parms", "k",
    "Deviance", 
    "AIC", "BIC", "AICc"),
  c(## logLik
    tas20.mlmvrem3.sent$fit.stats$REML[1],
    ## Number of parameters (parms)
    tas20.mlmvrem3.sent$parms,
    ## Number of effect sizes (k)
    length(tas20$Correlation),
    ## Deviance
    tas20.mlmvrem3.sent$fit.stats$REML[2],
    ## AIC
    tas20.mlmvrem3.sent$fit.stats$REML[3],
    ## BIC
    tas20.mlmvrem3.sent$fit.stats$REML[4],
    ## AICc
    tas20.mlmvrem3.sent$fit.stats$REML[5]))

colnames(newfit.mlmvrem3.sent) <- c("Fit criterion", "mlmvrem3.sent")
newfit.mlmvrem3.sent
```

```
##   Fit criterion mlmvrem3.sent
## 1        logLik      929.9608
## 2         parms       21.0000
## 3             k      880.0000
## 4      Deviance    -1859.9217
## 5           AIC    -1817.9217
## 6           BIC    -1717.7833
## 7          AICc    -1816.8320
```

```
### Create a new model fit data frame
newfit.mlmvrem4.sent <- data.frame(
  c("logLik", "parms", "k",
    "Deviance", 
    "AIC", "BIC", "AICc"),
  c(## logLik
    tas20.mlmvrem4.sent$fit.stats$REML[1],
    ## Number of parameters (parms)
    tas20.mlmvrem4.sent$parms,
    ## Number of effect sizes (k)
    length(tas20$Correlation),
    ## Deviance
    tas20.mlmvrem4.sent$fit.stats$REML[2],
    ## AIC
    tas20.mlmvrem4.sent$fit.stats$REML[3],
    ## BIC
    tas20.mlmvrem4.sent$fit.stats$REML[4],
    ## AICc
    tas20.mlmvrem4.sent$fit.stats$REML[5]))

colnames(newfit.mlmvrem4.sent) <- c("Fit criterion", "mlmvrem4.sent")
newfit.mlmvrem4.sent
```

```
##   Fit criterion mlmvrem4.sent
## 1        logLik      906.6167
## 2         parms       12.0000
## 3             k      880.0000
## 4      Deviance    -1813.2334
## 5           AIC    -1789.2334
## 6           BIC    -1732.0114
## 7          AICc    -1788.8693
```

```
## Overview of the model fit
newfit.overview.sent <- cbind.data.frame(newfit.mlmvrem1.sent,
                                    newfit.mlmvrem2.sent$mlmvrem2.sent,
                                    newfit.mlmvrem3.sent$mlmvrem3.sent,
                                    newfit.mlmvrem4.sent$mlmvrem4.sent)

colnames(newfit.overview.sent) <- c("Fit criterion",
                               "Model 1",
                               "Model 2",
                               "Model 3",
                               "Model 4")

newfit.overview.sent
```

```
##   Fit criterion    Model 1    Model 2    Model 3    Model 4
## 1        logLik   950.2943   938.0735   929.9608   906.6167
## 2         parms    30.0000    21.0000    21.0000    12.0000
## 3             k   880.0000   880.0000   880.0000   880.0000
## 4      Deviance -1900.5887 -1876.1470 -1859.9217 -1813.2334
## 5           AIC -1840.5887 -1834.1470 -1817.9217 -1789.2334
## 6           BIC -1697.5339 -1734.0087 -1717.7833 -1732.0114
## 7          AICc -1838.3718 -1833.0574 -1816.8320 -1788.8693
```

```
## Additional comparisons
anova(tas20.mlmvrem1.sent, tas20.mlmvrem2.sent)
```

```
## 
##         df        AIC        BIC       AICc   logLik     LRT   pval        QE 
## Full    30 -1840.5887 -1697.5339 -1838.3718 950.2943                6986.5768 
## Reduced 21 -1834.1470 -1734.0087 -1833.0574 938.0735 24.4417 0.0037 6986.5768
```

```
## Result: Model 1 is preferred over Model 2.

anova(tas20.mlmvrem1.sent, tas20.mlmvrem3.sent)
```

```
## 
##         df        AIC        BIC       AICc   logLik     LRT   pval        QE 
## Full    30 -1840.5887 -1697.5339 -1838.3718 950.2943                6986.5768 
## Reduced 21 -1817.9217 -1717.7833 -1816.8320 929.9608 40.6670 <.0001 6986.5768
```

```
## Result: Model 1 is preferred over Model 3.

anova(tas20.mlmvrem1.sent, tas20.mlmvrem4.sent)
```

```
## 
##         df        AIC        BIC       AICc   logLik     LRT   pval        QE 
## Full    30 -1840.5887 -1697.5339 -1838.3718 950.2943                6986.5768 
## Reduced 12 -1789.2334 -1732.0114 -1788.8693 906.6167 87.3553 <.0001 6986.5768
```

```
## Result: Model 1 is preferred over Model 4.
```

`Model 1` is favored over the other models (given the AIC
and outcomes of LRTs).

## Sensitivity Analyses

The model specification has many degrees of freedom, and
meta-analysts can represent effect size multiplicity at different
levels. Hence, we performed sensitivity analyses to examine the
influence of such choices on the meta-analytic findings. In the previous
analyses, we manipulated the values of \(rho\) and \(phi\) and we summarize the effects of this
manipulation here. Given that Models 1 and 2 were preferred over Models
3 and 4, we chose one of these models for illustrative purposes.

```
## Extract some findings from the sensitivity analyses
## Pooled correlation coefficients
SensAna.Corr <- data.frame(
  ## Row names
  c("I11-I12", "I11-I17", "I12-I17",
    "I2-I11", "I2-I12", "I2-I17",
    "I2-I4", "I4-I11", "I4-I12",
    "I4-I17"),
  ## Pooled correlations
  tas20.mlmvrem1$b,
  tas20.mlmvrem1.sens$b[,1],
  tas20.mlmvrem1.sent$b[,1]
)

colnames(SensAna.Corr) <- c("Correlation",
                              "rho=phi=0.0", 
                              "rho=phi=0.5",
                              "rho=phi=1.0")
rownames(SensAna.Corr) <- NULL
SensAna.Corr
```

```
##    Correlation rho=phi=0.0 rho=phi=0.5 rho=phi=1.0
## 1      I11-I12   0.3143325   0.3195034   0.3213418
## 2      I11-I17   0.3469820   0.3510389   0.3518189
## 3      I12-I17   0.2806570   0.2848330   0.2863937
## 4       I2-I11   0.4497150   0.4535519   0.4529675
## 5       I2-I12   0.3514609   0.3565983   0.3601982
## 6       I2-I17   0.3856644   0.3897407   0.3930211
## 7        I2-I4   0.4218548   0.4263690   0.4287027
## 8       I4-I11   0.3499278   0.3568501   0.3611199
## 9       I4-I12   0.2794383   0.2872768   0.2938799
## 10      I4-I17   0.3142220   0.3213674   0.3271352
```

```
## Save the results
##write.csv2(SensAna.Corr, file = "Sensitivity06-Corr.csv")

## Convert data to long format
SensAna.Corr.long <- reshape(SensAna.Corr,
                               direction = "long",
                               idvar = c("Correlation"),
                               varying = list(names(SensAna.Corr)[2:4]),
                               v.names = "Corr",
                               timevar = "RhoAndPhi",
                               times = c("0.0", "0.5", "1.0"))

rownames(SensAna.Corr.long) <- NULL
SensAna.Corr.long
```

```
##    Correlation RhoAndPhi      Corr
## 1      I11-I12       0.0 0.3143325
## 2      I11-I17       0.0 0.3469820
## 3      I12-I17       0.0 0.2806570
## 4       I2-I11       0.0 0.4497150
## 5       I2-I12       0.0 0.3514609
## 6       I2-I17       0.0 0.3856644
## 7        I2-I4       0.0 0.4218548
## 8       I4-I11       0.0 0.3499278
## 9       I4-I12       0.0 0.2794383
## 10      I4-I17       0.0 0.3142220
## 11     I11-I12       0.5 0.3195034
## 12     I11-I17       0.5 0.3510389
## 13     I12-I17       0.5 0.2848330
## 14      I2-I11       0.5 0.4535519
## 15      I2-I12       0.5 0.3565983
## 16      I2-I17       0.5 0.3897407
## 17       I2-I4       0.5 0.4263690
## 18      I4-I11       0.5 0.3568501
## 19      I4-I12       0.5 0.2872768
## 20      I4-I17       0.5 0.3213674
## 21     I11-I12       1.0 0.3213418
## 22     I11-I17       1.0 0.3518189
## 23     I12-I17       1.0 0.2863937
## 24      I2-I11       1.0 0.4529675
## 25      I2-I12       1.0 0.3601982
## 26      I2-I17       1.0 0.3930211
## 27       I2-I4       1.0 0.4287027
## 28      I4-I11       1.0 0.3611199
## 29      I4-I12       1.0 0.2938799
## 30      I4-I17       1.0 0.3271352
```

```
## Some xy plots
lattice::bwplot(Corr ~  RhoAndPhi | Correlation, 
                auto.key = TRUE, 
                data = SensAna.Corr.long,
                xlab = "Values of rho=phi",
                ylab = "Weighted average correlation",
                type = "a")
```

```
## Within-study heterogeneity variances
SensAna.VarW <- data.frame(
  ## Row names
  c("I11-I12", "I11-I17", "I12-I17",
    "I2-I11", "I2-I12", "I2-I17",
    "I2-I4", "I4-I11", "I4-I12",
    "I4-I17"),
  ## Within-study heterogeneity
  tas20.mlmvrem1$tau2,
  tas20.mlmvrem1.sens$tau2,
  tas20.mlmvrem1.sent$tau2
)

colnames(SensAna.VarW) <- c("Correlation",
                              "within_rho=phi=0.0", 
                              "within_rho=phi=0.5",
                              "within_rho=phi=1.0")
SensAna.VarW
```

```
##    Correlation within_rho=phi=0.0 within_rho=phi=0.5 within_rho=phi=1.0
## 1      I11-I12       0.0008363877       0.0004235517        0.001897843
## 2      I11-I17       0.0006175184       0.0003479348        0.001799434
## 3      I12-I17       0.0014580899       0.0006996448        0.001477171
## 4       I2-I11       0.0003506707       0.0001969019        0.001813669
## 5       I2-I12       0.0020222655       0.0011048613        0.001776235
## 6       I2-I17       0.0022487919       0.0013415759        0.002708108
## 7        I2-I4       0.0088424085       0.0055883271        0.008950128
## 8       I4-I11       0.0031856332       0.0017248898        0.003686697
## 9       I4-I12       0.0028289663       0.0019156835        0.004333034
## 10      I4-I17       0.0045084750       0.0024029993        0.004672614
```

```
## Save the results
##write.csv2(SensAna.VarW, file = "Sensitivity06-Var-Within.csv")


## Convert data to long format
SensAna.VarW.long <- reshape(SensAna.VarW,
                               direction = "long",
                               idvar = c("Correlation"),
                               varying = list(names(SensAna.VarW)[2:4]),
                               v.names = "VarW",
                               timevar = "RhoAndPhi",
                               times = c("0.0", "0.5", "1.0"))

rownames(SensAna.VarW.long) <- NULL
SensAna.VarW.long
```

```
##    Correlation RhoAndPhi         VarW
## 1      I11-I12       0.0 0.0008363877
## 2      I11-I17       0.0 0.0006175184
## 3      I12-I17       0.0 0.0014580899
## 4       I2-I11       0.0 0.0003506707
## 5       I2-I12       0.0 0.0020222655
## 6       I2-I17       0.0 0.0022487919
## 7        I2-I4       0.0 0.0088424085
## 8       I4-I11       0.0 0.0031856332
## 9       I4-I12       0.0 0.0028289663
## 10      I4-I17       0.0 0.0045084750
## 11     I11-I12       0.5 0.0004235517
## 12     I11-I17       0.5 0.0003479348
## 13     I12-I17       0.5 0.0006996448
## 14      I2-I11       0.5 0.0001969019
## 15      I2-I12       0.5 0.0011048613
## 16      I2-I17       0.5 0.0013415759
## 17       I2-I4       0.5 0.0055883271
## 18      I4-I11       0.5 0.0017248898
## 19      I4-I12       0.5 0.0019156835
## 20      I4-I17       0.5 0.0024029993
## 21     I11-I12       1.0 0.0018978428
## 22     I11-I17       1.0 0.0017994336
## 23     I12-I17       1.0 0.0014771712
## 24      I2-I11       1.0 0.0018136687
## 25      I2-I12       1.0 0.0017762354
## 26      I2-I17       1.0 0.0027081084
## 27       I2-I4       1.0 0.0089501284
## 28      I4-I11       1.0 0.0036866973
## 29      I4-I12       1.0 0.0043330341
## 30      I4-I17       1.0 0.0046726143
```

```
## Some xy plots
lattice::bwplot(VarW ~  RhoAndPhi | Correlation, 
                auto.key = TRUE, 
                data = SensAna.VarW.long,
                xlab = "Values of rho=phi",
                ylab = "Within-study variance",
                type = "a")
```

```
## Heterogeneity variances
SensAna.VarB <- data.frame(
  ## Row names
  c("I11-I12", "I11-I17", "I12-I17",
    "I2-I11", "I2-I12", "I2-I17",
    "I2-I4", "I4-I11", "I4-I12",
    "I4-I17"),
  ## Between-study heterogeneity
  tas20.mlmvrem1$gamma2,
  tas20.mlmvrem1.sens$gamma2,
  tas20.mlmvrem1.sent$gamma2
)

colnames(SensAna.VarB) <- c("Correlation",
                              "between_rho=phi=0.0", 
                              "between_rho=phi=0.5",
                              "between_rho=phi=1.0")
SensAna.VarB
```

```
##    Correlation between_rho=phi=0.0 between_rho=phi=0.5 between_rho=phi=1.0
## 1      I11-I12         0.004874803         0.004688239         0.008097956
## 2      I11-I17         0.004919181         0.004530343         0.007317264
## 3      I12-I17         0.006567701         0.006153293         0.011839405
## 4       I2-I11         0.003924894         0.003560545         0.005157548
## 5       I2-I12         0.005017425         0.005029490         0.009906682
## 6       I2-I17         0.005157625         0.004879977         0.008325833
## 7        I2-I4         0.010190395         0.009828553         0.013370863
## 8       I4-I11         0.007309420         0.006810799         0.010513693
## 9       I4-I12         0.009920981         0.008227479         0.012722459
## 10      I4-I17         0.008459115         0.008123887         0.013593433
```

```
## Save the results
##write.csv2(SensAna.VarB, file = "Sensitivity06-Var-Between.csv")


## Convert data to long format
SensAna.VarB.long <- reshape(SensAna.VarB,
                               direction = "long",
                               idvar = c("Correlation"),
                               varying = list(names(SensAna.VarB)[2:4]),
                               v.names = "VarB",
                               timevar = "RhoAndPhi",
                               times = c("0.0", "0.5", "1.0"))

rownames(SensAna.VarB.long) <- NULL
SensAna.VarB.long
```

```
##    Correlation RhoAndPhi        VarB
## 1      I11-I12       0.0 0.004874803
## 2      I11-I17       0.0 0.004919181
## 3      I12-I17       0.0 0.006567701
## 4       I2-I11       0.0 0.003924894
## 5       I2-I12       0.0 0.005017425
## 6       I2-I17       0.0 0.005157625
## 7        I2-I4       0.0 0.010190395
## 8       I4-I11       0.0 0.007309420
## 9       I4-I12       0.0 0.009920981
## 10      I4-I17       0.0 0.008459115
## 11     I11-I12       0.5 0.004688239
## 12     I11-I17       0.5 0.004530343
## 13     I12-I17       0.5 0.006153293
## 14      I2-I11       0.5 0.003560545
## 15      I2-I12       0.5 0.005029490
## 16      I2-I17       0.5 0.004879977
## 17       I2-I4       0.5 0.009828553
## 18      I4-I11       0.5 0.006810799
## 19      I4-I12       0.5 0.008227479
## 20      I4-I17       0.5 0.008123887
## 21     I11-I12       1.0 0.008097956
## 22     I11-I17       1.0 0.007317264
## 23     I12-I17       1.0 0.011839405
## 24      I2-I11       1.0 0.005157548
## 25      I2-I12       1.0 0.009906682
## 26      I2-I17       1.0 0.008325833
## 27       I2-I4       1.0 0.013370863
## 28      I4-I11       1.0 0.010513693
## 29      I4-I12       1.0 0.012722459
## 30      I4-I17       1.0 0.013593433
```

```
## Some xy plots
lattice::bwplot(VarB ~  RhoAndPhi | Correlation, 
                auto.key = TRUE, 
                data = SensAna.VarB.long,
                xlab = "Values of rho=phi",
                ylab = "Between-study variance",
                type = "a")
```

## Multivariate Random-Effects Approaches Ignoring the Hierarchical Effect Size Multiplicity

The following approaches represent multivariate random-effects models
that do not consider the nesting of multiple correlation coefficients in
study samples or primary studies. However, the heterogeneity variances
can still be correlation-specific.

### Multivariate Random-Effects Models with an overall estimate of heterogeneity

```
# No-intercept random-effects model

## Model specification
tas20.mvrem <- rma.mv(yi = Correlation,
                      V = V, 
                      data = tas20,
                      random = list(~ 1 | ESID),
                      method = "REML",
                      mods = ~ factor(Cell) - 1,
                      time = TRUE,
                      sparse = TRUE,
                      control = list(optimizer = "optimParallel",
                                     ncpus = ncores))
```

```
## 
## Processing time: 0 hours, 0 minutes, 7 seconds
```

```
## Model summary
summary(tas20.mvrem)
```

```
## 
## Multivariate Meta-Analysis Model (k = 880; method: REML)
## 
##     logLik    Deviance         AIC         BIC        AICc   
##   656.6757  -1313.3513  -1291.3513  -1238.8979  -1291.0436   
## 
## Variance Components:
## 
##             estim    sqrt  nlvls  fixed  factor 
## sigma^2    0.0086  0.0927    880     no    ESID 
## 
## Test for Residual Heterogeneity:
## QE(df = 870) = 6986.5768, p-val < .0001
## 
## Test of Moderators (coefficients 1:10):
## QM(df = 10) = 7754.6283, p-val < .0001
## 
## Model Results:
## 
##                      estimate      se     zval    pval   ci.lb   ci.ub      
## factor(Cell)I11-I12    0.3123  0.0111  28.0990  <.0001  0.2905  0.3341  *** 
## factor(Cell)I11-I17    0.3452  0.0110  31.2453  <.0001  0.3236  0.3669  *** 
## factor(Cell)I12-I17    0.2803  0.0111  25.1525  <.0001  0.2584  0.3021  *** 
## factor(Cell)I2-I11     0.4444  0.0109  40.8214  <.0001  0.4231  0.4658  *** 
## factor(Cell)I2-I12     0.3509  0.0110  31.7635  <.0001  0.3292  0.3725  *** 
## factor(Cell)I2-I17     0.3871  0.0110  35.3020  <.0001  0.3656  0.4086  *** 
## factor(Cell)I2-I4      0.4263  0.0109  39.1402  <.0001  0.4050  0.4477  *** 
## factor(Cell)I4-I11     0.3540  0.0110  32.0891  <.0001  0.3324  0.3756  *** 
## factor(Cell)I4-I12     0.2851  0.0111  25.6114  <.0001  0.2633  0.3070  *** 
## factor(Cell)I4-I17     0.3207  0.0111  28.9660  <.0001  0.2990  0.3424  *** 
## 
## ---
## Signif. codes:  0 '***' 0.001 '**' 0.01 '*' 0.05 '.' 0.1 ' ' 1
```

```
## Processing time in seconds
tas20.mvrem$time
```

```
## [1] 7.002
```

```
## Extract relevant elements
## Asymptotic covariance matrix
ACOV.mvrem <- tas20.mvrem$vb

## Pooled correlation matrix
CORR.mvrem <- vec2symMat(x = c(1, tas20.mvrem$b[7], tas20.mvrem$b[4], tas20.mvrem$b[5], tas20.mvrem$b[6],
                                1, tas20.mvrem$b[8], tas20.mvrem$b[9], tas20.mvrem$b[10],
                                1, tas20.mvrem$b[1], tas20.mvrem$b[2],
                                1, tas20.mvrem$b[3],
                                1),
                          diag = TRUE)

colnames(CORR.mvrem) <- c("Item2", "Item4", "Item11", "Item12", "Item17")
rownames(CORR.mvrem) <- colnames(CORR.mvrem)

## Inspect the pooled correlation matrix
CORR.mvrem
```

```
##            Item2     Item4    Item11    Item12    Item17
## Item2  1.0000000 0.4263242 0.4444247 0.3508852 0.3870954
## Item4  0.4263242 1.0000000 0.3540091 0.2851343 0.3206589
## Item11 0.4444247 0.3540091 1.0000000 0.3122949 0.3452388
## Item12 0.3508852 0.2851343 0.3122949 1.0000000 0.2802898
## Item17 0.3870954 0.3206589 0.3452388 0.2802898 1.0000000
```

```
## Plot the pooled correlation matrix
corrplot(CORR.mvrem, 
         type = "upper", 
         order = "original", 
         tl.col = "black", 
         tl.srt = 60,
         addCoef.col = "black",
         number.cex = 0.9,
         cl.cex = 1,
         tl.cex = 1)
```

```
## Alternative model specification
tas20.mvrem2 <- rma.mv(yi = Correlation,
                       V = V, 
                       data = tas20,
                       random = list(~ factor(Cell) | ESID),
                       method = "REML",
                       mods = ~ factor(Cell) - 1,
                       struc = "CS",
                       rho = 0,
                       time = TRUE,
                       sparse = TRUE,
                       control = list(optimizer = "optimParallel", 
                                      ncpus = ncores))
```

```
## 
## Processing time: 0 hours, 0 minutes, 7.29 seconds
```

```
## Model summary
summary(tas20.mvrem2)
```

```
## 
## Multivariate Meta-Analysis Model (k = 880; method: REML)
## 
##     logLik    Deviance         AIC         BIC        AICc   
##   656.6757  -1313.3513  -1291.3513  -1238.8979  -1291.0436   
## 
## Variance Components:
## 
## outer factor: ESID         (nlvls = 880)
## inner factor: factor(Cell) (nlvls = 10)
## 
##             estim    sqrt  fixed 
## tau^2      0.0086  0.0927     no 
## rho        0.0000            yes 
## 
## Test for Residual Heterogeneity:
## QE(df = 870) = 6986.5768, p-val < .0001
## 
## Test of Moderators (coefficients 1:10):
## QM(df = 10) = 7754.6283, p-val < .0001
## 
## Model Results:
## 
##                      estimate      se     zval    pval   ci.lb   ci.ub      
## factor(Cell)I11-I12    0.3123  0.0111  28.0990  <.0001  0.2905  0.3341  *** 
## factor(Cell)I11-I17    0.3452  0.0110  31.2453  <.0001  0.3236  0.3669  *** 
## factor(Cell)I12-I17    0.2803  0.0111  25.1525  <.0001  0.2584  0.3021  *** 
## factor(Cell)I2-I11     0.4444  0.0109  40.8214  <.0001  0.4231  0.4658  *** 
## factor(Cell)I2-I12     0.3509  0.0110  31.7635  <.0001  0.3292  0.3725  *** 
## factor(Cell)I2-I17     0.3871  0.0110  35.3020  <.0001  0.3656  0.4086  *** 
## factor(Cell)I2-I4      0.4263  0.0109  39.1402  <.0001  0.4050  0.4477  *** 
## factor(Cell)I4-I11     0.3540  0.0110  32.0891  <.0001  0.3324  0.3756  *** 
## factor(Cell)I4-I12     0.2851  0.0111  25.6114  <.0001  0.2633  0.3070  *** 
## factor(Cell)I4-I17     0.3207  0.0111  28.9660  <.0001  0.2990  0.3424  *** 
## 
## ---
## Signif. codes:  0 '***' 0.001 '**' 0.01 '*' 0.05 '.' 0.1 ' ' 1
```

### Multivariate Random-Effects Models with correlation-specific estimates of heterogeneity

```
## Model specification
tas20.mvrem3 <- rma.mv(yi = Correlation,
                       V = V, 
                       data = tas20,
                       random = list(~ factor(Cell) | ESID),
                       method = "REML",
                       mods = ~ factor(Cell) - 1,
                       struc = "HCS",
                       rho = 0,
                       time = TRUE,
                       sparse = TRUE,
                       control = list(optimizer = "optimParallel", 
                                      ncpus = ncores))
```

```
## 
## Processing time: 0 hours, 0 minutes, 6.31 seconds
```

```
## Model summary
summary(tas20.mvrem3)
```

```
## 
## Multivariate Meta-Analysis Model (k = 880; method: REML)
## 
##     logLik    Deviance         AIC         BIC        AICc   
##   683.6622  -1367.3243  -1327.3243  -1231.9545  -1326.3349   
## 
## Variance Components:
## 
## outer factor: ESID         (nlvls = 880)
## inner factor: factor(Cell) (nlvls = 10)
## 
##              estim    sqrt  k.lvl  fixed    level 
## tau^2.1     0.0053  0.0727     88     no  I11-I12 
## tau^2.2     0.0049  0.0699     88     no  I11-I17 
## tau^2.3     0.0069  0.0830     88     no  I12-I17 
## tau^2.4     0.0042  0.0648     88     no   I2-I11 
## tau^2.5     0.0064  0.0801     88     no   I2-I12 
## tau^2.6     0.0070  0.0836     88     no   I2-I17 
## tau^2.7     0.0178  0.1335     88     no    I2-I4 
## tau^2.8     0.0095  0.0974     88     no   I4-I11 
## tau^2.9     0.0111  0.1054     88     no   I4-I12 
## tau^2.10    0.0124  0.1113     88     no   I4-I17 
## rho         0.0000                   yes          
## 
## Test for Residual Heterogeneity:
## QE(df = 870) = 6986.5768, p-val < .0001
## 
## Test of Moderators (coefficients 1:10):
## QM(df = 10) = 8815.6271, p-val < .0001
## 
## Model Results:
## 
##                      estimate      se     zval    pval   ci.lb   ci.ub      
## factor(Cell)I11-I12    0.3136  0.0092  34.1091  <.0001  0.2956  0.3317  *** 
## factor(Cell)I11-I17    0.3463  0.0089  39.0526  <.0001  0.3289  0.3637  *** 
## factor(Cell)I12-I17    0.2809  0.0102  27.5296  <.0001  0.2609  0.3009  *** 
## factor(Cell)I2-I11     0.4469  0.0082  54.5330  <.0001  0.4308  0.4630  *** 
## factor(Cell)I2-I12     0.3523  0.0098  35.8405  <.0001  0.3330  0.3716  *** 
## factor(Cell)I2-I17     0.3880  0.0101  38.5100  <.0001  0.3683  0.4078  *** 
## factor(Cell)I2-I4      0.4229  0.0150  28.2253  <.0001  0.3936  0.4523  *** 
## factor(Cell)I4-I11     0.3527  0.0115  30.7256  <.0001  0.3302  0.3752  *** 
## factor(Cell)I4-I12     0.2840  0.0124  22.9684  <.0001  0.2597  0.3082  *** 
## factor(Cell)I4-I17     0.3190  0.0129  24.7350  <.0001  0.2937  0.3443  *** 
## 
## ---
## Signif. codes:  0 '***' 0.001 '**' 0.01 '*' 0.05 '.' 0.1 ' ' 1
```

```
## Processing time in seconds
tas20.mvrem3$time
```

```
## [1] 6.305
```

```
## Extract relevant elements
## Asymptotic covariance matrix
ACOV.mvrem3 <- tas20.mvrem3$vb

## Pooled correlation matrix
CORR.mvrem3 <- vec2symMat(x = c(1, tas20.mvrem3$b[7], tas20.mvrem3$b[4], tas20.mvrem3$b[5], tas20.mvrem3$b[6],
                                1, tas20.mvrem3$b[8], tas20.mvrem3$b[9], tas20.mvrem3$b[10],
                                1, tas20.mvrem3$b[1], tas20.mvrem3$b[2],
                                1, tas20.mvrem3$b[3],
                                1),
                          diag = TRUE)

colnames(CORR.mvrem3) <- c("Item2", "Item4", "Item11", "Item12", "Item17")
rownames(CORR.mvrem3) <- colnames(CORR.mvrem3)

## Inspect the pooled correlation matrix
CORR.mvrem3
```

```
##            Item2     Item4    Item11    Item12    Item17
## Item2  1.0000000 0.4229206 0.4468901 0.3523001 0.3880359
## Item4  0.4229206 1.0000000 0.3527367 0.2839536 0.3190045
## Item11 0.4468901 0.3527367 1.0000000 0.3136353 0.3462934
## Item12 0.3523001 0.2839536 0.3136353 1.0000000 0.2808636
## Item17 0.3880359 0.3190045 0.3462934 0.2808636 1.0000000
```

```
## Plot the pooled correlation matrix
corrplot(CORR.mvrem3, 
         type = "upper", 
         order = "original", 
         tl.col = "black", 
         tl.srt = 60,
         addCoef.col = "black",
         number.cex = 0.9,
         cl.cex = 1,
         tl.cex = 1)
```

The first stage of the two-stage structural equation modeling (TSSEM;
see Cheung,
2015 and Harrer
et al., 2021) approach estimates a multivariate random-effects model
with correlation-specific heterogeneity estimates. This step can pool
correlation matrices via a multivariate random-effects model with
maximum-likelihood estimation.

```
## Check for positive definiteness
is.pd(cmatnew)
```

```
##  [1] TRUE TRUE TRUE TRUE TRUE TRUE TRUE TRUE TRUE TRUE TRUE TRUE TRUE TRUE TRUE
## [16] TRUE TRUE TRUE TRUE TRUE TRUE TRUE TRUE TRUE TRUE TRUE TRUE TRUE TRUE TRUE
## [31] TRUE TRUE TRUE TRUE TRUE TRUE TRUE TRUE TRUE TRUE TRUE TRUE TRUE TRUE TRUE
## [46] TRUE TRUE TRUE TRUE TRUE TRUE TRUE TRUE TRUE TRUE TRUE TRUE TRUE TRUE TRUE
## [61] TRUE TRUE TRUE TRUE TRUE TRUE TRUE TRUE TRUE TRUE TRUE TRUE TRUE TRUE TRUE
## [76] TRUE TRUE TRUE TRUE TRUE TRUE TRUE TRUE TRUE TRUE TRUE TRUE TRUE
```

```
# Pooling correlation matrices using the REM option
## Note: This step may take several hours.
tas20.tssem <- tssem1(cmatnew, dat$n, 
                      method = "REM", 
                      RE.type = "Diag", 
                      I2 = "I2q") 

# Rerun the model to obtain more robust results
tas20.tssem <- rerun(tas20.tssem)
```

```
## Running TSSEM1 Correlation with 20 parameters
```

```
## 
## Beginning initial fit attempt
```

```
## Running TSSEM1 Correlation with 20 parameters
```

```
## 
##  Lowest minimum so far:  -1446.37306551261
```

```
## 
## Solution found
```

```
## 
##  Solution found!  Final fit=-1446.3731 (started at -1446.3731)  (1 attempt(s): 1 valid, 0 errors)
```

```
# Summarize the results
summary(tas20.tssem)
```

```
## 
## Call:
## meta(y = ES, v = acovR, RE.constraints = Diag(paste0(RE.startvalues, 
##     "*Tau2_", 1:no.es, "_", 1:no.es)), RE.lbound = RE.lbound, 
##     I2 = I2, model.name = model.name, suppressWarnings = TRUE, 
##     silent = silent, run = run)
## 
## 95% confidence intervals: z statistic approximation (robust=FALSE)
## Coefficients:
##               Estimate  Std.Error     lbound     ubound z value  Pr(>|z|)    
## Intercept1  0.41884772 0.01434415 0.39073370 0.44696175 29.1999 < 2.2e-16 ***
## Intercept2  0.44637354 0.00779063 0.43110417 0.46164290 57.2962 < 2.2e-16 ***
## Intercept3  0.35260069 0.00929534 0.33438215 0.37081923 37.9330 < 2.2e-16 ***
## Intercept4  0.38775363 0.00966505 0.36881048 0.40669679 40.1191 < 2.2e-16 ***
## Intercept5  0.34856686 0.01080521 0.32738904 0.36974468 32.2591 < 2.2e-16 ***
## Intercept6  0.28311849 0.01173912 0.26011024 0.30612674 24.1175 < 2.2e-16 ***
## Intercept7  0.31532620 0.01226240 0.29129234 0.33936006 25.7149 < 2.2e-16 ***
## Intercept8  0.31477690 0.00874341 0.29764012 0.33191367 36.0016 < 2.2e-16 ***
## Intercept9  0.34557706 0.00862486 0.32867265 0.36248147 40.0676 < 2.2e-16 ***
## Intercept10 0.28069132 0.00969995 0.26167976 0.29970288 28.9374 < 2.2e-16 ***
## Tau2_1_1    0.01597720 0.00272157 0.01064302 0.02131138  5.8706 4.343e-09 ***
## Tau2_2_2    0.00367252 0.00077069 0.00216200 0.00518305  4.7652 1.886e-06 ***
## Tau2_3_3    0.00556031 0.00103550 0.00353078 0.00758985  5.3697 7.886e-08 ***
## Tau2_4_4    0.00621296 0.00117627 0.00390752 0.00851841  5.2819 1.278e-07 ***
## Tau2_5_5    0.00800258 0.00152484 0.00501396 0.01099121  5.2482 1.536e-07 ***
## Tau2_6_6    0.00963816 0.00172494 0.00625733 0.01301899  5.5875 2.303e-08 ***
## Tau2_7_7    0.01078974 0.00194629 0.00697508 0.01460441  5.5437 2.961e-08 ***
## Tau2_8_8    0.00459676 0.00090938 0.00281441 0.00637911  5.0548 4.308e-07 ***
## Tau2_9_9    0.00447601 0.00092398 0.00266504 0.00628697  4.8443 1.271e-06 ***
## Tau2_10_10  0.00595187 0.00114679 0.00370421 0.00819953  5.1900 2.103e-07 ***
## ---
## Signif. codes:  0 '***' 0.001 '**' 0.01 '*' 0.05 '.' 0.1 ' ' 1
## 
## Q statistic on the homogeneity of effect sizes: 6317.555
## Degrees of freedom of the Q statistic: 870
## P value of the Q statistic: 0
## 
## Heterogeneity indices (based on the estimated Tau2):
##                               Estimate
## Intercept1: I2 (Q statistic)    0.9453
## Intercept2: I2 (Q statistic)    0.8213
## Intercept3: I2 (Q statistic)    0.8523
## Intercept4: I2 (Q statistic)    0.8700
## Intercept5: I2 (Q statistic)    0.8852
## Intercept6: I2 (Q statistic)    0.8953
## Intercept7: I2 (Q statistic)    0.9081
## Intercept8: I2 (Q statistic)    0.8171
## Intercept9: I2 (Q statistic)    0.8182
## Intercept10: I2 (Q statistic)   0.8446
## 
## Number of studies (or clusters): 88
## Number of observed statistics: 880
## Number of estimated parameters: 20
## Degrees of freedom: 860
## -2 log likelihood: -1446.373 
## OpenMx status1: 0 ("0" or "1": The optimization is considered fine.
## Other values may indicate problems.)
```

```
# Extract the resultant correlation matrix
CORR.tssem <- vec2symMat(coef(tas20.tssem, select="fixed"), diag=FALSE)
colnames(CORR.tssem) <- colnames(CORR.mvrem)
rownames(CORR.tssem) <- colnames(CORR.mvrem)
CORR.tssem
```

```
##            Item2     Item4    Item11    Item12    Item17
## Item2  1.0000000 0.4188477 0.4463735 0.3526007 0.3877536
## Item4  0.4188477 1.0000000 0.3485669 0.2831185 0.3153262
## Item11 0.4463735 0.3485669 1.0000000 0.3147769 0.3455771
## Item12 0.3526007 0.2831185 0.3147769 1.0000000 0.2806913
## Item17 0.3877536 0.3153262 0.3455771 0.2806913 1.0000000
```

```
# Correlogram
corrplot(CORR.tssem, 
         type = "upper", 
         order = "original", 
         tl.col = "black", 
         tl.srt = 60,
         addCoef.col = "black",
         number.cex = 0.9,
         cl.cex = 1,
         tl.cex = 1)
```

```
## For comparison: MVREM with ML estimation
## Model specification
tas20.mvrem.ml <- rma.mv(yi = Correlation, 
                         V = V, 
                         data = tas20,
                         random = list(~ factor(Cell) | ESID),
                         method = "ML",
                         mods = ~ factor(Cell) - 1,
                         struc = "HCS",
                         rho = 0,
                         time = TRUE,
                         sparse = TRUE,
                         control = list(optimizer = "optimParallel", 
                                        ncpus = ncores))
```

```
## 
## Processing time: 0 hours, 0 minutes, 5.2 seconds
```

```
## Model summary
summary(tas20.mvrem.ml)
```

```
## 
## Multivariate Meta-Analysis Model (k = 880; method: ML)
## 
##     logLik    Deviance         AIC         BIC        AICc   
##   697.6039   2774.4289  -1355.2079  -1259.6094  -1354.2300   
## 
## Variance Components:
## 
## outer factor: ESID         (nlvls = 880)
## inner factor: factor(Cell) (nlvls = 10)
## 
##              estim    sqrt  k.lvl  fixed    level 
## tau^2.1     0.0052  0.0719     88     no  I11-I12 
## tau^2.2     0.0048  0.0691     88     no  I11-I17 
## tau^2.3     0.0067  0.0821     88     no  I12-I17 
## tau^2.4     0.0041  0.0641     88     no   I2-I11 
## tau^2.5     0.0063  0.0794     88     no   I2-I12 
## tau^2.6     0.0069  0.0828     88     no   I2-I17 
## tau^2.7     0.0175  0.1325     88     no    I2-I4 
## tau^2.8     0.0093  0.0964     88     no   I4-I11 
## tau^2.9     0.0109  0.1045     88     no   I4-I12 
## tau^2.10    0.0122  0.1103     88     no   I4-I17 
## rho         0.0000                   yes          
## 
## Test for Residual Heterogeneity:
## QE(df = 870) = 6986.5768, p-val < .0001
## 
## Test of Moderators (coefficients 1:10):
## QM(df = 10) = 8924.9018, p-val < .0001
## 
## Model Results:
## 
##                      estimate      se     zval    pval   ci.lb   ci.ub      
## factor(Cell)I11-I12    0.3136  0.0091  34.3623  <.0001  0.2957  0.3315  *** 
## factor(Cell)I11-I17    0.3463  0.0088  39.3798  <.0001  0.3290  0.3635  *** 
## factor(Cell)I12-I17    0.2808  0.0101  27.7408  <.0001  0.2610  0.3007  *** 
## factor(Cell)I2-I11     0.4469  0.0081  54.9636  <.0001  0.4310  0.4628  *** 
## factor(Cell)I2-I12     0.3523  0.0098  36.1038  <.0001  0.3332  0.3714  *** 
## factor(Cell)I2-I17     0.3880  0.0100  38.8144  <.0001  0.3684  0.4076  *** 
## factor(Cell)I2-I4      0.4230  0.0149  28.4344  <.0001  0.3938  0.4521  *** 
## factor(Cell)I4-I11     0.3528  0.0114  30.9729  <.0001  0.3304  0.3751  *** 
## factor(Cell)I4-I12     0.2839  0.0123  23.1333  <.0001  0.2599  0.3080  *** 
## factor(Cell)I4-I17     0.3190  0.0128  24.9262  <.0001  0.2939  0.3441  *** 
## 
## ---
## Signif. codes:  0 '***' 0.001 '**' 0.01 '*' 0.05 '.' 0.1 ' ' 1
```

```
## Processing time in seconds
tas20.mvrem.ml$time
```

```
## [1] 5.199
```

```
## Extract relevant elements
## Asymptotic covariance matrix
ACOV.mvrem.ml <- tas20.mvrem.ml$vb

## Pooled correlation matrix
CORR.mvrem.ml <- vec2symMat(x = c(1, tas20.mvrem.ml$b[7], tas20.mvrem.ml$b[4], tas20.mvrem.ml$b[5], tas20.mvrem.ml$b[6],
                                1, tas20.mvrem.ml$b[8], tas20.mvrem.ml$b[9], tas20.mvrem.ml$b[10],
                                1, tas20.mvrem.ml$b[1], tas20.mvrem.ml$b[2],
                                1, tas20.mvrem.ml$b[3],
                                1),
                          diag = TRUE)

colnames(CORR.mvrem.ml) <- c("Item2", "Item4", "Item11", "Item12", "Item17")
rownames(CORR.mvrem.ml) <- colnames(CORR.mvrem.ml)

## Inspect the pooled correlation matrix
CORR.mvrem.ml
```

```
##            Item2     Item4    Item11    Item12    Item17
## Item2  1.0000000 0.4229529 0.4468923 0.3522924 0.3879938
## Item4  0.4229529 1.0000000 0.3527706 0.2839499 0.3190143
## Item11 0.4468923 0.3527706 1.0000000 0.3136152 0.3462551
## Item12 0.3522924 0.2839499 0.3136152 1.0000000 0.2808145
## Item17 0.3879938 0.3190143 0.3462551 0.2808145 1.0000000
```

```
## Plot the pooled correlation matrix
corrplot(CORR.mvrem.ml, 
         type = "upper", 
         order = "original", 
         tl.col = "black", 
         tl.srt = 60,
         addCoef.col = "black",
         number.cex = 0.9,
         cl.cex = 1,
         tl.cex = 1)
```

# Moderator Analyses

In the following section, we show how moderator effects can be
estimated for each of the types of correlations. We base the following
analyses on the model `mlmvrem2`.

## Moderation by a categorical variable (type of the sample)

```
## Description of the moderator
table(tas20$Clinical)
```

```
## 
##   0   1   2 
## 660 200  20
```

```
## Codes: 0 = Non-clinical, 1 = Clinical, 2 = Mixed

## Recode the variable
tas20$ClinicalBinary <- NA
tas20$ClinicalBinary[tas20$Clinical == 0] <- 0
tas20$ClinicalBinary[tas20$Clinical == 1] <- 1
tas20$ClinicalBinary[tas20$Clinical == 2] <- 1
table(tas20$ClinicalBinary)
```

```
## 
##   0   1 
## 660 220
```

```
## Model that tests for moderator effects
## Model specification
tas20.clinical <- rma.mv(yi = Correlation,
                         V = V, 
                         data = tas20,
                         random = list(~ factor(Cell) | ESID,
                                       ~ factor(Cell) | StudyID),
                         struc = c("HCS", "CS"),
                         rho = 0,
                         phi = 0,
                         method = "REML",
                         mods = ~ factor(Cell) - 1 + factor(Cell):factor(ClinicalBinary),
                         time = TRUE,
                         sparse = TRUE,
                         control = list(optimizer = "optimParallel",
                                        ncpus = ncores))
```

```
## 
## Processing time: 0 hours, 0 minutes, 11.6 seconds
```

```
## Model summary
summary(tas20.clinical)
```

```
## 
## Multivariate Meta-Analysis Model (k = 880; method: REML)
## 
##     logLik    Deviance         AIC         BIC        AICc   
##   720.5345  -1441.0690  -1379.0690  -1231.6041  -1376.6729   
## 
## Variance Components:
## 
## outer factor: ESID         (nlvls = 880)
## inner factor: factor(Cell) (nlvls = 10)
## 
##              estim    sqrt  k.lvl  fixed    level 
## tau^2.1     0.0009  0.0307     88     no  I11-I12 
## tau^2.2     0.0007  0.0268     88     no  I11-I17 
## tau^2.3     0.0020  0.0447     88     no  I12-I17 
## tau^2.4     0.0004  0.0200     88     no   I2-I11 
## tau^2.5     0.0022  0.0470     88     no   I2-I12 
## tau^2.6     0.0025  0.0495     88     no   I2-I17 
## tau^2.7     0.0116  0.1075     88     no    I2-I4 
## tau^2.8     0.0040  0.0630     88     no   I4-I11 
## tau^2.9     0.0053  0.0727     88     no   I4-I12 
## tau^2.10    0.0061  0.0779     88     no   I4-I17 
## rho         0.0000                   yes          
## 
## outer factor: StudyID      (nlvls = 62)
## inner factor: factor(Cell) (nlvls = 10)
## 
##             estim    sqrt  fixed 
## gamma^2    0.0050  0.0708     no 
## phi        0.0000            yes 
## 
## Test for Residual Heterogeneity:
## QE(df = 860) = 6847.4154, p-val < .0001
## 
## Test of Moderators (coefficients 1:20):
## QM(df = 20) = 6792.2177, p-val < .0001
## 
## Model Results:
## 
##                                              estimate      se     zval    pval 
## factor(Cell)I11-I12                            0.3197  0.0120  26.6801  <.0001 
## factor(Cell)I11-I17                            0.3510  0.0117  29.9672  <.0001 
## factor(Cell)I12-I17                            0.2843  0.0128  22.1769  <.0001 
## factor(Cell)I2-I11                             0.4546  0.0112  40.6692  <.0001 
## factor(Cell)I2-I12                             0.3539  0.0128  27.5584  <.0001 
## factor(Cell)I2-I17                             0.3896  0.0129  30.1740  <.0001 
## factor(Cell)I2-I4                              0.4434  0.0177  25.1162  <.0001 
## factor(Cell)I4-I11                             0.3694  0.0139  26.5508  <.0001 
## factor(Cell)I4-I12                             0.2901  0.0148  19.5701  <.0001 
## factor(Cell)I4-I17                             0.3316  0.0152  21.8398  <.0001 
## factor(Cell)I11-I12:factor(ClinicalBinary)1   -0.0222  0.0184  -1.2079  0.2271 
## factor(Cell)I11-I17:factor(ClinicalBinary)1   -0.0163  0.0174  -0.9351  0.3498 
## factor(Cell)I12-I17:factor(ClinicalBinary)1   -0.0145  0.0207  -0.7012  0.4832 
## factor(Cell)I2-I11:factor(ClinicalBinary)1    -0.0214  0.0157  -1.3686  0.1711 
## factor(Cell)I2-I12:factor(ClinicalBinary)1    -0.0107  0.0208  -0.5162  0.6057 
## factor(Cell)I2-I17:factor(ClinicalBinary)1    -0.0152  0.0209  -0.7286  0.4663 
## factor(Cell)I2-I4:factor(ClinicalBinary)1     -0.0835  0.0324  -2.5751  0.0100 
## factor(Cell)I4-I11:factor(ClinicalBinary)1    -0.0745  0.0238  -3.1240  0.0018 
## factor(Cell)I4-I12:factor(ClinicalBinary)1    -0.0377  0.0259  -1.4561  0.1454 
## factor(Cell)I4-I17:factor(ClinicalBinary)1    -0.0640  0.0267  -2.3952  0.0166 
##                                                ci.lb    ci.ub      
## factor(Cell)I11-I12                           0.2962   0.3432  *** 
## factor(Cell)I11-I17                           0.3281   0.3740  *** 
## factor(Cell)I12-I17                           0.2591   0.3094  *** 
## factor(Cell)I2-I11                            0.4327   0.4765  *** 
## factor(Cell)I2-I12                            0.3288   0.3791  *** 
## factor(Cell)I2-I17                            0.3643   0.4149  *** 
## factor(Cell)I2-I4                             0.4088   0.4780  *** 
## factor(Cell)I4-I11                            0.3421   0.3967  *** 
## factor(Cell)I4-I12                            0.2610   0.3191  *** 
## factor(Cell)I4-I17                            0.3018   0.3613  *** 
## factor(Cell)I11-I12:factor(ClinicalBinary)1  -0.0582   0.0138      
## factor(Cell)I11-I17:factor(ClinicalBinary)1  -0.0505   0.0179      
## factor(Cell)I12-I17:factor(ClinicalBinary)1  -0.0552   0.0261      
## factor(Cell)I2-I11:factor(ClinicalBinary)1   -0.0521   0.0093      
## factor(Cell)I2-I12:factor(ClinicalBinary)1   -0.0515   0.0300      
## factor(Cell)I2-I17:factor(ClinicalBinary)1   -0.0562   0.0257      
## factor(Cell)I2-I4:factor(ClinicalBinary)1    -0.1470  -0.0199    * 
## factor(Cell)I4-I11:factor(ClinicalBinary)1   -0.1212  -0.0278   ** 
## factor(Cell)I4-I12:factor(ClinicalBinary)1   -0.0884   0.0130      
## factor(Cell)I4-I17:factor(ClinicalBinary)1   -0.1164  -0.0116    * 
## 
## ---
## Signif. codes:  0 '***' 0.001 '**' 0.01 '*' 0.05 '.' 0.1 ' ' 1
```

```
## Cluster-robust standard errors
robust(tas20.clinical, cluster = StudyID, clubSandwich = TRUE)
```

```
## 
## Multivariate Meta-Analysis Model (k = 880; method: REML)
## 
## Variance Components:
## 
## outer factor: ESID         (nlvls = 880)
## inner factor: factor(Cell) (nlvls = 10)
## 
##              estim    sqrt  k.lvl  fixed    level 
## tau^2.1     0.0009  0.0307     88     no  I11-I12 
## tau^2.2     0.0007  0.0268     88     no  I11-I17 
## tau^2.3     0.0020  0.0447     88     no  I12-I17 
## tau^2.4     0.0004  0.0200     88     no   I2-I11 
## tau^2.5     0.0022  0.0470     88     no   I2-I12 
## tau^2.6     0.0025  0.0495     88     no   I2-I17 
## tau^2.7     0.0116  0.1075     88     no    I2-I4 
## tau^2.8     0.0040  0.0630     88     no   I4-I11 
## tau^2.9     0.0053  0.0727     88     no   I4-I12 
## tau^2.10    0.0061  0.0779     88     no   I4-I17 
## rho         0.0000                   yes          
## 
## outer factor: StudyID      (nlvls = 62)
## inner factor: factor(Cell) (nlvls = 10)
## 
##             estim    sqrt  fixed 
## gamma^2    0.0050  0.0708     no 
## phi        0.0000            yes 
## 
## Test for Residual Heterogeneity:
## QE(df = 860) = 6847.4154, p-val < .0001
## 
## Number of estimates:   880
## Number of clusters:    62
## Estimates per cluster: 10-40 (mean: 14.19, median: 10)
## 
## Test of Moderators (coefficients 1:20):¹
## F(df1 = 20, df2 = 21.17) = 87.2071, p-val < .0001
## 
## Model Results:
## 
##                                              estimate      se¹     tval¹ 
## factor(Cell)I11-I12                            0.3197  0.0157   20.3540  
## factor(Cell)I11-I17                            0.3510  0.0135   25.9449  
## factor(Cell)I12-I17                            0.2843  0.0162   17.5353  
## factor(Cell)I2-I11                             0.4546  0.0125   36.2394  
## factor(Cell)I2-I12                             0.3539  0.0158   22.3666  
## factor(Cell)I2-I17                             0.3896  0.0138   28.2896  
## factor(Cell)I2-I4                              0.4434  0.0214   20.7108  
## factor(Cell)I4-I11                             0.3694  0.0181   20.4351  
## factor(Cell)I4-I12                             0.2901  0.0190   15.2840  
## factor(Cell)I4-I17                             0.3316  0.0183   18.1182  
## factor(Cell)I11-I12:factor(ClinicalBinary)1   -0.0222  0.0179   -1.2383  
## factor(Cell)I11-I17:factor(ClinicalBinary)1   -0.0163  0.0232   -0.7024  
## factor(Cell)I12-I17:factor(ClinicalBinary)1   -0.0145  0.0273   -0.5326  
## factor(Cell)I2-I11:factor(ClinicalBinary)1    -0.0214  0.0159   -1.3490  
## factor(Cell)I2-I12:factor(ClinicalBinary)1    -0.0107  0.0229   -0.4693  
## factor(Cell)I2-I17:factor(ClinicalBinary)1    -0.0152  0.0282   -0.5395  
## factor(Cell)I2-I4:factor(ClinicalBinary)1     -0.0835  0.0328   -2.5430  
## factor(Cell)I4-I11:factor(ClinicalBinary)1    -0.0745  0.0232   -3.2162  
## factor(Cell)I4-I12:factor(ClinicalBinary)1    -0.0377  0.0308   -1.2237  
## factor(Cell)I4-I17:factor(ClinicalBinary)1    -0.0640  0.0339   -1.8863  
##                                                 df¹    pval¹    ci.lb¹ 
## factor(Cell)I11-I12                          49.13   <.0001    0.2881  
## factor(Cell)I11-I17                          49.68   <.0001    0.3238  
## factor(Cell)I12-I17                          48.51   <.0001    0.2517  
## factor(Cell)I2-I11                           50.94   <.0001    0.4294  
## factor(Cell)I2-I12                           48.69   <.0001    0.3221  
## factor(Cell)I2-I17                           48.89   <.0001    0.3619  
## factor(Cell)I2-I4                            46.18   <.0001    0.4003  
## factor(Cell)I4-I11                           48.01   <.0001    0.3331  
## factor(Cell)I4-I12                           47.26   <.0001    0.2519  
## factor(Cell)I4-I17                           47.16   <.0001    0.2948  
## factor(Cell)I11-I12:factor(ClinicalBinary)1  18.65   0.2310   -0.0597  
## factor(Cell)I11-I17:factor(ClinicalBinary)1  17.65   0.4916   -0.0652  
## factor(Cell)I12-I17:factor(ClinicalBinary)1  22.77   0.5995   -0.0710  
## factor(Cell)I2-I11:factor(ClinicalBinary)1   14.67   0.1978   -0.0554  
## factor(Cell)I2-I12:factor(ClinicalBinary)1    23.2   0.6432   -0.0580  
## factor(Cell)I2-I17:factor(ClinicalBinary)1   23.67   0.5946   -0.0735  
## factor(Cell)I2-I4:factor(ClinicalBinary)1    29.23   0.0165   -0.1506  
## factor(Cell)I4-I11:factor(ClinicalBinary)1   25.64   0.0035   -0.1221  
## factor(Cell)I4-I12:factor(ClinicalBinary)1   26.98   0.2317   -0.1009  
## factor(Cell)I4-I17:factor(ClinicalBinary)1   27.38   0.0699   -0.1336  
##                                                ci.ub¹      
## factor(Cell)I11-I12                           0.3513   *** 
## factor(Cell)I11-I17                           0.3782   *** 
## factor(Cell)I12-I17                           0.3168   *** 
## factor(Cell)I2-I11                            0.4798   *** 
## factor(Cell)I2-I12                            0.3857   *** 
## factor(Cell)I2-I17                            0.4172   *** 
## factor(Cell)I2-I4                             0.4865   *** 
## factor(Cell)I4-I11                            0.4058   *** 
## factor(Cell)I4-I12                            0.3282   *** 
## factor(Cell)I4-I17                            0.3684   *** 
## factor(Cell)I11-I12:factor(ClinicalBinary)1   0.0154       
## factor(Cell)I11-I17:factor(ClinicalBinary)1   0.0325       
## factor(Cell)I12-I17:factor(ClinicalBinary)1   0.0419       
## factor(Cell)I2-I11:factor(ClinicalBinary)1    0.0125       
## factor(Cell)I2-I12:factor(ClinicalBinary)1    0.0366       
## factor(Cell)I2-I17:factor(ClinicalBinary)1    0.0430       
## factor(Cell)I2-I4:factor(ClinicalBinary)1    -0.0164     * 
## factor(Cell)I4-I11:factor(ClinicalBinary)1   -0.0268    ** 
## factor(Cell)I4-I12:factor(ClinicalBinary)1    0.0255       
## factor(Cell)I4-I17:factor(ClinicalBinary)1    0.0056     . 
## 
## ---
## Signif. codes:  0 '***' 0.001 '**' 0.01 '*' 0.05 '.' 0.1 ' ' 1
## 
## 1) results based on cluster-robust inference (var-cov estimator: CR2,
##    approx t/F-tests and confidence intervals, df: Satterthwaite approx)
```

```
## Processing time in seconds
tas20.clinical$time
```

```
## [1] 11.598
```

```
## Variance explanation
## Proportional reduction of the variances

## Level: Primary studies
100*max((1-tas20.clinical$gamma2/tas20.mlmvrem2$gamma2), 0)
```

```
## [1] 6.408155
```

```
## Level: Effect sizes
100*max((1-tas20.clinical$tau2[1]/tas20.mlmvrem2$tau2[1]), 0)
```

```
## [1] 0
```

```
100*max((1-tas20.clinical$tau2[2]/tas20.mlmvrem2$tau2[2]), 0)
```

```
## [1] 0
```

```
100*max((1-tas20.clinical$tau2[3]/tas20.mlmvrem2$tau2[3]), 0)
```

```
## [1] 0
```

```
100*max((1-tas20.clinical$tau2[4]/tas20.mlmvrem2$tau2[4]), 0)
```

```
## [1] 0
```

```
100*max((1-tas20.clinical$tau2[5]/tas20.mlmvrem2$tau2[5]), 0)
```

```
## [1] 0
```

```
100*max((1-tas20.clinical$tau2[6]/tas20.mlmvrem2$tau2[6]), 0)
```

```
## [1] 0
```

```
100*max((1-tas20.clinical$tau2[7]/tas20.mlmvrem2$tau2[7]), 0)
```

```
## [1] 5.561111
```

```
100*max((1-tas20.clinical$tau2[8]/tas20.mlmvrem2$tau2[8]), 0)
```

```
## [1] 6.654061
```

```
100*max((1-tas20.clinical$tau2[9]/tas20.mlmvrem2$tau2[9]), 0)
```

```
## [1] 0
```

```
100*max((1-tas20.clinical$tau2[10]/tas20.mlmvrem2$tau2[10]), 0)
```

```
## [1] 0.4595325
```

```
## Model that provides the direct estimates for each subgroup
## Model specification
tas20.clinical2 <- rma.mv(yi = Correlation,
                          V = V, 
                          data = tas20,
                          random = list(~ factor(Cell) | ESID,
                                        ~ factor(Cell)  | StudyID),
                          struc = c("HCS", "CS"),
                          rho = 0,
                          phi = 0,
                          method = "REML",
                          mods = ~ factor(Cell):factor(ClinicalBinary) - 1,
                          time = TRUE,
                          sparse = TRUE,
                          control = list(optimizer = "optimParallel",
                                         ncpus = ncores))
```

```
## 
## Processing time: 0 hours, 0 minutes, 16.88 seconds
```

```
## Model summary
summary(tas20.clinical2)
```

```
## 
## Multivariate Meta-Analysis Model (k = 880; method: REML)
## 
##     logLik    Deviance         AIC         BIC        AICc   
##   720.5345  -1441.0690  -1379.0690  -1231.6041  -1376.6729   
## 
## Variance Components:
## 
## outer factor: ESID         (nlvls = 880)
## inner factor: factor(Cell) (nlvls = 10)
## 
##              estim    sqrt  k.lvl  fixed    level 
## tau^2.1     0.0009  0.0307     88     no  I11-I12 
## tau^2.2     0.0007  0.0268     88     no  I11-I17 
## tau^2.3     0.0020  0.0447     88     no  I12-I17 
## tau^2.4     0.0004  0.0200     88     no   I2-I11 
## tau^2.5     0.0022  0.0470     88     no   I2-I12 
## tau^2.6     0.0025  0.0495     88     no   I2-I17 
## tau^2.7     0.0116  0.1075     88     no    I2-I4 
## tau^2.8     0.0040  0.0630     88     no   I4-I11 
## tau^2.9     0.0053  0.0727     88     no   I4-I12 
## tau^2.10    0.0061  0.0779     88     no   I4-I17 
## rho         0.0000                   yes          
## 
## outer factor: StudyID      (nlvls = 62)
## inner factor: factor(Cell) (nlvls = 10)
## 
##             estim    sqrt  fixed 
## gamma^2    0.0050  0.0708     no 
## phi        0.0000            yes 
## 
## Test for Residual Heterogeneity:
## QE(df = 860) = 6847.4154, p-val < .0001
## 
## Test of Moderators (coefficients 1:20):
## QM(df = 20) = 6792.2177, p-val < .0001
## 
## Model Results:
## 
##                                              estimate      se     zval    pval 
## factor(Cell)I11-I12:factor(ClinicalBinary)0    0.3197  0.0120  26.6801  <.0001 
## factor(Cell)I11-I17:factor(ClinicalBinary)0    0.3510  0.0117  29.9672  <.0001 
## factor(Cell)I12-I17:factor(ClinicalBinary)0    0.2843  0.0128  22.1769  <.0001 
## factor(Cell)I2-I11:factor(ClinicalBinary)0     0.4546  0.0112  40.6692  <.0001 
## factor(Cell)I2-I12:factor(ClinicalBinary)0     0.3539  0.0128  27.5584  <.0001 
## factor(Cell)I2-I17:factor(ClinicalBinary)0     0.3896  0.0129  30.1740  <.0001 
## factor(Cell)I2-I4:factor(ClinicalBinary)0      0.4434  0.0177  25.1162  <.0001 
## factor(Cell)I4-I11:factor(ClinicalBinary)0     0.3694  0.0139  26.5508  <.0001 
## factor(Cell)I4-I12:factor(ClinicalBinary)0     0.2901  0.0148  19.5701  <.0001 
## factor(Cell)I4-I17:factor(ClinicalBinary)0     0.3316  0.0152  21.8398  <.0001 
## factor(Cell)I11-I12:factor(ClinicalBinary)1    0.2975  0.0174  17.0775  <.0001 
## factor(Cell)I11-I17:factor(ClinicalBinary)1    0.3347  0.0167  19.9911  <.0001 
## factor(Cell)I12-I17:factor(ClinicalBinary)1    0.2697  0.0192  14.0818  <.0001 
## factor(Cell)I2-I11:factor(ClinicalBinary)1     0.4331  0.0155  27.9186  <.0001 
## factor(Cell)I2-I12:factor(ClinicalBinary)1     0.3432  0.0192  17.8688  <.0001 
## factor(Cell)I2-I17:factor(ClinicalBinary)1     0.3743  0.0193  19.4144  <.0001 
## factor(Cell)I2-I4:factor(ClinicalBinary)1      0.3599  0.0287  12.5238  <.0001 
## factor(Cell)I4-I11:factor(ClinicalBinary)1     0.2949  0.0217  13.6093  <.0001 
## factor(Cell)I4-I12:factor(ClinicalBinary)1     0.2524  0.0233  10.8475  <.0001 
## factor(Cell)I4-I17:factor(ClinicalBinary)1     0.2675  0.0240  11.1549  <.0001 
##                                               ci.lb   ci.ub      
## factor(Cell)I11-I12:factor(ClinicalBinary)0  0.2962  0.3432  *** 
## factor(Cell)I11-I17:factor(ClinicalBinary)0  0.3281  0.3740  *** 
## factor(Cell)I12-I17:factor(ClinicalBinary)0  0.2591  0.3094  *** 
## factor(Cell)I2-I11:factor(ClinicalBinary)0   0.4327  0.4765  *** 
## factor(Cell)I2-I12:factor(ClinicalBinary)0   0.3288  0.3791  *** 
## factor(Cell)I2-I17:factor(ClinicalBinary)0   0.3643  0.4149  *** 
## factor(Cell)I2-I4:factor(ClinicalBinary)0    0.4088  0.4780  *** 
## factor(Cell)I4-I11:factor(ClinicalBinary)0   0.3421  0.3967  *** 
## factor(Cell)I4-I12:factor(ClinicalBinary)0   0.2610  0.3191  *** 
## factor(Cell)I4-I17:factor(ClinicalBinary)0   0.3018  0.3613  *** 
## factor(Cell)I11-I12:factor(ClinicalBinary)1  0.2634  0.3317  *** 
## factor(Cell)I11-I17:factor(ClinicalBinary)1  0.3019  0.3675  *** 
## factor(Cell)I12-I17:factor(ClinicalBinary)1  0.2322  0.3073  *** 
## factor(Cell)I2-I11:factor(ClinicalBinary)1   0.4027  0.4636  *** 
## factor(Cell)I2-I12:factor(ClinicalBinary)1   0.3055  0.3808  *** 
## factor(Cell)I2-I17:factor(ClinicalBinary)1   0.3365  0.4121  *** 
## factor(Cell)I2-I4:factor(ClinicalBinary)1    0.3036  0.4163  *** 
## factor(Cell)I4-I11:factor(ClinicalBinary)1   0.2525  0.3374  *** 
## factor(Cell)I4-I12:factor(ClinicalBinary)1   0.2068  0.2980  *** 
## factor(Cell)I4-I17:factor(ClinicalBinary)1   0.2205  0.3145  *** 
## 
## ---
## Signif. codes:  0 '***' 0.001 '**' 0.01 '*' 0.05 '.' 0.1 ' ' 1
```

```
## Cluster-robust standard errors
robust(tas20.clinical2, cluster = StudyID, clubSandwich = TRUE)
```

```
## 
## Multivariate Meta-Analysis Model (k = 880; method: REML)
## 
## Variance Components:
## 
## outer factor: ESID         (nlvls = 880)
## inner factor: factor(Cell) (nlvls = 10)
## 
##              estim    sqrt  k.lvl  fixed    level 
## tau^2.1     0.0009  0.0307     88     no  I11-I12 
## tau^2.2     0.0007  0.0268     88     no  I11-I17 
## tau^2.3     0.0020  0.0447     88     no  I12-I17 
## tau^2.4     0.0004  0.0200     88     no   I2-I11 
## tau^2.5     0.0022  0.0470     88     no   I2-I12 
## tau^2.6     0.0025  0.0495     88     no   I2-I17 
## tau^2.7     0.0116  0.1075     88     no    I2-I4 
## tau^2.8     0.0040  0.0630     88     no   I4-I11 
## tau^2.9     0.0053  0.0727     88     no   I4-I12 
## tau^2.10    0.0061  0.0779     88     no   I4-I17 
## rho         0.0000                   yes          
## 
## outer factor: StudyID      (nlvls = 62)
## inner factor: factor(Cell) (nlvls = 10)
## 
##             estim    sqrt  fixed 
## gamma^2    0.0050  0.0708     no 
## phi        0.0000            yes 
## 
## Test for Residual Heterogeneity:
## QE(df = 860) = 6847.4154, p-val < .0001
## 
## Number of estimates:   880
## Number of clusters:    62
## Estimates per cluster: 10-40 (mean: 14.19, median: 10)
## 
## Test of Moderators (coefficients 1:20):¹
## F(df1 = 20, df2 = 21.17) = 87.2071, p-val < .0001
## 
## Model Results:
## 
##                                              estimate      se¹     tval¹ 
## factor(Cell)I11-I12:factor(ClinicalBinary)0    0.3197  0.0157   20.3540  
## factor(Cell)I11-I17:factor(ClinicalBinary)0    0.3510  0.0135   25.9449  
## factor(Cell)I12-I17:factor(ClinicalBinary)0    0.2843  0.0162   17.5353  
## factor(Cell)I2-I11:factor(ClinicalBinary)0     0.4546  0.0125   36.2394  
## factor(Cell)I2-I12:factor(ClinicalBinary)0     0.3539  0.0158   22.3666  
## factor(Cell)I2-I17:factor(ClinicalBinary)0     0.3896  0.0138   28.2896  
## factor(Cell)I2-I4:factor(ClinicalBinary)0      0.4434  0.0214   20.7108  
## factor(Cell)I4-I11:factor(ClinicalBinary)0     0.3694  0.0181   20.4351  
## factor(Cell)I4-I12:factor(ClinicalBinary)0     0.2901  0.0190   15.2840  
## factor(Cell)I4-I17:factor(ClinicalBinary)0     0.3316  0.0183   18.1182  
## factor(Cell)I11-I12:factor(ClinicalBinary)1    0.2975  0.0146   20.4349  
## factor(Cell)I11-I17:factor(ClinicalBinary)1    0.3347  0.0225   14.8696  
## factor(Cell)I12-I17:factor(ClinicalBinary)1    0.2697  0.0258   10.4397  
## factor(Cell)I2-I11:factor(ClinicalBinary)1     0.4331  0.0153   28.3220  
## factor(Cell)I2-I12:factor(ClinicalBinary)1     0.3432  0.0214   16.0232  
## factor(Cell)I2-I17:factor(ClinicalBinary)1     0.3743  0.0282   13.2728  
## factor(Cell)I2-I4:factor(ClinicalBinary)1      0.3599  0.0281   12.8144  
## factor(Cell)I4-I11:factor(ClinicalBinary)1     0.2949  0.0177   16.6159  
## factor(Cell)I4-I12:factor(ClinicalBinary)1     0.2524  0.0285    8.8564  
## factor(Cell)I4-I17:factor(ClinicalBinary)1     0.2675  0.0312    8.5823  
##                                                 df¹    pval¹   ci.lb¹   ci.ub¹ 
## factor(Cell)I11-I12:factor(ClinicalBinary)0  49.13   <.0001   0.2881   0.3513  
## factor(Cell)I11-I17:factor(ClinicalBinary)0  49.68   <.0001   0.3238   0.3782  
## factor(Cell)I12-I17:factor(ClinicalBinary)0  48.51   <.0001   0.2517   0.3168  
## factor(Cell)I2-I11:factor(ClinicalBinary)0   50.94   <.0001   0.4294   0.4798  
## factor(Cell)I2-I12:factor(ClinicalBinary)0   48.69   <.0001   0.3221   0.3857  
## factor(Cell)I2-I17:factor(ClinicalBinary)0   48.89   <.0001   0.3619   0.4172  
## factor(Cell)I2-I4:factor(ClinicalBinary)0    46.18   <.0001   0.4003   0.4865  
## factor(Cell)I4-I11:factor(ClinicalBinary)0   48.01   <.0001   0.3331   0.4058  
## factor(Cell)I4-I12:factor(ClinicalBinary)0   47.26   <.0001   0.2519   0.3282  
## factor(Cell)I4-I17:factor(ClinicalBinary)0   47.16   <.0001   0.2948   0.3684  
## factor(Cell)I11-I12:factor(ClinicalBinary)1  20.74   <.0001   0.2672   0.3278  
## factor(Cell)I11-I17:factor(ClinicalBinary)1  21.09   <.0001   0.2879   0.3815  
## factor(Cell)I12-I17:factor(ClinicalBinary)1  21.22   <.0001   0.2160   0.3234  
## factor(Cell)I2-I11:factor(ClinicalBinary)1   21.03   <.0001   0.4013   0.4650  
## factor(Cell)I2-I12:factor(ClinicalBinary)1   21.37   <.0001   0.2987   0.3877  
## factor(Cell)I2-I17:factor(ClinicalBinary)1   21.64   <.0001   0.3158   0.4329  
## factor(Cell)I2-I4:factor(ClinicalBinary)1    21.11   <.0001   0.3015   0.4183  
## factor(Cell)I4-I11:factor(ClinicalBinary)1   21.19   <.0001   0.2580   0.3318  
## factor(Cell)I4-I12:factor(ClinicalBinary)1   21.13   <.0001   0.1931   0.3116  
## factor(Cell)I4-I17:factor(ClinicalBinary)1   21.16   <.0001   0.2027   0.3323  
##                                                  
## factor(Cell)I11-I12:factor(ClinicalBinary)0  *** 
## factor(Cell)I11-I17:factor(ClinicalBinary)0  *** 
## factor(Cell)I12-I17:factor(ClinicalBinary)0  *** 
## factor(Cell)I2-I11:factor(ClinicalBinary)0   *** 
## factor(Cell)I2-I12:factor(ClinicalBinary)0   *** 
## factor(Cell)I2-I17:factor(ClinicalBinary)0   *** 
## factor(Cell)I2-I4:factor(ClinicalBinary)0    *** 
## factor(Cell)I4-I11:factor(ClinicalBinary)0   *** 
## factor(Cell)I4-I12:factor(ClinicalBinary)0   *** 
## factor(Cell)I4-I17:factor(ClinicalBinary)0   *** 
## factor(Cell)I11-I12:factor(ClinicalBinary)1  *** 
## factor(Cell)I11-I17:factor(ClinicalBinary)1  *** 
## factor(Cell)I12-I17:factor(ClinicalBinary)1  *** 
## factor(Cell)I2-I11:factor(ClinicalBinary)1   *** 
## factor(Cell)I2-I12:factor(ClinicalBinary)1   *** 
## factor(Cell)I2-I17:factor(ClinicalBinary)1   *** 
## factor(Cell)I2-I4:factor(ClinicalBinary)1    *** 
## factor(Cell)I4-I11:factor(ClinicalBinary)1   *** 
## factor(Cell)I4-I12:factor(ClinicalBinary)1   *** 
## factor(Cell)I4-I17:factor(ClinicalBinary)1   *** 
## 
## ---
## Signif. codes:  0 '***' 0.001 '**' 0.01 '*' 0.05 '.' 0.1 ' ' 1
## 
## 1) results based on cluster-robust inference (var-cov estimator: CR2,
##    approx t/F-tests and confidence intervals, df: Satterthwaite approx)
```

```
## Processing time in seconds
tas20.clinical2$time
```

```
## [1] 16.884
```

Three correlations (`I2-I4`, `I4-I11`, and
`I4-I17`) were moderated negatively by the type of the
sample.

## Moderation by a continuous variable (gender distribution in the sample)

```
## Description of the moderator
psych::describe(tas20$PropFemale)
```

```
##    vars   n  mean    sd median trimmed   mad min max range  skew kurtosis   se
## X1    1 830 54.43 23.42   58.5   56.02 11.22   0 100   100 -0.66     0.77 0.81
```

```
hist(tas20$PropFemale,
     xlab = "Proportion of female participants",
     main = "Gender distribution in the study samples")
```

```
## Model that tests for moderator effects
## Model specification
tas20.gender <- rma.mv(yi = Correlation,
                       V = V,
                       data = tas20,
                       random = list(~ factor(Cell) | ESID,
                                     ~ factor(Cell) | StudyID),
                       struc = c("HCS", "CS"),
                       rho = 0,
                       phi = 0,
                       method = "REML",
                       mods = ~ factor(Cell) - 1 + factor(Cell):PropFemale,
                       time = TRUE,
                       sparse = TRUE,
                       control = list(optimizer = "optimParallel",
                                      ncpus = ncores))
```

```
## Warning: 50 rows with NAs omitted from model fitting.
```

```
## 
## Processing time: 0 hours, 0 minutes, 12.44 seconds
```

```
## Model summary
summary(tas20.gender)
```

```
## 
## Multivariate Meta-Analysis Model (k = 830; method: REML)
## 
##     logLik    Deviance         AIC         BIC        AICc   
##   699.3652  -1398.7305  -1336.7305  -1191.1224  -1334.1804   
## 
## Variance Components:
## 
## outer factor: ESID         (nlvls = 830)
## inner factor: factor(Cell) (nlvls = 10)
## 
##              estim    sqrt  k.lvl  fixed    level 
## tau^2.1     0.0012  0.0342     83     no  I11-I12 
## tau^2.2     0.0008  0.0285     83     no  I11-I17 
## tau^2.3     0.0026  0.0511     83     no  I12-I17 
## tau^2.4     0.0005  0.0220     83     no   I2-I11 
## tau^2.5     0.0024  0.0491     83     no   I2-I12 
## tau^2.6     0.0030  0.0546     83     no   I2-I17 
## tau^2.7     0.0128  0.1131     83     no    I2-I4 
## tau^2.8     0.0047  0.0686     83     no   I4-I11 
## tau^2.9     0.0061  0.0781     83     no   I4-I12 
## tau^2.10    0.0075  0.0864     83     no   I4-I17 
## rho         0.0000                   yes          
## 
## outer factor: StudyID      (nlvls = 58)
## inner factor: factor(Cell) (nlvls = 10)
## 
##             estim    sqrt  fixed 
## gamma^2    0.0039  0.0621     no 
## phi        0.0000            yes 
## 
## Test for Residual Heterogeneity:
## QE(df = 810) = 5747.7403, p-val < .0001
## 
## Test of Moderators (coefficients 1:20):
## QM(df = 20) = 6790.6675, p-val < .0001
## 
## Model Results:
## 
##                                 estimate      se     zval    pval    ci.lb 
## factor(Cell)I11-I12               0.3358  0.0196  17.1544  <.0001   0.2974 
## factor(Cell)I11-I17               0.3816  0.0181  21.0417  <.0001   0.3461 
## factor(Cell)I12-I17               0.3045  0.0234  13.0352  <.0001   0.2587 
## factor(Cell)I2-I11                0.4509  0.0163  27.7223  <.0001   0.4190 
## factor(Cell)I2-I12                0.3499  0.0227  15.3991  <.0001   0.3053 
## factor(Cell)I2-I17                0.4123  0.0236  17.4329  <.0001   0.3659 
## factor(Cell)I2-I4                 0.3465  0.0374   9.2702  <.0001   0.2732 
## factor(Cell)I4-I11                0.3343  0.0272  12.2783  <.0001   0.2809 
## factor(Cell)I4-I12                0.2512  0.0296   8.4888  <.0001   0.1932 
## factor(Cell)I4-I17                0.3102  0.0313   9.8952  <.0001   0.2487 
## factor(Cell)I11-I12:PropFemale   -0.0006  0.0003  -1.7937  0.0729  -0.0012 
## factor(Cell)I11-I17:PropFemale   -0.0007  0.0003  -2.6703  0.0076  -0.0013 
## factor(Cell)I12-I17:PropFemale   -0.0006  0.0004  -1.4667  0.1425  -0.0013 
## factor(Cell)I2-I11:PropFemale    -0.0002  0.0002  -0.8460  0.3975  -0.0007 
## factor(Cell)I2-I12:PropFemale    -0.0001  0.0004  -0.3330  0.7391  -0.0008 
## factor(Cell)I2-I17:PropFemale    -0.0006  0.0004  -1.5483  0.1215  -0.0014 
## factor(Cell)I2-I4:PropFemale      0.0012  0.0006   1.9366  0.0528  -0.0000 
## factor(Cell)I4-I11:PropFemale     0.0001  0.0004   0.1980  0.8431  -0.0008 
## factor(Cell)I4-I12:PropFemale     0.0004  0.0005   0.7501  0.4532  -0.0006 
## factor(Cell)I4-I17:PropFemale    -0.0000  0.0005  -0.0665  0.9470  -0.0011 
##                                   ci.ub      
## factor(Cell)I11-I12              0.3742  *** 
## factor(Cell)I11-I17              0.4172  *** 
## factor(Cell)I12-I17              0.3503  *** 
## factor(Cell)I2-I11               0.4827  *** 
## factor(Cell)I2-I12               0.3944  *** 
## factor(Cell)I2-I17               0.4586  *** 
## factor(Cell)I2-I4                0.4197  *** 
## factor(Cell)I4-I11               0.3877  *** 
## factor(Cell)I4-I12               0.3092  *** 
## factor(Cell)I4-I17               0.3716  *** 
## factor(Cell)I11-I12:PropFemale   0.0001    . 
## factor(Cell)I11-I17:PropFemale  -0.0002   ** 
## factor(Cell)I12-I17:PropFemale   0.0002      
## factor(Cell)I2-I11:PropFemale    0.0003      
## factor(Cell)I2-I12:PropFemale    0.0006      
## factor(Cell)I2-I17:PropFemale    0.0002      
## factor(Cell)I2-I4:PropFemale     0.0024    . 
## factor(Cell)I4-I11:PropFemale    0.0010      
## factor(Cell)I4-I12:PropFemale    0.0013      
## factor(Cell)I4-I17:PropFemale    0.0010      
## 
## ---
## Signif. codes:  0 '***' 0.001 '**' 0.01 '*' 0.05 '.' 0.1 ' ' 1
```

```
## Cluster-robust standard errors
robust(tas20.gender, cluster = StudyID, clubSandwich = TRUE)
```

```
## 
## Multivariate Meta-Analysis Model (k = 830; method: REML)
## 
## Variance Components:
## 
## outer factor: ESID         (nlvls = 830)
## inner factor: factor(Cell) (nlvls = 10)
## 
##              estim    sqrt  k.lvl  fixed    level 
## tau^2.1     0.0012  0.0342     83     no  I11-I12 
## tau^2.2     0.0008  0.0285     83     no  I11-I17 
## tau^2.3     0.0026  0.0511     83     no  I12-I17 
## tau^2.4     0.0005  0.0220     83     no   I2-I11 
## tau^2.5     0.0024  0.0491     83     no   I2-I12 
## tau^2.6     0.0030  0.0546     83     no   I2-I17 
## tau^2.7     0.0128  0.1131     83     no    I2-I4 
## tau^2.8     0.0047  0.0686     83     no   I4-I11 
## tau^2.9     0.0061  0.0781     83     no   I4-I12 
## tau^2.10    0.0075  0.0864     83     no   I4-I17 
## rho         0.0000                   yes          
## 
## outer factor: StudyID      (nlvls = 58)
## inner factor: factor(Cell) (nlvls = 10)
## 
##             estim    sqrt  fixed 
## gamma^2    0.0039  0.0621     no 
## phi        0.0000            yes 
## 
## Test for Residual Heterogeneity:
## QE(df = 810) = 5747.7403, p-val < .0001
## 
## Number of estimates:   830
## Number of clusters:    58
## Estimates per cluster: 10-40 (mean: 14.31, median: 10)
## 
## Test of Moderators (coefficients 1:20):¹
## F(df1 = 20, df2 = 0.04) = 0.2763, p-val = 0.9602
## 
## Model Results:
## 
##                                 estimate      se¹     tval¹     df¹    pval¹ 
## factor(Cell)I11-I12               0.3358  0.0294   11.4208       8   <.0001  
## factor(Cell)I11-I17               0.3816  0.0200   19.0487    7.47   <.0001  
## factor(Cell)I12-I17               0.3045  0.0293   10.3752    9.66   <.0001  
## factor(Cell)I2-I11                0.4509  0.0162   27.8828    7.05   <.0001  
## factor(Cell)I2-I12                0.3499  0.0297   11.7784    9.54   <.0001  
## factor(Cell)I2-I17                0.4123  0.0236   17.4529   10.18   <.0001  
## factor(Cell)I2-I4                 0.3465  0.0334   10.3776   12.94   <.0001  
## factor(Cell)I4-I11                0.3343  0.0250   13.3821   11.01   <.0001  
## factor(Cell)I4-I12                0.2512  0.0271    9.2774   11.52   <.0001  
## factor(Cell)I4-I17                0.3102  0.0250   12.4202   11.96   <.0001  
## factor(Cell)I11-I12:PropFemale   -0.0006  0.0005   -1.1227    4.87   0.3139  
## factor(Cell)I11-I17:PropFemale   -0.0007  0.0003   -2.1506    4.14   0.0956  
## factor(Cell)I12-I17:PropFemale   -0.0006  0.0005   -1.1029    7.04   0.3064  
## factor(Cell)I2-I11:PropFemale    -0.0002  0.0002   -0.8255    3.33   0.4641  
## factor(Cell)I2-I12:PropFemale    -0.0001  0.0005   -0.2387    6.81   0.8183  
## factor(Cell)I2-I17:PropFemale    -0.0006  0.0004   -1.4119     7.6   0.1976  
## factor(Cell)I2-I4:PropFemale      0.0012  0.0006    2.1762    11.9   0.0504  
## factor(Cell)I4-I11:PropFemale     0.0001  0.0004    0.2272    8.95   0.8254  
## factor(Cell)I4-I12:PropFemale     0.0004  0.0004    0.8942    9.73   0.3928  
## factor(Cell)I4-I17:PropFemale    -0.0000  0.0004   -0.0878    10.4   0.9317  
##                                   ci.lb¹   ci.ub¹      
## factor(Cell)I11-I12              0.2680   0.4036   *** 
## factor(Cell)I11-I17              0.3349   0.4284   *** 
## factor(Cell)I12-I17              0.2388   0.3702   *** 
## factor(Cell)I2-I11               0.4127   0.4891   *** 
## factor(Cell)I2-I12               0.2832   0.4165   *** 
## factor(Cell)I2-I17               0.3598   0.4648   *** 
## factor(Cell)I2-I4                0.2743   0.4187   *** 
## factor(Cell)I4-I11               0.2793   0.3893   *** 
## factor(Cell)I4-I12               0.1919   0.3104   *** 
## factor(Cell)I4-I17               0.2557   0.3646   *** 
## factor(Cell)I11-I12:PropFemale  -0.0018   0.0007       
## factor(Cell)I11-I17:PropFemale  -0.0017   0.0002     . 
## factor(Cell)I12-I17:PropFemale  -0.0017   0.0006       
## factor(Cell)I2-I11:PropFemale   -0.0010   0.0005       
## factor(Cell)I2-I12:PropFemale   -0.0013   0.0011       
## factor(Cell)I2-I17:PropFemale   -0.0016   0.0004       
## factor(Cell)I2-I4:PropFemale    -0.0000   0.0024     . 
## factor(Cell)I4-I11:PropFemale   -0.0008   0.0010       
## factor(Cell)I4-I12:PropFemale   -0.0006   0.0013       
## factor(Cell)I4-I17:PropFemale   -0.0009   0.0008       
## 
## ---
## Signif. codes:  0 '***' 0.001 '**' 0.01 '*' 0.05 '.' 0.1 ' ' 1
## 
## 1) results based on cluster-robust inference (var-cov estimator: CR2,
##    approx t/F-tests and confidence intervals, df: Satterthwaite approx)
```

```
## Processing time in seconds
tas20.gender$time
```

```
## [1] 12.444
```

```
## Variance explanation
## New sampling covariance matrix
SampCov2 <- metafor::rcalc(Correlation ~ Var1 + Var2 | SampleID,
                           ni = N,
                           data = tas20[complete.cases(tas20$PropFemale),])

## Extract and save the sampling variance-covariance matrix
V2 <- SampCov2$V

## Sampling variances
diag(V2)
```

```
##        I2.I4       I11.I2       I11.I4       I12.I2       I12.I4      I11.I12 
## 1.599125e-03 1.236524e-03 1.515391e-03 1.361136e-03 1.482373e-03 1.129108e-03 
##       I17.I2       I17.I4      I11.I17      I12.I17        I2.I4       I11.I2 
## 1.317307e-03 1.457387e-03 1.040501e-03 1.141033e-03 2.083161e-03 2.913334e-03 
##       I11.I4       I12.I2       I12.I4      I11.I12       I17.I2       I17.I4 
## 2.856482e-03 3.696393e-03 3.283130e-03 4.037332e-03 3.219459e-03 2.766440e-03 
##      I11.I17      I12.I17        I2.I4       I11.I2       I11.I4       I12.I2 
## 3.664168e-03 3.677764e-03 6.558311e-04 6.299473e-04 6.648659e-04 7.703254e-04 
##       I12.I4      I11.I12       I17.I2       I17.I4      I11.I17      I12.I17 
## 7.971858e-04 7.683016e-04 7.982759e-04 7.996276e-04 7.947588e-04 8.885878e-04 
##        I2.I4       I11.I2       I11.I4       I12.I2       I12.I4      I11.I12 
## 2.756611e-03 3.714954e-03 3.664673e-03 3.286145e-03 3.214281e-03 3.948851e-03 
##       I17.I2       I17.I4      I11.I17      I12.I17        I2.I4       I11.I2 
## 2.848336e-03 2.756611e-03 3.714954e-03 3.286145e-03 1.364100e-03 1.801300e-03 
##       I11.I4       I12.I2       I12.I4      I11.I12       I17.I2       I17.I4 
## 1.819041e-03 1.871273e-03 1.887397e-03 2.135155e-03 1.336858e-03 1.364100e-03 
##      I11.I17      I12.I17        I2.I4       I11.I2       I11.I4       I12.I2 
## 1.801300e-03 1.871273e-03 8.158149e-04 9.290667e-04 9.037801e-04 1.107972e-03 
##       I12.I4      I11.I12       I17.I2       I17.I4      I11.I17      I12.I17 
## 1.091215e-03 1.156059e-03 1.009262e-03 9.876559e-04 1.071643e-03 1.200662e-03 
##        I2.I4       I11.I2       I11.I4       I12.I2       I12.I4      I11.I12 
## 9.047625e-04 9.455370e-04 1.151861e-03 1.302409e-03 1.418708e-03 1.435139e-03 
##       I17.I2       I17.I4      I11.I17      I12.I17        I2.I4       I11.I2 
## 1.005929e-03 1.198120e-03 1.225947e-03 1.459005e-03 4.526830e-03 3.826374e-03 
##       I11.I4       I12.I2       I12.I4      I11.I12       I17.I2       I17.I4 
## 5.122206e-03 4.631698e-03 5.395418e-03 5.177501e-03 3.172277e-03 4.884266e-03 
##      I11.I17      I12.I17        I2.I4       I11.I2       I11.I4       I12.I2 
## 4.371965e-03 4.959768e-03 3.399607e-03 3.531606e-03 4.223479e-03 4.390758e-03 
##       I12.I4      I11.I12       I17.I2       I17.I4      I11.I17      I12.I17 
## 4.839166e-03 4.897457e-03 3.465824e-03 4.175235e-03 4.269701e-03 4.868503e-03 
##        I2.I4       I11.I2       I11.I4       I12.I2       I12.I4      I11.I12 
## 1.919698e-03 2.021024e-03 2.344643e-03 2.367488e-03 2.595976e-03 2.643837e-03 
##       I17.I2       I17.I4      I11.I17      I12.I17        I2.I4       I11.I2 
## 1.850888e-03 2.218407e-03 2.297394e-03 2.563007e-03 5.881133e-04 7.532840e-04 
##       I11.I4       I12.I2       I12.I4      I11.I12       I17.I2       I17.I4 
## 7.831177e-04 9.972691e-04 1.003446e-03 1.023640e-03 7.532840e-04 7.831177e-04 
##      I11.I17      I12.I17        I2.I4       I11.I2       I11.I4       I12.I2 
## 8.840223e-04 1.023640e-03 1.207370e-03 1.025076e-03 1.245361e-03 1.304703e-03 
##       I12.I4      I11.I12       I17.I2       I17.I4      I11.I17      I12.I17 
## 1.413616e-03 1.332708e-03 1.220224e-03 1.363773e-03 1.256920e-03 1.419168e-03 
##        I2.I4       I11.I2       I11.I4       I12.I2       I12.I4      I11.I12 
## 2.548471e-03 3.462226e-03 3.938566e-03 4.454939e-03 4.603384e-03 4.720985e-03 
##       I17.I2       I17.I4      I11.I17      I12.I17        I2.I4       I11.I2 
## 3.514732e-03 3.974575e-03 4.353279e-03 4.727280e-03 1.824390e-04 3.691964e-04 
##       I11.I4       I12.I2       I12.I4      I11.I12       I17.I2       I17.I4 
## 4.035307e-04 3.981659e-04 3.980344e-04 4.370922e-04 4.220180e-04 4.079157e-04 
##      I11.I17      I12.I17        I2.I4       I11.I2       I11.I4       I12.I2 
## 4.328949e-04 3.814419e-04 1.521860e-03 1.234503e-03 1.404132e-03 1.898232e-03 
##       I12.I4      I11.I12       I17.I2       I17.I4      I11.I17      I12.I17 
## 1.983799e-03 1.822774e-03 1.764798e-03 1.869096e-03 1.673416e-03 2.113058e-03 
##        I2.I4       I11.I2       I11.I4       I12.I2       I12.I4      I11.I12 
## 3.244120e-03 2.502262e-03 3.511968e-03 3.488623e-03 4.019556e-03 3.746659e-03 
##       I17.I2       I17.I4      I11.I17      I12.I17        I2.I4       I11.I2 
## 3.121384e-03 3.650800e-03 3.386124e-03 3.945675e-03 7.021458e-04 6.887308e-04 
##       I11.I4       I12.I2       I12.I4      I11.I12       I17.I2       I17.I4 
## 8.458662e-04 1.021931e-03 1.075502e-03 1.072252e-03 1.003798e-03 1.063422e-03 
##      I11.I17      I12.I17        I2.I4       I11.I2       I11.I4       I12.I2 
## 1.059799e-03 1.142828e-03 1.884817e-04 3.056365e-04 3.279328e-04 2.277019e-04 
##       I12.I4      I11.I12       I17.I2       I17.I4      I11.I17      I12.I17 
## 2.302509e-04 3.450495e-04 2.917098e-04 2.712210e-04 3.459968e-04 2.994805e-04 
##        I2.I4       I11.I2       I11.I4       I12.I2       I12.I4      I11.I12 
## 1.758401e-03 2.249010e-03 2.289691e-03 3.007416e-03 3.113100e-03 3.164951e-03 
##       I17.I2       I17.I4      I11.I17      I12.I17        I2.I4       I11.I2 
## 2.697279e-03 2.809256e-03 2.986797e-03 3.060880e-03 4.207575e-04 3.709871e-04 
##       I11.I4       I12.I2       I12.I4      I11.I12       I17.I2       I17.I4 
## 4.308508e-04 4.438532e-04 4.517396e-04 4.471198e-04 4.186442e-04 4.446665e-04 
##      I11.I17      I12.I17        I2.I4       I11.I2       I11.I4       I12.I2 
## 4.293554e-04 4.515488e-04 2.367710e-04 2.924077e-04 3.177167e-04 3.341753e-04 
##       I12.I4      I11.I12       I17.I2       I17.I4      I11.I17      I12.I17 
## 3.533531e-04 3.806236e-04 2.972489e-04 3.218728e-04 3.573662e-04 3.829137e-04 
##        I2.I4       I11.I2       I11.I4       I12.I2       I12.I4      I11.I12 
## 1.726539e-03 2.059211e-03 2.238925e-03 1.907217e-03 2.100293e-03 2.369952e-03 
##       I17.I2       I17.I4      I11.I17      I12.I17        I2.I4       I11.I2 
## 2.457169e-03 2.280909e-03 2.803632e-03 2.715210e-03 1.067243e-03 1.664983e-03 
##       I11.I4       I12.I2       I12.I4      I11.I12       I17.I2       I17.I4 
## 1.932354e-03 2.641486e-03 2.718439e-03 2.276301e-03 2.686437e-03 2.701651e-03 
##      I11.I17      I12.I17        I2.I4       I11.I2       I11.I4       I12.I2 
## 2.096382e-03 2.537486e-03 1.819312e-03 1.461944e-03 1.944933e-03 1.729723e-03 
##       I12.I4      I11.I12       I17.I2       I17.I4      I11.I17      I12.I17 
## 2.080528e-03 1.873504e-03 2.392344e-03 2.392344e-03 2.392344e-03 2.392344e-03 
##        I2.I4       I11.I2       I11.I4       I12.I2       I12.I4      I11.I12 
## 3.190455e-03 3.352036e-03 3.788874e-03 3.020368e-03 3.487750e-03 3.639526e-03 
##       I17.I2       I17.I4      I11.I17      I12.I17        I2.I4       I11.I2 
## 2.527576e-03 3.033693e-03 3.200032e-03 2.858970e-03 9.855211e-04 1.988280e-03 
##       I11.I4       I12.I2       I12.I4      I11.I12       I17.I2       I17.I4 
## 2.041326e-03 1.872443e-03 1.733672e-03 1.890238e-03 1.856938e-03 1.623631e-03 
##      I11.I17      I12.I17        I2.I4       I11.I2       I11.I4       I12.I2 
## 1.973016e-03 1.958431e-03 2.504528e-03 2.089728e-03 2.440555e-03 2.552306e-03 
##       I12.I4      I11.I12       I17.I2       I17.I4      I11.I17      I12.I17 
## 2.596533e-03 2.515036e-03 2.288425e-03 2.508880e-03 2.109125e-03 2.554834e-03 
##        I2.I4       I11.I2       I11.I4       I12.I2       I12.I4      I11.I12 
## 1.702256e-04 2.139733e-04 2.469858e-04 2.600233e-04 2.837332e-04 3.036084e-04 
##       I17.I2       I17.I4      I11.I17      I12.I17        I2.I4       I11.I2 
## 2.017881e-04 2.370755e-04 2.676048e-04 2.982290e-04 2.307982e-04 2.624307e-04 
##       I11.I4       I12.I2       I12.I4      I11.I12       I17.I2       I17.I4 
## 2.995895e-04 3.523563e-04 3.717869e-04 3.832068e-04 2.482702e-04 2.879347e-04 
##      I11.I17      I12.I17        I2.I4       I11.I2       I11.I4       I12.I2 
## 3.120960e-04 3.781637e-04 4.193519e-03 4.014135e-03 4.384907e-03 4.342899e-03 
##       I12.I4      I11.I12       I17.I2       I17.I4      I11.I17      I12.I17 
## 4.443222e-03 4.428919e-03 3.733069e-03 4.333453e-03 4.245548e-03 4.405898e-03 
##        I2.I4       I11.I2       I11.I4       I12.I2       I12.I4      I11.I12 
## 2.028961e-03 1.542083e-03 2.150082e-03 1.736323e-03 2.209170e-03 1.949253e-03 
##       I17.I2       I17.I4      I11.I17      I12.I17        I2.I4       I11.I2 
## 1.782157e-03 2.222735e-03 1.981100e-03 2.079832e-03 1.718820e-03 1.495342e-03 
##       I11.I4       I12.I2       I12.I4      I11.I12       I17.I2       I17.I4 
## 2.026835e-03 1.823610e-03 2.187266e-03 2.092019e-03 1.767678e-03 2.160602e-03 
##      I11.I17      I12.I17        I2.I4       I11.I2       I11.I4       I12.I2 
## 2.057339e-03 2.207517e-03 6.282669e-04 4.284313e-04 6.506146e-04 5.964041e-04 
##       I12.I4      I11.I12       I17.I2       I17.I4      I11.I17      I12.I17 
## 6.934466e-04 5.375475e-04 5.459028e-04 6.807315e-04 4.810634e-04 6.050322e-04 
##        I2.I4       I11.I2       I11.I4       I12.I2       I12.I4      I11.I12 
## 7.967281e-04 7.000597e-04 8.998769e-04 8.641061e-04 9.766630e-04 9.423210e-04 
##       I17.I2       I17.I4      I11.I17      I12.I17        I2.I4       I11.I2 
## 8.549572e-04 9.725047e-04 9.366008e-04 9.966252e-04 2.015893e-03 2.043188e-03 
##       I11.I4       I12.I2       I12.I4      I11.I12       I17.I2       I17.I4 
## 2.292928e-03 2.149315e-03 2.369768e-03 2.386706e-03 2.250125e-03 2.442152e-03 
##      I11.I17      I12.I17        I2.I4       I11.I2       I11.I4       I12.I2 
## 2.456845e-03 2.513470e-03 1.326123e-03 1.075737e-03 1.332703e-03 1.326431e-03 
##       I12.I4      I11.I12       I17.I2       I17.I4      I11.I17      I12.I17 
## 1.537073e-03 1.348193e-03 1.206878e-03 1.441321e-03 1.226666e-03 1.438512e-03 
##        I2.I4       I11.I2       I11.I4       I12.I2       I12.I4      I11.I12 
## 2.309397e-03 3.477027e-03 3.496165e-03 4.827273e-03 5.367924e-03 4.122511e-03 
##       I17.I2       I17.I4      I11.I17      I12.I17        I2.I4       I11.I2 
## 3.990148e-03 4.162151e-03 3.141367e-03 3.376650e-03 1.156112e-03 1.168353e-03 
##       I11.I4       I12.I2       I12.I4      I11.I12       I17.I2       I17.I4 
## 1.263469e-03 1.330707e-03 1.383125e-03 1.387937e-03 1.357882e-03 1.402932e-03 
##      I11.I17      I12.I17        I2.I4       I11.I2       I11.I4       I12.I2 
## 1.407060e-03 1.460432e-03 8.463504e-04 9.713057e-04 1.045469e-03 9.945733e-04 
##       I12.I4      I11.I12       I17.I2       I17.I4      I11.I17      I12.I17 
## 1.063848e-03 1.131992e-03 1.017188e-03 1.081657e-03 1.144897e-03 1.156403e-03 
##        I2.I4       I11.I2       I11.I4       I12.I2       I12.I4      I11.I12 
## 1.407531e-03 1.648952e-03 1.648952e-03 1.979988e-03 1.979988e-03 2.147872e-03 
##       I17.I2       I17.I4      I11.I17      I12.I17        I2.I4       I11.I2 
## 1.947975e-03 1.947975e-03 2.120439e-03 2.347924e-03 4.359478e-03 3.944602e-03 
##       I11.I4       I12.I2       I12.I4      I11.I12       I17.I2       I17.I4 
## 4.287730e-03 4.173188e-03 4.346673e-03 3.819961e-03 4.223489e-03 4.359478e-03 
##      I11.I17      I12.I17        I2.I4       I11.I2       I11.I4       I12.I2 
## 3.944602e-03 4.173188e-03 3.210464e-03 3.754773e-03 3.764964e-03 5.452099e-03 
##       I12.I4      I11.I12       I17.I2       I17.I4      I11.I17      I12.I17 
## 5.379659e-03 5.295781e-03 4.424213e-03 4.219242e-03 4.111453e-03 5.288729e-03 
##        I2.I4       I11.I2       I11.I4       I12.I2       I12.I4      I11.I12 
## 1.210132e-03 1.487307e-03 1.607512e-03 1.727272e-03 1.826771e-03 2.013564e-03 
##       I17.I2       I17.I4      I11.I17      I12.I17        I2.I4       I11.I2 
## 1.503665e-03 1.476849e-03 1.840319e-03 1.985894e-03 1.255863e-03 1.632906e-03 
##       I11.I4       I12.I2       I12.I4      I11.I12       I17.I2       I17.I4 
## 1.835765e-03 1.577110e-03 1.791818e-03 2.017605e-03 1.433613e-03 1.677669e-03 
##      I11.I17      I12.I17        I2.I4       I11.I2       I11.I4       I12.I2 
## 1.937864e-03 1.900285e-03 1.616238e-03 5.517395e-03 5.517395e-03 5.231651e-03 
##       I12.I4      I11.I12       I17.I2       I17.I4      I11.I17      I12.I17 
## 5.231651e-03 6.231791e-03 5.044574e-03 5.044574e-03 6.201926e-03 6.132511e-03 
##        I2.I4       I11.I2       I11.I4       I12.I2       I12.I4      I11.I12 
## 3.879613e-04 3.739606e-04 4.354901e-04 4.243344e-04 4.572361e-04 4.289967e-04 
##       I17.I2       I17.I4      I11.I17      I12.I17        I2.I4       I11.I2 
## 4.462144e-04 4.697940e-04 4.451931e-04 4.610038e-04 2.822307e-04 2.688419e-04 
##       I11.I4       I12.I2       I12.I4      I11.I12       I17.I2       I17.I4 
## 3.111590e-04 3.079701e-04 3.330722e-04 3.040385e-04 3.417775e-04 3.555454e-04 
##      I11.I17      I12.I17        I2.I4       I11.I2       I11.I4       I12.I2 
## 3.270564e-04 3.355362e-04 2.834294e-03 3.060514e-03 3.658949e-03 4.098582e-03 
##       I12.I4      I11.I12       I17.I2       I17.I4      I11.I17      I12.I17 
## 4.377635e-03 4.443327e-03 2.485635e-03 3.240292e-03 3.428600e-03 4.272063e-03 
##        I2.I4       I11.I2       I11.I4       I12.I2       I12.I4      I11.I12 
## 3.956324e-03 2.673192e-03 4.138226e-03 3.472154e-03 4.300260e-03 3.813351e-03 
##       I17.I2       I17.I4      I11.I17      I12.I17        I2.I4       I11.I2 
## 3.253456e-03 4.257647e-03 3.663835e-03 4.041762e-03 3.183939e-03 3.072408e-03 
##       I11.I4       I12.I2       I12.I4      I11.I12       I17.I2       I17.I4 
## 3.130977e-03 3.422794e-03 3.435937e-03 3.407014e-03 3.303608e-03 3.332459e-03 
##      I11.I17      I12.I17        I2.I4       I11.I2       I11.I4       I12.I2 
## 3.269065e-03 3.466545e-03 1.379235e-03 1.886432e-03 1.947726e-03 1.999749e-03 
##       I12.I4      I11.I12       I17.I2       I17.I4      I11.I17      I12.I17 
## 2.056936e-03 2.408681e-03 2.357162e-03 2.400168e-03 2.660313e-03 2.715082e-03 
##        I2.I4       I11.I2       I11.I4       I12.I2       I12.I4      I11.I12 
## 2.845704e-03 2.387568e-03 2.687847e-03 2.629114e-03 2.864341e-03 2.419195e-03 
##       I17.I2       I17.I4      I11.I17      I12.I17        I2.I4       I11.I2 
## 2.679562e-03 2.900851e-03 2.481452e-03 2.703078e-03 1.352837e-03 1.675918e-03 
##       I11.I4       I12.I2       I12.I4      I11.I12       I17.I2       I17.I4 
## 1.569295e-03 1.675918e-03 1.569295e-03 1.837262e-03 1.878957e-03 1.796492e-03 
##      I11.I17      I12.I17        I2.I4       I11.I2       I11.I4       I12.I2 
## 2.002208e-03 2.002208e-03 5.753215e-04 5.646924e-04 6.826430e-04 5.963549e-04 
##       I12.I4      I11.I12       I17.I2       I17.I4      I11.I17      I12.I17 
## 7.056864e-04 6.983344e-04 6.373948e-04 7.352054e-04 7.286659e-04 7.480547e-04 
##        I2.I4       I11.I2       I11.I4       I12.I2       I12.I4      I11.I12 
## 8.080673e-04 7.046609e-04 7.508912e-04 8.746035e-04 9.041689e-04 8.277777e-04 
##       I17.I2       I17.I4      I11.I17      I12.I17        I2.I4       I11.I2 
## 8.467607e-04 8.792094e-04 7.955181e-04 9.323028e-04 5.437756e-03 4.832971e-03 
##       I11.I4       I12.I2       I12.I4      I11.I12       I17.I2       I17.I4 
## 5.899407e-03 6.158188e-03 6.832159e-03 6.508682e-03 5.926429e-03 6.672120e-03 
##      I11.I17      I12.I17        I2.I4       I11.I2       I11.I4       I12.I2 
## 6.313540e-03 7.085914e-03 8.244164e-03 6.983477e-03 9.422539e-03 8.336052e-03 
##       I12.I4      I11.I12       I17.I2       I17.I4      I11.I17      I12.I17 
## 9.653275e-03 9.449946e-03 5.370953e-03 9.120184e-03 8.496134e-03 9.164563e-03 
##        I2.I4       I11.I2       I11.I4       I12.I2       I12.I4      I11.I12 
## 2.170857e-03 2.209946e-03 2.209946e-03 2.622176e-03 2.622176e-03 2.651621e-03 
##       I17.I2       I17.I4      I11.I17      I12.I17        I2.I4       I11.I2 
## 2.439327e-03 2.439327e-03 2.472854e-03 2.822528e-03 1.847355e-03 2.383664e-03 
##       I11.I4       I12.I2       I12.I4      I11.I12       I17.I2       I17.I4 
## 1.971942e-03 2.597395e-03 2.253902e-03 2.670929e-03 2.020312e-03 1.509699e-03 
##      I11.I17      I12.I17        I2.I4       I11.I2       I11.I4       I12.I2 
## 2.134331e-03 2.390368e-03 1.507484e-03 2.464785e-03 1.949831e-03 2.715689e-03 
##       I12.I4      I11.I12       I17.I2       I17.I4      I11.I17      I12.I17 
## 2.325101e-03 2.891519e-03 1.955365e-03 1.240118e-03 2.309793e-03 2.599712e-03 
##        I2.I4       I11.I2       I11.I4       I12.I2       I12.I4      I11.I12 
## 5.301106e-03 6.037069e-03 5.650050e-03 5.769314e-03 5.248139e-03 6.009940e-03 
##       I17.I2       I17.I4      I11.I17      I12.I17        I2.I4       I11.I2 
## 5.659431e-03 5.084886e-03 5.925843e-03 5.618780e-03 3.005337e-03 3.562976e-03 
##       I11.I4       I12.I2       I12.I4      I11.I12       I17.I2       I17.I4 
## 4.511048e-03 3.736263e-03 3.646142e-03 4.665368e-03 3.833410e-03 4.398114e-03 
##      I11.I17      I12.I17        I2.I4       I11.I2       I11.I4       I12.I2 
## 3.614759e-03 3.861898e-03 4.838629e-04 3.368878e-04 5.065548e-04 4.232962e-04 
##       I12.I4      I11.I12       I17.I2       I17.I4      I11.I17      I12.I17 
## 5.374842e-04 4.509387e-04 3.747223e-04 4.686125e-04 4.015855e-04 5.311601e-04 
##        I2.I4       I11.I2       I11.I4       I12.I2       I12.I4      I11.I12 
## 6.110967e-03 5.302297e-03 6.578408e-03 6.753974e-03 7.227289e-03 7.020763e-03 
##       I17.I2       I17.I4      I11.I17      I12.I17        I2.I4       I11.I2 
## 6.117068e-03 6.948449e-03 6.582642e-03 7.228804e-03 2.340873e-03 2.027323e-03 
##       I11.I4       I12.I2       I12.I4      I11.I12       I17.I2       I17.I4 
## 2.318969e-03 3.151003e-03 3.202352e-03 3.146007e-03 2.960353e-03 3.055866e-03 
##      I11.I17      I12.I17        I2.I4       I11.I2       I11.I4       I12.I2 
## 2.951100e-03 3.304767e-03 5.558679e-03 2.979706e-03 5.730119e-03 4.483914e-03 
##       I12.I4      I11.I12       I17.I2       I17.I4      I11.I17      I12.I17 
## 6.071726e-03 4.844999e-03 3.434983e-03 5.840058e-03 3.957150e-03 5.080948e-03 
##        I2.I4       I11.I2       I11.I4       I12.I2       I12.I4      I11.I12 
## 1.147047e-03 1.015494e-03 1.289720e-03 1.478112e-03 1.671287e-03 1.587735e-03 
##       I17.I2       I17.I4      I11.I17      I12.I17        I2.I4       I11.I2 
## 1.223783e-03 1.464262e-03 1.359420e-03 1.718747e-03 5.601603e-03 4.157713e-03 
##       I11.I4       I12.I2       I12.I4      I11.I12       I17.I2       I17.I4 
## 5.756375e-03 5.383598e-03 6.289671e-03 5.562786e-03 4.020106e-03 5.693448e-03 
##      I11.I17      I12.I17        I2.I4       I11.I2       I11.I4       I12.I2 
## 4.338010e-03 5.489872e-03 1.183974e-03 7.368213e-04 1.176949e-03 9.244692e-04 
##       I12.I4      I11.I12       I17.I2       I17.I4      I11.I17      I12.I17 
## 1.261618e-03 9.122765e-04 9.635703e-04 1.278485e-03 9.520916e-04 1.092652e-03 
##        I2.I4       I11.I2       I11.I4       I12.I2       I12.I4      I11.I12 
## 2.551300e-03 1.713121e-03 2.700355e-03 2.416821e-03 3.244471e-03 2.635775e-03 
##       I17.I2       I17.I4      I11.I17      I12.I17        I2.I4       I11.I2 
## 1.828924e-03 2.438745e-03 2.272319e-03 2.644191e-03 6.845350e-03 5.148546e-03 
##       I11.I4       I12.I2       I12.I4      I11.I12       I17.I2       I17.I4 
## 6.783531e-03 6.216018e-03 6.933699e-03 5.964926e-03 6.174315e-03 6.928057e-03 
##      I11.I17      I12.I17        I2.I4       I11.I2       I11.I4       I12.I2 
## 5.911159e-03 6.558212e-03 6.526385e-03 4.080360e-03 6.435437e-03 4.197747e-03 
##       I12.I4      I11.I12       I17.I2       I17.I4      I11.I17      I12.I17 
## 6.455137e-03 3.558014e-03 4.683726e-03 6.534204e-03 4.139681e-03 4.254995e-03 
##        I2.I4       I11.I2       I11.I4       I12.I2       I12.I4      I11.I12 
## 3.386974e-03 4.866016e-03 5.250308e-03 4.267879e-03 4.787532e-03 5.615892e-03 
##       I17.I2       I17.I4      I11.I17      I12.I17        I2.I4       I11.I2 
## 3.836405e-03 4.447625e-03 5.440556e-03 5.048393e-03 4.254981e-03 2.068709e-03 
##       I11.I4       I12.I2       I12.I4      I11.I12       I17.I2       I17.I4 
## 4.268260e-03 3.118857e-03 4.490570e-03 3.157381e-03 2.852257e-03 4.437717e-03 
##      I11.I17      I12.I17        I2.I4       I11.I2       I11.I4       I12.I2 
## 2.895877e-03 3.664186e-03 3.502504e-04 2.963654e-04 4.182100e-04 3.405000e-04 
##       I12.I4      I11.I12       I17.I2       I17.I4      I11.I17      I12.I17 
## 4.478701e-04 4.105529e-04 3.977713e-04 4.849735e-04 4.550316e-04 4.796672e-04 
##        I2.I4       I11.I2       I11.I4       I12.I2       I12.I4      I11.I12 
## 1.389771e-03 1.045266e-03 1.481819e-03 1.409647e-03 1.704541e-03 1.499334e-03 
##       I17.I2       I17.I4      I11.I17      I12.I17        I2.I4       I11.I2 
## 1.533014e-03 1.776384e-03 1.607589e-03 1.785682e-03 1.604108e-03 1.224203e-03 
##       I11.I4       I12.I2       I12.I4      I11.I12       I17.I2       I17.I4 
## 1.621387e-03 1.506093e-03 1.658960e-03 1.545009e-03 1.515672e-03 1.660180e-03 
##      I11.I17      I12.I17        I2.I4       I11.I2       I11.I4       I12.I2 
## 1.552498e-03 1.633409e-03 6.483343e-05 3.863057e-05 6.619376e-05 4.763780e-05 
##       I12.I4      I11.I12       I17.I2       I17.I4      I11.I17      I12.I17 
## 6.932505e-05 5.046547e-05 4.763780e-05 6.932505e-05 5.046547e-05 5.716704e-05 
##        I2.I4       I11.I2       I11.I4       I12.I2       I12.I4      I11.I12 
## 1.433027e-04 8.619418e-05 1.480101e-04 1.115258e-04 1.600060e-04 1.187348e-04 
##       I17.I2       I17.I4      I11.I17      I12.I17        I2.I4       I11.I2 
## 1.115258e-04 1.600060e-04 1.187348e-04 1.375763e-04 3.813117e-03 3.833274e-03 
##       I11.I4       I12.I2       I12.I4      I11.I12       I17.I2       I17.I4 
## 3.472033e-03 3.813117e-03 3.355913e-03 3.472033e-03 3.906250e-03 3.906250e-03 
##      I11.I17      I12.I17        I2.I4       I11.I2       I11.I4       I12.I2 
## 3.906250e-03 3.906250e-03 4.890944e-03 4.049430e-03 4.817385e-03 4.903898e-03 
##       I12.I4      I11.I12       I17.I2       I17.I4      I11.I17      I12.I17 
## 5.094963e-03 4.833560e-03 3.997407e-03 4.799844e-03 3.716858e-03 4.816784e-03 
##        I2.I4       I11.I2       I11.I4       I12.I2       I12.I4      I11.I12 
## 8.367243e-04 8.184589e-04 8.634833e-04 1.026275e-03 1.033865e-03 1.031140e-03 
##       I17.I2       I17.I4      I11.I17      I12.I17        I2.I4       I11.I2 
## 9.211711e-04 9.481838e-04 9.384538e-04 1.046134e-03 1.680103e-03 2.187068e-03 
##       I11.I4       I12.I2       I12.I4      I11.I12       I17.I2       I17.I4 
## 2.155450e-03 2.111281e-03 2.078190e-03 2.490842e-03 2.298585e-03 2.269220e-03 
##      I11.I17      I12.I17 
## 2.632582e-03 2.579530e-03
```

```
## New baseline model due to missing data
tas20.gender.baseline <- rma.mv(yi = Correlation,
                                V = V2, 
                                data = tas20[complete.cases(tas20$PropFemale),],
                                random = list(~ factor(Cell) | ESID,
                                              ~ factor(Cell) | StudyID),
                                struc = c("HCS", "CS"),
                                rho = 0,
                                phi = 0,
                                method = "REML",
                                mods = ~ factor(Cell) - 1,
                                time = TRUE,
                                sparse = TRUE,
                                control = list(optimizer = "optimParallel",
                                               ncpus = ncores))
```

```
## 
## Processing time: 0 hours, 0 minutes, 11.26 seconds
```

```
## Model summary
summary(tas20.gender.baseline)
```

```
## 
## Multivariate Meta-Analysis Model (k = 830; method: REML)
## 
##     logLik    Deviance         AIC         BIC        AICc   
##   706.8765  -1413.7531  -1371.7531  -1272.8577  -1370.5952   
## 
## Variance Components:
## 
## outer factor: ESID         (nlvls = 830)
## inner factor: factor(Cell) (nlvls = 10)
## 
##              estim    sqrt  k.lvl  fixed    level 
## tau^2.1     0.0009  0.0305     83     no  I11-I12 
## tau^2.2     0.0008  0.0279     83     no  I11-I17 
## tau^2.3     0.0021  0.0462     83     no  I12-I17 
## tau^2.4     0.0004  0.0197     83     no   I2-I11 
## tau^2.5     0.0021  0.0461     83     no   I2-I12 
## tau^2.6     0.0026  0.0513     83     no   I2-I17 
## tau^2.7     0.0128  0.1130     83     no    I2-I4 
## tau^2.8     0.0041  0.0643     83     no   I4-I11 
## tau^2.9     0.0054  0.0738     83     no   I4-I12 
## tau^2.10    0.0068  0.0824     83     no   I4-I17 
## rho         0.0000                   yes          
## 
## outer factor: StudyID      (nlvls = 58)
## inner factor: factor(Cell) (nlvls = 10)
## 
##             estim    sqrt  fixed 
## gamma^2    0.0044  0.0664     no 
## phi        0.0000            yes 
## 
## Test for Residual Heterogeneity:
## QE(df = 820) = 6641.4568, p-val < .0001
## 
## Test of Moderators (coefficients 1:10):
## QM(df = 10) = 6474.3920, p-val < .0001
## 
## Model Results:
## 
##                      estimate      se     zval    pval   ci.lb   ci.ub      
## factor(Cell)I11-I12    0.3061  0.0108  28.3015  <.0001  0.2849  0.3273  *** 
## factor(Cell)I11-I17    0.3415  0.0106  32.0769  <.0001  0.3206  0.3623  *** 
## factor(Cell)I12-I17    0.2746  0.0116  23.7438  <.0001  0.2519  0.2972  *** 
## factor(Cell)I2-I11     0.4397  0.0102  43.0598  <.0001  0.4197  0.4597  *** 
## factor(Cell)I2-I12     0.3429  0.0115  29.9025  <.0001  0.3205  0.3654  *** 
## factor(Cell)I2-I17     0.3800  0.0117  32.5529  <.0001  0.3572  0.4029  *** 
## factor(Cell)I2-I4      0.4119  0.0162  25.4181  <.0001  0.3801  0.4437  *** 
## factor(Cell)I4-I11     0.3392  0.0126  27.0238  <.0001  0.3146  0.3638  *** 
## factor(Cell)I4-I12     0.2709  0.0133  20.3807  <.0001  0.2448  0.2969  *** 
## factor(Cell)I4-I17     0.3084  0.0139  22.2522  <.0001  0.2813  0.3356  *** 
## 
## ---
## Signif. codes:  0 '***' 0.001 '**' 0.01 '*' 0.05 '.' 0.1 ' ' 1
```

```
## Cluster-robust standard errors
robust(tas20.gender.baseline, cluster = StudyID, clubSandwich = TRUE)
```

```
## 
## Multivariate Meta-Analysis Model (k = 830; method: REML)
## 
## Variance Components:
## 
## outer factor: ESID         (nlvls = 830)
## inner factor: factor(Cell) (nlvls = 10)
## 
##              estim    sqrt  k.lvl  fixed    level 
## tau^2.1     0.0009  0.0305     83     no  I11-I12 
## tau^2.2     0.0008  0.0279     83     no  I11-I17 
## tau^2.3     0.0021  0.0462     83     no  I12-I17 
## tau^2.4     0.0004  0.0197     83     no   I2-I11 
## tau^2.5     0.0021  0.0461     83     no   I2-I12 
## tau^2.6     0.0026  0.0513     83     no   I2-I17 
## tau^2.7     0.0128  0.1130     83     no    I2-I4 
## tau^2.8     0.0041  0.0643     83     no   I4-I11 
## tau^2.9     0.0054  0.0738     83     no   I4-I12 
## tau^2.10    0.0068  0.0824     83     no   I4-I17 
## rho         0.0000                   yes          
## 
## outer factor: StudyID      (nlvls = 58)
## inner factor: factor(Cell) (nlvls = 10)
## 
##             estim    sqrt  fixed 
## gamma^2    0.0044  0.0664     no 
## phi        0.0000            yes 
## 
## Test for Residual Heterogeneity:
## QE(df = 820) = 6641.4568, p-val < .0001
## 
## Number of estimates:   830
## Number of clusters:    58
## Estimates per cluster: 10-40 (mean: 14.31, median: 10)
## 
## Test of Moderators (coefficients 1:10):¹
## F(df1 = 10, df2 = 45.62) = 208.3266, p-val < .0001
## 
## Model Results:
## 
##                      estimate      se¹     tval¹     df¹    pval¹   ci.lb¹ 
## factor(Cell)I11-I12    0.3061  0.0130   23.6114   54.73   <.0001   0.2801  
## factor(Cell)I11-I17    0.3415  0.0129   26.4802   54.99   <.0001   0.3156  
## factor(Cell)I12-I17    0.2746  0.0150   18.2427   54.61   <.0001   0.2444  
## factor(Cell)I2-I11     0.4397  0.0107   41.0124    55.3   <.0001   0.4182  
## factor(Cell)I2-I12     0.3429  0.0138   24.9293    54.7   <.0001   0.3154  
## factor(Cell)I2-I17     0.3800  0.0140   27.1739   54.79   <.0001   0.3520  
## factor(Cell)I2-I4      0.4119  0.0186   22.1117   52.55   <.0001   0.3745  
## factor(Cell)I4-I11     0.3392  0.0149   22.8065   54.24   <.0001   0.3094  
## factor(Cell)I4-I12     0.2709  0.0170   15.9779   53.79   <.0001   0.2369  
## factor(Cell)I4-I17     0.3084  0.0175   17.6643   53.57   <.0001   0.2734  
##                       ci.ub¹      
## factor(Cell)I11-I12  0.3321   *** 
## factor(Cell)I11-I17  0.3673   *** 
## factor(Cell)I12-I17  0.3047   *** 
## factor(Cell)I2-I11   0.4612   *** 
## factor(Cell)I2-I12   0.3705   *** 
## factor(Cell)I2-I17   0.4081   *** 
## factor(Cell)I2-I4    0.4493   *** 
## factor(Cell)I4-I11   0.3690   *** 
## factor(Cell)I4-I12   0.3049   *** 
## factor(Cell)I4-I17   0.3435   *** 
## 
## ---
## Signif. codes:  0 '***' 0.001 '**' 0.01 '*' 0.05 '.' 0.1 ' ' 1
## 
## 1) results based on cluster-robust inference (var-cov estimator: CR2,
##    approx t/F-tests and confidence intervals, df: Satterthwaite approx)
```

```
## Variance explanation
## Proportional reduction of the variances

## Level: Primary studies
100*max((1-tas20.gender$gamma2/tas20.gender.baseline$gamma2), 0)
```

```
## [1] 12.41798
```

```
## Level: Effect sizes
100*max((1-tas20.gender$tau2[1]/tas20.gender.baseline$tau2[1]), 0)
```

```
## [1] 0
```

```
100*max((1-tas20.gender$tau2[2]/tas20.gender.baseline$tau2[2]), 0)
```

```
## [1] 0
```

```
100*max((1-tas20.gender$tau2[3]/tas20.gender.baseline$tau2[3]), 0)
```

```
## [1] 0
```

```
100*max((1-tas20.gender$tau2[4]/tas20.gender.baseline$tau2[4]), 0)
```

```
## [1] 0
```

```
100*max((1-tas20.gender$tau2[5]/tas20.gender.baseline$tau2[5]), 0)
```

```
## [1] 0
```

```
100*max((1-tas20.gender$tau2[6]/tas20.gender.baseline$tau2[6]), 0)
```

```
## [1] 0
```

```
100*max((1-tas20.gender$tau2[7]/tas20.gender.baseline$tau2[7]), 0)
```

```
## [1] 0
```

```
100*max((1-tas20.gender$tau2[8]/tas20.gender.baseline$tau2[8]), 0)
```

```
## [1] 0
```

```
100*max((1-tas20.gender$tau2[9]/tas20.gender.baseline$tau2[9]), 0)
```

```
## [1] 0
```

```
100*max((1-tas20.gender$tau2[10]/tas20.gender.baseline$tau2[10]), 0)
```

```
## [1] 0
```

# Meta-Analytic Structural Equation Modeling

The pooled correlation matrix and the respective asymptotic sampling
covariance matrix can then be submitted to structural equation modeling
to test theories, models, and hypotheses about the relations among
variables.

In this example, we test the extent to which a single-factor model
may represent the factor structure underlying the five indicators. We
use model `mlmvrem2` for these analyses.

```
## Model specification

## Single-factor model
## Model specification
SFM <- " 
          # Measurement model
          gF =~ Item2 + Item4 + Item11 + Item12 + Item17

"

## RAM specification of the model
RAM <- lavaan2RAM(SFM, 
                  obs.variables = c("Item2", "Item4", "Item11", 
                                    "Item12", "Item17"))

A <- RAM$A
S <- RAM$S
F <- RAM$F

checkRAM(A, S, cor.analysis = TRUE)

## Model estimation based on the correlation matrix from model 3
tas20.stage2.sfm <- wls(Cov = CORR.mlmvrem1, 
                        aCov = ACOV.mlmvrem1,
                        n = OverallN, 
                        Amatrix = A, 
                        Smatrix = S,
                        Fmatrix = F,
                        model.name = "Single factor model",
                        cor.analysis = TRUE,
                        diag.constraints = TRUE)

tas20.stage2.sfm <- rerun(tas20.stage2.sfm)
```

```
## Polite note from mxTryHard: Hessian not checked as model contains mxConstraints
```

```
## Running Single factor model with 10 parameters
```

```
## 
## Beginning initial fit attempt
```

```
## Running Single factor model with 10 parameters
```

```
## 
##  Lowest minimum so far:  3.00140471703666
```

```
## 
## Solution found
```

```
## Final run, for Hessian and/or standard errors and/or confidence intervals
```

```
## Running Single factor model with 10 parameters
```

```
## 
##  Solution found!  Final fit=3.0014047 (started at 3.0014047)  (1 attempt(s): 1 valid, 0 errors)
```

```
## Model summary
summary(tas20.stage2.sfm)
```

```
## 
## Call:
## wls(Cov = CORR.mlmvrem1, aCov = ACOV.mlmvrem1, n = OverallN, 
##     Amatrix = A, Smatrix = S, Fmatrix = F, diag.constraints = TRUE, 
##     cor.analysis = TRUE, model.name = "Single factor model")
## 
## 95% confidence intervals: z statistic approximation
## Coefficients:
##                  Estimate Std.Error   lbound   ubound z value  Pr(>|z|)    
## Item11ONgF       0.623861  0.010936 0.602426 0.645296  57.045 < 2.2e-16 ***
## Item12ONgF       0.495281  0.010905 0.473908 0.516653  45.420 < 2.2e-16 ***
## Item17ONgF       0.544133  0.011176 0.522228 0.566038  48.687 < 2.2e-16 ***
## Item2ONgF        0.719184  0.011542 0.696562 0.741806  62.309 < 2.2e-16 ***
## Item4ONgF        0.574495  0.010725 0.553475 0.595515  53.567 < 2.2e-16 ***
## Item11WITHItem11 0.610797  0.013645 0.584053 0.637542  44.762 < 2.2e-16 ***
## Item12WITHItem12 0.754697  0.010802 0.733526 0.775868  69.869 < 2.2e-16 ***
## Item17WITHItem17 0.703919  0.012163 0.680081 0.727758  57.875 < 2.2e-16 ***
## Item2WITHItem2   0.482775  0.016602 0.450235 0.515314  29.079 < 2.2e-16 ***
## Item4WITHItem4   0.669956  0.012323 0.645803 0.694108  54.367 < 2.2e-16 ***
## ---
## Signif. codes:  0 '***' 0.001 '**' 0.01 '*' 0.05 '.' 0.1 ' ' 1
## 
## Goodness-of-fit indices:
##                                                 Value
## Sample size                                69722.0000
## Chi-square of target model                     3.0014
## DF of target model                             5.0000
## p value of target model                        0.6998
## Number of constraints imposed on "Smatrix"     5.0000
## DF manually adjusted                           0.0000
## Chi-square of independence model            6948.5358
## DF of independence model                      10.0000
## RMSEA                                          0.0000
## RMSEA lower 95% CI                             0.0000
## RMSEA upper 95% CI                             0.0040
## SRMR                                           0.0066
## TLI                                            1.0006
## CFI                                            1.0000
## AIC                                           -6.9986
## BIC                                          -52.7600
## OpenMx status1: 0 ("0" or "1": The optimization is considered fine.
## Other values indicate problems.)
```

```
## Model plot
plot(tas20.stage2.sfm)
```

```
sfm.plot <- meta2semPlot(tas20.stage2.sfm)

##pdf(file = "SFM-mlmvrem1.pdf", height = 2, width = 3)
semPlot::semPaths(sfm.plot, 
                  whatLabels="est",
                  rotation = 1,
                  edge.label.cex = 1.1,
                  sizeMan = 8,
                  color = "grey",
                  layout = "tree2",
                  width = 9,
                  height = 6,
                  nCharNodes = 6)
```

```
##dev.off()
```

# R session info

```
sessionInfo()
```

```
## R version 4.4.3 (2025-02-28)
## Platform: x86_64-apple-darwin20
## Running under: macOS Ventura 13.7.4
## 
## Matrix products: default
## BLAS:   /Library/Frameworks/R.framework/Versions/4.4-x86_64/Resources/lib/libRblas.0.dylib 
## LAPACK: /Library/Frameworks/R.framework/Versions/4.4-x86_64/Resources/lib/libRlapack.dylib;  LAPACK version 3.12.0
## 
## locale:
## [1] en_US.UTF-8/en_US.UTF-8/en_US.UTF-8/C/en_US.UTF-8/en_US.UTF-8
## 
## time zone: Europe/Oslo
## tzcode source: internal
## 
## attached base packages:
## [1] parallel  stats     graphics  grDevices utils     datasets  methods  
## [8] base     
## 
## other attached packages:
##  [1] lattice_0.22-6      semPlot_1.1.6       optimParallel_1.0-2
##  [4] dplyr_1.1.4         corrplot_0.94       clubSandwich_0.5.11
##  [7] robumeta_2.1        metafor_4.8-0       numDeriv_2016.8-1.1
## [10] metadat_1.4-0       Matrix_1.7-2        metaSEM_1.5.0      
## [13] OpenMx_2.21.12      psych_2.4.6.26      pacman_0.5.1       
## 
## loaded via a namespace (and not attached):
##  [1] mnormt_2.1.1       pbapply_1.7-2      gridExtra_2.3      fdrtool_1.2.18    
##  [5] sandwich_3.1-1     rlang_1.1.5        magrittr_2.0.3     rockchalk_1.8.157 
##  [9] compiler_4.4.3     png_0.1-8          vctrs_0.6.5        reshape2_1.4.4    
## [13] quadprog_1.5-8     stringr_1.5.1      pkgconfig_2.0.3    fastmap_1.2.0     
## [17] arm_1.14-4         backports_1.5.0    pbivnorm_0.6.0     rmarkdown_2.29    
## [21] nloptr_2.1.1       xfun_0.51          cachem_1.1.0       kutils_1.73       
## [25] jsonlite_1.9.1     jpeg_0.1-10        lavaan_0.6-19      cluster_2.1.8     
## [29] R6_2.6.1           bslib_0.9.0        stringi_1.8.4      boot_1.3-31       
## [33] rpart_4.1.24       jquerylib_0.1.4    Rcpp_1.0.13-1      knitr_1.49        
## [37] zoo_1.8-12         base64enc_0.1-3    splines_4.4.3      nnet_7.3-20       
## [41] igraph_2.0.3       tidyselect_1.2.1   rstudioapi_0.17.1  abind_1.4-8       
## [45] yaml_2.3.10        codetools_0.2-20   qgraph_1.9.8       tibble_3.2.1      
## [49] plyr_1.8.9         coda_0.19-4.1      evaluate_1.0.3     foreign_0.8-89    
## [53] RcppParallel_5.1.9 zip_2.3.1          pillar_1.10.1      carData_3.0-5     
## [57] checkmate_2.3.2    stats4_4.4.3       ellipse_0.5.0      generics_0.1.3    
## [61] mathjaxr_1.6-0     ggplot2_3.5.1      munsell_0.5.1      scales_1.3.0      
## [65] minqa_1.2.8        gtools_3.9.5       xtable_1.8-4       glue_1.8.0        
## [69] mi_1.1             Hmisc_5.1-3        tools_4.4.3        data.table_1.17.0 
## [73] lme4_1.1-35.5      openxlsx_4.2.7.1   mvtnorm_1.3-3      XML_3.99-0.17     
## [77] grid_4.4.3         sem_3.1-16         matrixcalc_1.0-6   colorspace_2.1-1  
## [81] nlme_3.1-166       htmlTable_2.4.3    Formula_1.2-5      cli_3.6.4         
## [85] corpcor_1.6.10     glasso_1.11        gtable_0.3.6       sass_0.4.9        
## [89] digest_0.6.37      htmlwidgets_1.6.4  htmltools_0.5.8.1  lifecycle_1.0.4   
## [93] lisrelToR_0.3      MASS_7.3-61
```
