# Supplementary material for: Meta-analyzing correlation matrices in the presence of hierarchical effect size multiplicity
Source: Res Synth Methods. 2025 Aug 7;16(6):828–58. doi: 10.1017/rsm.2025.10027 (PMC12657669; doi:10.1017/rsm.2025.10027)
Supplement: Scherer and Campos supplementary material [file S1759287925100276sup001.zip › S1759287925100276sup001/S3-Supplementary-Material.html]

Supplementary Material


# Supplementary Material

#### Ilustrative Example: Factor Structure of the TAS-20 Subscale Measuring Difficulties Describing Feelings (DDF) - Analyses with Fisher’s r-to-Z Transformation

#### 28 March 2025

# Purpose, Content, and Context of this Illustrative Example

With this data example, we illustrate how the multilevel,
multivariate, and random-effects approach can be implemented in the R
package `metafor`. Specifically, we provide the analytic code
for specifying and estimating a series of models that accommodate both
the dependencies among multiple correlation coefficients and the
hierarchical structure of the data (e.g., with multiple correlation
coefficients nested in primary studies or study samples).

In this example, we specify block-diagonal sampling covariance
matrices of the correlation matrices of our interest. We essentially
assume that each correlation within a correlation matrix has its own
sampling variance, and effect sizes within a correlation matrix are
dependent. This is a **sampling covariance dependence
structure**.

In this structure, we construct the sampling variance-covariance
matrices, following the equations by Steiger (1980) and
Olkin and Finn
(1990), which have been implemented by Wolfgang Viechtbauer in the R
package `metafor`.

In the following analyses, we Z-transform the Pearson correlation
coefficients (r).

# Meta-Analytic Data

## Source and Acknowledgement

We chose a subset of the data published by Schroeders et
al. (2022), which contains item-item correlations and/or the
elements used to retrieve them from the Toronto Alexithymia Scale
(TAS-20). At this point, we wish to acknowledge the originality of
Schroeders et al.’s works and do not claim any credit for the work
behind generating this data set.

For illustrative purposes, we chose the data focusing on the
dimension DDF, that is, participants’ self-reported difficulties with
describing feelings. This dimension is measured by the following
indicators (see Schroeders et al. (2022)):

- Item 2 (I2): It is difficult for me to find the right words for my
  feelings.
- Item 4 (I4): I am able to describe my feelings easily.
  (reverse-coded)
- Item 11 (I11): I find it hard to describe how I feel about
  people.
- Item 12 (I12): People tell me to describe my feelings more.
- Item 17 (I17): It is difficult for me to reveal my innermost
  feeling, even to close friends.

## Install and load relevant `R` packages

```
# Install R packages (if needed)
# install.packages("pacman")
library(pacman)
pacman::p_load(psych,
               metaSEM,
               metafor,
               robumeta,
               clubSandwich,
               corrplot,
               dplyr,
               psych,
               optimParallel,
               semPlot,
               lattice)

## Number of available cores
ncores <- parallel::detectCores() - 1
```

## Data Input and Preparation

```
## Data set in wide format
tas20 <- read.csv2("TAS20-Data.csv", header = TRUE)
## Create a new effect size ID
tas20$ESID <- seq_len(nrow(tas20))
## Check the data
head(tas20, 10)
```

```
##    Reference SampleID    Cell Correlation StudyID PubYear Country PubType   N
## 1  aluja2020       83   I2-I4      0.1402      57    2020   Spain       1 602
## 2  aluja2020       83  I2-I11      0.3714      57    2020   Spain       1 602
## 3  aluja2020       83  I4-I11      0.2137      57    2020   Spain       1 602
## 4  aluja2020       83  I2-I12      0.3091      57    2020   Spain       1 602
## 5  aluja2020       83  I4-I12      0.2369      57    2020   Spain       1 602
## 6  aluja2020       83 I11-I12      0.4198      57    2020   Spain       1 602
## 7  aluja2020       83  I2-I17      0.3320      57    2020   Spain       1 602
## 8  aluja2020       83  I4-I17      0.2532      57    2020   Spain       1 602
## 9  aluja2020       83 I11-I17      0.4574      57    2020   Spain       1 602
## 10 aluja2020       83 I12-I17      0.4146      57    2020   Spain       1 602
##    PropFemale MeanAge Clinical Var1 Var2 ESID
## 1       25.25    52.8        0   I2   I4    1
## 2       25.25    52.8        0   I2  I11    2
## 3       25.25    52.8        0   I4  I11    3
## 4       25.25    52.8        0   I2  I12    4
## 5       25.25    52.8        0   I4  I12    5
## 6       25.25    52.8        0  I11  I12    6
## 7       25.25    52.8        0   I2  I17    7
## 8       25.25    52.8        0   I4  I17    8
## 9       25.25    52.8        0  I11  I17    9
## 10      25.25    52.8        0  I12  I17   10
```

## Convenience Functions for the Transformations

```
## Create a function for the z-to-r transformation
ztor <- function(fisherz){
  r <- (exp(2*fisherz)-1)/(exp(2*fisherz)+1)
  return(r)
}

## Create a function for the r-to-z transformation
rtoz <- function(pearsonr){
  zr <- 0.5*log((1+pearsonr)/(1-pearsonr))
  return(zr)
}

## Output after back-transformation
outztor <- function(workingmodel){
  output <- ztor(cbind.data.frame(workingmodel$beta, 
                                  workingmodel$se, 
                                  workingmodel$ci.lb, 
                                  workingmodel$ci.ub))
  colnames(output) <- c("Estimate", "SE", "CI95low", "CI95upp")
  return(output)
}

## Add the z value to the data set
tas20$z <- rtoz(tas20$Correlation)

## Add the sampling variance z to the data set
tas20$vz <- 1/(tas20$N - 3)
```

# Description of the Meta-Analytic Dataset

In the following section, we describe the meta-analytic sample by
providing the number of effect sizes and articles, along with some
characteristics of the samples.

```
## Number of effect sizes
nrow(tas20)
```

```
## [1] 880
```

```
## Number of articles
length(table(tas20$StudyID))
```

```
## [1] 62
```

```
table(tas20$StudyID)
```

```
## 
##  1  2  3  4  5  6  7  8  9 10 11 12 13 14 15 16 17 18 19 20 21 22 23 24 25 26 
## 30 30 10 20 10 10 10 10 10 10 10 20 10 10 10 10 20 10 20 20 10 10 10 20 20 20 
## 27 28 29 30 31 32 33 34 35 36 37 38 39 40 41 42 43 44 45 46 47 48 49 50 51 52 
## 10 10 20 10 10 10 10 10 20 20 20 10 20 10 10 20 40 10 10 10 20 10 10 10 20 20 
## 53 54 55 56 57 58 59 60 61 62 
## 10 10 20 20 10 10 10 10 10 10
```

```
## Average number of correlation matrices per study
mean(table(tas20$StudyID)/10)
```

```
## [1] 1.419355
```

```
sd(table(tas20$StudyID)/10)
```

```
## [1] 0.6414223
```

```
median(table(tas20$StudyID)/10)
```

```
## [1] 1
```

```
min(table(tas20$StudyID)/10)
```

```
## [1] 1
```

```
max(table(tas20$StudyID)/10)
```

```
## [1] 4
```

```
## Number of samples
length(table(tas20$SampleID))
```

```
## [1] 88
```

```
table(tas20$SampleID)
```

```
## 
##  1  2  3  4  5  6  7  8  9 10 11 12 13 14 15 16 17 18 19 20 21 22 23 24 25 26 
## 10 10 10 10 10 10 10 10 10 10 10 10 10 10 10 10 10 10 10 10 10 10 10 10 10 10 
## 27 28 29 30 31 32 33 34 35 36 37 38 39 40 41 42 43 44 45 46 47 48 49 50 51 52 
## 10 10 10 10 10 10 10 10 10 10 10 10 10 10 10 10 10 10 10 10 10 10 10 10 10 10 
## 53 54 55 56 57 58 59 60 61 62 63 64 65 66 67 68 69 70 71 72 73 74 75 76 77 78 
## 10 10 10 10 10 10 10 10 10 10 10 10 10 10 10 10 10 10 10 10 10 10 10 10 10 10 
## 79 80 81 82 83 84 85 86 87 88 
## 10 10 10 10 10 10 10 10 10 10
```

```
## Overall sample size
OverallN <- round(round(sum(aggregate(tas20$N, 
                                      by = list(tas20$SampleID),
                                      FUN = mean)$x, 
                            na.rm = TRUE), 
                        0))
OverallN
```

```
## [1] 69722
```

```
## Distribution of sample sizes
psych::describe(tas20$N)
```

```
##    vars   n  mean      sd median trimmed    mad min   max range skew kurtosis
## X1    1 880 792.3 1508.81    327  493.85 247.59  99 12706 12607 5.94     42.1
##       se
## X1 50.86
```

```
hist(tas20$N,
     xlab = "Sample size",
     main = "Distribution of the study sample sizes")
```

```
## Number of types of correlations
length(table(tas20$Cell))
```

```
## [1] 10
```

```
## Correlation coefficients
psych::describe(tas20$Correlation)
```

```
##    vars   n mean   sd median trimmed  mad   min  max range  skew kurtosis se
## X1    1 880 0.35 0.14   0.36    0.36 0.13 -0.19 0.73  0.92 -0.33     0.45  0
```

```
hist(tas20$Correlation,
     xlab = "Pearson correlation coefficient",
     main = "Distribution of the correlations")
```

```
## Types of correlations
table(tas20$Cell)
```

```
## 
## I11-I12 I11-I17 I12-I17  I2-I11  I2-I12  I2-I17   I2-I4  I4-I11  I4-I12  I4-I17 
##      88      88      88      88      88      88      88      88      88      88
```

```
round(prop.table(table(tas20$Cell))*100,2)
```

```
## 
## I11-I12 I11-I17 I12-I17  I2-I11  I2-I12  I2-I17   I2-I4  I4-I11  I4-I12  I4-I17 
##      10      10      10      10      10      10      10      10      10      10
```

# Constructing Sampling Covariance Matrices

As a first step, we construct the sampling variance-covariance
matrices. These matrices contain the known sampling variances in the
diagonal and, if meta-analysts decide to account for the dependencies
among sampling errors, the sampling covariances in the off-diagonal
part.

```
## Generate the sampling covariance matrices
## Upper level: Correlation matrices (independent samples)
## Note: Each sample contributes only one correlation matrix.
## Hence, SampleID identifies the correlation matrix.

## rcalc() in metafor
## Source: https://wviechtb.github.io/metafor/reference/rcalc.html
SampCov <- metafor::rcalc(Correlation ~ Var1 + Var2 | SampleID,
                          ni = N,
                          data = tas20,
                          rtoz = TRUE)

## Extract and save the sampling variance-covariance matrix
## Note: This is the sampling covariance matrix for the transformed z values.
V <- SampCov$V

## Illustrate some variances for some samples
blsplit(V, tas20$SampleID)$`1`
```

```
##                I2.I4       I11.I2       I11.I4       I12.I2       I12.I4
## I2.I4   0.0033003300 0.0011338516 0.0011937783 0.0010759643 0.0011324192
## I11.I2  0.0011338516 0.0033003300 0.0016530653 0.0008820646 0.0006503944
## I11.I4  0.0011937783 0.0016530653 0.0033003300 0.0006498645 0.0009022850
## I12.I2  0.0010759643 0.0008820646 0.0006498645 0.0033003300 0.0016862211
## I12.I4  0.0011324192 0.0006503944 0.0009022850 0.0016862211 0.0033003300
## I11.I12 0.0006730529 0.0012755967 0.0012404854 0.0013729952 0.0013351040
## I17.I2  0.0008879822 0.0007308384 0.0005519977 0.0006951883 0.0005299732
## I17.I4  0.0009335844 0.0005536978 0.0007474266 0.0005311724 0.0007109338
## I11.I17 0.0005809183 0.0010523801 0.0010240724 0.0004826930 0.0004775778
## I12.I17 0.0005625817 0.0004872803 0.0004817237 0.0010757553 0.0010469070
##              I11.I12       I17.I2       I17.I4      I11.I17      I12.I17
## I2.I4   0.0006730529 0.0008879822 0.0009335844 0.0005809183 0.0005625817
## I11.I2  0.0012755967 0.0007308384 0.0005536978 0.0010523801 0.0004872803
## I11.I4  0.0012404854 0.0005519977 0.0007474266 0.0010240724 0.0004817237
## I12.I2  0.0013729952 0.0006951883 0.0005311724 0.0004826930 0.0010757553
## I12.I4  0.0013351040 0.0005299732 0.0007109338 0.0004775778 0.0010469070
## I11.I12 0.0033003300 0.0004750284 0.0004710579 0.0008019448 0.0008622261
## I17.I2  0.0004750284 0.0033003300 0.0017785157 0.0014551118 0.0013823129
## I17.I4  0.0004710579 0.0017785157 0.0033003300 0.0014157228 0.0013450917
## I11.I17 0.0008019448 0.0014551118 0.0014157228 0.0033003300 0.0011068742
## I12.I17 0.0008622261 0.0013823129 0.0013450917 0.0011068742 0.0033003300
```

```
blsplit(V, tas20$SampleID)$`10`
```

```
##                I2.I4       I11.I2       I11.I4       I12.I2       I12.I4
## I2.I4   0.0035460993 0.0007497473 0.0008207778 0.0003449813 0.0003775825
## I11.I2  0.0007497473 0.0035460993 0.0006801295 0.0003998410 0.0001401992
## I11.I4  0.0008207778 0.0006801295 0.0035460993 0.0001395245 0.0004070601
## I12.I2  0.0003449813 0.0003998410 0.0001395245 0.0035460993 0.0007634847
## I12.I4  0.0003775825 0.0001401992 0.0004070601 0.0007634847 0.0035460993
## I11.I12 0.0001389834 0.0003628038 0.0003374647 0.0008849833 0.0008231198
## I17.I2  0.0005160090 0.0005983487 0.0002030918 0.0002757285 0.0001024102
## I17.I4  0.0005648163 0.0002037459 0.0006090538 0.0001022457 0.0002807260
## I11.I17 0.0002026913 0.0005428555 0.0005048233 0.0001145265 0.0001083434
## I12.I17 0.0001033664 0.0001163843 0.0001095711 0.0006101505 0.0005675733
##              I11.I12       I17.I2       I17.I4      I11.I17      I12.I17
## I2.I4   0.0001389834 0.0005160090 0.0005648163 0.0002026913 0.0001033664
## I11.I2  0.0003628038 0.0005983487 0.0002037459 0.0005428555 0.0001163843
## I11.I4  0.0003374647 0.0002030918 0.0006090538 0.0005048233 0.0001095711
## I12.I2  0.0008849833 0.0002757285 0.0001022457 0.0001145265 0.0006101505
## I12.I4  0.0008231198 0.0001024102 0.0002807260 0.0001083434 0.0005675733
## I11.I12 0.0035460993 0.0001143077 0.0001079627 0.0002697119 0.0006577556
## I17.I2  0.0001143077 0.0035460993 0.0007359161 0.0008534619 0.0003931111
## I17.I4  0.0001079627 0.0007359161 0.0035460993 0.0007935889 0.0003656829
## I11.I17 0.0002697119 0.0008534619 0.0007935889 0.0035460993 0.0004237775
## I12.I17 0.0006577556 0.0003931111 0.0003656829 0.0004237775 0.0035460993
```

# Pooling Correlation Matrices Meta-Analytically

As a next step, we pooled the correlation matrices via several
multilevel, multivariate, and random-effects models. These models
contain different assumptions on the within- and between-study random
effects and hence the variance estimates in these models. In other
words, these models quantify heterogeneity in the meta-analytic data
under different assumptions.

## Multilevel Multivariate Random-Effects Models Accounting for Hierarchical Effect Size Multiplicity

### Model with level-specific variance estimates (`Model 1`)

This model assumes that each type of pooled correlation has its own
set of estimates of the amounts of residual heterogeneity within and
between studies (`mlmvrem1`).

The structure of random effects follows a heteroscedastic compound
symmetry (`HCS`) structure. We vary the \(\rho\) and \(\phi\) parameters in this structure, using
values of 0, 0.5, and 1 to study the sensitivity of parameter choice. In
our view, selecting values of these correlations should be informed by
substantive reasons, theories, and evidence.

```
## Random effects: rho = 0, phi = 0
## Source: https://wviechtb.github.io/metafor/reference/rma.mv.html

## Model specification
tas20.mlmvrem1 <- rma.mv(z,
                         V, 
                         data = tas20,
                         random = list(~ factor(Cell) | ESID,
                                       ~ factor(Cell) | StudyID),
                         struc = c("HCS", "HCS"),
                         rho = 0,
                         phi = 0,
                         method = "REML",
                         mods = ~ factor(Cell) - 1,
                         time = TRUE,
                         sparse = TRUE,
                         control = list(optimizer = "optimParallel",
                                        ncpus = ncores))
```

```
## 
## Processing time: 0 hours, 0 minutes, 8.68 seconds
```

```
## Model summary
summary(tas20.mlmvrem1)
```

```
## 
## Multivariate Meta-Analysis Model (k = 880; method: REML)
## 
##     logLik    Deviance         AIC         BIC        AICc   
##   620.1644  -1240.3287  -1180.3287  -1037.2739  -1178.1118   
## 
## Variance Components:
## 
## outer factor: ESID         (nlvls = 880)
## inner factor: factor(Cell) (nlvls = 10)
## 
##              estim    sqrt  k.lvl  fixed    level 
## tau^2.1     0.0010  0.0316     88     no  I11-I12 
## tau^2.2     0.0008  0.0286     88     no  I11-I17 
## tau^2.3     0.0016  0.0404     88     no  I12-I17 
## tau^2.4     0.0006  0.0253     88     no   I2-I11 
## tau^2.5     0.0025  0.0503     88     no   I2-I12 
## tau^2.6     0.0030  0.0549     88     no   I2-I17 
## tau^2.7     0.0133  0.1155     88     no    I2-I4 
## tau^2.8     0.0041  0.0643     88     no   I4-I11 
## tau^2.9     0.0033  0.0577     88     no   I4-I12 
## tau^2.10    0.0056  0.0747     88     no   I4-I17 
## rho         0.0000                   yes          
## 
## outer factor: StudyID      (nlvls = 62)
## inner factor: factor(Cell) (nlvls = 10)
## 
##                estim    sqrt  k.lvl  fixed    level 
## gamma^2.1     0.0054  0.0734     88     no  I11-I12 
## gamma^2.2     0.0057  0.0753     88     no  I11-I17 
## gamma^2.3     0.0069  0.0831     88     no  I12-I17 
## gamma^2.4     0.0058  0.0765     88     no   I2-I11 
## gamma^2.5     0.0061  0.0779     88     no   I2-I12 
## gamma^2.6     0.0064  0.0799     88     no   I2-I17 
## gamma^2.7     0.0137  0.1172     88     no    I2-I4 
## gamma^2.8     0.0081  0.0898     88     no   I4-I11 
## gamma^2.9     0.0109  0.1044     88     no   I4-I12 
## gamma^2.10    0.0096  0.0979     88     no   I4-I17 
## phi           0.0000                   yes          
## 
## Test for Residual Heterogeneity:
## QE(df = 870) = 6548.1258, p-val < .0001
## 
## Test of Moderators (coefficients 1:10):
## QM(df = 10) = 5526.0637, p-val < .0001
## 
## Model Results:
## 
##                      estimate      se     zval    pval   ci.lb   ci.ub      
## factor(Cell)I11-I12    0.3201  0.0116  27.6194  <.0001  0.2973  0.3428  *** 
## factor(Cell)I11-I17    0.3561  0.0117  30.4445  <.0001  0.3331  0.3790  *** 
## factor(Cell)I12-I17    0.2832  0.0130  21.8577  <.0001  0.2578  0.3086  *** 
## factor(Cell)I2-I11     0.4784  0.0117  40.7987  <.0001  0.4554  0.5013  *** 
## factor(Cell)I2-I12     0.3627  0.0128  28.2527  <.0001  0.3375  0.3878  *** 
## factor(Cell)I2-I17     0.4019  0.0133  30.2822  <.0001  0.3758  0.4279  *** 
## factor(Cell)I2-I4      0.4524  0.0206  21.9535  <.0001  0.4120  0.4928  *** 
## factor(Cell)I4-I11     0.3614  0.0148  24.4720  <.0001  0.3325  0.3904  *** 
## factor(Cell)I4-I12     0.2842  0.0160  17.8007  <.0001  0.2529  0.3155  *** 
## factor(Cell)I4-I17     0.3223  0.0162  19.9447  <.0001  0.2907  0.3540  *** 
## 
## ---
## Signif. codes:  0 '***' 0.001 '**' 0.01 '*' 0.05 '.' 0.1 ' ' 1
```

```
outztor(tas20.mlmvrem1)
```

```
##                      Estimate         SE   CI95low   CI95upp
## factor(Cell)I11-I12 0.3095589 0.01158762 0.2888811 0.3299479
## factor(Cell)I11-I17 0.3417420 0.01169495 0.3213401 0.3618268
## factor(Cell)I12-I17 0.2758679 0.01295601 0.2522454 0.2991618
## factor(Cell)I2-I11  0.4449314 0.01172441 0.4263132 0.4631729
## factor(Cell)I2-I12  0.3475521 0.01283547 0.3252422 0.3694753
## factor(Cell)I2-I17  0.3815352 0.01326955 0.3590943 0.4035351
## factor(Cell)I2-I4   0.4238931 0.02060554 0.3902005 0.4564519
## factor(Cell)I4-I11  0.3464655 0.01476772 0.3207431 0.3716773
## factor(Cell)I4-I12  0.2767895 0.01596442 0.2476517 0.3054271
## factor(Cell)I4-I17  0.3116177 0.01616011 0.2827423 0.3399289
```

```
## Cluster-robust standard errors
tas20.mlmvrem1.robust <- robust(tas20.mlmvrem1, 
                                cluster = StudyID, 
                                clubSandwich = TRUE)
summary(tas20.mlmvrem1.robust)
```

```
## 
## Multivariate Meta-Analysis Model (k = 880; method: REML)
## 
##     logLik    Deviance         AIC         BIC        AICc   
##   620.1644  -1240.3287  -1180.3287  -1037.2739  -1178.1118   
## 
## Variance Components:
## 
## outer factor: ESID         (nlvls = 880)
## inner factor: factor(Cell) (nlvls = 10)
## 
##              estim    sqrt  k.lvl  fixed    level 
## tau^2.1     0.0010  0.0316     88     no  I11-I12 
## tau^2.2     0.0008  0.0286     88     no  I11-I17 
## tau^2.3     0.0016  0.0404     88     no  I12-I17 
## tau^2.4     0.0006  0.0253     88     no   I2-I11 
## tau^2.5     0.0025  0.0503     88     no   I2-I12 
## tau^2.6     0.0030  0.0549     88     no   I2-I17 
## tau^2.7     0.0133  0.1155     88     no    I2-I4 
## tau^2.8     0.0041  0.0643     88     no   I4-I11 
## tau^2.9     0.0033  0.0577     88     no   I4-I12 
## tau^2.10    0.0056  0.0747     88     no   I4-I17 
## rho         0.0000                   yes          
## 
## outer factor: StudyID      (nlvls = 62)
## inner factor: factor(Cell) (nlvls = 10)
## 
##                estim    sqrt  k.lvl  fixed    level 
## gamma^2.1     0.0054  0.0734     88     no  I11-I12 
## gamma^2.2     0.0057  0.0753     88     no  I11-I17 
## gamma^2.3     0.0069  0.0831     88     no  I12-I17 
## gamma^2.4     0.0058  0.0765     88     no   I2-I11 
## gamma^2.5     0.0061  0.0779     88     no   I2-I12 
## gamma^2.6     0.0064  0.0799     88     no   I2-I17 
## gamma^2.7     0.0137  0.1172     88     no    I2-I4 
## gamma^2.8     0.0081  0.0898     88     no   I4-I11 
## gamma^2.9     0.0109  0.1044     88     no   I4-I12 
## gamma^2.10    0.0096  0.0979     88     no   I4-I17 
## phi           0.0000                   yes          
## 
## Test for Residual Heterogeneity:
## QE(df = 870) = 6548.1258, p-val < .0001
## 
## Number of estimates:   880
## Number of clusters:    62
## Estimates per cluster: 10-40 (mean: 14.19, median: 10)
## 
## Test of Moderators (coefficients 1:10):¹
## F(df1 = 10, df2 = 50.05) = 126.9511, p-val < .0001
## 
## Model Results:
## 
##                      estimate      se¹     tval¹     df¹    pval¹   ci.lb¹ 
## factor(Cell)I11-I12    0.3201  0.0145   22.0633   58.27   <.0001   0.2910  
## factor(Cell)I11-I17    0.3561  0.0145   24.5906   58.42   <.0001   0.3271  
## factor(Cell)I12-I17    0.2832  0.0161   17.6353   58.93   <.0001   0.2511  
## factor(Cell)I2-I11     0.4784  0.0143   33.5484   58.51   <.0001   0.4498  
## factor(Cell)I2-I12     0.3627  0.0161   22.5642   58.52   <.0001   0.3305  
## factor(Cell)I2-I17     0.4019  0.0162   24.7467    58.6   <.0001   0.3694  
## factor(Cell)I2-I4      0.4524  0.0233   19.4452   58.97   <.0001   0.4059  
## factor(Cell)I4-I11     0.3614  0.0175   20.6881   59.04   <.0001   0.3265  
## factor(Cell)I4-I12     0.2842  0.0189   15.0297   59.75   <.0001   0.2464  
## factor(Cell)I4-I17     0.3223  0.0192   16.8188   59.23   <.0001   0.2840  
##                       ci.ub¹      
## factor(Cell)I11-I12  0.3491   *** 
## factor(Cell)I11-I17  0.3850   *** 
## factor(Cell)I12-I17  0.3153   *** 
## factor(Cell)I2-I11   0.5069   *** 
## factor(Cell)I2-I12   0.3948   *** 
## factor(Cell)I2-I17   0.4344   *** 
## factor(Cell)I2-I4    0.4990   *** 
## factor(Cell)I4-I11   0.3964   *** 
## factor(Cell)I4-I12   0.3220   *** 
## factor(Cell)I4-I17   0.3607   *** 
## 
## ---
## Signif. codes:  0 '***' 0.001 '**' 0.01 '*' 0.05 '.' 0.1 ' ' 1
## 
## 1) results based on cluster-robust inference (var-cov estimator: CR2,
##    approx t/F-tests and confidence intervals, df: Satterthwaite approx)
```

```
## Sensitivity analyses
## Rho = Phi = 0.5
## Model specification
tas20.mlmvrem1.sens <- rma.mv(z,
                              V, 
                              data = tas20,
                              random = list(~ factor(Cell) | ESID,
                                            ~ factor(Cell) | StudyID),
                              struc = c("HCS", "HCS"),
                              rho = 0.5,
                              phi = 0.5,
                              method = "REML",
                              mods = ~ factor(Cell) - 1,
                              time = TRUE,
                              sparse = TRUE,
                              control = list(optimizer = "optimParallel",
                                             ncpus = ncores))
```

```
## 
## Processing time: 0 hours, 0 minutes, 29.29 seconds
```

```
## Model summary
summary(tas20.mlmvrem1.sens)
```

```
## 
## Multivariate Meta-Analysis Model (k = 880; method: REML)
## 
##     logLik    Deviance         AIC         BIC        AICc   
##   794.4902  -1588.9804  -1528.9804  -1385.9257  -1526.7635   
## 
## Variance Components:
## 
## outer factor: ESID         (nlvls = 880)
## inner factor: factor(Cell) (nlvls = 10)
## 
##              estim    sqrt  k.lvl  fixed    level 
## tau^2.1     0.0005  0.0231     88     no  I11-I12 
## tau^2.2     0.0005  0.0219     88     no  I11-I17 
## tau^2.3     0.0008  0.0282     88     no  I12-I17 
## tau^2.4     0.0004  0.0198     88     no   I2-I11 
## tau^2.5     0.0014  0.0375     88     no   I2-I12 
## tau^2.6     0.0018  0.0422     88     no   I2-I17 
## tau^2.7     0.0084  0.0915     88     no    I2-I4 
## tau^2.8     0.0022  0.0469     88     no   I4-I11 
## tau^2.9     0.0022  0.0471     88     no   I4-I12 
## tau^2.10    0.0029  0.0541     88     no   I4-I17 
## rho         0.5000                   yes          
## 
## outer factor: StudyID      (nlvls = 62)
## inner factor: factor(Cell) (nlvls = 10)
## 
##                estim    sqrt  k.lvl  fixed    level 
## gamma^2.1     0.0056  0.0747     88     no  I11-I12 
## gamma^2.2     0.0056  0.0751     88     no  I11-I17 
## gamma^2.3     0.0070  0.0834     88     no  I12-I17 
## gamma^2.4     0.0057  0.0754     88     no   I2-I11 
## gamma^2.5     0.0065  0.0808     88     no   I2-I12 
## gamma^2.6     0.0066  0.0811     88     no   I2-I17 
## gamma^2.7     0.0148  0.1215     88     no    I2-I4 
## gamma^2.8     0.0087  0.0931     88     no   I4-I11 
## gamma^2.9     0.0098  0.0992     88     no   I4-I12 
## gamma^2.10    0.0101  0.1005     88     no   I4-I17 
## phi           0.5000                   yes          
## 
## Test for Residual Heterogeneity:
## QE(df = 870) = 6548.1258, p-val < .0001
## 
## Test of Moderators (coefficients 1:10):
## QM(df = 10) = 2279.5484, p-val < .0001
## 
## Model Results:
## 
##                      estimate      se     zval    pval   ci.lb   ci.ub      
## factor(Cell)I11-I12    0.3274  0.0115  28.5446  <.0001  0.3049  0.3499  *** 
## factor(Cell)I11-I17    0.3624  0.0115  31.5413  <.0001  0.3399  0.3849  *** 
## factor(Cell)I12-I17    0.2895  0.0126  23.0229  <.0001  0.2648  0.3141  *** 
## factor(Cell)I2-I11     0.4852  0.0115  42.3155  <.0001  0.4628  0.5077  *** 
## factor(Cell)I2-I12     0.3706  0.0126  29.4167  <.0001  0.3459  0.3952  *** 
## factor(Cell)I2-I17     0.4084  0.0128  31.8667  <.0001  0.3833  0.4335  *** 
## factor(Cell)I2-I4      0.4601  0.0195  23.5704  <.0001  0.4218  0.4984  *** 
## factor(Cell)I4-I11     0.3710  0.0143  25.9510  <.0001  0.3430  0.3991  *** 
## factor(Cell)I4-I12     0.2943  0.0150  19.6637  <.0001  0.2649  0.3236  *** 
## factor(Cell)I4-I17     0.3320  0.0154  21.5583  <.0001  0.3018  0.3621  *** 
## 
## ---
## Signif. codes:  0 '***' 0.001 '**' 0.01 '*' 0.05 '.' 0.1 ' ' 1
```

```
outztor(tas20.mlmvrem1.sens)
```

```
##                      Estimate         SE   CI95low   CI95upp
## factor(Cell)I11-I12 0.3161871 0.01146943 0.2958126 0.3362741
## factor(Cell)I11-I17 0.3473071 0.01148852 0.3273525 0.3669520
## factor(Cell)I12-I17 0.2816661 0.01257338 0.2588227 0.3041946
## factor(Cell)I2-I11  0.4504339 0.01146679 0.4323383 0.4681668
## factor(Cell)I2-I12  0.3544747 0.01259599 0.3327019 0.3758699
## factor(Cell)I2-I17  0.3871344 0.01281598 0.3655739 0.4082797
## factor(Cell)I2-I4   0.4301636 0.01951766 0.3984783 0.4608234
## factor(Cell)I4-I11  0.3548928 0.01429637 0.3301604 0.3791383
## factor(Cell)I4-I12  0.2860694 0.01496453 0.2589177 0.3127695
## factor(Cell)I4-I17  0.3202775 0.01539684 0.2929377 0.3470940
```

```
## Cluster-robust standard errors
summary(
  robust(tas20.mlmvrem1.sens,
         cluster = StudyID,
         clubSandwich = TRUE)
)
```

```
## 
## Multivariate Meta-Analysis Model (k = 880; method: REML)
## 
##     logLik    Deviance         AIC         BIC        AICc   
##   794.4902  -1588.9804  -1528.9804  -1385.9257  -1526.7635   
## 
## Variance Components:
## 
## outer factor: ESID         (nlvls = 880)
## inner factor: factor(Cell) (nlvls = 10)
## 
##              estim    sqrt  k.lvl  fixed    level 
## tau^2.1     0.0005  0.0231     88     no  I11-I12 
## tau^2.2     0.0005  0.0219     88     no  I11-I17 
## tau^2.3     0.0008  0.0282     88     no  I12-I17 
## tau^2.4     0.0004  0.0198     88     no   I2-I11 
## tau^2.5     0.0014  0.0375     88     no   I2-I12 
## tau^2.6     0.0018  0.0422     88     no   I2-I17 
## tau^2.7     0.0084  0.0915     88     no    I2-I4 
## tau^2.8     0.0022  0.0469     88     no   I4-I11 
## tau^2.9     0.0022  0.0471     88     no   I4-I12 
## tau^2.10    0.0029  0.0541     88     no   I4-I17 
## rho         0.5000                   yes          
## 
## outer factor: StudyID      (nlvls = 62)
## inner factor: factor(Cell) (nlvls = 10)
## 
##                estim    sqrt  k.lvl  fixed    level 
## gamma^2.1     0.0056  0.0747     88     no  I11-I12 
## gamma^2.2     0.0056  0.0751     88     no  I11-I17 
## gamma^2.3     0.0070  0.0834     88     no  I12-I17 
## gamma^2.4     0.0057  0.0754     88     no   I2-I11 
## gamma^2.5     0.0065  0.0808     88     no   I2-I12 
## gamma^2.6     0.0066  0.0811     88     no   I2-I17 
## gamma^2.7     0.0148  0.1215     88     no    I2-I4 
## gamma^2.8     0.0087  0.0931     88     no   I4-I11 
## gamma^2.9     0.0098  0.0992     88     no   I4-I12 
## gamma^2.10    0.0101  0.1005     88     no   I4-I17 
## phi           0.5000                   yes          
## 
## Test for Residual Heterogeneity:
## QE(df = 870) = 6548.1258, p-val < .0001
## 
## Number of estimates:   880
## Number of clusters:    62
## Estimates per cluster: 10-40 (mean: 14.19, median: 10)
## 
## Test of Moderators (coefficients 1:10):¹
## F(df1 = 10, df2 = 49.14) = 120.4870, p-val < .0001
## 
## Model Results:
## 
##                      estimate      se¹     tval¹     df¹    pval¹   ci.lb¹ 
## factor(Cell)I11-I12    0.3274  0.0150   21.7889   58.26   <.0001   0.2973  
## factor(Cell)I11-I17    0.3624  0.0150   24.2139    58.3   <.0001   0.3324  
## factor(Cell)I12-I17    0.2895  0.0164   17.6041   58.92   <.0001   0.2566  
## factor(Cell)I2-I11     0.4852  0.0147   32.9347   58.31   <.0001   0.4558  
## factor(Cell)I2-I12     0.3706  0.0163   22.7179   58.72   <.0001   0.3379  
## factor(Cell)I2-I17     0.4084  0.0164   24.9544    58.7   <.0001   0.3757  
## factor(Cell)I2-I4      0.4601  0.0239   19.2437    59.6   <.0001   0.4123  
## factor(Cell)I4-I11     0.3710  0.0179   20.6927   59.35   <.0001   0.3352  
## factor(Cell)I4-I12     0.2943  0.0191   15.3977    59.6   <.0001   0.2560  
## factor(Cell)I4-I17     0.3320  0.0196   16.9643   59.58   <.0001   0.2928  
##                       ci.ub¹      
## factor(Cell)I11-I12  0.3575   *** 
## factor(Cell)I11-I17  0.3923   *** 
## factor(Cell)I12-I17  0.3224   *** 
## factor(Cell)I2-I11   0.5147   *** 
## factor(Cell)I2-I12   0.4032   *** 
## factor(Cell)I2-I17   0.4412   *** 
## factor(Cell)I2-I4    0.5079   *** 
## factor(Cell)I4-I11   0.4069   *** 
## factor(Cell)I4-I12   0.3325   *** 
## factor(Cell)I4-I17   0.3711   *** 
## 
## ---
## Signif. codes:  0 '***' 0.001 '**' 0.01 '*' 0.05 '.' 0.1 ' ' 1
## 
## 1) results based on cluster-robust inference (var-cov estimator: CR2,
##    approx t/F-tests and confidence intervals, df: Satterthwaite approx)
```

```
## Sensitivity analyses
## Rho = 0, Phi is estimated
## Model specification
tas20.mlmvrem1.sense <- rma.mv(z,
                              V, 
                              data = tas20,
                              random = list(~ factor(Cell) | ESID,
                                            ~ factor(Cell) | StudyID),
                              struc = c("HCS", "HCS"),
                              rho = 0,
                              method = "REML",
                              mods = ~ factor(Cell) - 1,
                              time = TRUE,
                              sparse = TRUE,
                              control = list(optimizer = "optimParallel",
                                             ncpus = ncores))
```

```
## 
## Processing time: 0 hours, 0 minutes, 26.34 seconds
```

```
## Model summary
summary(tas20.mlmvrem1.sense)
```

```
## 
## Multivariate Meta-Analysis Model (k = 880; method: REML)
## 
##     logLik    Deviance         AIC         BIC        AICc   
##   829.8610  -1659.7220  -1597.7220  -1449.8987  -1595.3545   
## 
## Variance Components:
## 
## outer factor: ESID         (nlvls = 880)
## inner factor: factor(Cell) (nlvls = 10)
## 
##              estim    sqrt  k.lvl  fixed    level 
## tau^2.1     0.0013  0.0354     88     no  I11-I12 
## tau^2.2     0.0014  0.0375     88     no  I11-I17 
## tau^2.3     0.0011  0.0334     88     no  I12-I17 
## tau^2.4     0.0018  0.0420     88     no   I2-I11 
## tau^2.5     0.0017  0.0415     88     no   I2-I12 
## tau^2.6     0.0025  0.0504     88     no   I2-I17 
## tau^2.7     0.0115  0.1072     88     no    I2-I4 
## tau^2.8     0.0035  0.0589     88     no   I4-I11 
## tau^2.9     0.0038  0.0615     88     no   I4-I12 
## tau^2.10    0.0039  0.0624     88     no   I4-I17 
## rho         0.0000                   yes          
## 
## outer factor: StudyID      (nlvls = 62)
## inner factor: factor(Cell) (nlvls = 10)
## 
##                estim    sqrt  k.lvl  fixed    level 
## gamma^2.1     0.0106  0.1029     88     no  I11-I12 
## gamma^2.2     0.0099  0.0996     88     no  I11-I17 
## gamma^2.3     0.0139  0.1177     88     no  I12-I17 
## gamma^2.4     0.0093  0.0966     88     no   I2-I11 
## gamma^2.5     0.0131  0.1146     88     no   I2-I12 
## gamma^2.6     0.0121  0.1102     88     no   I2-I17 
## gamma^2.7     0.0225  0.1499     88     no    I2-I4 
## gamma^2.8     0.0150  0.1226     88     no   I4-I11 
## gamma^2.9     0.0166  0.1289     88     no   I4-I12 
## gamma^2.10    0.0186  0.1365     88     no   I4-I17 
## phi           0.9141                    no          
## 
## Test for Residual Heterogeneity:
## QE(df = 870) = 6548.1258, p-val < .0001
## 
## Test of Moderators (coefficients 1:10):
## QM(df = 10) = 1539.2512, p-val < .0001
## 
## Model Results:
## 
##                      estimate      se     zval    pval   ci.lb   ci.ub      
## factor(Cell)I11-I12    0.3309  0.0148  22.3446  <.0001  0.3019  0.3600  *** 
## factor(Cell)I11-I17    0.3649  0.0145  25.1792  <.0001  0.3365  0.3933  *** 
## factor(Cell)I12-I17    0.2926  0.0165  17.7529  <.0001  0.2603  0.3249  *** 
## factor(Cell)I2-I11     0.4869  0.0143  34.0242  <.0001  0.4589  0.5150  *** 
## factor(Cell)I2-I12     0.3754  0.0164  22.9494  <.0001  0.3433  0.4074  *** 
## factor(Cell)I2-I17     0.4126  0.0162  25.5316  <.0001  0.3809  0.4443  *** 
## factor(Cell)I2-I4      0.4638  0.0232  20.0186  <.0001  0.4184  0.5092  *** 
## factor(Cell)I4-I11     0.3761  0.0179  21.0242  <.0001  0.3410  0.4112  *** 
## factor(Cell)I4-I12     0.3006  0.0187  16.0667  <.0001  0.2640  0.3373  *** 
## factor(Cell)I4-I17     0.3378  0.0196  17.2227  <.0001  0.2993  0.3762  *** 
## 
## ---
## Signif. codes:  0 '***' 0.001 '**' 0.01 '*' 0.05 '.' 0.1 ' ' 1
```

```
outztor(tas20.mlmvrem1.sense)
```

```
##                      Estimate         SE   CI95low   CI95upp
## factor(Cell)I11-I12 0.3193682 0.01480978 0.2930637 0.3451895
## factor(Cell)I11-I17 0.3495217 0.01449109 0.3243445 0.3742040
## factor(Cell)I12-I17 0.2845641 0.01648260 0.2546070 0.3139754
## factor(Cell)I2-I11  0.4517761 0.01431029 0.4291710 0.4738157
## factor(Cell)I2-I12  0.3586911 0.01635555 0.3304415 0.3862986
## factor(Cell)I2-I17  0.3906878 0.01615941 0.3635208 0.4171908
## factor(Cell)I2-I4   0.4331447 0.02316235 0.3955443 0.4692956
## factor(Cell)I4-I11  0.3593231 0.01788739 0.3284109 0.3894663
## factor(Cell)I4-I12  0.2918907 0.01870926 0.2579939 0.3250698
## factor(Cell)I4-I17  0.3254932 0.01960989 0.2907080 0.3594191
```

```
## Cluster-robust standard errors
summary(
  robust(tas20.mlmvrem1.sense,
         cluster = StudyID,
         clubSandwich = TRUE)
)
```

```
## 
## Multivariate Meta-Analysis Model (k = 880; method: REML)
## 
##     logLik    Deviance         AIC         BIC        AICc   
##   829.8610  -1659.7220  -1597.7220  -1449.8987  -1595.3545   
## 
## Variance Components:
## 
## outer factor: ESID         (nlvls = 880)
## inner factor: factor(Cell) (nlvls = 10)
## 
##              estim    sqrt  k.lvl  fixed    level 
## tau^2.1     0.0013  0.0354     88     no  I11-I12 
## tau^2.2     0.0014  0.0375     88     no  I11-I17 
## tau^2.3     0.0011  0.0334     88     no  I12-I17 
## tau^2.4     0.0018  0.0420     88     no   I2-I11 
## tau^2.5     0.0017  0.0415     88     no   I2-I12 
## tau^2.6     0.0025  0.0504     88     no   I2-I17 
## tau^2.7     0.0115  0.1072     88     no    I2-I4 
## tau^2.8     0.0035  0.0589     88     no   I4-I11 
## tau^2.9     0.0038  0.0615     88     no   I4-I12 
## tau^2.10    0.0039  0.0624     88     no   I4-I17 
## rho         0.0000                   yes          
## 
## outer factor: StudyID      (nlvls = 62)
## inner factor: factor(Cell) (nlvls = 10)
## 
##                estim    sqrt  k.lvl  fixed    level 
## gamma^2.1     0.0106  0.1029     88     no  I11-I12 
## gamma^2.2     0.0099  0.0996     88     no  I11-I17 
## gamma^2.3     0.0139  0.1177     88     no  I12-I17 
## gamma^2.4     0.0093  0.0966     88     no   I2-I11 
## gamma^2.5     0.0131  0.1146     88     no   I2-I12 
## gamma^2.6     0.0121  0.1102     88     no   I2-I17 
## gamma^2.7     0.0225  0.1499     88     no    I2-I4 
## gamma^2.8     0.0150  0.1226     88     no   I4-I11 
## gamma^2.9     0.0166  0.1289     88     no   I4-I12 
## gamma^2.10    0.0186  0.1365     88     no   I4-I17 
## phi           0.9141                    no          
## 
## Test for Residual Heterogeneity:
## QE(df = 870) = 6548.1258, p-val < .0001
## 
## Number of estimates:   880
## Number of clusters:    62
## Estimates per cluster: 10-40 (mean: 14.19, median: 10)
## 
## Test of Moderators (coefficients 1:10):¹
## F(df1 = 10, df2 = 45.3) = 113.4940, p-val < .0001
## 
## Model Results:
## 
##                      estimate      se¹     tval¹     df¹    pval¹   ci.lb¹ 
## factor(Cell)I11-I12    0.3309  0.0157   21.1407   59.85   <.0001   0.2996  
## factor(Cell)I11-I17    0.3649  0.0151   24.1531   59.72   <.0001   0.3347  
## factor(Cell)I12-I17    0.2926  0.0166   17.6181   60.24   <.0001   0.2594  
## factor(Cell)I2-I11     0.4869  0.0153   31.9085   59.55   <.0001   0.4564  
## factor(Cell)I2-I12     0.3754  0.0167   22.5320   60.12   <.0001   0.3421  
## factor(Cell)I2-I17     0.4126  0.0166   24.8749    59.9   <.0001   0.3794  
## factor(Cell)I2-I4      0.4638  0.0235   19.7222   59.84   <.0001   0.4167  
## factor(Cell)I4-I11     0.3761  0.0178   21.1489   60.11   <.0001   0.3405  
## factor(Cell)I4-I12     0.3006  0.0190   15.8490   60.19   <.0001   0.2627  
## factor(Cell)I4-I17     0.3378  0.0200   16.8947   60.32   <.0001   0.2978  
##                       ci.ub¹      
## factor(Cell)I11-I12  0.3623   *** 
## factor(Cell)I11-I17  0.3951   *** 
## factor(Cell)I12-I17  0.3259   *** 
## factor(Cell)I2-I11   0.5175   *** 
## factor(Cell)I2-I12   0.4087   *** 
## factor(Cell)I2-I17   0.4458   *** 
## factor(Cell)I2-I4    0.5108   *** 
## factor(Cell)I4-I11   0.4117   *** 
## factor(Cell)I4-I12   0.3386   *** 
## factor(Cell)I4-I17   0.3778   *** 
## 
## ---
## Signif. codes:  0 '***' 0.001 '**' 0.01 '*' 0.05 '.' 0.1 ' ' 1
## 
## 1) results based on cluster-robust inference (var-cov estimator: CR2,
##    approx t/F-tests and confidence intervals, df: Satterthwaite approx)
```

### Models with some level-specific and some constrained variance estimates (`Model 2` and `Model 3`)

Model `mlmvrem2` assumes that each type of pooled
correlation has its own set of estimates of the amounts of residual
heterogeneity between effect sizes within studies (aka
`Model 2`).

#### Correlation-specific effect sizes and within-study heterogeneity, overall between-study heterogeneity (`Model 2`)

```
## Model specification
tas20.mlmvrem2 <- rma.mv(z,
                         V, 
                         data = tas20,
                         random = list(~ factor(Cell) | ESID,
                                       ~ factor(Cell) | StudyID),
                         struc = c("HCS", "CS"),
                         rho = 0,
                         phi = 0,
                         method = "REML",
                         mods = ~ factor(Cell) - 1,
                         time = TRUE,
                         sparse = TRUE,
                         control = list(optimizer = "optimParallel",
                                        ncpus = ncores))
```

```
## 
## Processing time: 0 hours, 0 minutes, 8.08 seconds
```

```
## Model summary
summary(tas20.mlmvrem2)
```

```
## 
## Multivariate Meta-Analysis Model (k = 880; method: REML)
## 
##     logLik    Deviance         AIC         BIC        AICc   
##   617.7094  -1235.4188  -1193.4188  -1093.2805  -1192.3292   
## 
## Variance Components:
## 
## outer factor: ESID         (nlvls = 880)
## inner factor: factor(Cell) (nlvls = 10)
## 
##              estim    sqrt  k.lvl  fixed    level 
## tau^2.1     0.0010  0.0310     88     no  I11-I12 
## tau^2.2     0.0008  0.0282     88     no  I11-I17 
## tau^2.3     0.0018  0.0422     88     no  I12-I17 
## tau^2.4     0.0006  0.0254     88     no   I2-I11 
## tau^2.5     0.0025  0.0504     88     no   I2-I12 
## tau^2.6     0.0031  0.0556     88     no   I2-I17 
## tau^2.7     0.0187  0.1368     88     no    I2-I4 
## tau^2.8     0.0051  0.0713     88     no   I4-I11 
## tau^2.9     0.0053  0.0731     88     no   I4-I12 
## tau^2.10    0.0072  0.0850     88     no   I4-I17 
## rho         0.0000                   yes          
## 
## outer factor: StudyID      (nlvls = 62)
## inner factor: factor(Cell) (nlvls = 10)
## 
##             estim    sqrt  fixed 
## gamma^2    0.0064  0.0802     no 
## phi        0.0000            yes 
## 
## Test for Residual Heterogeneity:
## QE(df = 870) = 6548.1258, p-val < .0001
## 
## Test of Moderators (coefficients 1:10):
## QM(df = 10) = 5497.8940, p-val < .0001
## 
## Model Results:
## 
##                      estimate      se     zval    pval   ci.lb   ci.ub      
## factor(Cell)I11-I12    0.3199  0.0123  26.0155  <.0001  0.2958  0.3440  *** 
## factor(Cell)I11-I17    0.3561  0.0122  29.1578  <.0001  0.3322  0.3800  *** 
## factor(Cell)I12-I17    0.2831  0.0127  22.2507  <.0001  0.2581  0.3080  *** 
## factor(Cell)I2-I11     0.4783  0.0121  39.4082  <.0001  0.4545  0.5021  *** 
## factor(Cell)I2-I12     0.3624  0.0131  27.7036  <.0001  0.3368  0.3881  *** 
## factor(Cell)I2-I17     0.4018  0.0133  30.1057  <.0001  0.3756  0.4280  *** 
## factor(Cell)I2-I4      0.4527  0.0191  23.7214  <.0001  0.4153  0.4901  *** 
## factor(Cell)I4-I11     0.3617  0.0142  25.4160  <.0001  0.3338  0.3896  *** 
## factor(Cell)I4-I12     0.2848  0.0143  19.8630  <.0001  0.2567  0.3129  *** 
## factor(Cell)I4-I17     0.3230  0.0151  21.3793  <.0001  0.2934  0.3527  *** 
## 
## ---
## Signif. codes:  0 '***' 0.001 '**' 0.01 '*' 0.05 '.' 0.1 ' ' 1
```

```
outztor(tas20.mlmvrem2)
```

```
##                      Estimate         SE   CI95low   CI95upp
## factor(Cell)I11-I12 0.3094341 0.01229665 0.2874802 0.3310630
## factor(Cell)I11-I17 0.3417800 0.01221249 0.3204687 0.3627455
## factor(Cell)I12-I17 0.2757324 0.01272060 0.2525402 0.2986081
## factor(Cell)I2-I11  0.4448816 0.01213648 0.4256011 0.4637583
## factor(Cell)I2-I12  0.3473408 0.01308117 0.3245965 0.3696836
## factor(Cell)I2-I17  0.3814857 0.01334542 0.3589143 0.4036111
## factor(Cell)I2-I4   0.4240760 0.01907968 0.3929225 0.4542573
## factor(Cell)I4-I11  0.3467004 0.01422976 0.3219282 0.3709983
## factor(Cell)I4-I12  0.2773691 0.01433871 0.2512291 0.3031047
## factor(Cell)I4-I17  0.3122577 0.01510905 0.2852883 0.3387329
```

```
## Cluster-robust standard errors
tas20.mlmvrem2.robust <- robust(tas20.mlmvrem2, 
                                cluster = StudyID, 
                                clubSandwich = TRUE)

summary(tas20.mlmvrem2.robust)
```

```
## 
## Multivariate Meta-Analysis Model (k = 880; method: REML)
## 
##     logLik    Deviance         AIC         BIC        AICc   
##   617.7094  -1235.4188  -1193.4188  -1093.2805  -1192.3292   
## 
## Variance Components:
## 
## outer factor: ESID         (nlvls = 880)
## inner factor: factor(Cell) (nlvls = 10)
## 
##              estim    sqrt  k.lvl  fixed    level 
## tau^2.1     0.0010  0.0310     88     no  I11-I12 
## tau^2.2     0.0008  0.0282     88     no  I11-I17 
## tau^2.3     0.0018  0.0422     88     no  I12-I17 
## tau^2.4     0.0006  0.0254     88     no   I2-I11 
## tau^2.5     0.0025  0.0504     88     no   I2-I12 
## tau^2.6     0.0031  0.0556     88     no   I2-I17 
## tau^2.7     0.0187  0.1368     88     no    I2-I4 
## tau^2.8     0.0051  0.0713     88     no   I4-I11 
## tau^2.9     0.0053  0.0731     88     no   I4-I12 
## tau^2.10    0.0072  0.0850     88     no   I4-I17 
## rho         0.0000                   yes          
## 
## outer factor: StudyID      (nlvls = 62)
## inner factor: factor(Cell) (nlvls = 10)
## 
##             estim    sqrt  fixed 
## gamma^2    0.0064  0.0802     no 
## phi        0.0000            yes 
## 
## Test for Residual Heterogeneity:
## QE(df = 870) = 6548.1258, p-val < .0001
## 
## Number of estimates:   880
## Number of clusters:    62
## Estimates per cluster: 10-40 (mean: 14.19, median: 10)
## 
## Test of Moderators (coefficients 1:10):¹
## F(df1 = 10, df2 = 49.63) = 129.9259, p-val < .0001
## 
## Model Results:
## 
##                      estimate      se¹     tval¹     df¹    pval¹   ci.lb¹ 
## factor(Cell)I11-I12    0.3199  0.0145   22.1194   58.81   <.0001   0.2910  
## factor(Cell)I11-I17    0.3561  0.0145   24.5607    58.8   <.0001   0.3271  
## factor(Cell)I12-I17    0.2831  0.0160   17.6597   58.73   <.0001   0.2510  
## factor(Cell)I2-I11     0.4783  0.0143   33.5554   58.79   <.0001   0.4498  
## factor(Cell)I2-I12     0.3624  0.0161   22.5689   58.69   <.0001   0.3303  
## factor(Cell)I2-I17     0.4018  0.0162   24.7400   58.61   <.0001   0.3693  
## factor(Cell)I2-I4      0.4527  0.0225   20.1041   56.18   <.0001   0.4076  
## factor(Cell)I4-I11     0.3617  0.0173   20.9640    58.3   <.0001   0.3272  
## factor(Cell)I4-I12     0.2848  0.0186   15.3148   58.28   <.0001   0.2476  
## factor(Cell)I4-I17     0.3230  0.0191   16.9136   57.95   <.0001   0.2848  
##                       ci.ub¹      
## factor(Cell)I11-I12  0.3489   *** 
## factor(Cell)I11-I17  0.3851   *** 
## factor(Cell)I12-I17  0.3151   *** 
## factor(Cell)I2-I11   0.5068   *** 
## factor(Cell)I2-I12   0.3946   *** 
## factor(Cell)I2-I17   0.4343   *** 
## factor(Cell)I2-I4    0.4978   *** 
## factor(Cell)I4-I11   0.3962   *** 
## factor(Cell)I4-I12   0.3221   *** 
## factor(Cell)I4-I17   0.3613   *** 
## 
## ---
## Signif. codes:  0 '***' 0.001 '**' 0.01 '*' 0.05 '.' 0.1 ' ' 1
## 
## 1) results based on cluster-robust inference (var-cov estimator: CR2,
##    approx t/F-tests and confidence intervals, df: Satterthwaite approx)
```

```
## Sensitivity analysis
## Random effects: rho = 0.5, phi = 0.5
## Model specification
tas20.mlmvrem2.sens <- rma.mv(z,
                              V, 
                              data = tas20,
                              random = list(~ factor(Cell) | ESID,
                                            ~ factor(Cell) | StudyID),
                              struc = c("HCS", "CS"),
                              rho = 0.5,
                              phi = 0.5,
                              method = "REML",
                              mods = ~ factor(Cell) - 1,
                              time = TRUE,
                              sparse = TRUE,
                              control = list(optimizer = "optimParallel",
                                             ncpus = ncores))
```

```
## 
## Processing time: 0 hours, 0 minutes, 25.93 seconds
```

```
## Model summary
summary(tas20.mlmvrem2.sens)
```

```
## 
## Multivariate Meta-Analysis Model (k = 880; method: REML)
## 
##     logLik    Deviance         AIC         BIC        AICc   
##   786.0989  -1572.1977  -1530.1977  -1430.0594  -1529.1081   
## 
## Variance Components:
## 
## outer factor: ESID         (nlvls = 880)
## inner factor: factor(Cell) (nlvls = 10)
## 
##              estim    sqrt  k.lvl  fixed    level 
## tau^2.1     0.0005  0.0224     88     no  I11-I12 
## tau^2.2     0.0004  0.0204     88     no  I11-I17 
## tau^2.3     0.0008  0.0284     88     no  I12-I17 
## tau^2.4     0.0004  0.0190     88     no   I2-I11 
## tau^2.5     0.0014  0.0376     88     no   I2-I12 
## tau^2.6     0.0018  0.0422     88     no   I2-I17 
## tau^2.7     0.0122  0.1104     88     no    I2-I4 
## tau^2.8     0.0026  0.0510     88     no   I4-I11 
## tau^2.9     0.0030  0.0545     88     no   I4-I12 
## tau^2.10    0.0036  0.0598     88     no   I4-I17 
## rho         0.5000                   yes          
## 
## outer factor: StudyID      (nlvls = 62)
## inner factor: factor(Cell) (nlvls = 10)
## 
##             estim    sqrt  fixed 
## gamma^2    0.0071  0.0845     no 
## phi        0.5000            yes 
## 
## Test for Residual Heterogeneity:
## QE(df = 870) = 6548.1258, p-val < .0001
## 
## Test of Moderators (coefficients 1:10):
## QM(df = 10) = 2053.3234, p-val < .0001
## 
## Model Results:
## 
##                      estimate      se     zval    pval   ci.lb   ci.ub      
## factor(Cell)I11-I12    0.3274  0.0126  26.0780  <.0001  0.3028  0.3520  *** 
## factor(Cell)I11-I17    0.3626  0.0125  28.9838  <.0001  0.3381  0.3871  *** 
## factor(Cell)I12-I17    0.2893  0.0127  22.7618  <.0001  0.2644  0.3142  *** 
## factor(Cell)I2-I11     0.4851  0.0125  38.8747  <.0001  0.4607  0.5096  *** 
## factor(Cell)I2-I12     0.3701  0.0130  28.4596  <.0001  0.3446  0.3956  *** 
## factor(Cell)I2-I17     0.4080  0.0132  30.9506  <.0001  0.3822  0.4339  *** 
## factor(Cell)I2-I4      0.4596  0.0173  26.6273  <.0001  0.4258  0.4935  *** 
## factor(Cell)I4-I11     0.3710  0.0136  27.3678  <.0001  0.3445  0.3976  *** 
## factor(Cell)I4-I12     0.2942  0.0137  21.4293  <.0001  0.2673  0.3211  *** 
## factor(Cell)I4-I17     0.3319  0.0140  23.7195  <.0001  0.3045  0.3594  *** 
## 
## ---
## Signif. codes:  0 '***' 0.001 '**' 0.01 '*' 0.05 '.' 0.1 ' ' 1
```

```
outztor(tas20.mlmvrem2.sens)
```

```
##                      Estimate         SE   CI95low   CI95upp
## factor(Cell)I11-I12 0.3161704 0.01255346 0.2938554 0.3381410
## factor(Cell)I11-I17 0.3474881 0.01250924 0.3257489 0.3688601
## factor(Cell)I12-I17 0.2815026 0.01270977 0.2584075 0.3042761
## factor(Cell)I2-I11  0.4503539 0.01247905 0.4306418 0.4696365
## factor(Cell)I2-I12  0.3540631 0.01300302 0.3315732 0.3761508
## factor(Cell)I2-I17  0.3867972 0.01318245 0.3646075 0.4085478
## factor(Cell)I2-I4   0.4297777 0.01725964 0.4017989 0.4569548
## factor(Cell)I4-I11  0.3549067 0.01355697 0.3314654 0.3779101
## factor(Cell)I4-I12  0.2859736 0.01372684 0.2610827 0.3104845
## factor(Cell)I4-I17  0.3202566 0.01399320 0.2954300 0.3446511
```

```
## Cluster-robust standard errors
summary(
  robust(tas20.mlmvrem2.sens,
         cluster = StudyID,
         clubSandwich = TRUE)
)
```

```
## 
## Multivariate Meta-Analysis Model (k = 880; method: REML)
## 
##     logLik    Deviance         AIC         BIC        AICc   
##   786.0989  -1572.1977  -1530.1977  -1430.0594  -1529.1081   
## 
## Variance Components:
## 
## outer factor: ESID         (nlvls = 880)
## inner factor: factor(Cell) (nlvls = 10)
## 
##              estim    sqrt  k.lvl  fixed    level 
## tau^2.1     0.0005  0.0224     88     no  I11-I12 
## tau^2.2     0.0004  0.0204     88     no  I11-I17 
## tau^2.3     0.0008  0.0284     88     no  I12-I17 
## tau^2.4     0.0004  0.0190     88     no   I2-I11 
## tau^2.5     0.0014  0.0376     88     no   I2-I12 
## tau^2.6     0.0018  0.0422     88     no   I2-I17 
## tau^2.7     0.0122  0.1104     88     no    I2-I4 
## tau^2.8     0.0026  0.0510     88     no   I4-I11 
## tau^2.9     0.0030  0.0545     88     no   I4-I12 
## tau^2.10    0.0036  0.0598     88     no   I4-I17 
## rho         0.5000                   yes          
## 
## outer factor: StudyID      (nlvls = 62)
## inner factor: factor(Cell) (nlvls = 10)
## 
##             estim    sqrt  fixed 
## gamma^2    0.0071  0.0845     no 
## phi        0.5000            yes 
## 
## Test for Residual Heterogeneity:
## QE(df = 870) = 6548.1258, p-val < .0001
## 
## Number of estimates:   880
## Number of clusters:    62
## Estimates per cluster: 10-40 (mean: 14.19, median: 10)
## 
## Test of Moderators (coefficients 1:10):¹
## F(df1 = 10, df2 = 49) = 122.5012, p-val < .0001
## 
## Model Results:
## 
##                      estimate      se¹     tval¹     df¹    pval¹   ci.lb¹ 
## factor(Cell)I11-I12    0.3274  0.0150   21.8849   58.98   <.0001   0.2975  
## factor(Cell)I11-I17    0.3626  0.0150   24.1024   58.98   <.0001   0.3325  
## factor(Cell)I12-I17    0.2893  0.0165   17.5615   58.98   <.0001   0.2563  
## factor(Cell)I2-I11     0.4851  0.0148   32.8551   58.99   <.0001   0.4556  
## factor(Cell)I2-I12     0.3701  0.0163   22.6908   58.96   <.0001   0.3374  
## factor(Cell)I2-I17     0.4080  0.0164   24.8330   58.92   <.0001   0.3751  
## factor(Cell)I2-I4      0.4596  0.0232   19.8522   57.18   <.0001   0.4133  
## factor(Cell)I4-I11     0.3710  0.0178   20.8885   58.83   <.0001   0.3355  
## factor(Cell)I4-I12     0.2942  0.0190   15.5019   58.78   <.0001   0.2562  
## factor(Cell)I4-I17     0.3319  0.0195   16.9893    58.7   <.0001   0.2928  
##                       ci.ub¹      
## factor(Cell)I11-I12  0.3573   *** 
## factor(Cell)I11-I17  0.3927   *** 
## factor(Cell)I12-I17  0.3223   *** 
## factor(Cell)I2-I11   0.5147   *** 
## factor(Cell)I2-I12   0.4027   *** 
## factor(Cell)I2-I17   0.4409   *** 
## factor(Cell)I2-I4    0.5060   *** 
## factor(Cell)I4-I11   0.4066   *** 
## factor(Cell)I4-I12   0.3322   *** 
## factor(Cell)I4-I17   0.3710   *** 
## 
## ---
## Signif. codes:  0 '***' 0.001 '**' 0.01 '*' 0.05 '.' 0.1 ' ' 1
## 
## 1) results based on cluster-robust inference (var-cov estimator: CR2,
##    approx t/F-tests and confidence intervals, df: Satterthwaite approx)
```

```
## Sensitivity analysis
## Random effects: rho = 1, phi = 1
## Model specification
tas20.mlmvrem2.sent <- rma.mv(z,
                              V, 
                              data = tas20,
                              random = list(~ factor(Cell) | ESID,
                                            ~ factor(Cell) | StudyID),
                              struc = c("HCS", "CS"),
                              rho = 1,
                              phi = 1,
                              method = "REML",
                              mods = ~ factor(Cell) - 1,
                              time = TRUE,
                              sparse = TRUE,
                              control = list(optimizer = "optimParallel",
                                             ncpus = ncores))
```

```
## 
## Processing time: 0 hours, 0 minutes, 13.47 seconds
```

```
## Model summary
summary(tas20.mlmvrem2.sent)
```

```
## 
## Multivariate Meta-Analysis Model (k = 880; method: REML)
## 
##     logLik    Deviance         AIC         BIC        AICc   
##   817.8745  -1635.7489  -1593.7489  -1493.6106  -1592.6593   
## 
## Variance Components:
## 
## outer factor: ESID         (nlvls = 880)
## inner factor: factor(Cell) (nlvls = 10)
## 
##              estim    sqrt  k.lvl  fixed    level 
## tau^2.1     0.0020  0.0447     88     no  I11-I12 
## tau^2.2     0.0020  0.0449     88     no  I11-I17 
## tau^2.3     0.0018  0.0422     88     no  I12-I17 
## tau^2.4     0.0029  0.0536     88     no   I2-I11 
## tau^2.5     0.0023  0.0480     88     no   I2-I12 
## tau^2.6     0.0036  0.0600     88     no   I2-I17 
## tau^2.7     0.0159  0.1260     88     no    I2-I4 
## tau^2.8     0.0055  0.0744     88     no   I4-I11 
## tau^2.9     0.0061  0.0782     88     no   I4-I12 
## tau^2.10    0.0070  0.0834     88     no   I4-I17 
## rho         1.0000                   yes          
## 
## outer factor: StudyID      (nlvls = 62)
## inner factor: factor(Cell) (nlvls = 10)
## 
##             estim    sqrt  fixed 
## gamma^2    0.0118  0.1086     no 
## phi        1.0000            yes 
## 
## Test for Residual Heterogeneity:
## QE(df = 870) = 6548.1258, p-val < .0001
## 
## Test of Moderators (coefficients 1:10):
## QM(df = 10) = 1166.1294, p-val < .0001
## 
## Model Results:
## 
##                      estimate      se     zval    pval   ci.lb   ci.ub      
## factor(Cell)I11-I12    0.3310  0.0157  21.0774  <.0001  0.3003  0.3618  *** 
## factor(Cell)I11-I17    0.3646  0.0157  23.2118  <.0001  0.3338  0.3953  *** 
## factor(Cell)I12-I17    0.2924  0.0156  18.7295  <.0001  0.2618  0.3230  *** 
## factor(Cell)I2-I11     0.4856  0.0160  30.2779  <.0001  0.4542  0.5171  *** 
## factor(Cell)I2-I12     0.3758  0.0158  23.7557  <.0001  0.3448  0.4068  *** 
## factor(Cell)I2-I17     0.4133  0.0163  25.3317  <.0001  0.3813  0.4453  *** 
## factor(Cell)I2-I4      0.4639  0.0202  22.9557  <.0001  0.4243  0.5035  *** 
## factor(Cell)I4-I11     0.3775  0.0170  22.2120  <.0001  0.3442  0.4108  *** 
## factor(Cell)I4-I12     0.3018  0.0172  17.5478  <.0001  0.2681  0.3355  *** 
## factor(Cell)I4-I17     0.3395  0.0175  19.4191  <.0001  0.3052  0.3737  *** 
## 
## ---
## Signif. codes:  0 '***' 0.001 '**' 0.01 '*' 0.05 '.' 0.1 ' ' 1
```

```
outztor(tas20.mlmvrem2.sent)
```

```
##                      Estimate         SE   CI95low   CI95upp
## factor(Cell)I11-I12 0.3194548 0.01570460 0.2915477 0.3468186
## factor(Cell)I11-I17 0.3492233 0.01570445 0.3219098 0.3759560
## factor(Cell)I12-I17 0.2843047 0.01560829 0.2559456 0.3121748
## factor(Cell)I2-I11  0.4507545 0.01603824 0.4253532 0.4754462
## factor(Cell)I2-I12  0.3590828 0.01581940 0.3317776 0.3857867
## factor(Cell)I2-I17  0.3912678 0.01631395 0.3638520 0.4180062
## factor(Cell)I2-I4   0.4332531 0.02020552 0.4005359 0.4648669
## factor(Cell)I4-I11  0.3605184 0.01699285 0.3311978 0.3891434
## factor(Cell)I4-I12  0.2929229 0.01719475 0.2618152 0.3234227
## factor(Cell)I4-I17  0.3270074 0.01747961 0.2960738 0.3572556
```

```
## Cluster-robust standard errors
summary(
  robust(tas20.mlmvrem2.sent,
         cluster = StudyID,
         clubSandwich = TRUE)
)
```

```
## 
## Multivariate Meta-Analysis Model (k = 880; method: REML)
## 
##     logLik    Deviance         AIC         BIC        AICc   
##   817.8745  -1635.7489  -1593.7489  -1493.6106  -1592.6593   
## 
## Variance Components:
## 
## outer factor: ESID         (nlvls = 880)
## inner factor: factor(Cell) (nlvls = 10)
## 
##              estim    sqrt  k.lvl  fixed    level 
## tau^2.1     0.0020  0.0447     88     no  I11-I12 
## tau^2.2     0.0020  0.0449     88     no  I11-I17 
## tau^2.3     0.0018  0.0422     88     no  I12-I17 
## tau^2.4     0.0029  0.0536     88     no   I2-I11 
## tau^2.5     0.0023  0.0480     88     no   I2-I12 
## tau^2.6     0.0036  0.0600     88     no   I2-I17 
## tau^2.7     0.0159  0.1260     88     no    I2-I4 
## tau^2.8     0.0055  0.0744     88     no   I4-I11 
## tau^2.9     0.0061  0.0782     88     no   I4-I12 
## tau^2.10    0.0070  0.0834     88     no   I4-I17 
## rho         1.0000                   yes          
## 
## outer factor: StudyID      (nlvls = 62)
## inner factor: factor(Cell) (nlvls = 10)
## 
##             estim    sqrt  fixed 
## gamma^2    0.0118  0.1086     no 
## phi        1.0000            yes 
## 
## Test for Residual Heterogeneity:
## QE(df = 870) = 6548.1258, p-val < .0001
## 
## Number of estimates:   880
## Number of clusters:    62
## Estimates per cluster: 10-40 (mean: 14.19, median: 10)
## 
## Test of Moderators (coefficients 1:10):¹
## F(df1 = 10, df2 = 40.8) = 107.1152, p-val < .0001
## 
## Model Results:
## 
##                      estimate      se¹     tval¹     df¹    pval¹   ci.lb¹ 
## factor(Cell)I11-I12    0.3310  0.0158   20.8904   59.91   <.0001   0.2993  
## factor(Cell)I11-I17    0.3646  0.0151   24.1731   59.91   <.0001   0.3344  
## factor(Cell)I12-I17    0.2924  0.0162   18.0026   59.95   <.0001   0.2599  
## factor(Cell)I2-I11     0.4856  0.0157   31.0253   59.79   <.0001   0.4543  
## factor(Cell)I2-I12     0.3758  0.0166   22.6015   59.87   <.0001   0.3426  
## factor(Cell)I2-I17     0.4133  0.0167   24.7045   59.66   <.0001   0.3798  
## factor(Cell)I2-I4      0.4639  0.0228   20.3204   57.63   <.0001   0.4182  
## factor(Cell)I4-I11     0.3775  0.0176   21.5053   59.33   <.0001   0.3424  
## factor(Cell)I4-I12     0.3018  0.0187   16.1603   59.23   <.0001   0.2644  
## factor(Cell)I4-I17     0.3395  0.0205   16.5279   59.09   <.0001   0.2984  
##                       ci.ub¹      
## factor(Cell)I11-I12  0.3627   *** 
## factor(Cell)I11-I17  0.3947   *** 
## factor(Cell)I12-I17  0.3248   *** 
## factor(Cell)I2-I11   0.5170   *** 
## factor(Cell)I2-I12   0.4091   *** 
## factor(Cell)I2-I17   0.4468   *** 
## factor(Cell)I2-I4    0.5096   *** 
## factor(Cell)I4-I11   0.4126   *** 
## factor(Cell)I4-I12   0.3391   *** 
## factor(Cell)I4-I17   0.3806   *** 
## 
## ---
## Signif. codes:  0 '***' 0.001 '**' 0.01 '*' 0.05 '.' 0.1 ' ' 1
## 
## 1) results based on cluster-robust inference (var-cov estimator: CR2,
##    approx t/F-tests and confidence intervals, df: Satterthwaite approx)
```

```
## Sensitivity analysis
## Random effects: rho = 0, phi estimated
## Model specification
tas20.mlmvrem2.senu <- rma.mv(z,
                              V, 
                              data = tas20,
                              random = list(~ factor(Cell) | ESID,
                                            ~ factor(Cell) | StudyID),
                              struc = c("HCS", "CS"),
                              rho = 0,
                              phi = NA,
                              method = "REML",
                              mods = ~ factor(Cell) - 1,
                              time = TRUE,
                              sparse = TRUE,
                              control = list(optimizer = "optimParallel",
                                             ncpus = ncores))
```

```
## 
## Processing time: 0 hours, 0 minutes, 19.39 seconds
```

```
## Model summary
summary(tas20.mlmvrem2.senu)
```

```
## 
## Multivariate Meta-Analysis Model (k = 880; method: REML)
## 
##     logLik    Deviance         AIC         BIC        AICc   
##   822.3037  -1644.6075  -1600.6075  -1495.7006  -1599.4127   
## 
## Variance Components:
## 
## outer factor: ESID         (nlvls = 880)
## inner factor: factor(Cell) (nlvls = 10)
## 
##              estim    sqrt  k.lvl  fixed    level 
## tau^2.1     0.0009  0.0307     88     no  I11-I12 
## tau^2.2     0.0010  0.0320     88     no  I11-I17 
## tau^2.3     0.0012  0.0343     88     no  I12-I17 
## tau^2.4     0.0013  0.0364     88     no   I2-I11 
## tau^2.5     0.0017  0.0415     88     no   I2-I12 
## tau^2.6     0.0024  0.0493     88     no   I2-I17 
## tau^2.7     0.0142  0.1192     88     no    I2-I4 
## tau^2.8     0.0041  0.0637     88     no   I4-I11 
## tau^2.9     0.0048  0.0692     88     no   I4-I12 
## tau^2.10    0.0051  0.0712     88     no   I4-I17 
## rho         0.0000                   yes          
## 
## outer factor: StudyID      (nlvls = 62)
## inner factor: factor(Cell) (nlvls = 10)
## 
##             estim    sqrt  fixed 
## gamma^2    0.0129  0.1135     no 
## phi        0.9130             no 
## 
## Test for Residual Heterogeneity:
## QE(df = 870) = 6548.1258, p-val < .0001
## 
## Test of Moderators (coefficients 1:10):
## QM(df = 10) = 1131.5372, p-val < .0001
## 
## Model Results:
## 
##                      estimate      se     zval    pval   ci.lb   ci.ub      
## factor(Cell)I11-I12    0.3315  0.0159  20.8032  <.0001  0.3003  0.3627  *** 
## factor(Cell)I11-I17    0.3654  0.0160  22.8868  <.0001  0.3341  0.3967  *** 
## factor(Cell)I12-I17    0.2925  0.0160  18.2504  <.0001  0.2611  0.3239  *** 
## factor(Cell)I2-I11     0.4879  0.0161  30.3396  <.0001  0.4564  0.5195  *** 
## factor(Cell)I2-I12     0.3753  0.0162  23.1157  <.0001  0.3435  0.4071  *** 
## factor(Cell)I2-I17     0.4124  0.0165  24.9831  <.0001  0.3800  0.4447  *** 
## factor(Cell)I2-I4      0.4636  0.0202  22.8988  <.0001  0.4239  0.5032  *** 
## factor(Cell)I4-I11     0.3764  0.0171  22.0380  <.0001  0.3430  0.4099  *** 
## factor(Cell)I4-I12     0.3003  0.0173  17.3216  <.0001  0.2663  0.3342  *** 
## factor(Cell)I4-I17     0.3378  0.0174  19.3796  <.0001  0.3036  0.3719  *** 
## 
## ---
## Signif. codes:  0 '***' 0.001 '**' 0.01 '*' 0.05 '.' 0.1 ' ' 1
```

```
outztor(tas20.mlmvrem2.senu)
```

```
##                      Estimate         SE   CI95low   CI95upp
## factor(Cell)I11-I12 0.3198746 0.01593408 0.2915639 0.3476255
## factor(Cell)I11-I17 0.3499756 0.01596486 0.3222203 0.3771296
## factor(Cell)I12-I17 0.2844004 0.01602363 0.2552818 0.3130035
## factor(Cell)I2-I11  0.4525873 0.01608155 0.4271679 0.4772918
## factor(Cell)I2-I12  0.3586031 0.01623352 0.3305646 0.3860091
## factor(Cell)I2-I17  0.3905015 0.01650534 0.3627412 0.4175694
## factor(Cell)I2-I4   0.4329824 0.02024114 0.4001975 0.4646605
## factor(Cell)I4-I11  0.3596027 0.01707925 0.3301098 0.3883941
## factor(Cell)I4-I12  0.2915429 0.01733224 0.2601579 0.3223126
## factor(Cell)I4-I17  0.3254927 0.01742781 0.2946193 0.3556873
```

```
## Cluster-robust standard errors
summary(
  robust(tas20.mlmvrem2.senu,
         cluster = StudyID,
         clubSandwich = TRUE)
)
```

```
## 
## Multivariate Meta-Analysis Model (k = 880; method: REML)
## 
##     logLik    Deviance         AIC         BIC        AICc   
##   822.3037  -1644.6075  -1600.6075  -1495.7006  -1599.4127   
## 
## Variance Components:
## 
## outer factor: ESID         (nlvls = 880)
## inner factor: factor(Cell) (nlvls = 10)
## 
##              estim    sqrt  k.lvl  fixed    level 
## tau^2.1     0.0009  0.0307     88     no  I11-I12 
## tau^2.2     0.0010  0.0320     88     no  I11-I17 
## tau^2.3     0.0012  0.0343     88     no  I12-I17 
## tau^2.4     0.0013  0.0364     88     no   I2-I11 
## tau^2.5     0.0017  0.0415     88     no   I2-I12 
## tau^2.6     0.0024  0.0493     88     no   I2-I17 
## tau^2.7     0.0142  0.1192     88     no    I2-I4 
## tau^2.8     0.0041  0.0637     88     no   I4-I11 
## tau^2.9     0.0048  0.0692     88     no   I4-I12 
## tau^2.10    0.0051  0.0712     88     no   I4-I17 
## rho         0.0000                   yes          
## 
## outer factor: StudyID      (nlvls = 62)
## inner factor: factor(Cell) (nlvls = 10)
## 
##             estim    sqrt  fixed 
## gamma^2    0.0129  0.1135     no 
## phi        0.9130             no 
## 
## Test for Residual Heterogeneity:
## QE(df = 870) = 6548.1258, p-val < .0001
## 
## Number of estimates:   880
## Number of clusters:    62
## Estimates per cluster: 10-40 (mean: 14.19, median: 10)
## 
## Test of Moderators (coefficients 1:10):¹
## F(df1 = 10, df2 = 45.33) = 113.7651, p-val < .0001
## 
## Model Results:
## 
##                      estimate      se¹     tval¹     df¹    pval¹   ci.lb¹ 
## factor(Cell)I11-I12    0.3315  0.0155   21.3424   60.16   <.0001   0.3004  
## factor(Cell)I11-I17    0.3654  0.0152   24.0058   60.15   <.0001   0.3350  
## factor(Cell)I12-I17    0.2925  0.0165   17.6884   60.14   <.0001   0.2594  
## factor(Cell)I2-I11     0.4879  0.0153   31.9513   60.13   <.0001   0.4574  
## factor(Cell)I2-I12     0.3753  0.0166   22.6473   60.09   <.0001   0.3421  
## factor(Cell)I2-I17     0.4124  0.0166   24.8240      60   <.0001   0.3792  
## factor(Cell)I2-I4      0.4636  0.0230   20.1622   58.26   <.0001   0.4175  
## factor(Cell)I4-I11     0.3764  0.0177   21.2930   59.79   <.0001   0.3411  
## factor(Cell)I4-I12     0.3003  0.0188   15.9632   59.69   <.0001   0.2626  
## factor(Cell)I4-I17     0.3378  0.0200   16.8813   59.65   <.0001   0.2977  
##                       ci.ub¹      
## factor(Cell)I11-I12  0.3626   *** 
## factor(Cell)I11-I17  0.3959   *** 
## factor(Cell)I12-I17  0.3255   *** 
## factor(Cell)I2-I11   0.5185   *** 
## factor(Cell)I2-I12   0.4084   *** 
## factor(Cell)I2-I17   0.4456   *** 
## factor(Cell)I2-I4    0.5096   *** 
## factor(Cell)I4-I11   0.4118   *** 
## factor(Cell)I4-I12   0.3379   *** 
## factor(Cell)I4-I17   0.3778   *** 
## 
## ---
## Signif. codes:  0 '***' 0.001 '**' 0.01 '*' 0.05 '.' 0.1 ' ' 1
## 
## 1) results based on cluster-robust inference (var-cov estimator: CR2,
##    approx t/F-tests and confidence intervals, df: Satterthwaite approx)
```

#### Correlation-specific effect sizes and between-study heterogeneity, overall within-study heterogeneity (`Model 3`)

Model `mlmvrem3` specifies study-specific random-effects
per correlation coefficient and assumes only one variance estimate at
the level of effect sizes (aka `Model 3`).

```
## Random effects: rho = 0, phi = 0
## Model specification
tas20.mlmvrem3 <- rma.mv(z,
                         V, 
                         data = tas20,
                         random = list(~ factor(Cell) | ESID,
                                       ~ factor(Cell) | StudyID),
                         struc = c("CS", "HCS"),
                         rho = 0,
                         phi = 0,
                         method = "REML",
                         mods = ~ factor(Cell) - 1,
                         time = TRUE,
                         sparse = TRUE)
```

```
## 
## Processing time: 0 hours, 0 minutes, 7.46 seconds
```

```
## Model summary
summary(tas20.mlmvrem3)
```

```
## 
## Multivariate Meta-Analysis Model (k = 880; method: REML)
## 
##     logLik    Deviance         AIC         BIC        AICc   
##   602.3316  -1204.6631  -1162.6631  -1062.5248  -1161.5735   
## 
## Variance Components:
## 
## outer factor: ESID         (nlvls = 880)
## inner factor: factor(Cell) (nlvls = 10)
## 
##             estim    sqrt  fixed 
## tau^2      0.0032  0.0566     no 
## rho        0.0000            yes 
## 
## outer factor: StudyID      (nlvls = 62)
## inner factor: factor(Cell) (nlvls = 10)
## 
##                estim    sqrt  k.lvl  fixed    level 
## gamma^2.1     0.0039  0.0623     88     no  I11-I12 
## gamma^2.2     0.0038  0.0614     88     no  I11-I17 
## gamma^2.3     0.0055  0.0741     88     no  I12-I17 
## gamma^2.4     0.0042  0.0645     88     no   I2-I11 
## gamma^2.5     0.0055  0.0744     88     no   I2-I12 
## gamma^2.6     0.0062  0.0790     88     no   I2-I17 
## gamma^2.7     0.0234  0.1530     88     no    I2-I4 
## gamma^2.8     0.0090  0.0947     88     no   I4-I11 
## gamma^2.9     0.0110  0.1051     88     no   I4-I12 
## gamma^2.10    0.0115  0.1074     88     no   I4-I17 
## phi           0.0000                   yes          
## 
## Test for Residual Heterogeneity:
## QE(df = 870) = 6548.1258, p-val < .0001
## 
## Test of Moderators (coefficients 1:10):
## QM(df = 10) = 5479.6903, p-val < .0001
## 
## Model Results:
## 
##                      estimate      se     zval    pval   ci.lb   ci.ub      
## factor(Cell)I11-I12    0.3198  0.0117  27.4060  <.0001  0.2970  0.3427  *** 
## factor(Cell)I11-I17    0.3559  0.0116  30.7006  <.0001  0.3332  0.3786  *** 
## factor(Cell)I12-I17    0.2833  0.0128  22.1328  <.0001  0.2582  0.3083  *** 
## factor(Cell)I2-I11     0.4772  0.0119  40.1818  <.0001  0.4539  0.5004  *** 
## factor(Cell)I2-I12     0.3626  0.0128  28.2906  <.0001  0.3375  0.3877  *** 
## factor(Cell)I2-I17     0.4016  0.0133  30.2631  <.0001  0.3756  0.4276  *** 
## factor(Cell)I2-I4      0.4526  0.0214  21.1586  <.0001  0.4107  0.4946  *** 
## factor(Cell)I4-I11     0.3617  0.0149  24.2927  <.0001  0.3325  0.3908  *** 
## factor(Cell)I4-I12     0.2845  0.0160  17.7901  <.0001  0.2531  0.3158  *** 
## factor(Cell)I4-I17     0.3222  0.0162  19.8327  <.0001  0.2903  0.3540  *** 
## 
## ---
## Signif. codes:  0 '***' 0.001 '**' 0.01 '*' 0.05 '.' 0.1 ' ' 1
```

```
outztor(tas20.mlmvrem3)
```

```
##                      Estimate         SE   CI95low   CI95upp
## factor(Cell)I11-I12 0.3093548 0.01166960 0.2885270 0.3298900
## factor(Cell)I11-I17 0.3416169 0.01159279 0.3213927 0.3615296
## factor(Cell)I12-I17 0.2759184 0.01279746 0.2525876 0.2989286
## factor(Cell)I2-I11  0.4439714 0.01187463 0.4250925 0.4624642
## factor(Cell)I2-I12  0.3475189 0.01281696 0.3252409 0.3694113
## factor(Cell)I2-I17  0.3813274 0.01326992 0.3588818 0.4033321
## factor(Cell)I2-I4   0.4240581 0.02138892 0.3890683 0.4578261
## factor(Cell)I4-I11  0.3466762 0.01488656 0.3207489 0.3720843
## factor(Cell)I4-I12  0.2770483 0.01598967 0.2478683 0.3057261
## factor(Cell)I4-I17  0.3114511 0.01624205 0.2824247 0.3399077
```

```
## Cluster-robust standard errors
tas20.mlmvrem3.robust <- robust(tas20.mlmvrem3, 
                                cluster = StudyID, 
                                clubSandwich = TRUE)

summary(tas20.mlmvrem3.robust)
```

```
## 
## Multivariate Meta-Analysis Model (k = 880; method: REML)
## 
##     logLik    Deviance         AIC         BIC        AICc   
##   602.3316  -1204.6631  -1162.6631  -1062.5248  -1161.5735   
## 
## Variance Components:
## 
## outer factor: ESID         (nlvls = 880)
## inner factor: factor(Cell) (nlvls = 10)
## 
##             estim    sqrt  fixed 
## tau^2      0.0032  0.0566     no 
## rho        0.0000            yes 
## 
## outer factor: StudyID      (nlvls = 62)
## inner factor: factor(Cell) (nlvls = 10)
## 
##                estim    sqrt  k.lvl  fixed    level 
## gamma^2.1     0.0039  0.0623     88     no  I11-I12 
## gamma^2.2     0.0038  0.0614     88     no  I11-I17 
## gamma^2.3     0.0055  0.0741     88     no  I12-I17 
## gamma^2.4     0.0042  0.0645     88     no   I2-I11 
## gamma^2.5     0.0055  0.0744     88     no   I2-I12 
## gamma^2.6     0.0062  0.0790     88     no   I2-I17 
## gamma^2.7     0.0234  0.1530     88     no    I2-I4 
## gamma^2.8     0.0090  0.0947     88     no   I4-I11 
## gamma^2.9     0.0110  0.1051     88     no   I4-I12 
## gamma^2.10    0.0115  0.1074     88     no   I4-I17 
## phi           0.0000                   yes          
## 
## Test for Residual Heterogeneity:
## QE(df = 870) = 6548.1258, p-val < .0001
## 
## Number of estimates:   880
## Number of clusters:    62
## Estimates per cluster: 10-40 (mean: 14.19, median: 10)
## 
## Test of Moderators (coefficients 1:10):¹
## F(df1 = 10, df2 = 49.84) = 120.2891, p-val < .0001
## 
## Model Results:
## 
##                      estimate      se¹     tval¹     df¹    pval¹   ci.lb¹ 
## factor(Cell)I11-I12    0.3198  0.0145   21.9847   56.97   <.0001   0.2907  
## factor(Cell)I11-I17    0.3559  0.0143   24.8085   56.86   <.0001   0.3272  
## factor(Cell)I12-I17    0.2833  0.0159   17.8461   58.12   <.0001   0.2515  
## factor(Cell)I2-I11     0.4772  0.0144   33.1870   57.21   <.0001   0.4484  
## factor(Cell)I2-I12     0.3626  0.0160   22.6229   58.17   <.0001   0.3305  
## factor(Cell)I2-I17     0.4016  0.0162   24.7539   58.51   <.0001   0.3691  
## factor(Cell)I2-I4      0.4526  0.0241   18.7887   60.59   <.0001   0.4045  
## factor(Cell)I4-I11     0.3617  0.0176   20.5890   59.39   <.0001   0.3265  
## factor(Cell)I4-I12     0.2845  0.0189   15.0428   59.79   <.0001   0.2467  
## factor(Cell)I4-I17     0.3222  0.0193   16.7310   59.86   <.0001   0.2836  
##                       ci.ub¹      
## factor(Cell)I11-I12  0.3490   *** 
## factor(Cell)I11-I17  0.3847   *** 
## factor(Cell)I12-I17  0.3150   *** 
## factor(Cell)I2-I11   0.5060   *** 
## factor(Cell)I2-I12   0.3947   *** 
## factor(Cell)I2-I17   0.4341   *** 
## factor(Cell)I2-I4    0.5008   *** 
## factor(Cell)I4-I11   0.3968   *** 
## factor(Cell)I4-I12   0.3223   *** 
## factor(Cell)I4-I17   0.3607   *** 
## 
## ---
## Signif. codes:  0 '***' 0.001 '**' 0.01 '*' 0.05 '.' 0.1 ' ' 1
## 
## 1) results based on cluster-robust inference (var-cov estimator: CR2,
##    approx t/F-tests and confidence intervals, df: Satterthwaite approx)
```

```
## Sensitivity analysis
## Random effects: rho = 0.5, phi = 0.5
## Model specification
tas20.mlmvrem3.sens <- rma.mv(z,
                              V, 
                              data = tas20,
                              random = list(~ factor(Cell) | ESID,
                                            ~ factor(Cell) | StudyID),
                              struc = c("CS", "HCS"),
                              rho = 0.5,
                              phi = 0.5,
                              method = "REML",
                              mods = ~ factor(Cell) - 1,
                              time = TRUE,
                              sparse = TRUE)
```

```
## 
## Processing time: 0 hours, 0 minutes, 24.46 seconds
```

```
## Model summary
summary(tas20.mlmvrem3.sens)
```

```
## 
## Multivariate Meta-Analysis Model (k = 880; method: REML)
## 
##     logLik    Deviance         AIC         BIC        AICc   
##   774.5270  -1549.0540  -1507.0540  -1406.9156  -1505.9644   
## 
## Variance Components:
## 
## outer factor: ESID         (nlvls = 880)
## inner factor: factor(Cell) (nlvls = 10)
## 
##             estim    sqrt  fixed 
## tau^2      0.0019  0.0437     no 
## rho        0.5000            yes 
## 
## outer factor: StudyID      (nlvls = 62)
## inner factor: factor(Cell) (nlvls = 10)
## 
##                estim    sqrt  k.lvl  fixed    level 
## gamma^2.1     0.0049  0.0702     88     no  I11-I12 
## gamma^2.2     0.0048  0.0696     88     no  I11-I17 
## gamma^2.3     0.0064  0.0801     88     no  I12-I17 
## gamma^2.4     0.0050  0.0707     88     no   I2-I11 
## gamma^2.5     0.0063  0.0793     88     no   I2-I12 
## gamma^2.6     0.0064  0.0802     88     no   I2-I17 
## gamma^2.7     0.0183  0.1354     88     no    I2-I4 
## gamma^2.8     0.0087  0.0931     88     no   I4-I11 
## gamma^2.9     0.0099  0.0996     88     no   I4-I12 
## gamma^2.10    0.0103  0.1017     88     no   I4-I17 
## phi           0.5000                   yes          
## 
## Test for Residual Heterogeneity:
## QE(df = 870) = 6548.1258, p-val < .0001
## 
## Test of Moderators (coefficients 1:10):
## QM(df = 10) = 2307.4597, p-val < .0001
## 
## Model Results:
## 
##                      estimate      se     zval    pval   ci.lb   ci.ub      
## factor(Cell)I11-I12    0.3266  0.0117  27.8059  <.0001  0.3036  0.3496  *** 
## factor(Cell)I11-I17    0.3619  0.0117  30.9772  <.0001  0.3390  0.3848  *** 
## factor(Cell)I12-I17    0.2893  0.0128  22.6562  <.0001  0.2643  0.3143  *** 
## factor(Cell)I2-I11     0.4835  0.0118  41.0036  <.0001  0.4604  0.5066  *** 
## factor(Cell)I2-I12     0.3700  0.0127  29.1622  <.0001  0.3452  0.3949  *** 
## factor(Cell)I2-I17     0.4080  0.0128  31.9125  <.0001  0.3829  0.4330  *** 
## factor(Cell)I2-I4      0.4605  0.0190  24.2744  <.0001  0.4233  0.4977  *** 
## factor(Cell)I4-I11     0.3709  0.0142  26.1877  <.0001  0.3432  0.3987  *** 
## factor(Cell)I4-I12     0.2943  0.0149  19.7805  <.0001  0.2652  0.3235  *** 
## factor(Cell)I4-I17     0.3319  0.0151  21.9552  <.0001  0.3023  0.3616  *** 
## 
## ---
## Signif. codes:  0 '***' 0.001 '**' 0.01 '*' 0.05 '.' 0.1 ' ' 1
```

```
outztor(tas20.mlmvrem3.sens)
```

```
##                      Estimate         SE   CI95low   CI95upp
## factor(Cell)I11-I12 0.3154780 0.01174580 0.2945990 0.3360559
## factor(Cell)I11-I17 0.3469104 0.01168312 0.3266090 0.3668918
## factor(Cell)I12-I17 0.2814924 0.01276853 0.2582896 0.3043705
## factor(Cell)I2-I11  0.4490441 0.01179117 0.4304028 0.4673025
## factor(Cell)I2-I12  0.3540215 0.01268813 0.3320802 0.3755800
## factor(Cell)I2-I17  0.3867520 0.01278350 0.3652393 0.4078518
## factor(Cell)I2-I4   0.4305126 0.01896936 0.3997422 0.4603139
## factor(Cell)I4-I11  0.3548194 0.01416398 0.3303168 0.3788440
## factor(Cell)I4-I12  0.2861221 0.01487907 0.2591275 0.3126700
## factor(Cell)I4-I17  0.3202716 0.01511824 0.2934309 0.3466078
```

```
## Cluster-robust standard errors
summary(
  robust(tas20.mlmvrem3.sens,
         cluster = StudyID,
         clubSandwich = TRUE)
)
```

```
## 
## Multivariate Meta-Analysis Model (k = 880; method: REML)
## 
##     logLik    Deviance         AIC         BIC        AICc   
##   774.5270  -1549.0540  -1507.0540  -1406.9156  -1505.9644   
## 
## Variance Components:
## 
## outer factor: ESID         (nlvls = 880)
## inner factor: factor(Cell) (nlvls = 10)
## 
##             estim    sqrt  fixed 
## tau^2      0.0019  0.0437     no 
## rho        0.5000            yes 
## 
## outer factor: StudyID      (nlvls = 62)
## inner factor: factor(Cell) (nlvls = 10)
## 
##                estim    sqrt  k.lvl  fixed    level 
## gamma^2.1     0.0049  0.0702     88     no  I11-I12 
## gamma^2.2     0.0048  0.0696     88     no  I11-I17 
## gamma^2.3     0.0064  0.0801     88     no  I12-I17 
## gamma^2.4     0.0050  0.0707     88     no   I2-I11 
## gamma^2.5     0.0063  0.0793     88     no   I2-I12 
## gamma^2.6     0.0064  0.0802     88     no   I2-I17 
## gamma^2.7     0.0183  0.1354     88     no    I2-I4 
## gamma^2.8     0.0087  0.0931     88     no   I4-I11 
## gamma^2.9     0.0099  0.0996     88     no   I4-I12 
## gamma^2.10    0.0103  0.1017     88     no   I4-I17 
## phi           0.5000                   yes          
## 
## Test for Residual Heterogeneity:
## QE(df = 870) = 6548.1258, p-val < .0001
## 
## Number of estimates:   880
## Number of clusters:    62
## Estimates per cluster: 10-40 (mean: 14.19, median: 10)
## 
## Test of Moderators (coefficients 1:10):¹
## F(df1 = 10, df2 = 49.09) = 115.2220, p-val < .0001
## 
## Model Results:
## 
##                      estimate      se¹     tval¹     df¹    pval¹   ci.lb¹ 
## factor(Cell)I11-I12    0.3266  0.0151   21.6864    57.8   <.0001   0.2965  
## factor(Cell)I11-I17    0.3619  0.0149   24.3299   57.74   <.0001   0.3321  
## factor(Cell)I12-I17    0.2893  0.0163   17.7284   58.62   <.0001   0.2566  
## factor(Cell)I2-I11     0.4835  0.0149   32.5412   57.84   <.0001   0.4538  
## factor(Cell)I2-I12     0.3700  0.0163   22.6934   58.58   <.0001   0.3374  
## factor(Cell)I2-I17     0.4080  0.0164   24.9176   58.63   <.0001   0.3752  
## factor(Cell)I2-I4      0.4605  0.0243   18.9386   60.44   <.0001   0.4119  
## factor(Cell)I4-I11     0.3709  0.0179   20.6802   59.38   <.0001   0.3351  
## factor(Cell)I4-I12     0.2943  0.0191   15.3906   59.65   <.0001   0.2561  
## factor(Cell)I4-I17     0.3319  0.0196   16.9651   59.73   <.0001   0.2928  
##                       ci.ub¹      
## factor(Cell)I11-I12  0.3568   *** 
## factor(Cell)I11-I17  0.3917   *** 
## factor(Cell)I12-I17  0.3220   *** 
## factor(Cell)I2-I11   0.5132   *** 
## factor(Cell)I2-I12   0.4027   *** 
## factor(Cell)I2-I17   0.4407   *** 
## factor(Cell)I2-I4    0.5092   *** 
## factor(Cell)I4-I11   0.4068   *** 
## factor(Cell)I4-I12   0.3326   *** 
## factor(Cell)I4-I17   0.3711   *** 
## 
## ---
## Signif. codes:  0 '***' 0.001 '**' 0.01 '*' 0.05 '.' 0.1 ' ' 1
## 
## 1) results based on cluster-robust inference (var-cov estimator: CR2,
##    approx t/F-tests and confidence intervals, df: Satterthwaite approx)
```

```
## Sensitivity analysis
## Random effects: rho = 1, phi = 1
## Model specification
tas20.mlmvrem3.sent <- rma.mv(z,
                              V, 
                              data = tas20,
                              random = list(~ factor(Cell) | ESID,
                                            ~ factor(Cell) | StudyID),
                              struc = c("CS", "HCS"),
                              rho = 1,
                              phi = 1,
                              method = "REML",
                              mods = ~ factor(Cell) - 1,
                              time = TRUE,
                              sparse = TRUE)
```

```
## 
## Processing time: 0 hours, 0 minutes, 53.63 seconds
```

```
## Model summary
summary(tas20.mlmvrem3.sent)
```

```
## 
## Multivariate Meta-Analysis Model (k = 880; method: REML)
## 
##     logLik    Deviance         AIC         BIC        AICc   
##   801.6340  -1603.2679  -1561.2679  -1461.1296  -1560.1783   
## 
## Variance Components:
## 
## outer factor: ESID         (nlvls = 880)
## inner factor: factor(Cell) (nlvls = 10)
## 
##             estim    sqrt  fixed 
## tau^2      0.0042  0.0644     no 
## rho        1.0000            yes 
## 
## outer factor: StudyID      (nlvls = 62)
## inner factor: factor(Cell) (nlvls = 10)
## 
##                estim    sqrt  k.lvl  fixed    level 
## gamma^2.1     0.0072  0.0850     88     no  I11-I12 
## gamma^2.2     0.0072  0.0849     88     no  I11-I17 
## gamma^2.3     0.0118  0.1087     88     no  I12-I17 
## gamma^2.4     0.0063  0.0795     88     no   I2-I11 
## gamma^2.5     0.0111  0.1052     88     no   I2-I12 
## gamma^2.6     0.0103  0.1016     88     no   I2-I17 
## gamma^2.7     0.0253  0.1592     88     no    I2-I4 
## gamma^2.8     0.0157  0.1253     88     no   I4-I11 
## gamma^2.9     0.0172  0.1311     88     no   I4-I12 
## gamma^2.10    0.0196  0.1399     88     no   I4-I17 
## phi           1.0000                   yes          
## 
## Test for Residual Heterogeneity:
## QE(df = 870) = 6548.1258, p-val < .0001
## 
## Test of Moderators (coefficients 1:10):
## QM(df = 10) = 1873.8942, p-val < .0001
## 
## Model Results:
## 
##                      estimate      se     zval    pval   ci.lb   ci.ub      
## factor(Cell)I11-I12    0.3288  0.0140  23.4502  <.0001  0.3013  0.3563  *** 
## factor(Cell)I11-I17    0.3637  0.0140  25.9517  <.0001  0.3363  0.3912  *** 
## factor(Cell)I12-I17    0.2920  0.0165  17.7069  <.0001  0.2597  0.3243  *** 
## factor(Cell)I2-I11     0.4835  0.0135  35.8421  <.0001  0.4571  0.5099  *** 
## factor(Cell)I2-I12     0.3736  0.0161  23.1847  <.0001  0.3420  0.4052  *** 
## factor(Cell)I2-I17     0.4125  0.0157  26.2138  <.0001  0.3817  0.4434  *** 
## factor(Cell)I2-I4      0.4627  0.0222  20.8100  <.0001  0.4192  0.5063  *** 
## factor(Cell)I4-I11     0.3759  0.0183  20.5072  <.0001  0.3400  0.4119  *** 
## factor(Cell)I4-I12     0.3014  0.0190  15.8741  <.0001  0.2642  0.3386  *** 
## factor(Cell)I4-I17     0.3390  0.0200  16.9611  <.0001  0.2999  0.3782  *** 
## 
## ---
## Signif. codes:  0 '***' 0.001 '**' 0.01 '*' 0.05 '.' 0.1 ' ' 1
```

```
outztor(tas20.mlmvrem3.sent)
```

```
##                      Estimate         SE   CI95low   CI95upp
## factor(Cell)I11-I12 0.3174563 0.01402097 0.2925324 0.3419492
## factor(Cell)I11-I17 0.3484962 0.01401478 0.3241351 0.3723954
## factor(Cell)I12-I17 0.2839876 0.01649008 0.2540067 0.3134232
## factor(Cell)I2-I11  0.4490347 0.01348865 0.4276781 0.4698901
## factor(Cell)I2-I12  0.3571137 0.01611154 0.3292555 0.3843507
## factor(Cell)I2-I17  0.3906061 0.01573527 0.3641586 0.4164242
## factor(Cell)I2-I4   0.4323188 0.02223305 0.3962241 0.4670794
## factor(Cell)I4-I11  0.3591788 0.01833018 0.3274883 0.3900621
## factor(Cell)I4-I12  0.2925557 0.01898206 0.2581733 0.3261979
## factor(Cell)I4-I17  0.3266140 0.01998622 0.2911805 0.3611525
```

```
## Cluster-robust standard errors
summary(
  robust(tas20.mlmvrem3.sent,
         cluster = StudyID,
         clubSandwich = TRUE)
)
```

```
## 
## Multivariate Meta-Analysis Model (k = 880; method: REML)
## 
##     logLik    Deviance         AIC         BIC        AICc   
##   801.6340  -1603.2679  -1561.2679  -1461.1296  -1560.1783   
## 
## Variance Components:
## 
## outer factor: ESID         (nlvls = 880)
## inner factor: factor(Cell) (nlvls = 10)
## 
##             estim    sqrt  fixed 
## tau^2      0.0042  0.0644     no 
## rho        1.0000            yes 
## 
## outer factor: StudyID      (nlvls = 62)
## inner factor: factor(Cell) (nlvls = 10)
## 
##                estim    sqrt  k.lvl  fixed    level 
## gamma^2.1     0.0072  0.0850     88     no  I11-I12 
## gamma^2.2     0.0072  0.0849     88     no  I11-I17 
## gamma^2.3     0.0118  0.1087     88     no  I12-I17 
## gamma^2.4     0.0063  0.0795     88     no   I2-I11 
## gamma^2.5     0.0111  0.1052     88     no   I2-I12 
## gamma^2.6     0.0103  0.1016     88     no   I2-I17 
## gamma^2.7     0.0253  0.1592     88     no    I2-I4 
## gamma^2.8     0.0157  0.1253     88     no   I4-I11 
## gamma^2.9     0.0172  0.1311     88     no   I4-I12 
## gamma^2.10    0.0196  0.1399     88     no   I4-I17 
## phi           1.0000                   yes          
## 
## Test for Residual Heterogeneity:
## QE(df = 870) = 6548.1258, p-val < .0001
## 
## Number of estimates:   880
## Number of clusters:    62
## Estimates per cluster: 10-40 (mean: 14.19, median: 10)
## 
## Test of Moderators (coefficients 1:10):¹
## F(df1 = 10, df2 = 41.26) = 113.8631, p-val < .0001
## 
## Model Results:
## 
##                      estimate      se¹     tval¹     df¹    pval¹   ci.lb¹ 
## factor(Cell)I11-I12    0.3288  0.0160   20.5308   58.37   <.0001   0.2968  
## factor(Cell)I11-I17    0.3637  0.0151   24.0753   58.36   <.0001   0.3335  
## factor(Cell)I12-I17    0.2920  0.0165   17.7367   59.58   <.0001   0.2591  
## factor(Cell)I2-I11     0.4835  0.0158   30.6679   57.95   <.0001   0.4519  
## factor(Cell)I2-I12     0.3736  0.0170   21.9948   59.45   <.0001   0.3396  
## factor(Cell)I2-I17     0.4125  0.0167   24.6506   59.29   <.0001   0.3790  
## factor(Cell)I2-I4      0.4627  0.0236   19.6109   60.55   <.0001   0.4156  
## factor(Cell)I4-I11     0.3759  0.0178   21.1559   60.07   <.0001   0.3404  
## factor(Cell)I4-I12     0.3014  0.0190   15.8800   60.18   <.0001   0.2634  
## factor(Cell)I4-I17     0.3390  0.0205   16.5562   60.33   <.0001   0.2981  
##                       ci.ub¹      
## factor(Cell)I11-I12  0.3609   *** 
## factor(Cell)I11-I17  0.3940   *** 
## factor(Cell)I12-I17  0.3250   *** 
## factor(Cell)I2-I11   0.5150   *** 
## factor(Cell)I2-I12   0.4076   *** 
## factor(Cell)I2-I17   0.4460   *** 
## factor(Cell)I2-I4    0.5099   *** 
## factor(Cell)I4-I11   0.4115   *** 
## factor(Cell)I4-I12   0.3393   *** 
## factor(Cell)I4-I17   0.3800   *** 
## 
## ---
## Signif. codes:  0 '***' 0.001 '**' 0.01 '*' 0.05 '.' 0.1 ' ' 1
## 
## 1) results based on cluster-robust inference (var-cov estimator: CR2,
##    approx t/F-tests and confidence intervals, df: Satterthwaite approx)
```

```
## Sensitivity analysis
## Random effects: rho = 0, phi estimated
## Model specification
tas20.mlmvrem3.senu <- rma.mv(z,
                              V, 
                              data = tas20,
                              random = list(~ factor(Cell) | ESID,
                                            ~ factor(Cell) | StudyID),
                              struc = c("CS", "HCS"),
                              rho = 0,
                              phi = NA,
                              method = "REML",
                              mods = ~ factor(Cell) - 1,
                              time = TRUE,
                              sparse = TRUE)
```

```
## 
## Processing time: 0 hours, 0 minutes, 40.32 seconds
```

```
## Model summary
summary(tas20.mlmvrem3.senu)
```

```
## 
## Multivariate Meta-Analysis Model (k = 880; method: REML)
## 
##     logLik    Deviance         AIC         BIC        AICc   
##   810.0208  -1620.0417  -1576.0417  -1471.1348  -1574.8469   
## 
## Variance Components:
## 
## outer factor: ESID         (nlvls = 880)
## inner factor: factor(Cell) (nlvls = 10)
## 
##             estim    sqrt  fixed 
## tau^2      0.0026  0.0514     no 
## rho        0.0000            yes 
## 
## outer factor: StudyID      (nlvls = 62)
## inner factor: factor(Cell) (nlvls = 10)
## 
##                estim    sqrt  k.lvl  fixed    level 
## gamma^2.1     0.0089  0.0944     88     no  I11-I12 
## gamma^2.2     0.0085  0.0922     88     no  I11-I17 
## gamma^2.3     0.0123  0.1111     88     no  I12-I17 
## gamma^2.4     0.0085  0.0922     88     no   I2-I11 
## gamma^2.5     0.0122  0.1102     88     no   I2-I12 
## gamma^2.6     0.0116  0.1077     88     no   I2-I17 
## gamma^2.7     0.0314  0.1771     88     no    I2-I4 
## gamma^2.8     0.0162  0.1273     88     no   I4-I11 
## gamma^2.9     0.0181  0.1346     88     no   I4-I12 
## gamma^2.10    0.0199  0.1409     88     no   I4-I17 
## phi           0.8979                    no          
## 
## Test for Residual Heterogeneity:
## QE(df = 870) = 6548.1258, p-val < .0001
## 
## Test of Moderators (coefficients 1:10):
## QM(df = 10) = 1616.9596, p-val < .0001
## 
## Model Results:
## 
##                      estimate      se     zval    pval   ci.lb   ci.ub      
## factor(Cell)I11-I12    0.3295  0.0145  22.7950  <.0001  0.3012  0.3578  *** 
## factor(Cell)I11-I17    0.3643  0.0142  25.6330  <.0001  0.3364  0.3921  *** 
## factor(Cell)I12-I17    0.2922  0.0163  17.9279  <.0001  0.2603  0.3241  *** 
## factor(Cell)I2-I11     0.4854  0.0142  34.1405  <.0001  0.4575  0.5132  *** 
## factor(Cell)I2-I12     0.3740  0.0162  23.0772  <.0001  0.3422  0.4057  *** 
## factor(Cell)I2-I17     0.4119  0.0159  25.8567  <.0001  0.3807  0.4431  *** 
## factor(Cell)I2-I4      0.4643  0.0240  19.3137  <.0001  0.4172  0.5114  *** 
## factor(Cell)I4-I11     0.3756  0.0181  20.6950  <.0001  0.3400  0.4111  *** 
## factor(Cell)I4-I12     0.2999  0.0190  15.7859  <.0001  0.2627  0.3371  *** 
## factor(Cell)I4-I17     0.3374  0.0197  17.0954  <.0001  0.2987  0.3761  *** 
## 
## ---
## Signif. codes:  0 '***' 0.001 '**' 0.01 '*' 0.05 '.' 0.1 ' ' 1
```

```
outztor(tas20.mlmvrem3.senu)
```

```
##                      Estimate         SE   CI95low   CI95upp
## factor(Cell)I11-I12 0.3180785 0.01445428 0.2923885 0.3433098
## factor(Cell)I11-I17 0.3489843 0.01421065 0.3242887 0.3732045
## factor(Cell)I12-I17 0.2841606 0.01629727 0.2545363 0.3132520
## factor(Cell)I2-I11  0.4505433 0.01421625 0.4280580 0.4724712
## factor(Cell)I2-I12  0.3574468 0.01620314 0.3294358 0.3848291
## factor(Cell)I2-I17  0.3900653 0.01592786 0.3632773 0.4162088
## factor(Cell)I2-I4   0.4335615 0.02403391 0.3945349 0.4710268
## factor(Cell)I4-I11  0.3588425 0.01814519 0.3274675 0.3894271
## factor(Cell)I4-I12  0.2912079 0.01899479 0.2567744 0.3249031
## factor(Cell)I4-I17  0.3251504 0.01973354 0.2901350 0.3592964
```

```
## Cluster-robust standard errors
summary(
  robust(tas20.mlmvrem3.senu,
         cluster = StudyID,
         clubSandwich = TRUE)
)
```

```
## 
## Multivariate Meta-Analysis Model (k = 880; method: REML)
## 
##     logLik    Deviance         AIC         BIC        AICc   
##   810.0208  -1620.0417  -1576.0417  -1471.1348  -1574.8469   
## 
## Variance Components:
## 
## outer factor: ESID         (nlvls = 880)
## inner factor: factor(Cell) (nlvls = 10)
## 
##             estim    sqrt  fixed 
## tau^2      0.0026  0.0514     no 
## rho        0.0000            yes 
## 
## outer factor: StudyID      (nlvls = 62)
## inner factor: factor(Cell) (nlvls = 10)
## 
##                estim    sqrt  k.lvl  fixed    level 
## gamma^2.1     0.0089  0.0944     88     no  I11-I12 
## gamma^2.2     0.0085  0.0922     88     no  I11-I17 
## gamma^2.3     0.0123  0.1111     88     no  I12-I17 
## gamma^2.4     0.0085  0.0922     88     no   I2-I11 
## gamma^2.5     0.0122  0.1102     88     no   I2-I12 
## gamma^2.6     0.0116  0.1077     88     no   I2-I17 
## gamma^2.7     0.0314  0.1771     88     no    I2-I4 
## gamma^2.8     0.0162  0.1273     88     no   I4-I11 
## gamma^2.9     0.0181  0.1346     88     no   I4-I12 
## gamma^2.10    0.0199  0.1409     88     no   I4-I17 
## phi           0.8979                    no          
## 
## Test for Residual Heterogeneity:
## QE(df = 870) = 6548.1258, p-val < .0001
## 
## Number of estimates:   880
## Number of clusters:    62
## Estimates per cluster: 10-40 (mean: 14.19, median: 10)
## 
## Test of Moderators (coefficients 1:10):¹
## F(df1 = 10, df2 = 46.05) = 109.0840, p-val < .0001
## 
## Model Results:
## 
##                      estimate      se¹     tval¹     df¹    pval¹   ci.lb¹ 
## factor(Cell)I11-I12    0.3295  0.0157   21.0193   59.31   <.0001   0.2981  
## factor(Cell)I11-I17    0.3643  0.0151   24.1098    59.2   <.0001   0.3341  
## factor(Cell)I12-I17    0.2922  0.0164   17.7647   59.93   <.0001   0.2593  
## factor(Cell)I2-I11     0.4854  0.0154   31.5253    59.2   <.0001   0.4546  
## factor(Cell)I2-I12     0.3740  0.0167   22.4493    59.9   <.0001   0.3406  
## factor(Cell)I2-I17     0.4119  0.0166   24.8409   59.82   <.0001   0.3787  
## factor(Cell)I2-I4      0.4643  0.0242   19.1859   60.75   <.0001   0.4159  
## factor(Cell)I4-I11     0.3756  0.0179   20.9690   60.29   <.0001   0.3397  
## factor(Cell)I4-I12     0.2999  0.0191   15.6770    60.4   <.0001   0.2616  
## factor(Cell)I4-I17     0.3374  0.0199   16.9943   60.48   <.0001   0.2977  
##                       ci.ub¹      
## factor(Cell)I11-I12  0.3609   *** 
## factor(Cell)I11-I17  0.3945   *** 
## factor(Cell)I12-I17  0.3251   *** 
## factor(Cell)I2-I11   0.5162   *** 
## factor(Cell)I2-I12   0.4073   *** 
## factor(Cell)I2-I17   0.4450   *** 
## factor(Cell)I2-I4    0.5127   *** 
## factor(Cell)I4-I11   0.4114   *** 
## factor(Cell)I4-I12   0.3381   *** 
## factor(Cell)I4-I17   0.3771   *** 
## 
## ---
## Signif. codes:  0 '***' 0.001 '**' 0.01 '*' 0.05 '.' 0.1 ' ' 1
## 
## 1) results based on cluster-robust inference (var-cov estimator: CR2,
##    approx t/F-tests and confidence intervals, df: Satterthwaite approx)
```

#### Correlation-specific effect sizes, overall within- and between-study heterogeneity (`Model 4`)

This approach assumes that there are two estimates of the amounts of
residual heterogeneity which are the same across the 21 correlations.
Moreover, we assume a zero correlation between the levels. The structure
of the random effects corresponds to a compound symmetric structure (CS)
with \(\rho = \phi = 0\).

This approach differs from the so-called WPL approach in the
meta-analytic structural equation modeling literature (see Wilson et al., 2016 and Stolwijk et al.,
2022). In the WPL approach, \(\rho = \phi
= 1\), and the within-study heterogeneity contains variation
among all correlations within a study, irrespective of their type.

```
## Multilevel no-intercept random-effects model
## Effect sizes nested in articles
## Note: This model assumes a compound symmetric (CS) structure.
## Source: https://wviechtb.github.io/metafor/reference/rma.mv.html

## Model specification
tas20.mlmvrem4 <- rma.mv(z,
                         V,
                         data = tas20,
                         random = list(~ factor(Cell) | ESID,
                                       ~ factor(Cell) | StudyID),
                         method = "REML",
                         mods = ~ factor(Cell) - 1,
                         struc = c("CS", "CS"),
                         phi = 0,
                         rho = 0,
                         time = TRUE,
                         sparse = TRUE,
                         control = list(optimizer = "optimParallel", 
                                        ncpus = ncores))
```

```
## 
## Processing time: 0 hours, 0 minutes, 7.13 seconds
```

```
## Model summary
summary(tas20.mlmvrem4)
```

```
## 
## Multivariate Meta-Analysis Model (k = 880; method: REML)
## 
##     logLik    Deviance         AIC         BIC        AICc   
##   581.4644  -1162.9288  -1138.9288  -1081.7069  -1138.5647   
## 
## Variance Components:
## 
## outer factor: ESID         (nlvls = 880)
## inner factor: factor(Cell) (nlvls = 10)
## 
##             estim    sqrt  fixed 
## tau^2      0.0033  0.0578     no 
## rho        0.0000            yes 
## 
## outer factor: StudyID      (nlvls = 62)
## inner factor: factor(Cell) (nlvls = 10)
## 
##             estim    sqrt  fixed 
## gamma^2    0.0082  0.0906     no 
## phi        0.0000            yes 
## 
## Test for Residual Heterogeneity:
## QE(df = 870) = 6548.1258, p-val < .0001
## 
## Test of Moderators (coefficients 1:10):
## QM(df = 10) = 4941.7975, p-val < .0001
## 
## Model Results:
## 
##                      estimate      se     zval    pval   ci.lb   ci.ub      
## factor(Cell)I11-I12    0.3201  0.0145  22.0422  <.0001  0.2916  0.3485  *** 
## factor(Cell)I11-I17    0.3565  0.0145  24.5529  <.0001  0.3281  0.3850  *** 
## factor(Cell)I12-I17    0.2837  0.0145  19.5299  <.0001  0.2552  0.3122  *** 
## factor(Cell)I2-I11     0.4770  0.0145  32.8474  <.0001  0.4485  0.5054  *** 
## factor(Cell)I2-I12     0.3620  0.0145  24.9398  <.0001  0.3335  0.3904  *** 
## factor(Cell)I2-I17     0.4012  0.0145  27.6361  <.0001  0.3728  0.4297  *** 
## factor(Cell)I2-I4      0.4529  0.0145  31.1811  <.0001  0.4245  0.4814  *** 
## factor(Cell)I4-I11     0.3627  0.0145  24.9830  <.0001  0.3342  0.3911  *** 
## factor(Cell)I4-I12     0.2854  0.0145  19.6613  <.0001  0.2569  0.3138  *** 
## factor(Cell)I4-I17     0.3232  0.0145  22.2686  <.0001  0.2947  0.3516  *** 
## 
## ---
## Signif. codes:  0 '***' 0.001 '**' 0.01 '*' 0.05 '.' 0.1 ' ' 1
```

```
outztor(tas20.mlmvrem4)
```

```
##                      Estimate         SE   CI95low   CI95upp
## factor(Cell)I11-I12 0.3095749 0.01451999 0.2836202 0.3350764
## factor(Cell)I11-I17 0.3421712 0.01452067 0.3168013 0.3670518
## factor(Cell)I12-I17 0.2763156 0.01452483 0.2498179 0.3023998
## factor(Cell)I2-I11  0.4438248 0.01452019 0.4206839 0.4663884
## factor(Cell)I2-I12  0.3469594 0.01451325 0.3216939 0.3717311
## factor(Cell)I2-I17  0.3809803 0.01451644 0.3563967 0.4050368
## factor(Cell)I2-I4   0.4243200 0.01452539 0.4006959 0.4473802
## factor(Cell)I4-I11  0.3475719 0.01451605 0.3223133 0.3723360
## factor(Cell)I4-I12  0.2778766 0.01451376 0.2514229 0.3039156
## factor(Cell)I4-I17  0.3123822 0.01451191 0.2864900 0.3378185
```

```
## Cluster-robust standard errors
tas20.mlmvrem4.robust <- robust(tas20.mlmvrem4, 
                           cluster = StudyID, 
                           clubSandwich = TRUE)
summary(tas20.mlmvrem4.robust)
```

```
## 
## Multivariate Meta-Analysis Model (k = 880; method: REML)
## 
##     logLik    Deviance         AIC         BIC        AICc   
##   581.4644  -1162.9288  -1138.9288  -1081.7069  -1138.5647   
## 
## Variance Components:
## 
## outer factor: ESID         (nlvls = 880)
## inner factor: factor(Cell) (nlvls = 10)
## 
##             estim    sqrt  fixed 
## tau^2      0.0033  0.0578     no 
## rho        0.0000            yes 
## 
## outer factor: StudyID      (nlvls = 62)
## inner factor: factor(Cell) (nlvls = 10)
## 
##             estim    sqrt  fixed 
## gamma^2    0.0082  0.0906     no 
## phi        0.0000            yes 
## 
## Test for Residual Heterogeneity:
## QE(df = 870) = 6548.1258, p-val < .0001
## 
## Number of estimates:   880
## Number of clusters:    62
## Estimates per cluster: 10-40 (mean: 14.19, median: 10)
## 
## Test of Moderators (coefficients 1:10):¹
## F(df1 = 10, df2 = 50.27) = 125.0900, p-val < .0001
## 
## Model Results:
## 
##                      estimate      se¹     tval¹     df¹    pval¹   ci.lb¹ 
## factor(Cell)I11-I12    0.3201  0.0144   22.2349   59.17   <.0001   0.2913  
## factor(Cell)I11-I17    0.3565  0.0145   24.5383   59.17   <.0001   0.3275  
## factor(Cell)I12-I17    0.2837  0.0160   17.7259   59.15   <.0001   0.2517  
## factor(Cell)I2-I11     0.4770  0.0144   33.1356   59.17   <.0001   0.4482  
## factor(Cell)I2-I12     0.3620  0.0160   22.6316   59.18   <.0001   0.3300  
## factor(Cell)I2-I17     0.4012  0.0163   24.5391   59.18   <.0001   0.3685  
## factor(Cell)I2-I4      0.4529  0.0235   19.2728   59.15   <.0001   0.4059  
## factor(Cell)I4-I11     0.3627  0.0175   20.7508   59.17   <.0001   0.3277  
## factor(Cell)I4-I12     0.2854  0.0189   15.1264   59.19   <.0001   0.2476  
## factor(Cell)I4-I17     0.3232  0.0193   16.7799   59.19   <.0001   0.2846  
##                       ci.ub¹      
## factor(Cell)I11-I12  0.3489   *** 
## factor(Cell)I11-I17  0.3856   *** 
## factor(Cell)I12-I17  0.3157   *** 
## factor(Cell)I2-I11   0.5058   *** 
## factor(Cell)I2-I12   0.3940   *** 
## factor(Cell)I2-I17   0.4339   *** 
## factor(Cell)I2-I4    0.5000   *** 
## factor(Cell)I4-I11   0.3977   *** 
## factor(Cell)I4-I12   0.3231   *** 
## factor(Cell)I4-I17   0.3617   *** 
## 
## ---
## Signif. codes:  0 '***' 0.001 '**' 0.01 '*' 0.05 '.' 0.1 ' ' 1
## 
## 1) results based on cluster-robust inference (var-cov estimator: CR2,
##    approx t/F-tests and confidence intervals, df: Satterthwaite approx)
```

```
## Sensitivity analysis
## Rho = 1, phi = 1
## Model specification
tas20.mlmvrem4.sent <- rma.mv(z,
                              V,
                              data = tas20,
                              random = list(~ factor(Cell) | ESID,
                                            ~ factor(Cell) | StudyID),
                              struc = c("CS", "CS"),
                              rho = 1,
                              phi = 1,
                              method = "REML",
                              mods = ~ factor(Cell) - 1,
                              time = TRUE,
                              sparse = TRUE,
                              control = list(optimizer = "optimParallel", 
                                             ncpus = ncores))
```

```
## 
## Processing time: 0 hours, 0 minutes, 8.63 seconds
```

```
## Model summary
summary(tas20.mlmvrem4.sent)
```

```
## 
## Multivariate Meta-Analysis Model (k = 880; method: REML)
## 
##     logLik    Deviance         AIC         BIC        AICc   
##   778.7508  -1557.5017  -1533.5017  -1476.2798  -1533.1376   
## 
## Variance Components:
## 
## outer factor: ESID         (nlvls = 880)
## inner factor: factor(Cell) (nlvls = 10)
## 
##             estim    sqrt  fixed 
## tau^2      0.0045  0.0673     no 
## rho        1.0000            yes 
## 
## outer factor: StudyID      (nlvls = 62)
## inner factor: factor(Cell) (nlvls = 10)
## 
##             estim    sqrt  fixed 
## gamma^2    0.0129  0.1135     no 
## phi        1.0000            yes 
## 
## Test for Residual Heterogeneity:
## QE(df = 870) = 6548.1258, p-val < .0001
## 
## Test of Moderators (coefficients 1:10):
## QM(df = 10) = 1071.4297, p-val < .0001
## 
## Model Results:
## 
##                      estimate      se     zval    pval   ci.lb   ci.ub      
## factor(Cell)I11-I12    0.3298  0.0172  19.2037  <.0001  0.2961  0.3635  *** 
## factor(Cell)I11-I17    0.3647  0.0172  21.2380  <.0001  0.3310  0.3983  *** 
## factor(Cell)I12-I17    0.2927  0.0172  17.0495  <.0001  0.2591  0.3264  *** 
## factor(Cell)I2-I11     0.4839  0.0172  28.1813  <.0001  0.4502  0.5175  *** 
## factor(Cell)I2-I12     0.3736  0.0172  21.7595  <.0001  0.3399  0.4072  *** 
## factor(Cell)I2-I17     0.4124  0.0172  24.0163  <.0001  0.3788  0.4461  *** 
## factor(Cell)I2-I4      0.4641  0.0172  27.0321  <.0001  0.4305  0.4978  *** 
## factor(Cell)I4-I11     0.3782  0.0172  22.0318  <.0001  0.3446  0.4119  *** 
## factor(Cell)I4-I12     0.3023  0.0172  17.6066  <.0001  0.2686  0.3359  *** 
## factor(Cell)I4-I17     0.3402  0.0172  19.8179  <.0001  0.3066  0.3739  *** 
## 
## ---
## Signif. codes:  0 '***' 0.001 '**' 0.01 '*' 0.05 '.' 0.1 ' ' 1
```

```
outztor(tas20.mlmvrem4.sent)
```

```
##                      Estimate         SE   CI95low   CI95upp
## factor(Cell)I11-I12 0.3183371 0.01717183 0.2877727 0.3482538
## factor(Cell)I11-I17 0.3493417 0.01717010 0.3194528 0.3785361
## factor(Cell)I12-I17 0.2846433 0.01716762 0.2534313 0.3152632
## factor(Cell)I2-I11  0.4493548 0.01716897 0.4220944 0.4758034
## factor(Cell)I2-I12  0.3571238 0.01716714 0.3274194 0.3861230
## factor(Cell)I2-I17  0.3905257 0.01717086 0.3616324 0.4186696
## factor(Cell)I2-I4   0.4334226 0.01716688 0.4057002 0.4603482
## factor(Cell)I4-I11  0.3611668 0.01716567 0.3315598 0.3900631
## factor(Cell)I4-I12  0.2934075 0.01716746 0.2623587 0.3238494
## factor(Cell)I4-I17  0.3276953 0.01716687 0.2973357 0.3573929
```

```
## Cluster-robust standard errors
summary(
  robust(tas20.mlmvrem4.sent,
         cluster = StudyID,
         clubSandwich = TRUE)
)
```

```
## 
## Multivariate Meta-Analysis Model (k = 880; method: REML)
## 
##     logLik    Deviance         AIC         BIC        AICc   
##   778.7508  -1557.5017  -1533.5017  -1476.2798  -1533.1376   
## 
## Variance Components:
## 
## outer factor: ESID         (nlvls = 880)
## inner factor: factor(Cell) (nlvls = 10)
## 
##             estim    sqrt  fixed 
## tau^2      0.0045  0.0673     no 
## rho        1.0000            yes 
## 
## outer factor: StudyID      (nlvls = 62)
## inner factor: factor(Cell) (nlvls = 10)
## 
##             estim    sqrt  fixed 
## gamma^2    0.0129  0.1135     no 
## phi        1.0000            yes 
## 
## Test for Residual Heterogeneity:
## QE(df = 870) = 6548.1258, p-val < .0001
## 
## Number of estimates:   880
## Number of clusters:    62
## Estimates per cluster: 10-40 (mean: 14.19, median: 10)
## 
## Test of Moderators (coefficients 1:10):¹
## F(df1 = 10, df2 = 41.5) = 105.7770, p-val < .0001
## 
## Model Results:
## 
##                      estimate      se¹     tval¹     df¹    pval¹   ci.lb¹ 
## factor(Cell)I11-I12    0.3298  0.0160   20.6102   59.67   <.0001   0.2978  
## factor(Cell)I11-I17    0.3647  0.0153   23.7974   59.67   <.0001   0.3340  
## factor(Cell)I12-I17    0.2927  0.0164   17.8330   59.68   <.0001   0.2599  
## factor(Cell)I2-I11     0.4839  0.0161   30.0451   59.68   <.0001   0.4517  
## factor(Cell)I2-I12     0.3736  0.0168   22.2168   59.68   <.0001   0.3399  
## factor(Cell)I2-I17     0.4124  0.0169   24.4373   59.67   <.0001   0.3787  
## factor(Cell)I2-I4      0.4641  0.0228   20.3476   59.68   <.0001   0.4185  
## factor(Cell)I4-I11     0.3782  0.0176   21.4378   59.68   <.0001   0.3429  
## factor(Cell)I4-I12     0.3023  0.0188   16.0475   59.68   <.0001   0.2646  
## factor(Cell)I4-I17     0.3402  0.0205   16.5918   59.68   <.0001   0.2992  
##                       ci.ub¹      
## factor(Cell)I11-I12  0.3618   *** 
## factor(Cell)I11-I17  0.3954   *** 
## factor(Cell)I12-I17  0.3256   *** 
## factor(Cell)I2-I11   0.5161   *** 
## factor(Cell)I2-I12   0.4072   *** 
## factor(Cell)I2-I17   0.4462   *** 
## factor(Cell)I2-I4    0.5097   *** 
## factor(Cell)I4-I11   0.4135   *** 
## factor(Cell)I4-I12   0.3400   *** 
## factor(Cell)I4-I17   0.3813   *** 
## 
## ---
## Signif. codes:  0 '***' 0.001 '**' 0.01 '*' 0.05 '.' 0.1 ' ' 1
## 
## 1) results based on cluster-robust inference (var-cov estimator: CR2,
##    approx t/F-tests and confidence intervals, df: Satterthwaite approx)
```

```
## Sensitivity analysis
## Rho = 0.5, phi = 0.5
## Model specification
tas20.mlmvrem4.sens <- rma.mv(z,
                              V,
                              data = tas20,
                              random = list(~ factor(Cell) | ESID,
                                            ~ factor(Cell) | StudyID),
                              struc = c("CS", "CS"),
                              rho = 0.5,
                              phi = 0.5,
                              method = "REML",
                              mods = ~ factor(Cell) - 1,
                              time = TRUE,
                              sparse = TRUE,
                              control = list(optimizer = "optimParallel", 
                                             ncpus = ncores))
```

```
## 
## Processing time: 0 hours, 0 minutes, 7.5 seconds
```

```
## Model summary
summary(tas20.mlmvrem4.sens)
```

```
## 
## Multivariate Meta-Analysis Model (k = 880; method: REML)
## 
##     logLik    Deviance         AIC         BIC        AICc   
##   751.5904  -1503.1808  -1479.1808  -1421.9589  -1478.8167   
## 
## Variance Components:
## 
## outer factor: ESID         (nlvls = 880)
## inner factor: factor(Cell) (nlvls = 10)
## 
##             estim    sqrt  fixed 
## tau^2      0.0021  0.0458     no 
## rho        0.5000            yes 
## 
## outer factor: StudyID      (nlvls = 62)
## inner factor: factor(Cell) (nlvls = 10)
## 
##             estim    sqrt  fixed 
## gamma^2    0.0082  0.0908     no 
## phi        0.5000            yes 
## 
## Test for Residual Heterogeneity:
## QE(df = 870) = 6548.1258, p-val < .0001
## 
## Test of Moderators (coefficients 1:10):
## QM(df = 10) = 1855.2697, p-val < .0001
## 
## Model Results:
## 
##                      estimate      se     zval    pval   ci.lb   ci.ub      
## factor(Cell)I11-I12    0.3275  0.0140  23.3756  <.0001  0.3000  0.3549  *** 
## factor(Cell)I11-I17    0.3632  0.0140  25.9259  <.0001  0.3357  0.3907  *** 
## factor(Cell)I12-I17    0.2901  0.0140  20.7099  <.0001  0.2627  0.3176  *** 
## factor(Cell)I2-I11     0.4839  0.0140  34.5425  <.0001  0.4564  0.5113  *** 
## factor(Cell)I2-I12     0.3698  0.0140  26.4071  <.0001  0.3424  0.3973  *** 
## factor(Cell)I2-I17     0.4080  0.0140  29.1245  <.0001  0.3805  0.4354  *** 
## factor(Cell)I2-I4      0.4607  0.0140  32.8838  <.0001  0.4332  0.4881  *** 
## factor(Cell)I4-I11     0.3722  0.0140  26.5790  <.0001  0.3448  0.3997  *** 
## factor(Cell)I4-I12     0.2952  0.0140  21.0785  <.0001  0.2677  0.3226  *** 
## factor(Cell)I4-I17     0.3329  0.0140  23.7741  <.0001  0.3055  0.3603  *** 
## 
## ---
## Signif. codes:  0 '***' 0.001 '**' 0.01 '*' 0.05 '.' 0.1 ' ' 1
```

```
outztor(tas20.mlmvrem4.sens)
```

```
##                      Estimate         SE   CI95low   CI95upp
## factor(Cell)I11-I12 0.3162578 0.01400872 0.2913357 0.3407510
## factor(Cell)I11-I17 0.3480340 0.01400842 0.3236754 0.3719315
## factor(Cell)I12-I17 0.2822493 0.01400807 0.2567887 0.3073185
## factor(Cell)I2-I11  0.4493372 0.01400704 0.4271574 0.4709766
## factor(Cell)I2-I12  0.3538201 0.01400302 0.3295796 0.3775943
## factor(Cell)I2-I17  0.3867575 0.01400729 0.3631642 0.4098552
## factor(Cell)I2-I4   0.4306142 0.01400752 0.4079875 0.4527123
## factor(Cell)I4-I11  0.3559271 0.01400318 0.3317264 0.3796596
## factor(Cell)I4-I12  0.2868993 0.01400310 0.2615177 0.3118845
## factor(Cell)I4-I17  0.3211209 0.01400156 0.2962940 0.3455142
```

```
## Cluster-robust standard errors
summary(
  robust(tas20.mlmvrem4.sens,
         cluster = StudyID,
         clubSandwich = TRUE)
)
```

```
## 
## Multivariate Meta-Analysis Model (k = 880; method: REML)
## 
##     logLik    Deviance         AIC         BIC        AICc   
##   751.5904  -1503.1808  -1479.1808  -1421.9589  -1478.8167   
## 
## Variance Components:
## 
## outer factor: ESID         (nlvls = 880)
## inner factor: factor(Cell) (nlvls = 10)
## 
##             estim    sqrt  fixed 
## tau^2      0.0021  0.0458     no 
## rho        0.5000            yes 
## 
## outer factor: StudyID      (nlvls = 62)
## inner factor: factor(Cell) (nlvls = 10)
## 
##             estim    sqrt  fixed 
## gamma^2    0.0082  0.0908     no 
## phi        0.5000            yes 
## 
## Test for Residual Heterogeneity:
## QE(df = 870) = 6548.1258, p-val < .0001
## 
## Number of estimates:   880
## Number of clusters:    62
## Estimates per cluster: 10-40 (mean: 14.19, median: 10)
## 
## Test of Moderators (coefficients 1:10):¹
## F(df1 = 10, df2 = 49.45) = 119.4961, p-val < .0001
## 
## Model Results:
## 
##                      estimate      se¹     tval¹     df¹    pval¹   ci.lb¹ 
## factor(Cell)I11-I12    0.3275  0.0150   21.7729   59.23   <.0001   0.2974  
## factor(Cell)I11-I17    0.3632  0.0151   23.9929   59.23   <.0001   0.3329  
## factor(Cell)I12-I17    0.2901  0.0165   17.5948   59.22   <.0001   0.2571  
## factor(Cell)I2-I11     0.4839  0.0149   32.3693   59.23   <.0001   0.4540  
## factor(Cell)I2-I12     0.3698  0.0163   22.6682   59.23   <.0001   0.3372  
## factor(Cell)I2-I17     0.4080  0.0165   24.6572   59.23   <.0001   0.3749  
## factor(Cell)I2-I4      0.4607  0.0238   19.3740   59.22   <.0001   0.4131  
## factor(Cell)I4-I11     0.3722  0.0179   20.8114   59.23   <.0001   0.3364  
## factor(Cell)I4-I12     0.2952  0.0191   15.4299   59.24   <.0001   0.2569  
## factor(Cell)I4-I17     0.3329  0.0196   16.9803   59.24   <.0001   0.2937  
##                       ci.ub¹      
## factor(Cell)I11-I12  0.3576   *** 
## factor(Cell)I11-I17  0.3935   *** 
## factor(Cell)I12-I17  0.3231   *** 
## factor(Cell)I2-I11   0.5138   *** 
## factor(Cell)I2-I12   0.4024   *** 
## factor(Cell)I2-I17   0.4411   *** 
## factor(Cell)I2-I4    0.5082   *** 
## factor(Cell)I4-I11   0.4080   *** 
## factor(Cell)I4-I12   0.3335   *** 
## factor(Cell)I4-I17   0.3721   *** 
## 
## ---
## Signif. codes:  0 '***' 0.001 '**' 0.01 '*' 0.05 '.' 0.1 ' ' 1
## 
## 1) results based on cluster-robust inference (var-cov estimator: CR2,
##    approx t/F-tests and confidence intervals, df: Satterthwaite approx)
```

```
## Sensitivity analysis
## Rho = 0, phi estimated
## Model specification
tas20.mlmvrem4.senu <- rma.mv(z,
                              V,
                              data = tas20,
                              random = list(~ factor(Cell) | ESID,
                                            ~ factor(Cell) | StudyID),
                              struc = c("CS", "CS"),
                              rho = 0,
                              phi = NA,
                              method = "REML",
                              mods = ~ factor(Cell) - 1,
                              time = TRUE,
                              sparse = TRUE,
                              control = list(optimizer = "optimParallel", 
                                             ncpus = ncores))
```

```
## 
## Processing time: 0 hours, 0 minutes, 7.9 seconds
```

```
## Model summary
summary(tas20.mlmvrem4.senu)
```

```
## 
## Multivariate Meta-Analysis Model (k = 880; method: REML)
## 
##     logLik    Deviance         AIC         BIC        AICc   
##   784.1135  -1568.2269  -1542.2269  -1480.2365  -1541.8017   
## 
## Variance Components:
## 
## outer factor: ESID         (nlvls = 880)
## inner factor: factor(Cell) (nlvls = 10)
## 
##             estim    sqrt  fixed 
## tau^2      0.0031  0.0557     no 
## rho        0.0000            yes 
## 
## outer factor: StudyID      (nlvls = 62)
## inner factor: factor(Cell) (nlvls = 10)
## 
##             estim    sqrt  fixed 
## gamma^2    0.0143  0.1196     no 
## phi        0.9012             no 
## 
## Test for Residual Heterogeneity:
## QE(df = 870) = 6548.1258, p-val < .0001
## 
## Test of Moderators (coefficients 1:10):
## QM(df = 10) = 1028.6298, p-val < .0001
## 
## Model Results:
## 
##                      estimate      se     zval    pval   ci.lb   ci.ub      
## factor(Cell)I11-I12    0.3306  0.0174  18.9547  <.0001  0.2964  0.3648  *** 
## factor(Cell)I11-I17    0.3657  0.0174  20.9672  <.0001  0.3315  0.3999  *** 
## factor(Cell)I12-I17    0.2930  0.0174  16.8016  <.0001  0.2588  0.3272  *** 
## factor(Cell)I2-I11     0.4861  0.0174  27.8709  <.0001  0.4519  0.5203  *** 
## factor(Cell)I2-I12     0.3738  0.0174  21.4367  <.0001  0.3396  0.4080  *** 
## factor(Cell)I2-I17     0.4119  0.0174  23.6170  <.0001  0.3777  0.4461  *** 
## factor(Cell)I2-I4      0.4643  0.0174  26.6226  <.0001  0.4301  0.4984  *** 
## factor(Cell)I4-I11     0.3772  0.0174  21.6327  <.0001  0.3430  0.4114  *** 
## factor(Cell)I4-I12     0.3007  0.0174  17.2449  <.0001  0.2665  0.3349  *** 
## factor(Cell)I4-I17     0.3384  0.0174  19.4094  <.0001  0.3043  0.3726  *** 
## 
## ---
## Signif. codes:  0 '***' 0.001 '**' 0.01 '*' 0.05 '.' 0.1 ' ' 1
```

```
outztor(tas20.mlmvrem4.senu)
```

```
##                      Estimate         SE   CI95low   CI95upp
## factor(Cell)I11-I12 0.3190733 0.01744064 0.2880408 0.3494363
## factor(Cell)I11-I17 0.3502225 0.01743963 0.3198795 0.3798479
## factor(Cell)I12-I17 0.2849004 0.01743751 0.2531980 0.3159915
## factor(Cell)I2-I11  0.4510921 0.01743834 0.4234502 0.4778949
## factor(Cell)I2-I12  0.3573230 0.01743624 0.3271523 0.3867660
## factor(Cell)I2-I17  0.3901025 0.01743998 0.3607395 0.4186930
## factor(Cell)I2-I4   0.4335475 0.01743669 0.4053868 0.4608862
## factor(Cell)I4-I11  0.3602812 0.01743523 0.3301826 0.3896480
## factor(Cell)I4-I12  0.2919744 0.01743658 0.2604068 0.3229184
## factor(Cell)I4-I17  0.3260888 0.01743543 0.2952145 0.3562827
```

```
## Cluster-robust standard errors
summary(
  robust(tas20.mlmvrem4.senu,
         cluster = StudyID,
         clubSandwich = TRUE)
)
```

```
## 
## Multivariate Meta-Analysis Model (k = 880; method: REML)
## 
##     logLik    Deviance         AIC         BIC        AICc   
##   784.1135  -1568.2269  -1542.2269  -1480.2365  -1541.8017   
## 
## Variance Components:
## 
## outer factor: ESID         (nlvls = 880)
## inner factor: factor(Cell) (nlvls = 10)
## 
##             estim    sqrt  fixed 
## tau^2      0.0031  0.0557     no 
## rho        0.0000            yes 
## 
## outer factor: StudyID      (nlvls = 62)
## inner factor: factor(Cell) (nlvls = 10)
## 
##             estim    sqrt  fixed 
## gamma^2    0.0143  0.1196     no 
## phi        0.9012             no 
## 
## Test for Residual Heterogeneity:
## QE(df = 870) = 6548.1258, p-val < .0001
## 
## Number of estimates:   880
## Number of clusters:    62
## Estimates per cluster: 10-40 (mean: 14.19, median: 10)
## 
## Test of Moderators (coefficients 1:10):¹
## F(df1 = 10, df2 = 46.15) = 112.2878, p-val < .0001
## 
## Model Results:
## 
##                      estimate      se¹     tval¹     df¹    pval¹   ci.lb¹ 
## factor(Cell)I11-I12    0.3306  0.0157   21.1152   60.07   <.0001   0.2993  
## factor(Cell)I11-I17    0.3657  0.0154   23.7746   60.07   <.0001   0.3349  
## factor(Cell)I12-I17    0.2930  0.0166   17.6792   60.08   <.0001   0.2599  
## factor(Cell)I2-I11     0.4861  0.0155   31.2958   60.08   <.0001   0.4550  
## factor(Cell)I2-I12     0.3738  0.0166   22.4752   60.08   <.0001   0.3405  
## factor(Cell)I2-I17     0.4119  0.0168   24.5779   60.07   <.0001   0.3784  
## factor(Cell)I2-I4      0.4643  0.0234   19.8776   60.08   <.0001   0.4175  
## factor(Cell)I4-I11     0.3772  0.0178   21.1707   60.08   <.0001   0.3416  
## factor(Cell)I4-I12     0.3007  0.0191   15.7813   60.08   <.0001   0.2626  
## factor(Cell)I4-I17     0.3384  0.0199   16.9783   60.08   <.0001   0.2986  
##                       ci.ub¹      
## factor(Cell)I11-I12  0.3619   *** 
## factor(Cell)I11-I17  0.3965   *** 
## factor(Cell)I12-I17  0.3262   *** 
## factor(Cell)I2-I11   0.5171   *** 
## factor(Cell)I2-I12   0.4071   *** 
## factor(Cell)I2-I17   0.4454   *** 
## factor(Cell)I2-I4    0.5110   *** 
## factor(Cell)I4-I11   0.4128   *** 
## factor(Cell)I4-I12   0.3388   *** 
## factor(Cell)I4-I17   0.3783   *** 
## 
## ---
## Signif. codes:  0 '***' 0.001 '**' 0.01 '*' 0.05 '.' 0.1 ' ' 1
## 
## 1) results based on cluster-robust inference (var-cov estimator: CR2,
##    approx t/F-tests and confidence intervals, df: Satterthwaite approx)
```

### Model comparisons and selection

#### Rho and phi fixed to zero

```
## Likelihood-ratio tests
## Model 1 vs. Model 2
anova(tas20.mlmvrem1, tas20.mlmvrem2)
```

```
## 
##         df        AIC        BIC       AICc   logLik    LRT   pval        QE 
## Full    30 -1180.3287 -1037.2739 -1178.1118 620.1644               6548.1258 
## Reduced 21 -1193.4188 -1093.2805 -1192.3292 617.7094 4.9099 0.8421 6548.1258
```

```
## Result: Models 1 and 2 do not differ significantly.

## Model 1 vs. Model 3
anova(tas20.mlmvrem1, tas20.mlmvrem3)
```

```
## 
##         df        AIC        BIC       AICc   logLik     LRT   pval        QE 
## Full    30 -1180.3287 -1037.2739 -1178.1118 620.1644                6548.1258 
## Reduced 21 -1162.6631 -1062.5248 -1161.5735 602.3316 35.6656 <.0001 6548.1258
```

```
## Result: Model 1 is preferred over Model 3.

## Model 1 vs. Model 4
anova(tas20.mlmvrem1, tas20.mlmvrem4)
```

```
## 
##         df        AIC        BIC       AICc   logLik     LRT   pval        QE 
## Full    30 -1180.3287 -1037.2739 -1178.1118 620.1644                6548.1258 
## Reduced 12 -1138.9288 -1081.7069 -1138.5647 581.4644 77.3999 <.0001 6548.1258
```

```
## Result: Model 1 is preferred over Model 4.

## Model 2 vs. Model 4
anova(tas20.mlmvrem2, tas20.mlmvrem4)
```

```
## 
##         df        AIC        BIC       AICc   logLik     LRT   pval        QE 
## Full    21 -1193.4188 -1093.2805 -1192.3292 617.7094                6548.1258 
## Reduced 12 -1138.9288 -1081.7069 -1138.5647 581.4644 72.4900 <.0001 6548.1258
```

```
## Result: Model 2 is preferred over Model 4.

## Model 3 vs. Model 4
anova(tas20.mlmvrem3, tas20.mlmvrem4)
```

```
## 
##         df        AIC        BIC       AICc   logLik     LRT   pval        QE 
## Full    21 -1162.6631 -1062.5248 -1161.5735 602.3316                6548.1258 
## Reduced 12 -1138.9288 -1081.7069 -1138.5647 581.4644 41.7343 <.0001 6548.1258
```

```
## Result: Model 3 is preferred over Model 4.

## Result: Preference of Model 2
```

Overall, Model 2 (i.e., `mlmvrem2`) seems a reasonable
model choice. In the subsequent moderator analyses, we illustrate the
model specification and estimation using the parsimonious Model 2.

#### Rho and phi fixed to 0.5

```
## Extract the fit indices
### Create a new model fit data frame
newfit.mlmvrem1.sens <- data.frame(
  c("logLik", "parms", "k",
    "Deviance", 
    "AIC", "BIC", "AICc"),
  c(## logLik
    tas20.mlmvrem1.sens$fit.stats$REML[1],
    ## Number of parameters (parms)
    tas20.mlmvrem1.sens$parms,
    ## Number of effect sizes (k)
    length(tas20$Correlation),
    ## Deviance
    tas20.mlmvrem1.sens$fit.stats$REML[2],
    ## AIC
    tas20.mlmvrem1.sens$fit.stats$REML[3],
    ## BIC
    tas20.mlmvrem1.sens$fit.stats$REML[4],
    ## AICc
    tas20.mlmvrem1.sens$fit.stats$REML[5]))

colnames(newfit.mlmvrem1.sens) <- c("Fit criterion", "mlmvrem1.sens")
newfit.mlmvrem1.sens
```

```
##   Fit criterion mlmvrem1.sens
## 1        logLik      794.4902
## 2         parms       30.0000
## 3             k      880.0000
## 4      Deviance    -1588.9804
## 5           AIC    -1528.9804
## 6           BIC    -1385.9257
## 7          AICc    -1526.7635
```

```
### Create a new model fit data frame
newfit.mlmvrem2.sens <- data.frame(
  c("logLik", "parms", "k",
    "Deviance", 
    "AIC", "BIC", "AICc"),
  c(## logLik
    tas20.mlmvrem2.sens$fit.stats$REML[1],
    ## Number of parameters (parms)
    tas20.mlmvrem2.sens$parms,
    ## Number of effect sizes (k)
    length(tas20$Correlation),
    ## Deviance
    tas20.mlmvrem2.sens$fit.stats$REML[2],
    ## AIC
    tas20.mlmvrem2.sens$fit.stats$REML[3],
    ## BIC
    tas20.mlmvrem2.sens$fit.stats$REML[4],
    ## AICc
    tas20.mlmvrem2.sens$fit.stats$REML[5]))

colnames(newfit.mlmvrem2.sens) <- c("Fit criterion", "mlmvrem2.sens")
newfit.mlmvrem2.sens
```

```
##   Fit criterion mlmvrem2.sens
## 1        logLik      786.0989
## 2         parms       21.0000
## 3             k      880.0000
## 4      Deviance    -1572.1977
## 5           AIC    -1530.1977
## 6           BIC    -1430.0594
## 7          AICc    -1529.1081
```

```
### Create a new model fit data frame
newfit.mlmvrem3.sens <- data.frame(
  c("logLik", "parms", "k",
    "Deviance", 
    "AIC", "BIC", "AICc"),
  c(## logLik
    tas20.mlmvrem3.sens$fit.stats$REML[1],
    ## Number of parameters (parms)
    tas20.mlmvrem3.sens$parms,
    ## Number of effect sizes (k)
    length(tas20$Correlation),
    ## Deviance
    tas20.mlmvrem3.sens$fit.stats$REML[2],
    ## AIC
    tas20.mlmvrem3.sens$fit.stats$REML[3],
    ## BIC
    tas20.mlmvrem3.sens$fit.stats$REML[4],
    ## AICc
    tas20.mlmvrem3.sens$fit.stats$REML[5]))

colnames(newfit.mlmvrem3.sens) <- c("Fit criterion", "mlmvrem3.sens")
newfit.mlmvrem3.sens
```

```
##   Fit criterion mlmvrem3.sens
## 1        logLik       774.527
## 2         parms        21.000
## 3             k       880.000
## 4      Deviance     -1549.054
## 5           AIC     -1507.054
## 6           BIC     -1406.916
## 7          AICc     -1505.964
```

```
### Create a new model fit data frame
newfit.mlmvrem4.sens <- data.frame(
  c("logLik", "parms", "k",
    "Deviance", 
    "AIC", "BIC", "AICc"),
  c(## logLik
    tas20.mlmvrem4.sens$fit.stats$REML[1],
    ## Number of parameters (parms)
    tas20.mlmvrem4.sens$parms,
    ## Number of effect sizes (k)
    length(tas20$Correlation),
    ## Deviance
    tas20.mlmvrem4.sens$fit.stats$REML[2],
    ## AIC
    tas20.mlmvrem4.sens$fit.stats$REML[3],
    ## BIC
    tas20.mlmvrem4.sens$fit.stats$REML[4],
    ## AICc
    tas20.mlmvrem4.sens$fit.stats$REML[5]))

colnames(newfit.mlmvrem4.sens) <- c("Fit criterion", "mlmvrem4.sens")
newfit.mlmvrem4.sens
```

```
##   Fit criterion mlmvrem4.sens
## 1        logLik      751.5904
## 2         parms       12.0000
## 3             k      880.0000
## 4      Deviance    -1503.1808
## 5           AIC    -1479.1808
## 6           BIC    -1421.9589
## 7          AICc    -1478.8167
```

```
## Overview of the model fit
newfit.overview.sens <- cbind.data.frame(newfit.mlmvrem1.sens,
                                    newfit.mlmvrem2.sens$mlmvrem2.sens,
                                    newfit.mlmvrem3.sens$mlmvrem3.sens,
                                    newfit.mlmvrem4.sens$mlmvrem4.sens)

colnames(newfit.overview.sens) <- c("Fit criterion",
                               "Model 1",
                               "Model 2",
                               "Model 3",
                               "Model 4")

newfit.overview.sens
```

```
##   Fit criterion    Model 1    Model 2   Model 3    Model 4
## 1        logLik   794.4902   786.0989   774.527   751.5904
## 2         parms    30.0000    21.0000    21.000    12.0000
## 3             k   880.0000   880.0000   880.000   880.0000
## 4      Deviance -1588.9804 -1572.1977 -1549.054 -1503.1808
## 5           AIC -1528.9804 -1530.1977 -1507.054 -1479.1808
## 6           BIC -1385.9257 -1430.0594 -1406.916 -1421.9589
## 7          AICc -1526.7635 -1529.1081 -1505.964 -1478.8167
```

```
## Additional comparisons
anova(tas20.mlmvrem1.sens, tas20.mlmvrem2.sens)
```

```
## 
##         df        AIC        BIC       AICc   logLik     LRT   pval        QE 
## Full    30 -1528.9804 -1385.9257 -1526.7635 794.4902                6548.1258 
## Reduced 21 -1530.1977 -1430.0594 -1529.1081 786.0989 16.7827 0.0522 6548.1258
```

```
## Result: Model 1 is preferred over Model 2.

anova(tas20.mlmvrem1.sens, tas20.mlmvrem3.sens)
```

```
## 
##         df        AIC        BIC       AICc   logLik     LRT   pval        QE 
## Full    30 -1528.9804 -1385.9257 -1526.7635 794.4902                6548.1258 
## Reduced 21 -1507.0540 -1406.9156 -1505.9644 774.5270 39.9265 <.0001 6548.1258
```

```
## Result: Model 1 is preferred over Model 3.

anova(tas20.mlmvrem1.sens, tas20.mlmvrem4.sens)
```

```
## 
##         df        AIC        BIC       AICc   logLik     LRT   pval        QE 
## Full    30 -1528.9804 -1385.9257 -1526.7635 794.4902                6548.1258 
## Reduced 12 -1479.1808 -1421.9589 -1478.8167 751.5904 85.7996 <.0001 6548.1258
```

```
## Result: Model 1 is preferred over Model 4.
```

`Model 1` is favored over the other models (given the AIC,
AICs, and outcomes of LRTs).

#### Rho and phi fixed to 1.0

```
## Sensitivity analyses
## Model 1


## Rho = Phi = 1
## Random effects are considered dependent.
## Source: https://wviechtb.github.io/metafor/reference/rma.mv.html

## Model specification
tas20.mlmvrem1.sent <- rma.mv(z,
                              V, 
                              data = tas20,
                              random = list(~ factor(Cell) | ESID,
                                            ~ factor(Cell) | StudyID),
                              struc = c("HCS", "HCS"),
                              rho = 1,
                              phi = 1,
                              method = "REML",
                              mods = ~ factor(Cell) - 1,
                              time = TRUE,
                              sparse = TRUE,
                              control = list(optimizer = "optimParallel",
                                             ncpus = ncores))
```

```
## 
## Processing time: 0 hours, 0 minutes, 24.77 seconds
```

```
## Model summary
summary(tas20.mlmvrem1.sent)
```

```
## 
## Multivariate Meta-Analysis Model (k = 880; method: REML)
## 
##     logLik    Deviance         AIC         BIC        AICc   
##   825.8439  -1651.6878  -1591.6878  -1448.6330  -1589.4709   
## 
## Variance Components:
## 
## outer factor: ESID         (nlvls = 880)
## inner factor: factor(Cell) (nlvls = 10)
## 
##              estim    sqrt  k.lvl  fixed    level 
## tau^2.1     0.0023  0.0484     88     no  I11-I12 
## tau^2.2     0.0023  0.0484     88     no  I11-I17 
## tau^2.3     0.0017  0.0408     88     no  I12-I17 
## tau^2.4     0.0031  0.0558     88     no   I2-I11 
## tau^2.5     0.0023  0.0484     88     no   I2-I12 
## tau^2.6     0.0037  0.0610     88     no   I2-I17 
## tau^2.7     0.0138  0.1174     88     no    I2-I4 
## tau^2.8     0.0049  0.0699     88     no   I4-I11 
## tau^2.9     0.0052  0.0720     88     no   I4-I12 
## tau^2.10    0.0060  0.0772     88     no   I4-I17 
## rho         1.0000                   yes          
## 
## outer factor: StudyID      (nlvls = 62)
## inner factor: factor(Cell) (nlvls = 10)
## 
##                estim    sqrt  k.lvl  fixed    level 
## gamma^2.1     0.0096  0.0979     88     no  I11-I12 
## gamma^2.2     0.0090  0.0946     88     no  I11-I17 
## gamma^2.3     0.0135  0.1161     88     no  I12-I17 
## gamma^2.4     0.0077  0.0877     88     no   I2-I11 
## gamma^2.5     0.0128  0.1133     88     no   I2-I12 
## gamma^2.6     0.0111  0.1055     88     no   I2-I17 
## gamma^2.7     0.0197  0.1405     88     no    I2-I4 
## gamma^2.8     0.0133  0.1155     88     no   I4-I11 
## gamma^2.9     0.0152  0.1232     88     no   I4-I12 
## gamma^2.10    0.0167  0.1290     88     no   I4-I17 
## phi           1.0000                   yes          
## 
## Test for Residual Heterogeneity:
## QE(df = 870) = 6548.1258, p-val < .0001
## 
## Test of Moderators (coefficients 1:10):
## QM(df = 10) = 1682.7210, p-val < .0001
## 
## Model Results:
## 
##                      estimate      se     zval    pval   ci.lb   ci.ub      
## factor(Cell)I11-I12    0.3302  0.0146  22.5804  <.0001  0.3015  0.3589  *** 
## factor(Cell)I11-I17    0.3640  0.0143  25.5337  <.0001  0.3361  0.3920  *** 
## factor(Cell)I12-I17    0.2922  0.0164  17.7898  <.0001  0.2600  0.3244  *** 
## factor(Cell)I2-I11     0.4848  0.0139  34.9745  <.0001  0.4576  0.5120  *** 
## factor(Cell)I2-I12     0.3756  0.0164  22.9591  <.0001  0.3436  0.4077  *** 
## factor(Cell)I2-I17     0.4133  0.0160  25.8063  <.0001  0.3819  0.4446  *** 
## factor(Cell)I2-I4      0.4635  0.0227  20.4402  <.0001  0.4190  0.5079  *** 
## factor(Cell)I4-I11     0.3766  0.0175  21.5291  <.0001  0.3424  0.4109  *** 
## factor(Cell)I4-I12     0.3022  0.0184  16.3843  <.0001  0.2660  0.3383  *** 
## factor(Cell)I4-I17     0.3395  0.0193  17.5707  <.0001  0.3016  0.3773  *** 
## 
## ---
## Signif. codes:  0 '***' 0.001 '**' 0.01 '*' 0.05 '.' 0.1 ' ' 1
```

```
outztor(tas20.mlmvrem1.sent)
```

```
##                      Estimate         SE   CI95low   CI95upp
## factor(Cell)I11-I12 0.3187001 0.01462227 0.2927199 0.3442102
## factor(Cell)I11-I17 0.3487624 0.01425605 0.3239830 0.3730636
## factor(Cell)I12-I17 0.2841739 0.01642465 0.2543163 0.3134903
## factor(Cell)I2-I11  0.4500899 0.01386099 0.4281623 0.4714879
## factor(Cell)I2-I12  0.3589029 0.01635922 0.3306516 0.3865115
## factor(Cell)I2-I17  0.3912309 0.01601226 0.3643272 0.4174820
## factor(Cell)I2-I4   0.4329086 0.02267062 0.3961124 0.4683166
## factor(Cell)I4-I11  0.3597921 0.01749297 0.3295808 0.3892672
## factor(Cell)I4-I12  0.2933176 0.01844197 0.2599387 0.3259964
## factor(Cell)I4-I17  0.3270031 0.01931774 0.2927780 0.3603914
```

```
## Cluster-robust standard errors
summary(
  robust(tas20.mlmvrem1.sent,
         cluster = StudyID,
         clubSandwich = TRUE)
)
```

```
## 
## Multivariate Meta-Analysis Model (k = 880; method: REML)
## 
##     logLik    Deviance         AIC         BIC        AICc   
##   825.8439  -1651.6878  -1591.6878  -1448.6330  -1589.4709   
## 
## Variance Components:
## 
## outer factor: ESID         (nlvls = 880)
## inner factor: factor(Cell) (nlvls = 10)
## 
##              estim    sqrt  k.lvl  fixed    level 
## tau^2.1     0.0023  0.0484     88     no  I11-I12 
## tau^2.2     0.0023  0.0484     88     no  I11-I17 
## tau^2.3     0.0017  0.0408     88     no  I12-I17 
## tau^2.4     0.0031  0.0558     88     no   I2-I11 
## tau^2.5     0.0023  0.0484     88     no   I2-I12 
## tau^2.6     0.0037  0.0610     88     no   I2-I17 
## tau^2.7     0.0138  0.1174     88     no    I2-I4 
## tau^2.8     0.0049  0.0699     88     no   I4-I11 
## tau^2.9     0.0052  0.0720     88     no   I4-I12 
## tau^2.10    0.0060  0.0772     88     no   I4-I17 
## rho         1.0000                   yes          
## 
## outer factor: StudyID      (nlvls = 62)
## inner factor: factor(Cell) (nlvls = 10)
## 
##                estim    sqrt  k.lvl  fixed    level 
## gamma^2.1     0.0096  0.0979     88     no  I11-I12 
## gamma^2.2     0.0090  0.0946     88     no  I11-I17 
## gamma^2.3     0.0135  0.1161     88     no  I12-I17 
## gamma^2.4     0.0077  0.0877     88     no   I2-I11 
## gamma^2.5     0.0128  0.1133     88     no   I2-I12 
## gamma^2.6     0.0111  0.1055     88     no   I2-I17 
## gamma^2.7     0.0197  0.1405     88     no    I2-I4 
## gamma^2.8     0.0133  0.1155     88     no   I4-I11 
## gamma^2.9     0.0152  0.1232     88     no   I4-I12 
## gamma^2.10    0.0167  0.1290     88     no   I4-I17 
## phi           1.0000                   yes          
## 
## Test for Residual Heterogeneity:
## QE(df = 870) = 6548.1258, p-val < .0001
## 
## Number of estimates:   880
## Number of clusters:    62
## Estimates per cluster: 10-40 (mean: 14.19, median: 10)
## 
## Test of Moderators (coefficients 1:10):¹
## F(df1 = 10, df2 = 40.76) = 116.0623, p-val < .0001
## 
## Model Results:
## 
##                      estimate      se¹     tval¹     df¹    pval¹   ci.lb¹ 
## factor(Cell)I11-I12    0.3302  0.0159   20.7461   59.47   <.0001   0.2984  
## factor(Cell)I11-I17    0.3640  0.0150   24.3137   59.33   <.0001   0.3341  
## factor(Cell)I12-I17    0.2922  0.0165   17.7440   60.16   <.0001   0.2593  
## factor(Cell)I2-I11     0.4848  0.0155   31.2527   58.79   <.0001   0.4538  
## factor(Cell)I2-I12     0.3756  0.0169   22.2600      60   <.0001   0.3419  
## factor(Cell)I2-I17     0.4133  0.0167   24.7578   59.53   <.0001   0.3799  
## factor(Cell)I2-I4      0.4635  0.0232   19.9570   59.27   <.0001   0.4170  
## factor(Cell)I4-I11     0.3766  0.0176   21.4553    59.7   <.0001   0.3415  
## factor(Cell)I4-I12     0.3022  0.0188   16.0780   59.86   <.0001   0.2646  
## factor(Cell)I4-I17     0.3395  0.0206   16.5176   59.92   <.0001   0.2984  
##                       ci.ub¹      
## factor(Cell)I11-I12  0.3620   *** 
## factor(Cell)I11-I17  0.3940   *** 
## factor(Cell)I12-I17  0.3252   *** 
## factor(Cell)I2-I11   0.5159   *** 
## factor(Cell)I2-I12   0.4094   *** 
## factor(Cell)I2-I17   0.4466   *** 
## factor(Cell)I2-I4    0.5099   *** 
## factor(Cell)I4-I11   0.4118   *** 
## factor(Cell)I4-I12   0.3398   *** 
## factor(Cell)I4-I17   0.3806   *** 
## 
## ---
## Signif. codes:  0 '***' 0.001 '**' 0.01 '*' 0.05 '.' 0.1 ' ' 1
## 
## 1) results based on cluster-robust inference (var-cov estimator: CR2,
##    approx t/F-tests and confidence intervals, df: Satterthwaite approx)
```

```
## Sensitivity analyses
## Rho = 0, phi estimated
## Model specification
tas20.mlmvrem1.senu <- rma.mv(z,
                              V, 
                              data = tas20,
                              random = list(~ factor(Cell) | ESID,
                                            ~ factor(Cell) | StudyID),
                              struc = c("HCS", "HCS"),
                              rho = 0,
                              phi = NA,
                              method = "REML",
                              mods = ~ factor(Cell) - 1,
                              time = TRUE,
                              sparse = TRUE,
                              control = list(optimizer = "optimParallel",
                                             ncpus = ncores))
```

```
## 
## Processing time: 0 hours, 0 minutes, 27.24 seconds
```

```
## Model summary
summary(tas20.mlmvrem1.senu)
```

```
## 
## Multivariate Meta-Analysis Model (k = 880; method: REML)
## 
##     logLik    Deviance         AIC         BIC        AICc   
##   829.8610  -1659.7220  -1597.7220  -1449.8987  -1595.3545   
## 
## Variance Components:
## 
## outer factor: ESID         (nlvls = 880)
## inner factor: factor(Cell) (nlvls = 10)
## 
##              estim    sqrt  k.lvl  fixed    level 
## tau^2.1     0.0013  0.0354     88     no  I11-I12 
## tau^2.2     0.0014  0.0375     88     no  I11-I17 
## tau^2.3     0.0011  0.0334     88     no  I12-I17 
## tau^2.4     0.0018  0.0420     88     no   I2-I11 
## tau^2.5     0.0017  0.0415     88     no   I2-I12 
## tau^2.6     0.0025  0.0504     88     no   I2-I17 
## tau^2.7     0.0115  0.1072     88     no    I2-I4 
## tau^2.8     0.0035  0.0589     88     no   I4-I11 
## tau^2.9     0.0038  0.0615     88     no   I4-I12 
## tau^2.10    0.0039  0.0624     88     no   I4-I17 
## rho         0.0000                   yes          
## 
## outer factor: StudyID      (nlvls = 62)
## inner factor: factor(Cell) (nlvls = 10)
## 
##                estim    sqrt  k.lvl  fixed    level 
## gamma^2.1     0.0106  0.1029     88     no  I11-I12 
## gamma^2.2     0.0099  0.0996     88     no  I11-I17 
## gamma^2.3     0.0139  0.1177     88     no  I12-I17 
## gamma^2.4     0.0093  0.0966     88     no   I2-I11 
## gamma^2.5     0.0131  0.1146     88     no   I2-I12 
## gamma^2.6     0.0121  0.1102     88     no   I2-I17 
## gamma^2.7     0.0225  0.1499     88     no    I2-I4 
## gamma^2.8     0.0150  0.1226     88     no   I4-I11 
## gamma^2.9     0.0166  0.1289     88     no   I4-I12 
## gamma^2.10    0.0186  0.1365     88     no   I4-I17 
## phi           0.9141                    no          
## 
## Test for Residual Heterogeneity:
## QE(df = 870) = 6548.1258, p-val < .0001
## 
## Test of Moderators (coefficients 1:10):
## QM(df = 10) = 1539.2512, p-val < .0001
## 
## Model Results:
## 
##                      estimate      se     zval    pval   ci.lb   ci.ub      
## factor(Cell)I11-I12    0.3309  0.0148  22.3446  <.0001  0.3019  0.3600  *** 
## factor(Cell)I11-I17    0.3649  0.0145  25.1792  <.0001  0.3365  0.3933  *** 
## factor(Cell)I12-I17    0.2926  0.0165  17.7529  <.0001  0.2603  0.3249  *** 
## factor(Cell)I2-I11     0.4869  0.0143  34.0242  <.0001  0.4589  0.5150  *** 
## factor(Cell)I2-I12     0.3754  0.0164  22.9494  <.0001  0.3433  0.4074  *** 
## factor(Cell)I2-I17     0.4126  0.0162  25.5316  <.0001  0.3809  0.4443  *** 
## factor(Cell)I2-I4      0.4638  0.0232  20.0186  <.0001  0.4184  0.5092  *** 
## factor(Cell)I4-I11     0.3761  0.0179  21.0242  <.0001  0.3410  0.4112  *** 
## factor(Cell)I4-I12     0.3006  0.0187  16.0667  <.0001  0.2640  0.3373  *** 
## factor(Cell)I4-I17     0.3378  0.0196  17.2227  <.0001  0.2993  0.3762  *** 
## 
## ---
## Signif. codes:  0 '***' 0.001 '**' 0.01 '*' 0.05 '.' 0.1 ' ' 1
```

```
outztor(tas20.mlmvrem1.senu)
```

```
##                      Estimate         SE   CI95low   CI95upp
## factor(Cell)I11-I12 0.3193682 0.01480978 0.2930637 0.3451895
## factor(Cell)I11-I17 0.3495217 0.01449109 0.3243445 0.3742040
## factor(Cell)I12-I17 0.2845641 0.01648260 0.2546070 0.3139754
## factor(Cell)I2-I11  0.4517761 0.01431029 0.4291710 0.4738157
## factor(Cell)I2-I12  0.3586911 0.01635555 0.3304415 0.3862986
## factor(Cell)I2-I17  0.3906878 0.01615941 0.3635208 0.4171908
## factor(Cell)I2-I4   0.4331447 0.02316235 0.3955443 0.4692956
## factor(Cell)I4-I11  0.3593231 0.01788739 0.3284109 0.3894663
## factor(Cell)I4-I12  0.2918907 0.01870926 0.2579939 0.3250698
## factor(Cell)I4-I17  0.3254932 0.01960989 0.2907080 0.3594191
```

```
## Cluster-robust standard errors
summary(
  robust(tas20.mlmvrem1.senu,
         cluster = StudyID,
         clubSandwich = TRUE)
)
```

```
## 
## Multivariate Meta-Analysis Model (k = 880; method: REML)
## 
##     logLik    Deviance         AIC         BIC        AICc   
##   829.8610  -1659.7220  -1597.7220  -1449.8987  -1595.3545   
## 
## Variance Components:
## 
## outer factor: ESID         (nlvls = 880)
## inner factor: factor(Cell) (nlvls = 10)
## 
##              estim    sqrt  k.lvl  fixed    level 
## tau^2.1     0.0013  0.0354     88     no  I11-I12 
## tau^2.2     0.0014  0.0375     88     no  I11-I17 
## tau^2.3     0.0011  0.0334     88     no  I12-I17 
## tau^2.4     0.0018  0.0420     88     no   I2-I11 
## tau^2.5     0.0017  0.0415     88     no   I2-I12 
## tau^2.6     0.0025  0.0504     88     no   I2-I17 
## tau^2.7     0.0115  0.1072     88     no    I2-I4 
## tau^2.8     0.0035  0.0589     88     no   I4-I11 
## tau^2.9     0.0038  0.0615     88     no   I4-I12 
## tau^2.10    0.0039  0.0624     88     no   I4-I17 
## rho         0.0000                   yes          
## 
## outer factor: StudyID      (nlvls = 62)
## inner factor: factor(Cell) (nlvls = 10)
## 
##                estim    sqrt  k.lvl  fixed    level 
## gamma^2.1     0.0106  0.1029     88     no  I11-I12 
## gamma^2.2     0.0099  0.0996     88     no  I11-I17 
## gamma^2.3     0.0139  0.1177     88     no  I12-I17 
## gamma^2.4     0.0093  0.0966     88     no   I2-I11 
## gamma^2.5     0.0131  0.1146     88     no   I2-I12 
## gamma^2.6     0.0121  0.1102     88     no   I2-I17 
## gamma^2.7     0.0225  0.1499     88     no    I2-I4 
## gamma^2.8     0.0150  0.1226     88     no   I4-I11 
## gamma^2.9     0.0166  0.1289     88     no   I4-I12 
## gamma^2.10    0.0186  0.1365     88     no   I4-I17 
## phi           0.9141                    no          
## 
## Test for Residual Heterogeneity:
## QE(df = 870) = 6548.1258, p-val < .0001
## 
## Number of estimates:   880
## Number of clusters:    62
## Estimates per cluster: 10-40 (mean: 14.19, median: 10)
## 
## Test of Moderators (coefficients 1:10):¹
## F(df1 = 10, df2 = 45.3) = 113.4940, p-val < .0001
## 
## Model Results:
## 
##                      estimate      se¹     tval¹     df¹    pval¹   ci.lb¹ 
## factor(Cell)I11-I12    0.3309  0.0157   21.1407   59.85   <.0001   0.2996  
## factor(Cell)I11-I17    0.3649  0.0151   24.1531   59.72   <.0001   0.3347  
## factor(Cell)I12-I17    0.2926  0.0166   17.6181   60.24   <.0001   0.2594  
## factor(Cell)I2-I11     0.4869  0.0153   31.9085   59.55   <.0001   0.4564  
## factor(Cell)I2-I12     0.3754  0.0167   22.5320   60.12   <.0001   0.3421  
## factor(Cell)I2-I17     0.4126  0.0166   24.8749    59.9   <.0001   0.3794  
## factor(Cell)I2-I4      0.4638  0.0235   19.7222   59.84   <.0001   0.4167  
## factor(Cell)I4-I11     0.3761  0.0178   21.1489   60.11   <.0001   0.3405  
## factor(Cell)I4-I12     0.3006  0.0190   15.8490   60.19   <.0001   0.2627  
## factor(Cell)I4-I17     0.3378  0.0200   16.8947   60.32   <.0001   0.2978  
##                       ci.ub¹      
## factor(Cell)I11-I12  0.3623   *** 
## factor(Cell)I11-I17  0.3951   *** 
## factor(Cell)I12-I17  0.3259   *** 
## factor(Cell)I2-I11   0.5175   *** 
## factor(Cell)I2-I12   0.4087   *** 
## factor(Cell)I2-I17   0.4458   *** 
## factor(Cell)I2-I4    0.5108   *** 
## factor(Cell)I4-I11   0.4117   *** 
## factor(Cell)I4-I12   0.3386   *** 
## factor(Cell)I4-I17   0.3778   *** 
## 
## ---
## Signif. codes:  0 '***' 0.001 '**' 0.01 '*' 0.05 '.' 0.1 ' ' 1
## 
## 1) results based on cluster-robust inference (var-cov estimator: CR2,
##    approx t/F-tests and confidence intervals, df: Satterthwaite approx)
```

```
## Extract the fit indices
### Create a new model fit data frame
newfit.mlmvrem1.sent <- data.frame(
  c("logLik", "parms", "k",
    "Deviance", 
    "AIC", "BIC", "AICc"),
  c(## logLik
    tas20.mlmvrem1.sent$fit.stats$REML[1],
    ## Number of parameters (parms)
    tas20.mlmvrem1.sent$parms,
    ## Number of effect sizes (k)
    length(tas20$Correlation),
    ## Deviance
    tas20.mlmvrem1.sent$fit.stats$REML[2],
    ## AIC
    tas20.mlmvrem1.sent$fit.stats$REML[3],
    ## BIC
    tas20.mlmvrem1.sent$fit.stats$REML[4],
    ## AICc
    tas20.mlmvrem1.sent$fit.stats$REML[5]))

colnames(newfit.mlmvrem1.sent) <- c("Fit criterion", "mlmvrem1.sent")
newfit.mlmvrem1.sent
```

```
##   Fit criterion mlmvrem1.sent
## 1        logLik      825.8439
## 2         parms       30.0000
## 3             k      880.0000
## 4      Deviance    -1651.6878
## 5           AIC    -1591.6878
## 6           BIC    -1448.6330
## 7          AICc    -1589.4709
```

```
### Create a new model fit data frame
newfit.mlmvrem2.sent <- data.frame(
  c("logLik", "parms", "k",
    "Deviance", 
    "AIC", "BIC", "AICc"),
  c(## logLik
    tas20.mlmvrem2.sent$fit.stats$REML[1],
    ## Number of parameters (parms)
    tas20.mlmvrem2.sent$parms,
    ## Number of effect sizes (k)
    length(tas20$Correlation),
    ## Deviance
    tas20.mlmvrem2.sent$fit.stats$REML[2],
    ## AIC
    tas20.mlmvrem2.sent$fit.stats$REML[3],
    ## BIC
    tas20.mlmvrem2.sent$fit.stats$REML[4],
    ## AICc
    tas20.mlmvrem2.sent$fit.stats$REML[5]))

colnames(newfit.mlmvrem2.sent) <- c("Fit criterion", "mlmvrem2.sent")
newfit.mlmvrem2.sent
```

```
##   Fit criterion mlmvrem2.sent
## 1        logLik      817.8745
## 2         parms       21.0000
## 3             k      880.0000
## 4      Deviance    -1635.7489
## 5           AIC    -1593.7489
## 6           BIC    -1493.6106
## 7          AICc    -1592.6593
```

```
### Create a new model fit data frame
newfit.mlmvrem3.sent <- data.frame(
  c("logLik", "parms", "k",
    "Deviance", 
    "AIC", "BIC", "AICc"),
  c(## logLik
    tas20.mlmvrem3.sent$fit.stats$REML[1],
    ## Number of parameters (parms)
    tas20.mlmvrem3.sent$parms,
    ## Number of effect sizes (k)
    length(tas20$Correlation),
    ## Deviance
    tas20.mlmvrem3.sent$fit.stats$REML[2],
    ## AIC
    tas20.mlmvrem3.sent$fit.stats$REML[3],
    ## BIC
    tas20.mlmvrem3.sent$fit.stats$REML[4],
    ## AICc
    tas20.mlmvrem3.sent$fit.stats$REML[5]))

colnames(newfit.mlmvrem3.sent) <- c("Fit criterion", "mlmvrem3.sent")
newfit.mlmvrem3.sent
```

```
##   Fit criterion mlmvrem3.sent
## 1        logLik       801.634
## 2         parms        21.000
## 3             k       880.000
## 4      Deviance     -1603.268
## 5           AIC     -1561.268
## 6           BIC     -1461.130
## 7          AICc     -1560.178
```

```
### Create a new model fit data frame
newfit.mlmvrem4.sent <- data.frame(
  c("logLik", "parms", "k",
    "Deviance", 
    "AIC", "BIC", "AICc"),
  c(## logLik
    tas20.mlmvrem4.sent$fit.stats$REML[1],
    ## Number of parameters (parms)
    tas20.mlmvrem4.sent$parms,
    ## Number of effect sizes (k)
    length(tas20$Correlation),
    ## Deviance
    tas20.mlmvrem4.sent$fit.stats$REML[2],
    ## AIC
    tas20.mlmvrem4.sent$fit.stats$REML[3],
    ## BIC
    tas20.mlmvrem4.sent$fit.stats$REML[4],
    ## AICc
    tas20.mlmvrem4.sent$fit.stats$REML[5]))

colnames(newfit.mlmvrem4.sent) <- c("Fit criterion", "mlmvrem4.sent")
newfit.mlmvrem4.sent
```

```
##   Fit criterion mlmvrem4.sent
## 1        logLik      778.7508
## 2         parms       12.0000
## 3             k      880.0000
## 4      Deviance    -1557.5017
## 5           AIC    -1533.5017
## 6           BIC    -1476.2798
## 7          AICc    -1533.1376
```

```
## Overview of the model fit
newfit.overview.sent <- cbind.data.frame(newfit.mlmvrem1.sent,
                                    newfit.mlmvrem2.sent$mlmvrem2.sent,
                                    newfit.mlmvrem3.sent$mlmvrem3.sent,
                                    newfit.mlmvrem4.sent$mlmvrem4.sent)

colnames(newfit.overview.sent) <- c("Fit criterion",
                               "Model 1",
                               "Model 2",
                               "Model 3",
                               "Model 4")

newfit.overview.sent
```

```
##   Fit criterion    Model 1    Model 2   Model 3    Model 4
## 1        logLik   825.8439   817.8745   801.634   778.7508
## 2         parms    30.0000    21.0000    21.000    12.0000
## 3             k   880.0000   880.0000   880.000   880.0000
## 4      Deviance -1651.6878 -1635.7489 -1603.268 -1557.5017
## 5           AIC -1591.6878 -1593.7489 -1561.268 -1533.5017
## 6           BIC -1448.6330 -1493.6106 -1461.130 -1476.2798
## 7          AICc -1589.4709 -1592.6593 -1560.178 -1533.1376
```

```
## Additional comparisons
anova(tas20.mlmvrem1.sent, tas20.mlmvrem2.sent)
```

```
## 
##         df        AIC        BIC       AICc   logLik     LRT   pval        QE 
## Full    30 -1591.6878 -1448.6330 -1589.4709 825.8439                6548.1258 
## Reduced 21 -1593.7489 -1493.6106 -1592.6593 817.8745 15.9389 0.0682 6548.1258
```

```
## Result: Model 1 is preferred over Model 2.

anova(tas20.mlmvrem1.sent, tas20.mlmvrem3.sent)
```

```
## 
##         df        AIC        BIC       AICc   logLik     LRT   pval        QE 
## Full    30 -1591.6878 -1448.6330 -1589.4709 825.8439                6548.1258 
## Reduced 21 -1561.2679 -1461.1296 -1560.1783 801.6340 48.4199 <.0001 6548.1258
```

```
## Result: Model 1 is preferred over Model 3.

anova(tas20.mlmvrem1.sent, tas20.mlmvrem4.sent)
```

```
## 
##         df        AIC        BIC       AICc   logLik     LRT   pval        QE 
## Full    30 -1591.6878 -1448.6330 -1589.4709 825.8439                6548.1258 
## Reduced 12 -1533.5017 -1476.2798 -1533.1376 778.7508 94.1861 <.0001 6548.1258
```

```
## Result: Model 1 is preferred over Model 4.
```

`Model 2` is favored over the other models (given the AIC
and outcomes of LRTs).

# R session info

```
sessionInfo()
```

```
## R version 4.4.3 (2025-02-28)
## Platform: x86_64-apple-darwin20
## Running under: macOS Ventura 13.7.4
## 
## Matrix products: default
## BLAS:   /Library/Frameworks/R.framework/Versions/4.4-x86_64/Resources/lib/libRblas.0.dylib 
## LAPACK: /Library/Frameworks/R.framework/Versions/4.4-x86_64/Resources/lib/libRlapack.dylib;  LAPACK version 3.12.0
## 
## locale:
## [1] en_US.UTF-8/en_US.UTF-8/en_US.UTF-8/C/en_US.UTF-8/en_US.UTF-8
## 
## time zone: Europe/Oslo
## tzcode source: internal
## 
## attached base packages:
## [1] parallel  stats     graphics  grDevices utils     datasets  methods  
## [8] base     
## 
## other attached packages:
##  [1] lattice_0.22-6      semPlot_1.1.6       optimParallel_1.0-2
##  [4] dplyr_1.1.4         corrplot_0.92       clubSandwich_0.5.10
##  [7] robumeta_2.1        metafor_4.6-0       numDeriv_2016.8-1.1
## [10] metadat_1.2-0       Matrix_1.7-2        metaSEM_1.4.0      
## [13] OpenMx_2.21.11      psych_2.4.3         pacman_0.5.1       
## 
## loaded via a namespace (and not attached):
##  [1] mnormt_2.1.1       pbapply_1.7-2      gridExtra_2.3      fdrtool_1.2.17    
##  [5] sandwich_3.1-0     rlang_1.1.4        magrittr_2.0.3     rockchalk_1.8.157 
##  [9] compiler_4.4.3     png_0.1-8          vctrs_0.6.5        reshape2_1.4.4    
## [13] quadprog_1.5-8     stringr_1.5.1      pkgconfig_2.0.3    fastmap_1.2.0     
## [17] arm_1.14-4         backports_1.5.0    pbivnorm_0.6.0     utf8_1.2.4        
## [21] rmarkdown_2.29     nloptr_2.0.3       xfun_0.49          cachem_1.1.0      
## [25] kutils_1.73        jsonlite_1.8.9     jpeg_0.1-10        lavaan_0.6-18     
## [29] cluster_2.1.8      R6_2.5.1           bslib_0.8.0        stringi_1.8.4     
## [33] boot_1.3-31        rpart_4.1.24       jquerylib_0.1.4    Rcpp_1.0.13-1     
## [37] knitr_1.49         zoo_1.8-12         base64enc_0.1-3    splines_4.4.3     
## [41] nnet_7.3-20        igraph_2.0.3       tidyselect_1.2.1   rstudioapi_0.17.1 
## [45] abind_1.4-5        yaml_2.3.10        codetools_0.2-20   qgraph_1.9.8      
## [49] tibble_3.2.1       plyr_1.8.9         coda_0.19-4.1      evaluate_1.0.1    
## [53] foreign_0.8-88     RcppParallel_5.1.8 zip_2.3.1          pillar_1.9.0      
## [57] carData_3.0-5      checkmate_2.3.1    stats4_4.4.3       ellipse_0.5.0     
## [61] generics_0.1.3     mathjaxr_1.6-0     ggplot2_3.5.1      munsell_0.5.1     
## [65] scales_1.3.0       minqa_1.2.6        gtools_3.9.5       xtable_1.8-4      
## [69] glue_1.8.0         mi_1.1             Hmisc_5.1-3        tools_4.4.3       
## [73] data.table_1.16.4  lme4_1.1-35.3      openxlsx_4.2.6.1   mvtnorm_1.3-2     
## [77] XML_3.99-0.17      grid_4.4.3         sem_3.1-15         colorspace_2.1-1  
## [81] nlme_3.1-167       htmlTable_2.4.3    Formula_1.2-5      cli_3.6.3         
## [85] fansi_1.0.6        corpcor_1.6.10     glasso_1.11        gtable_0.3.6      
## [89] sass_0.4.9         digest_0.6.37      htmlwidgets_1.6.4  htmltools_0.5.8.1 
## [93] lifecycle_1.0.4    lisrelToR_0.3      MASS_7.3-64
```
